# Enantioselective Synthesis of the Cyclopiazonic Acid Family Using Sulfur Ylides

**DOI:** 10.1002/anie.201712065

**Published:** 2018-01-09

**Authors:** Oleksandr Zhurakovskyi, Yunus E. Türkmen, Lorenz E. Löffler, Vijayalakshmi A. Moorthie, C. Chun Chen, Michael A. Shaw, Mark R. Crimmin, Marco Ferrara, Mushtaq Ahmad, Mehrnoosh Ostovar, Johnathan V. Matlock, Varinder K. Aggarwal

**Affiliations:** ^1^ School of Chemistry University of Bristol Cantock's Close Bristol BS8 1TS UK

**Keywords:** (3+2)-cycloaddition, aziridination, sulfur ylide, total synthesis, α-cyclopiazonic acid

## Abstract

A convergent, nine‐step (LLS), enantioselective synthesis of α‐cyclopiazonic acid and related natural products is reported. The route features a) an enantioselective aziridination of an imine with a chiral sulfur ylide; b) a bioinspired (3+2)‐cycloaddition of the aziridine onto an alkene; and c) installation of the acetyltetramic acid by an unprecedented tandem carbonylative lactamization/N−O cleavage of a bromoisoxazole.

Indole alkaloids have long been a source of inspiration for the development of new synthetic methods and strategies. α‐Cyclopiazonic acid (α‐CPA, **1**) is a prenylated indole alkaloid produced by a number of *Penicillium* species including *P. commune*, *P. griseofulvum*, and *P. camemberti*.^1^ It is a potent inhibitor of Ca^2+^‐dependent ATPase (SERCA) which prevents calcium reuptake in muscle.^2^ In addition to its significant biological activity, α‐CPA‐producing fungi are found in cheese, meat, and other dietary products, making it important to the food industry.

Several structurally related natural products have been identified (Figure 1): iso‐α‐cyclopiazonic acid (**2**),^3^ α‐CPA imine (**3**),1b speradines A–D,^4^ and aspergillines A–E,^5^ all sharing a 3‐acetyltetramic acid unit.

**Figure 1:**
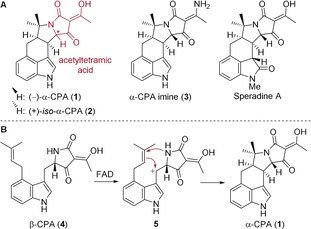
A) α‐CPA and related natural products. B) Biosynthesis of α‐CPA.

Biosynthetically, α‐CPA is derived from l‐tryptophan (Figure 1 B).1a,1b, 6 The tetramic acid is assembled at an early stage followed by several alkylations to give β‐cyclopiazonic acid (β‐CPA, **4**), a direct biosynthetic precursor of α‐CPA. Flavin‐mediated oxidation of β‐CPA and subsequent cyclization give α‐CPA.6f

Four total syntheses of α‐CPA have been published (Figure 2 A).^7^ They all share the same end‐game strategy, in which the tetramic acid unit is installed by a Dieckmann condensation, forming the C6−C7 bond. Kozikowski7a and Natsume7b constructed the C–D rings in a stepwise manner, but with low diastereoselectivity. Knight developed an elegant cationic cascade, in which acyclic precursor **9** was converted into indole **6** with high stereocontrol,7c,7d although Scherkenbeck found that the same substrate cyclized to give a 1:1 mixture of diastereomers across the CD ring junction under slightly different conditions.7e,7f

**Figure 2:**
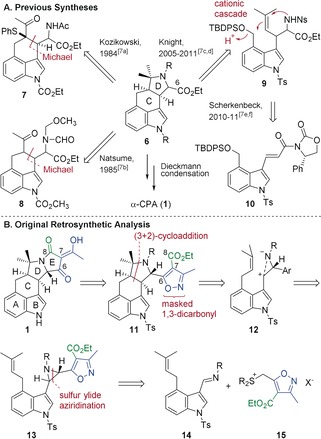
A) Previous syntheses of α‐CPA. B) Our retrosynthetic analysis.

In our retrosynthetic approach to α‐CPA we considered a different, bioinspired strategy (Figure 2 B). We were attracted by the possibility of using an aziridine **13** as a precursor to the zwitterionic intermediate **12** that would participate in a (3+2)‐cycloaddition to construct the C–D ring system. Whilst this type of (3+2)‐cycloaddition has been reported for the construction of pyrrolidines,^8^ its application in total synthesis is much rarer.^9^ Aziridine **13** could be assembled from simple building blocks **14** and **15** using our asymmetric sulfur ylide methodology.^10^ We envisaged using an isoxazole as a masked 1,3‐dicarbonyl group^11^ attached to the sulfur ylide. A further attractive feature of this approach is that the ylide could carry all the carbons and functionality required for making rings C and D. We would then have to build ring E by N−C8 bond formation, rather than the C6−C7 bond, which is commonly used to construct tetramic acids.

We began our synthesis by targeting the imine building block **14** which was obtained in 4 steps from commercially available indole **16** (Scheme 1 A). Suzuki cross‐coupling of aryl bromide **16** with allyl boronic ester followed by *N*‐tosylation gave indole **17**. Cross‐metathesis of the terminal alkene **17** in neat 2‐methyl‐2‐butene^12^ delivered **18** in good yield. Initial attempts to affect a one‐step prenylation of **16** under various conditions led to substantial prenylboration of the aldehyde giving alcohol **19**. The aldehyde **18** was converted into the *N*‐nosyl^13^ imine **14**, thus completing the synthesis of the indole fragment.

**Scheme 1:**
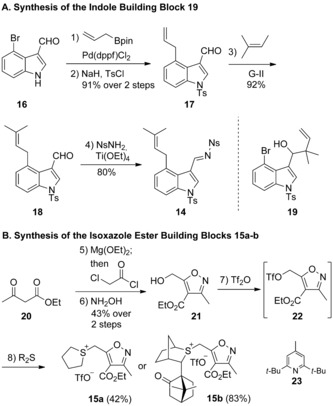
Reagents and conditions: 1) Allyl‐Bpin, Pd(dppf)Cl_2_, KOH, THF‐H_2_O, 65 °C; 2) NaH, TsCl, DMF, 0 to 23 °C, 91 % over 2 steps; 3) 2‐methyl‐2‐butene, Grubbs 2^nd^ gen cat., 23 °C, 92 %; 4) NsNH_2_, Ti(OEt)_4_, CH_2_Cl_2_, 23 °C, 80 %; 5) Mg(OEt)_2_, PhH‐EtOH, 23 °C, then 2‐chloroacetyl chloride, MeCN‐PhH‐EtOH, 0 to 23 °C, 44 %; 6) NH_2_OH⋅HCl, NaOAc, EtOH, reflux, 98 %; 7) Tf_2_O, **23**, CH_2_Cl_2_, 0 °C; 8) R_2_S, Et_2_O, 0 °C, 42 % for **15 a**, 83 % for **15 b**. Pin=pinacolato, dppf=1,1′‐bis(diphenylphosphino)ferrocene, Ts=4‐toluenesulfonyl, Ns=4‐nitrobenzenesulfonyl, Tf=trifluoromethanesulfonyl.

Sulfonium salts **15 a**,**b** were prepared from known alcohol **21** 14 by a two‐step sequence via triflate **22** (Scheme 1 B). The use of the triflate instead of a corresponding bromide resulted in 1) much faster alkylations, and 2) the sulfonium salts precipitating directly from the ethereal solvent, permitting straightforward isolation by simple filtration.^15^

Our initial synthetic campaign was performed with an achiral sulfonium salt **15 a** to evaluate the viability of the route (Scheme 2). Reaction of imine **14** with an ylide derived from **15 a** proceeded smoothly and delivered aziridine **24** in good yield (72 %) and diastereoselectivity (*trans*/*cis* 9:1). *Trans*‐**24** was prone to rapid isomerization into *cis*‐**24** in CDCl_3_ or on silica, and so was used crude. Notably, compound **24** already contains all the carbon atoms present in α‐CPA.

**Scheme 2:**
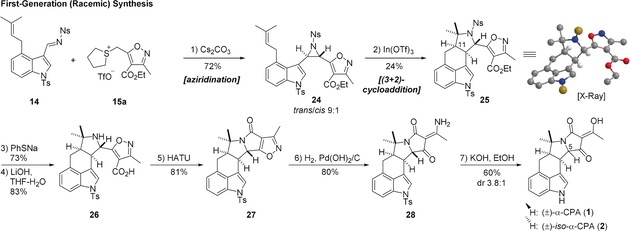
Reagents and conditions: 1) Cs_2_CO_3_, CH_2_Cl_2_, −40 °C, 72 %; 2) In(OTf)_3_, CH_2_Cl_2_, −78 to 23 °C, 24 %; 3) PhSNa, DMF, 23 °C, 73 %; 4) LiOH, THF‐MeOH‐H_2_O, 23 °C, 83 %; 5) HATU, DIPEA, DMF, 23 °C, 81 %; 6) H_2_ (1 atm), Pd(OH)_2_/C, MeOH, 80 %; 7) KOH, EtOH, 65 °C. HATU=*N*‐[(dimethylamino)‐1*H*‐1,2,3‐triazolo‐[4,5‐b]pyridin‐1‐ylmethylene]‐*N*‐methylmethanaminium hexafluorophosphate *N*‐oxide, DIPEA= *N*,*N*‐diisopropylethylamine.

We then explored the bioinspired cycloaddition of **24** and tested a range of Lewis and Brønsted acids (see the Supporting Information, SI), and found that treatment of CH_2_Cl_2_ solutions of **24** with 2 equiv of In(OTf)_3_ or 0.1–1 equiv of TfOH triggered the desired reaction. This gave pyrrolidine **25** as a mixture of diastereomers at C‐11 (d.r. 3:1, in favor of the desired *cis*‐isomer), from which the desired *cis* product was isolated as a single isomer in 24 % yield by crystallization from MeCN–H_2_O. The nosyl group was removed with PhSNa and the ester was hydrolyzed with LiOH yielding amino acid **26**. Subjecting **26** to a standard amide coupling conditions (HATU, DIPEA, DMF) resulted in formation of lactam **27**. Subsequent hydrogenolysis of the N−O bond under Pd catalysis gave *N*‐Ts α‐CPA imine **28** in 80 % yield, which was then hydrolyzed7b to give (±)‐α‐CPA (**1**) in 60 % yield (dr 3.8:1). The racemic synthesis of **1** was thus achieved in 11 steps (longest linear sequence).

Unexpectedly, the attempted enantioselective campaign met with failure. The use of the chiral sulfonium salt **15 b** gave the desired aziridine **24** but with poor diastereo‐ and enantioselectivity (dr 1:0.9, er 40:60). We believe that the ylide derived from sulfonium salt **15 b** behaves as a stabilized rather than a semi‐stabilized ylide and so reacts reversibly with the imine **14**, resulting in low stereocontrol.10a,10c We therefore considered alternative isoxazole substrates **31 a**–**c** (Scheme 3) bearing a less anion‐stabilizing group (bromide in place of the ester). The bromine atom could also conveniently serve as a handle for a Pd‐mediated carbonylative coupling.

**Scheme 3:**
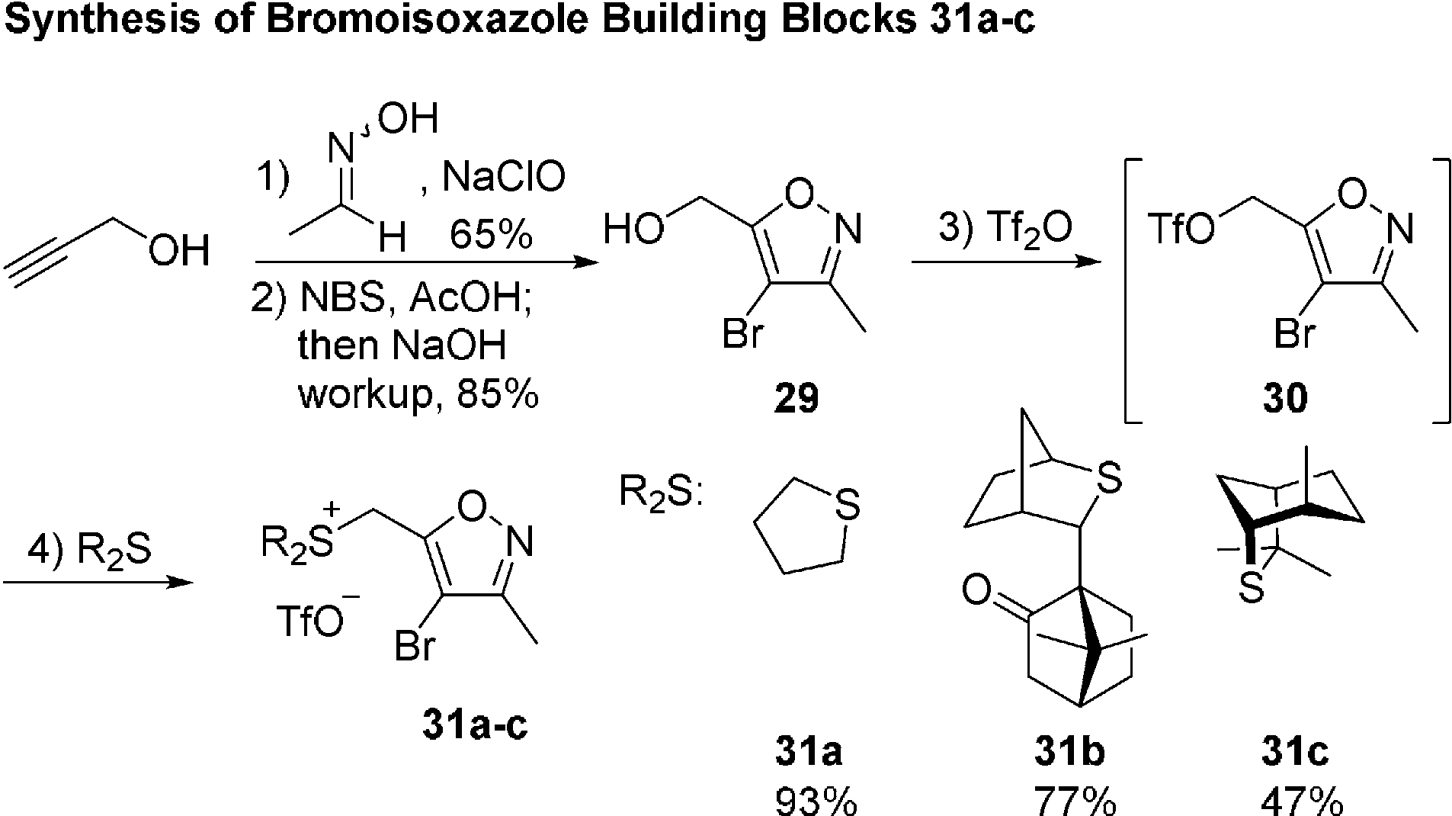
Reagents and conditions 1) acetaldoxime, NaClO, CH_2_Cl_2_‐H_2_O, 0 to 23 °C, 65 %; 2) NBS, H_2_SO_4_, AcOH, 110 °C, then NaOH workup, 85 %; 3) Tf_2_O, **23**, CH_2_Cl_2_, 0 °C; 4) R_2_S, Et_2_O, 0 °C, 93 % for **31 a**, 77 % for **31 b**, 47 % for **31 c**. NBS=*N*‐bromosuccinimide.

Our second‐generation synthesis of α‐CPA began with the synthesis of bromoisoxazole sulfonium salts **31 a**–**c** as shown in Scheme 3. Alcohol **29** was prepared in 2 steps from propargyl alcohol using a modified literature procedure.^16^ Triflation of **29** followed by the nucleophilic substitution with a range of sulfides delivered the desired salts **31 a**–**c** in moderate to excellent yields.

Aziridination of imine **14** with ylides derived from **31 a**–**c** afforded aziridine **32** in good yields, and as before, under exceptionally mild conditions (Scheme 4). The camphor‐derived salt **31 b** performed better than the isothiocineole‐derived salt **31 c** giving the aziridine **32** with good diastereoselectivity (*trans*/*cis* 9:1) and excellent enantioselectivity (er 98:2 for *trans*, 89:11 for *cis*). The high enantioselectivity provided validation of our hypothesis: the ylide with the less electron‐withdrawing bromine atom is now behaving as a semi‐stabilized ylide, rendering betaine formation the enantiodetermining step.

**Scheme 4:**
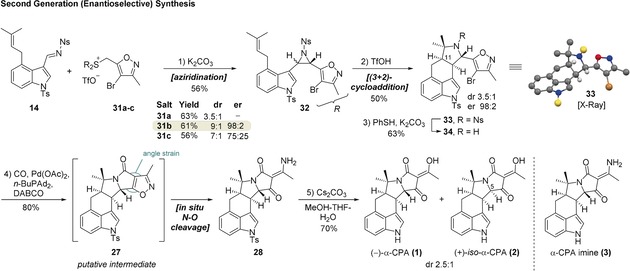
Reagents and conditions: 1) K_2_CO_3_, MeCN, −20 °C, 56 % (*trans*/*cis* 9:1, er 98:2 [*trans*], 89:11 [*cis*]); 2) TfOH, CH_2_Cl_2_, −55 to 10 °C, 50 % (dr 3.5:1, er 98:2); 3) PhSH, K_2_CO_3_, 18‐crown‐6, MeCN, 23 °C, 63 %; 4) CO (1 atm), Pd(OAc)_2_, *n*‐BuPAd_2_, DABCO, DMSO, 120 °C, 80 %; 5) Cs_2_CO_3_, MeOH‐THF‐H_2_O, 65 °C, 70 %. Ad=adamantyl, DABCO=1,4‐diazabicyclo[2.2.2]octane.

As with aziridine **24**, *trans*‐aziridine **32** was prone to isomerization into *cis*‐**32**, and thus was used without purification. Treatment of crude **32** with TfOH gave pyrrolidine **33** as a 3.5:1 mixture of diastereomers^17^ at C‐11 in favor of the desired *cis*‐isomer, in 50 % yield with complete enantiospecificity (er 98:2).^18^ Deprotection of the diastereomeric mixture with PhSH/K_2_CO_3_ gave amine **34**, at which point the diastereomers were separated. We were initially concerned about the next Pd‐catalyzed carbonylation‐amide formation due to the severe angle strain inherent in the fused bicyclic isoxazole **27**.^19^ However, we were delighted to find that treatment of **34** with Pd(OAc)_2_ under an atmosphere of CO in the presence of DABCO and *n*‐BuPAd_2_ 20 triggered a reaction cascade leading *directly* to the formation of *N*‐Ts α‐CPA imine **28** in 80 % yield. The cascade involves palladium‐catalyzed carbonylation, acylation, followed by reduction of the N−O bond in situ,^21^ facilitated by the inherent angle strain of the fused unsaturated ring system **27**. Presumably, the facility of the cyclization stems from ready formation of the undistorted amino‐acyl palladium intermediate, before ring strain is introduced through the subsequent reductive elimination. Hydrolysis of *N*‐Ts species **28** under basic conditions7e in MeOH‐THF‐H_2_O (10:10:1) provided a mixture of (−)‐α‐CPA (**1**) and (+)‐iso‐α‐CPA (**2**) (dr 2.5:1) which was separated by reverse‐phase prep‐HPLC.

Synthetic (−)‐α‐CPA was identical in all respects to the natural material, including TLC, LCMS, HRMS, NMR and optical rotation^3^ data (see SI). When the reaction was performed under strictly anhydrous conditions, α‐CPA imine (**3**) was the major product. This constitutes the first direct synthesis of α‐CPA imine: the previous method relied on the amination of α‐CPA itself.7e This completed our synthesis of the α‐CPA family.

In summary, we have achieved an enantioselective total synthesis of (−)‐α‐CPA and (+)‐iso‐α‐CPA in 9 steps (LLS) from commercially available materials (13 total steps). The route is convergent with the key asymmetric aziridination bringing together the two halves of the molecule with high stereoselectivity and with all the functionality required to complete the target. Additional features of the sequence include 1) a bio‐inspired intramolecular alkene–aziridine (3+2)‐cycloaddition to assemble a polysubstituted pyrrolidine; and 2) a one‐pot carbonylative lactamization/isoxazole cleavage to give an acetyltetramic acid. The latter represents a novel route to tetramic acids which could have broader applications in synthesis.

*In memory of Gilbert Stork*

## Conflict of interest

The authors declare no conflict of interest.

## Supporting information

As a service to our authors and readers, this journal provides supporting information supplied by the authors. Such materials are peer reviewed and may be re‐organized for online delivery, but are not copy‐edited or typeset. Technical support issues arising from supporting information (other than missing files) should be addressed to the authors.

Supplementary
