# Supplementary material for: Enantioselective Synthesis of the Cyclopiazonic Acid Family Using Sulfur Ylides
Source: Angew Chem Int Ed Engl. 2018 Jan 9;57(5):1346–50. doi: 10.1002/anie.201712065 (PMC5817397; doi:10.1002/anie.201712065)
Supplement: Supplementary file 1 — Supplementary [file ANIE-57-1346-s001.pdf]

Angewandte Chemie
Eine Zeitschrift der Gesellschaft Deutscher Chemiker

Supporting Information

Enantioselective Synthesis of the Cyclopiazonic Acid Family Using Sulfur Ylides

*Oleksandr Zhurakovskyi, Yunus E. Türkmen, Lorenz E. Löffler, Vijayalakshmi A. Moorthie, C. Chun Chen, Michael A. Shaw, Mark R. Crimmin, Marco Ferrara, Mushtaq Ahmad, Mehrnoosh Ostovar, Johnathan V. Matlock, and Varinder K. Aggarwal**

anie_201712065_sm_miscellaneous_information.pdf

Table of Contents

Synthetic Overview 2
General Experimental 3
Optimization of Key Reactions... 5
Synthetic Procedures 11
References 47
NMR Spectra... 48

Synthetic Overview

Indole Building Block 14

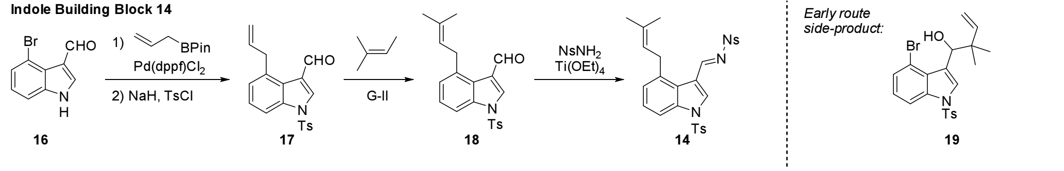

Synthesis of the Isoxazole Ester Building Blocks 15a-b

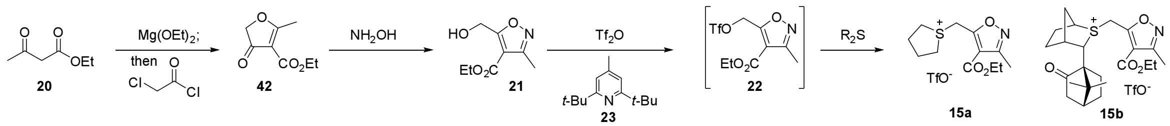

First-Generation (Racemic) Synthesis

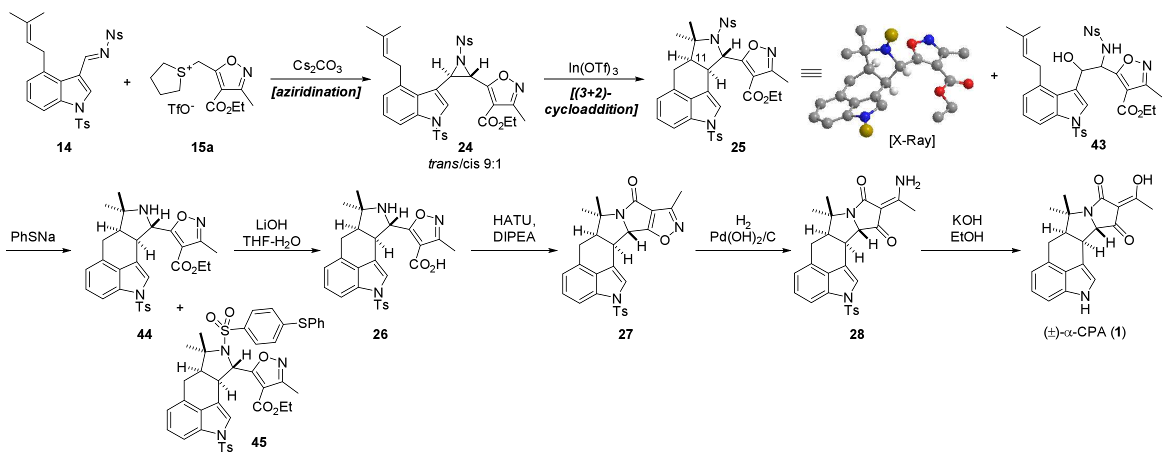

Synthesis of Bromoisoxazole Building Blocks 31a-c

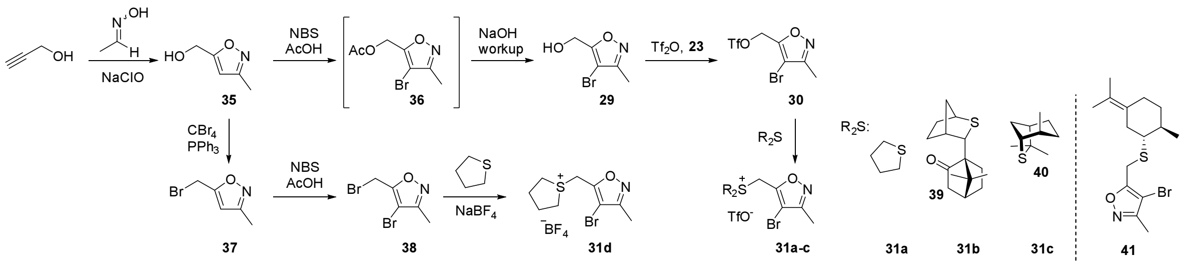

Second Generation (Enantioselective) Synthesis

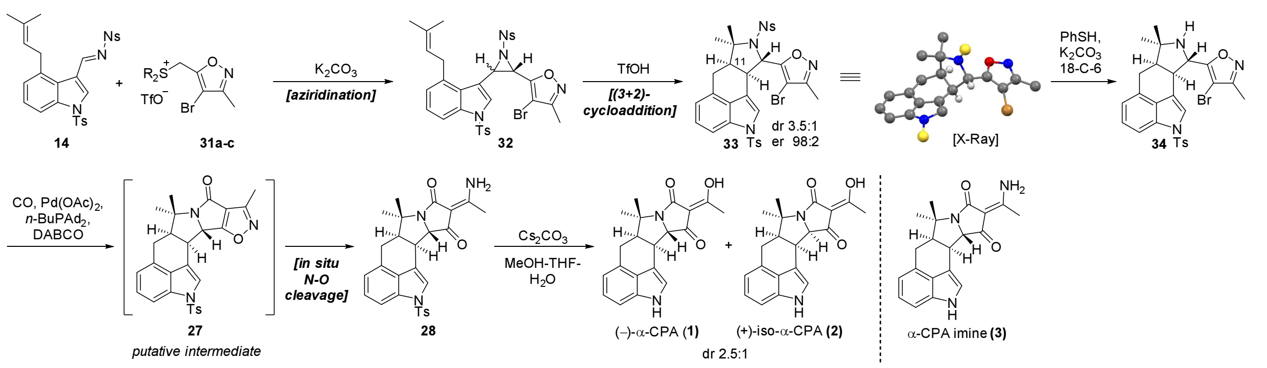

General Experimental

Unless indicated otherwise, reactions were performed in oven- or flame-dried glassware under nitrogen. Reactions were monitored by thin-layer chromatography (TLC) or reverse-phase LCMS where appropriate. Purification was performed using standard[1] flash chromatography on silica gel, reverse-phase preparative HPLC, or recrystallization. The products were analyzed using LCMS, NMR, and HRMS where appropriate.

TLC

TLC analysis was performed using Merck aluminum-backed plates pre-coated with silica gel (Silica Gel 60 F_{254}). The plates were visualized under UV light and stained with $KMnO_4$ or anisaldehyde stain.

LCMS

Reverse-phase LCMS traces were obtained on Agilent 1260 Infinity II system with Agilent PoroShell 120 EC-C18 column (3.0×50 mm, 2.7 μm) using acetonitrile-water gradients (50→90% or 10→90% MeCN-H_2O), and observing at 254 or 214 nm.

Silica Chromatography

Flash column chromatography was performed using standard conditions[1] on silica gel (Aldrich, 60 Å F_{254} 230-400 mesh, 40-63 μm) or on Biotage Isolera One automated system, as indicated.

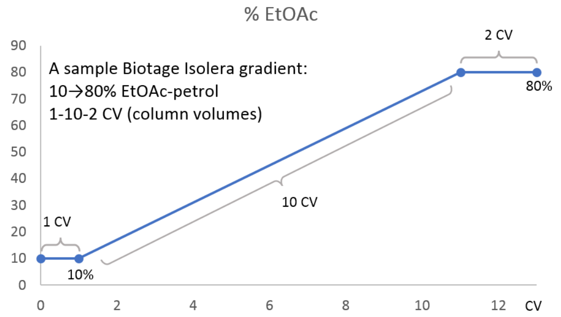

Prep-HPLC

Where necessary, reverse-phase preparative HPLC was performed on Waters AutoPurification system with ACE 5 C18 columns (250×4.6 mm for method development, 250×21.2 mm for preparative runs), using acetonitrile-water gradients.

NMR

Routine NMR spectra were recorded on Varian, Bruker and JEOL spectrometers at 400 MHz for ^{1}H and 100 MHz for ^{13}C spectra. High-resolution spectra were run on Bruker Cryocarbon 500 spectrometer (500 MHz for ^{1}H and 125 MHz for ^{13}C). Signals are reported relative to the residual signal of the non-deuterated solvent ($CDCl_3$: δ = 7.26 ppm, CD_3CN: δ = 1.94 ppm for ^{1}H spectra; and $CDCl_3$: δ = 77.16 ppm, CD_3CN: δ = 118.26 ppm for ^{13}C spectra). ^{1}H NMR data are reported as follows: integration, chemical shift (parts per million, ppm), multiplicity (s = singlet, d = doublet, t = triplet, q = quartet, dd = doublet of doublets, m = multiplet, br = broad, *app* = apparent), coupling constant (Hz) and description.

IR

Infrared spectra were recorded on a PerkinElmer Spectrum One ATR FT-IR: spectrometer as thin films. Only selected peaks are reported. Data represented as follows: frequency of absorption (cm^{-1}), and intensity of absorption (s = strong, m = medium, w = weak, br = broad.

HRMS

High-resolution mass spectra (HRMS) were recorded by the University of Bristol Spectrometry Services Laboratory using electrospray ionization (ESI; Bruker micrOTOF II) and chemical ionization (CI; VG AutoSpec) techniques.

Melting Points

Melting points were determined using a Reichert hot stage apparatus with a digital thermometer.

SFC

Chiral supercritical fluid chromatography (SFC) was performed on a Waters TharSFC system using Whelk-O 1 column (4.6×250 mm, 5 μm), and monitored using a diode array detector (DAD).

Crystallography

X-ray diffraction experiments on compounds **25** and **33** were carried out at 100(2) K on a Bruker APEX II CCD diffractometer using Mo-K_α radiation (λ = 0.71073 Å). Intensities were integrated in SAINT[2] and absorption corrections were based on equivalent reflections using SADABS.[3] Structure **25** was solved using Superflip[4,5] while **33** was solved using ShelXT,[6] both of the structures were refined against F^2 in SHELXL[7,8] using Olex2.[9] All of the non-hydrogen atoms were refined anisotropically. All of the hydrogen atoms were located geometrically

and refined using a riding model. In the case of **25** one of the NO_2 group O and the acetonitrile solvent molecules in the lattice displayed disorder, the occupancies of the fragments was determined by refining them against a free variable with the sum of the two sites set to equal 1, the occupancies were then fixed at the refined values. Restraints and constraints were used to maintain sensible geometries and thermal parameters. In **33**, Squeeze within Platon[10,11] was used to remove disordered solvent from the lattice that could not be sensibly modelled. Crystal structure and refinement data are given in Tables 8-9. Crystallographic data for compounds **25** and **33** have been deposited with the Cambridge Crystallographic Data Centre as supplementary publication CCDC 1584104-1584105. Copies of the data can be obtained free of charge on application to CCDC, 12 Union Road, Cambridge CB2 1EZ, UK [fax(+44) 1223 336033, e-mail: deposit@ccdc.cam.ac.uk].

Optical Rotation

Optical rotations of enantioenriched samples were measured on Bellingham+Stanley ADP220 polarimeter (l = 1 dm or 0.25 dm).

Quantitative NMR (qNMR) studies

A stock solution of 50.0 mg dimethylsulfone (MSM, 97%) in $CDCl_3$ (10.0 mL) was prepared, giving the reference concentration of 5.00 mg/mL. The sample to be analyzed (5–25 mg, weighed to ±0.1 mg) was dissolved in this solution (0.70 mL, 3.50 mg of the MSM reference) and placed into NMR tube. A ^{1}H NMR spectrum was acquired. Line broadening was applied at 0.3 Hz, followed by the baseline correction (polynominal fit, 1 Hz filter). The reference peaks were integrated and the purity of the sample was estimated using the following equation:[12]

$$P_{sample} = \frac{I_{sample}}{I_{ref}} \times \frac{N_{ref}}{N_{sample}} \times \frac{MW_{sample}}{MW_{ref}} \times \frac{m_{sample}}{m_{ref}} \times P_{ref},$$

where *sample* and *ref* refer to the sample and reference material parameters, respectively. *I* is the NMR integral of the signal. *N* is the number of protons in the signal. *MW* is the molecular weight. M is the mass. *P* is the purity.

Materials and solvents

Anhydrous tetrahydrofuran (THF), dichloromethane (DCM) and acetonitrile (MeCN) were obtained by passage over activated alumina in a solvent purification system.[13] DMF and DMSO were dried by storing over activated molecular sieves (3 Å, ~20 wt%) under nitrogen.[14] Degassed solvents were obtained by sonication under vacuum, as reported by Buchwald.[15] "Petrol" refers to petroleum ether (40−60 °C).

Unless stated otherwise, all other commercially available reagents were used as received.

4-Bromo-3-formylindole was purchased from Frontier Scientific, Inc. and used as received. $Pd(dppf)Cl_2 \cdot DCM$ was purchased from Strem Chemicals, Inc. and stored at 23 °C in a desiccator. TsCl was recrystallized from hexane and stored at 23 °C under nitrogen. Hoveyda-Grubbs 2nd generation catalyst (97%) and Grubbs 2nd generation catalyst were purchased from Sigma-Aldrich and stored at 4 °C under nitrogen. $Ti(OEt)_4$ (technical grade, 90%) was purchased from Sigma-Aldrich and used as received. $In(OTf)_3$ was purchased from Sigma-Aldrich and stored at 23 °C in under nitrogen. *N*-bromosuccinimide (NBS) was recrystallized from water[16] and stored at −20 °C under nitrogen. Triflic acid was purchased from Sigma-Aldrich and used as a stock solution in anhydrous DCM (0.1 M), stored in a Schlenk tube under nitrogen.

Optimization of Key Reactions

Racemic synthesis of aziridine 24

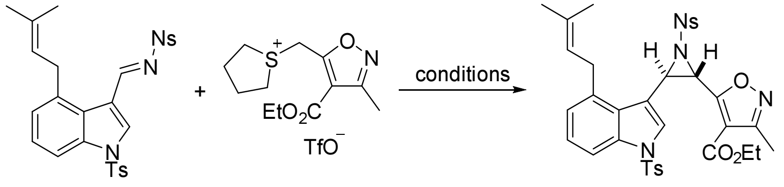

14 **15a** **24**

Table 1. Optimization of aziridination with sulfonium salt **15a**.

#	Conditions	qNMR Yield	*trans/cis*
1	2 eq K_2CO_3, 1.3 eq **15a**, MeCN, 0 °C→23 °C, 20 hr	32%	2.1:1
2	Same as above, 0 °C→23 °C, **4 hr**	40%	4.4:1
3	Same as above, **3×conc**	50%	3.6:1
4	1.2 eq P2 base, 1.2 eq $R_3S^+TfO^-$, DCM, −78 °C, 4 hr	45%	7.7:1
5	1.2 eq KHMDS, 1.2 eq **15a**, THF, −78 °C, 30 min	34%	2.8:1
6	1.2 eq KHMDS, 1.2 eq **15a**, THF, −78 °C, 4 hr	37%	3.3:1
7	2 eq Cs_2CO_3, 1.3 eq **15a**, MeCN, 0 °C, 4 hr	31%	5.7:1
8-1	2 eq Cs_2CO_3, 1.3 eq **15a**, DCM, −40→0 °C, 4 hr	65–67%	7:1
8-2	Scaleup of 8-1 to 300 mg of **15a**	70%	12:1

Note: Reactions performed on 50 mg scale of imine **14**.

(3+2)-Cycloaddition of *cis*-aziridine 24

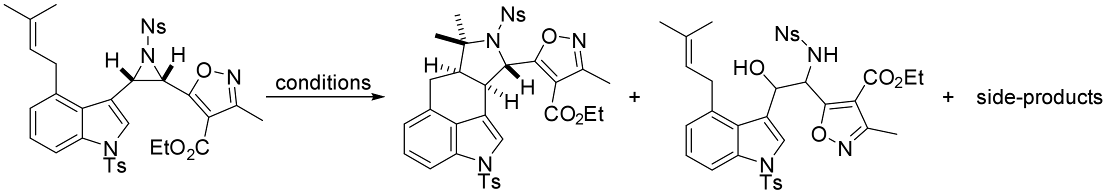

cis-24 9.49-9.54 min | **25** 8.35 min | **43** Side-product 7.50-7.63 min

General procedure

The starting aziridine *cis*-**24** (15 mg, purified by HPLC) was placed into a flame-dried Schlenk tube, followed by naphthalene (internal standard, scintillation grade, 6−7 mg, weighed precisely). The tube was back filled with nitrogen. Anhydrous solvent (1 mL of DCM, unless indicated otherwise) was then added and the mixture was cooled to the starting temperature with dry ice bath (−70 °C, unless stated otherwise). The Lewis/Brønsted acid was then added (solids were added neat, liquids were pre-dissolved in anhydrous DCM to 0.2 M) and the reaction mixture was allowed to slowly warm up, while being stirred in the ice bath (warming rate: −70→−10 °C over 6 hr). Reaction aliquots were sampled as indicated and analyzed by LCMS:

$$\%Conv = \frac{Int_{SM} - Int_{SM}^{0}}{Int_{SM}^{0}},$$

where Int_{SM} is the current integral of the starting material measured vs. internal standard

$$\%TM = \frac{Int_{TM}/Int_{IS}}{Int_{SM}^{0}/Int_{IS}^{0}},$$

where Int_{TM} is the current integral of the target material peak, Int_{IS} is the current integral of the internal standard, Int^0_{SM} is the initial integral of the starting material, and Int^0_{IS} is the initial integral of the internal standard.

Table 2. Optimization of the (3+2)-cycloaddition of *cis*-aziridine

#	Conditions	LCMS conv.	(3+2) Yield		Major side-product	Notes
			LCMS	qNMR		
1	1 eq **TfOH**, DCM, −70 °C, 10 min	95%	30%	37%	**43**	Very rapid reaction at −70 °C.
2	1 eq **$B(C_6F_5)_3$**, DCM, −70→23 °C, 25 hr	100%	10%	8%	**43**	Reaction started at 0 °C and finished at 10 °C. $B(C_6F_5)_3$ insoluble at −70 °C.
3	1 eq **BF_3·OEt_2**, DCM, −70→23 °C, 5 hr	>99%	26%	28%		Reaction started at −30 °C and finished at −5 °C.
4	1 eq **Cu^I(hfacac)-COD**, DCM, −70 →23 °C, 20.5 hr	No reaction (66% SM recovered)			—	—
5	1 eq **$Cu^{II}(hfacac)_2$-H_2O**, DCM, −70 →23 °C, 2 d	50%	0%	—	**43**	Clean and very slow formation of A. Started at 23 °C. DCM self-evaporated. **Prep-HPLC for A (500b, cryo500)**
6	1 eq **$Bi(OTf)_3$**, DCM, −70 →23 °C, 22 hr	100%	20%	21%	Other	Reaction started at −10 °C and finished at 5 °C. $Bi(OTf)_3$ poorly soluble in DCM.
7	1 eq **$Sn(OTf)_2$**, DCM, −73→5 °C, 5 hr	>99%	20%	—	Other	Started at −5 °C, finished at 5 °C.
8	1 eq **$In(OTf)_3$**, DCM, −65→23 °C, 6 hr	100%	25%	25%	Other	Started at −5 °C, ended at 10 °C.
9	**0.1 eq TfOH**, DCM, −70→0 °C, 5 hr	96%	16%	16%	**43** (after −10 °C)	Started at −70 °C, major side-product build-up at −10 °C.
10	**0.3 eq TfOH**, DCM, −70→23 °C, 5 hr	99%	22%	24%	**43**	Begins at −70 °C, rapid at −40 °C, ends at −10 °C.
11	0.3 eq TfOH, **Et_2O**, −70→23 °C, 18 hr	73%	—		**43**	Slow formation of A, no (3+2) at all, SM insoluble in Et_2O.
12	0.3 eq TfOH, **MeCN**, −40→23 °C, 3 d	99%	—		**43**	SM insoluble in MeCN below 0 °C, slow formation of A (begins at 5 °C, ends at 23 °C).
13	1 eq **$Sc(OTf)_3$**, DCM, −70→23 °C, 4.5 hr	99%	26%	25%	**43**	$Sc(OTf)_3$ insoluble at −78°C, dissolves at 23 °C Reaction starts around 23 °C, finished in ~2 hr.
14	0.3 eq TfOH, DCM, **−40→−35 °C**, 15 min	100%	18%	19%	–	NMR much less pure than entry 10.
15	0.3+0.6 eq TfOH, DMF, −50→23 °C, 1 d	0%	—			No reaction.
16	0.3 eq TfOH, $PhCF_3$, −20 °C, 1 min	>95%	11%	10%	**43**	Very rapid and messy reaction.
17	0.3+0.6 eq **HCl**, DCM (+ trace Et_2O), −65→23 °C, 24 hr	29%	—		Other	Very slow formation of a new side-product at 8.93 min.
18	**$CDCl_3$**, 40→57 °C, 23 hr	60%			Other	Slow formation of side-product at 8.37 min.
19	DCM, **SiO_2 (activ.)**, 23 °C, 3 d	37%	—		Other	Trace amount of side-product.
20	**0.22 eq $Pd(OAc)_2$, 0.88 eq PPh_3,** THF, 23 °C, 16 hr	5%			Other	Trace amount of side-prod.
21	1+1 eq **$Cu(OTf)_2$**, DCM, 23 °C, 2 hr	100%	16%		Other	Looks cleaner than TfOH but the yield is still low.
22	2 eq **$Cu(OTf)_2$**, DCM, 23 °C, 6 min	100%	17%		Other	Looks cleaner than TfOH but the yield is still low.
23	0.2 eq **Pd_2dba_3**, + 1 eq *n*-Bu_4Cl, 0.6 eq P(*o*-tolyl)$_3$, DCM, 23 °C, 24 hr	0%			–	No reaction.
24	0.2 eq **Pd_2dba_3,** + 1 eq *n*-Bu_4Cl, 0.6 eq P(*o*-tolyl)$_3$, **THF**, 23 °C, 24 hr	0%			–	No reaction.
25	0.2 eq **Pd_2dba_3**, +1 eq *n*-Bu_4Cl, 0.6 eq P(*o*-tolyl)$_3$, THF, **55 °C**, 2 hr	5%	0%		**43**	Slow formation of aminoalcohol A.
26	1 eq **AgOTf**, DCM, 20 min −40 °C, 23 °C, 50 min	>99%	0%		7.50 min	Formation of undesired side-products.
27	1 eq **$Ti(OiPr)_4$**, DCM, −40→23 °C, 24 hr	0%			–	No reaction.
28	+ 1 eq **$TiCl_4$**, DCM, 23 °C, 2 hr	66%	0%		8.78 min	Slow formation of another side-product.
29	0.5 eq **(±)-CSA**, then + 1 eq **(−)-CSA**, DCM, −40→23 °C	100%	0%		–	No reaction with (±)-CSA (solubility?), then complete decomposition with (−)-CSA
30	0.2 eq **$Rh(NBD)_2BF_4^-$**, DCM, 0 °C→23 °C, 48 hr	0%	–			No reaction

Enantioselective synthesis of aziridine 32

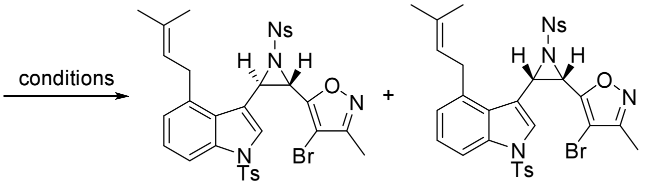

***trans*-32** ***cis*-32**

Table 3. Optimization of enantioselective synthesis of aziridine **32**

#	Conditions	Yield	*trans/cis*	er
1	1.2 eq **31b**, 1.2 eq Cs_2CO_3, DCM (0.23 M), 0 °C (1 hr) → 23 °C (29 hr)	32%	1:5	14:86
2	(*in situ*) 2 eq **39**, 2 eq **38**, 2 eq K_2CO_3, MeCN (1.8 M), 0 °C → 23 °C, 20 hr	29%	3:7	15:85
3	1.2 eq **31b**, 2 eq K_2CO_3, MeCN (0.23 M), 0 °C, 7 hr	62%	5.4:1	7:93
4	1.2 eq **31b**, 3 eq K_2CO_3, MeCN (0.11 M), −20 °C, 16 hr, ~85% conversion	63%	10:1	*Trans* 2:98; *cis* 4:96
5	1.2 eq **31b**, 3 eq K_2CO_3, MeCN (0.05 M), −30 °C, 22 hr (cryostat)	64%	9:1	4:96
6	Scaleup of entry 4 to 410 mg of **14**	56%	9:1	*trans*: 2:98<br>*cis*: 11:89
7	200 mg imine, 1.2 eq **31c**, 3 eq K_2CO_3, MeCN (0.23 M), 23 °C (insol. at 0 °C), 90 min	61%	5:1	24:76
8	1.2 eq **31c**, 1.2 eq P2 base, DCM (0.11 M), −78 °C, 40 min	N/A<br>(NMR too crowded)		38:62
9	1.2 eq **31c**, 1.2 eq Cs_2CO_3, DCM, 0.1 M, −40 → −20 °C, 6 hr	69%	8:1	N/A (issues with purification)
10	1.2 eq **31c**, 1.2 + 2 eq Cs_2CO_3, DCM, 0.1 M, −40 °C (cryostat), 24 hr	71%	7:1	*cis*: 52:48<br>*trans*: 25:75 (desired)<br>Converted: 32:68
11	1.2 eq **31c**, 1.2 eq KHMDS (1 M in THF), DCM, 0.1 M, −78 °C, 10 min	56%	7:1	*cis*: 64:36 (opposite enantiomer)<br>*trans*: 26:74 (desired)<br>Converted: 37:62 (desired)
12	1.2 eq **31c**, 1.2 eq KHMDS (1 M in THF), DCM, 0.1 M, −78 °C, 10 min	59%	1.5:1	*cis*: 74:26 (opposite enantiomer),<br>*trans*: 5:95 (desired)

Note: Reactions performed on 125 (100) mg of the imine **14** (80% purity by qNMR), unless indicated otherwise.

Optimization of the (3+2)-cycloaddition of *cis*-aziridine 32

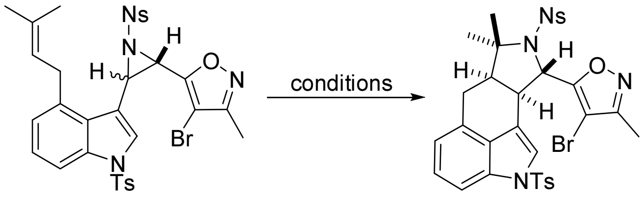

32 **33**

t_R = 9.2 min t_R = 8.1 min

The starting aziridine **32** (purified by LCMS, 20 mg, 0.028 mmol) was placed into a flame-dried tube (8×90 mm), followed by a crystal of naphthaline (scintillation grade, internal standard). The tube was back filled with nitrogen. Anhydrous DCM (1 mL) was added and the tube was cooled to −78 °C. The promoter was then added (TfOH [0.1 M in DCM] or $In(OTf)_3$ [neat], see Table 4) and the reaction progress monitored by LCMS (Agilent InfinityLab Poroshell 120 EC-C18, 2.7 um, 3.0×50 mm, 50→90% MeCN-water, 0.5 mL/min).
For comparison of the reaction traces under TfOH and $In(OTf)_3$ catalysis, see p. SI-8.

Table 4. Optimization of the (3+2)-cycloaddition of *cis*-aziridine **32**

#	Conditions	LCMS conv.	(3+2) Yield		Notes
			LCMS	qNMR	
1	*Cis*-**32**, 2 eq $In(OTf)_3$, DCM, −78 → 0 °C, 5 hr	>98%	46%	33%	d.r. 2.5:1<br>Reaction starts above −20 °C. *Trans*-aziridine consumed almost immediately, giving transient intermediate at 7.48 min (LCMS). *Cis*-aziridine takes several hours to complete.
2	*Trans*-**32**, 2 eq $In(OTf)_3$, DCM, −20 → 0 °C, 4 hr	>98%	–	65% (34% isolated after prep-HPLC)	Aziridine **32** (*trans/cis* 3.5:1, ~65% purity) used crude. Product d.r. 2:1. Purified by prep-HPLC.
3	*Trans*-**32**, 2 eq $In(OTf)_3$, DCM, −20 → −10 °C (Cryostat), 4.5 hr (scaleup of entry 2)	96%	–	62% (308 mg) isolated	Aziridine **32** (*trans/cis* 3.5:1, ~65% purity) used crude. Scale 0.69 mmol. Product d.r. 3:1. Purified by Biotage I. S..
4	*Cis*-**32**, 1 eq TfOH, DCM, −78 → 0 °C, 5 hr	>98%	36%	44%	d.r. 2.6:1 (LCMS).
5	*Trans*-**32** (d.r. 3:1), 0.2 eq TfOH, DCM, −40 → −10 °C, 480 mg scale	>95%	–	30% isolated	Reactions of *cis*-aziridine *cis*-**32** are very sluggish compared to *trans*-aziridine. Purified by Biotage I. S.. Product d.r. 3:1 (NMR).
6	*Trans*-**32**, 2 eq $In(OTf)_3$, DCM, −20 → −10 °C, 4 hr	>98%	–	46% isolated	d.r. 3.2:1 (NMR). Purified by Biotage I. S.
7	(+)-*Trans*-**32** (d.r. 9:1), 1 eq TfOH, DCM, −55 → 10 °C, 21 hr	>99%	–	73% Isolated	Reaction is initially very sluggish, until >0.5 eq of TfOH added – probably an impurity of imine or chiral auxiliary competes for acid.

Note. *Trans*-**32** refers to aziridine mixtures enriched in *trans*-isomer (typically 3:1 for the racemic route and 9:1 for the enantioselective route).

Comparison of TfOH and $In(OTf)_3$ in promoting the (3+2)-cycloaddition

The relative area under the curve (AUC) for the target material is essentially identical under the two conditions:

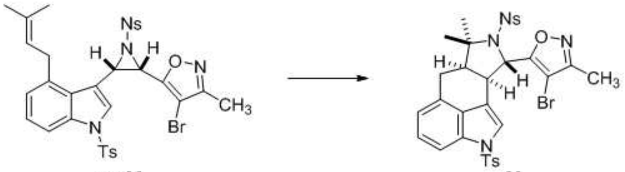

cis-**32**
(purified by prep-HPLC)

33

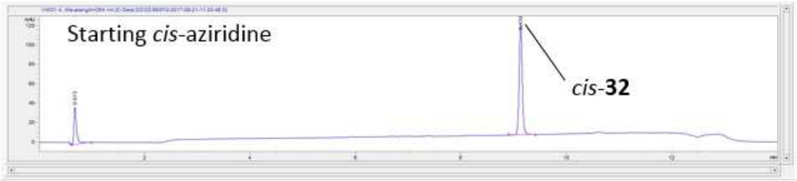

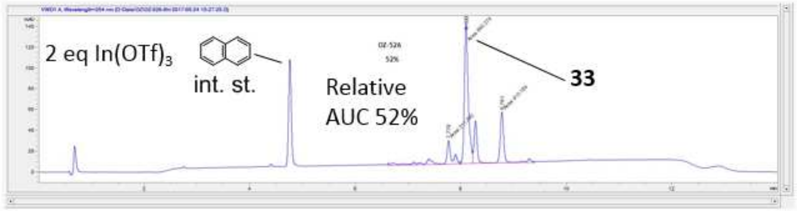

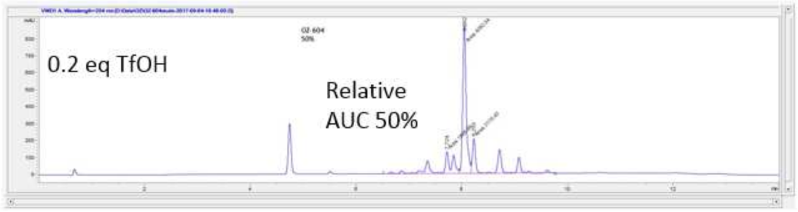

Optimization of denosylation of compound 33

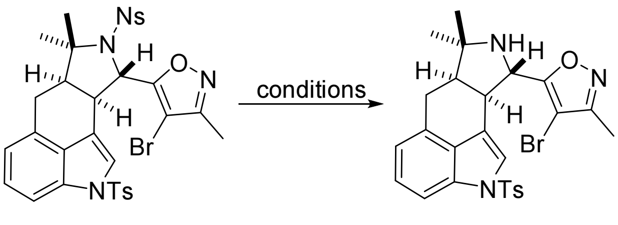

33 **34**

Table 5. Optimization of denosylation of compound **33**

#	Conditions	Yield & Notes
1	0.014 M, 2 eq Cs_2CO_3, 36 eq PhSH, DME, 23 °C	No reaction.
2	0.023 M, 15 eq PhSNa, 60 °C, DMF, 3 hr	70%, partial decomposition.
3	0.055 M, 15 eq PhSNa, DMF, 23 °C, 40 min	50%
4	0.055 M, 3 eq PhSNa, DMF, 23 °C, 2.5 hr	50%
5	0.052 M, 1.3 eq PhSNa, DMF, 23 °C, 5+12 hr	Halts after 50% conversion.
6	0.052 M, 2 eq PhSH, 3 eq K_2CO_3, 3 eq 18-crown-6, MeCN (degassed), 23 °C, 5 hr	67% isolated
7	0.077 M, 3 eq PhSNa, MeCN, 23 °C, 5 hr	Halts after 50% conversion.
8	0.077 M, 6 eq PhSNa, MeCN 23 °C, 17 hr	52%

Optimization of carbonylation/N-O cleavage

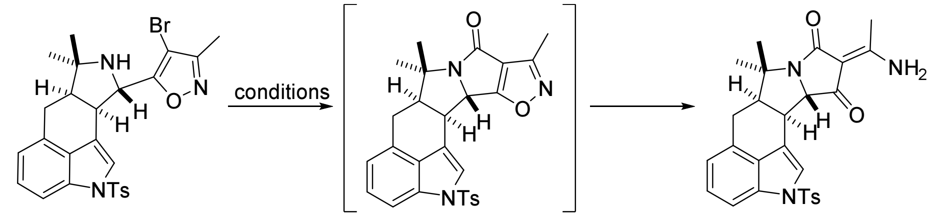

34 **27** **28**

Table 6. Optimization of carbonylation/N-O cleavage

#	Conditions	Yield & Notes
1	40 mol% $Pd(OAc)_2$, 3 eq DABCO, 1.2 eq $BuPAd_2$, CO (1 atm), DMSO (0.046 M), 120 °C, 5 hr	67% isolated.
2	20 mol% $Pd(OAc)_2$, 3 eq DABCO, 1.2 eq $BuPAd_2$, CO (1 atm), DMSO (0.046 M), 120 °C, 22 hr	40% isolated (incomplete even after 22 hr).
3	40 mol% $Pd(OAc)_2$, 3 eq DABCO, 1.2 eq PPh_3, CO (1 atm), DMSO (0.046 M), 120 °C, 16 hr	~15% conversion, partial decomposition.
4	40 mol% $Pd(OAc)_2$, 3 eq DABCO, 1.2 eq $BuPAd_2$, CO (1 atm), DMSO (0.046 M), 23 °C, 1 hr	No reaction.
5	40 mol% $Pd(OAc)_2$, 3 eq DABCO, 1.2 eq PPh_3, CO (1 atm), DMSO (0.046 M), 23 °C, 1 hr	No reaction.
6	5 mol% $Pd(OAc)_2$, 3 eq DABCO, 1.2 eq $BuPAd_2$, CO (1 atm), DMSO (0.07 M), 120 °C, 19 hr	80% isolated.
7	5 mol% $Pd(OAc)_2$, 1.0 eq DABCO, 1.2 eq $BuPAd_2$, CO (1 atm), DMSO (0.06 M), 120 °C, 19 hr	~50% conversion
8	5 mol% $Pd(OAc)_2$, 3.0 eq DABCO, 0.1 eq $BuPAd_2$, CO (1 atm), DMSO (0.06 M), 120 °C, 19 hr	~50% conversion

Identification of the reducing agent in tandem carbonylation/N–O cleavage

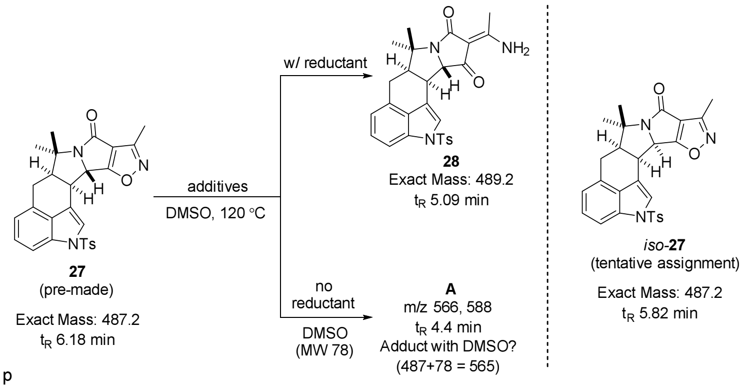

p

Table 7. Identification of the reducing agent in tandem carbonylation/N–O cleavage

#	Conditions	Deviation	Result
1	Background: DMSO, 120 °C, 1 hr	**Background test: No additives**	50% conversion, major new product **A** (4.49 min, m/z 566, 588), 4% iso-**27**,
2	0.05 eq $Pd(OAc)_2$, 3 eq DABCO, 1.2 eq *n*-$BuPAd_2$, N_2 atmosphere, DMSO, 120 °C, 2 hr	**No CO**	100% conversion, moderately clean reduction to **28**, 13% *iso*-**27**
3	0.05 eq $Pd(OAc)_2$, 1.2 eq *n*-$BuPAd_2$, N_2 atmosphere, DMSO, 120 °C, 3 hr	**No DABCO, no CO**	100% conversion, major new product **A** (4.49 min, m/z 566, 588), 1% iso-**27** – overall looks like entry 1 gone to completion.
4	0.05 eq $Pd(OAc)_2$, 3 eq DABCO, N_2 atmosphere, DMSO, 120 °C, 2 hr	**No *n*-$BuPAd_2$, no CO**	100% conversion, no-so-clean reduction to **28**, several minor side-products
5	0.05 eq $Pd(OAc)_2$, 1.2 eq *n*-$BuPAd_2$, CO atmosphere, DMSO, 120 °C, 2 hr	**No DABCO, with CO**	100% conversion, clean reduction to **28**

Conclusions:

1. Both CO and DABCO can serve as stoichiometric reductants of the N–O bond in **27** (entry 2, 4, 5)
2. Reduction in the presence of CO is much cleaner than with DABCO (entry 5 vs. 4)
3. No reduction occurs in the absence of CO and DABCO (entry 1)
4. *n*-$BuPAd_2$ does not act as the N–O reductant under these conditions (entry 3)

Synthetic Procedures

(−)-α-Cyclopiazonic acid, 1, and (+)-iso-α-Cyclopiazonic acid, 2

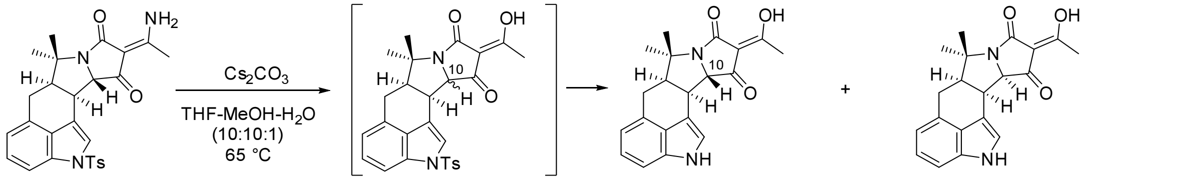

28 **(−)-α-Cyclopiazonic Acid (1)** **(+)-iso-α-Cyclopiazonic Acid (2)**

The *N*-Ts group was removed under the previously published conditions.[17]

HPLC-grade THF, MeOH, and water were degassed (by sonication under vacuum for 5 min) individually. *N*-Ts α-CPA imine **28** (20 mg, 0.041 mmol) was placed into a 1.75 mL vial with a stirbar, followed by Cs_2CO_3 (53 mg, 0.16 mmol, 4 eq). Degassed solvents (0.50 mL of THF, 0.50 mL of MeOH, and 0.05 mL of water) were added, the overhead space was flushed with argon, the vial tightly capped and heated in a 65 °C oil bath, with daily monitoring of the reaction progress by LCMS (50-90 MeCN-H_2O). After 4 days (approx. 90 hr), the conversion was deemed complete.

The reaction mixture was cooled to 23 °C and diluted with water (10 mL). 2 M aq. HCl was added until pH 0, and the aq. layer was extracted with DCM (5×2 mL). The combined organic layer was washed with brine (3 mL), dried over Na_2SO_4 and concentrated to give brown oil (approx. 20 mg). Purification by chromatography on silica (0.6×6 cm, 1→3→6→10% MeOH-DCM, 10-10-10-20 mL) provided the inseparable mixture of α-CPA and iso-α-CPA (d.r. 2.5:1) as brown solid (9.5 mg, 70%, d.r. 2.5:1).

Analytically pure samples of α-CPA and iso-α-CPA were then obtained by semi-preparative HPLC (XTerra Prep MS C18 OBD 10μm, 19×50 mm, 10 mL/min, 20→95% MeCN-H_2O+0.3% TFA, over 12 min) as off-white powders. The synthetic (−)-α-CPA was identical to a commercial sample (Alfa Aesar) by TLC, LCMS, IR and NMR analysis, as well as matched the reported[18] optical rotation.

Notes. 1. The C-10 stereocenter undergoes rapid equilibration under the reaction conditions. The process is much faster than the *N*-Ts cleavage or NH_2 hydrolysis. 2. It is essential to acidify the aqueous layer during workup, otherwise α-CPA and iso-α-CPA would not be extracted. 3. Prep-HPLC separation should be performed with 0.1-0.3% TFA in the eluent, to ensure adequate peak shape. 4. α-CPA and iso-α-CPA are rather acidic and their solutions in acetone-d_6 do not match the reported spectra unless acidified with 0.1% TFA.

Yield: 9.5 mg (70%, d.r. 2.5:1)

(−)-α-Cyclopiazonic acid, 1

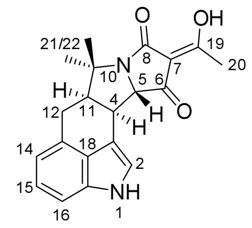

(−)-α-Cyclopiazonic Acid (1)

Off-white solid.

TLC (10:90 MeOH-DCM): R_f = 0.18.

Mp (racemic, MeCN-H_2O): 221-222 °C (dec.).
Mp (commercial (−)-α-CPA, Alfa Aesar): dec. above 224 °C.

^{1}H NMR (500 MHz, Acetone-*d*$_6$+0.1%TFA): δ = 10.04 (br.s, 1H, NH), 7.18 (d, *J* = 8.1 Hz, 1H, H-16), 7.13 (d, *J* = 1.8 Hz, 1H, H-2), 7.03 (dd, *J* = 8.1, 7.0 Hz, 1H, H-15), 6.81 (d, *J* = 7.0 Hz, 1H, H-14), 4.11 (d, *J* = 11.2 Hz, 1H, H-5), 3.68 (dd, *J* = 11.2, 5.9 Hz, 1H, H-4), 3.15 – 2.95 (m, 2H, H-12), 2.60 (dt, *J* = 11.7, 5.9 Hz, 1H, H-11), 2.38 (s, 3H, H-20), 1.67 (s, 3H, H-21/22), 1.60 (s, 3H, H-22/21).

^{1}H NMR (400 MHz, Chloroform-*d*) δ = 8.11 (br.s, 1H, NH), 7.22 (d, *J*=8.2, 1H, H-16), 7.20 – 7.10 (m, 2H, H-2,15), 6.92 (dd, *J*=6.9, 0.7, 1H, H-14), 4.10 (d, *J*=11.0, 1H, H-5), 3.67 (dd, *J*=11.0, 5.8, 1H, H-4), 3.11 – 3.02 (m, 2H, H-12), 2.65 (ddd, *J*=9.8, 7.7, 5.8, 1H, H-11), 2.46 (s, 3H, H-20), 1.68 (s, 3H, H-21/22), 1.64 (s, 3H, H-22/21).

^{13}C NMR (125 MHz, Acetone-*d*$_6$+0.1%TFA): δ = 195.1 (C-6), 184.2 (C-19), 175.8 (C-8), 134.7 (C-17), 129.7 (C-13), 127.0 (C-18), 123.2 (C-15), 122.1 (C-2), 116.6 (C-14), 110.3 (C-3), 109.5 (C-16), 106.3 (C-7), 72.7 (C-5), 63.9 (C-10), 54.0 (C-11), 36.9 (C-4), 27.1 (C-12), 26.4 (C-22/21), 24.6 (C-21/22), 19.2 (C-20).

^{13}C NMR (125 MHz, Chloroform-*d*) δ = 195.5 (C-6), 185.6 (C-19), 175.4 (C-8), 133.5 (C-17), 128.7 (C-13), 126.0 (C-18), 123.2 (C-15), 121.0 (C-2), 116.7 (C-14), 110.0 (C-3), 108.8 (C-16), 105.6 (C-7), 72.0 (C-5), 63.7 (C-10), 53.2 (C-11), 36.3 (C-4), 26.7 (C-12), 26.5 (C-21/22), 24.6 (C-22/21), 20.1 (C-20).

HRMS (TOF ESI$^+$), m/z: calcd for $C_{20}H_{21}N_2O_3^+$ [M+H]$^+$ 337.1547, found 337.1551.

IR (neat) ν_{max}: 3360 (br. s), 1704 (m), 1606 (s), 1446 (m), 1385 (m), 1368 (m), 751 (m).

$[\alpha]_D^{21}$ = −98° (*c* = 0.37, $CHCl_3$); lit.[18] $[\alpha]_D^{20}$ = −92° (*c* = 0.100, $CHCl_3$).

LCMS (50→90% MeCN-H_2O + 0.1% HCOOH): t_R 4.80 min (natural sample [Alfa Aesar J61594] t_R 4.77 min).

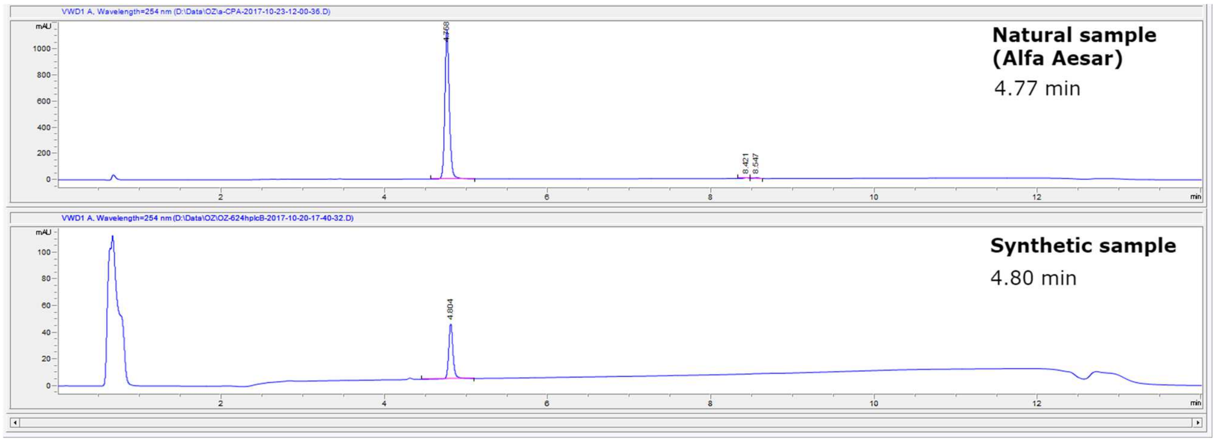

Comparison of NMR Data in Chloroform

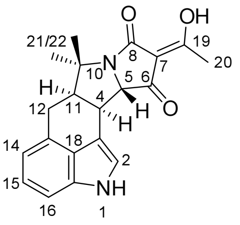

(−)-α-Cyclopiazonic Acid (1)
$CDCl_3$

#	δ_C (nat)*	δ_C (obs)	$\Delta(\delta_C)$	δ_H (nat)*	δ_H (obs)	$\Delta(\delta_H)$
1				8.08 (1H, br.s)	8.11 (1H, br.s)	**0.03**
2	121.0	121.0	**0.0**	7.16 (2H, m)	7.16 (2H, m)	**0.00**
3	110.2	110.0	−**0.2**			
4	36.2	36.3	**0.1**	3.67 (1H, dd, J = 11.0, 5.5)	3.67 (1H, dd, 11.0, 5.7)	**0.00**
5	72.0	72.0	**0.0**	4.07 (1H, d, J = 11.0)	4.10 (1H, d, J = 11.0)	**0.03**
6	195.3	195.5	**0.2**			
7	105.8	105.6	−**0.2**			
8	175.4	175.4	**0.0**			
10	63.6	63.7	**0.1**			
11	53.2	53.2	**0.0**	2.64 (1H, ddd, J = 10.5, 7.2, 5.5)	2.65 (1H, ddd, J = 9.9, 7.6, 5.8)	**0.01**
12	26.7	26.7	**0.0**	3.06 (2H, m)	3.06 (2H, m)	**0.00**
13	128.8	128.7	**−0.1**			
14	116.7	116.7	**0.0**	6.92 (1H, dd, J = 6.9, 0.9)	6.92 (1H, dd, J = 6.9, 0.7)	**0.00**
15	123.2	123.2	**0.0**	7.16 (2H, m)	7.16 (2H, m)	**0.00**
16	108.8	108.8	**0.0**	7.22 (1H, d, J = 8.2)	7.22 (1H, d, J = 8.2)	**0.00**
17	133.5	133.5	**0.0**			
18	126.1	126.0	−**0.1**			
19	185.0	185.6	**0.6**			
20	19.9	20.1	**0.2**	2.45 (3H, s)	2.46 (3H, s)	**0.00**
21	24.6	24.6	**0.0**	1.68 (3H, s)	1.68 (3H, s)	**0.00**
22	26.5	26.5	**0.0**	1.64 (3H, s)	1.64 (3H, s)	**0.00**

Notes: * Commercial α-CPA from Alfa Aesar (J61594). Signals assigned based on 2D NMR data (COSY, HSQC, HMBC).

Comparison of NMR Data in Acetone

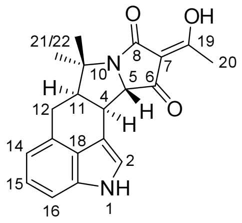

(–)-α-Cyclopiazonic Acid (1)

acetone-d_6 + 0.1% TFA

#	Lit. δ_C[18]	Obs. δ_C	$\Delta(\delta_C)$	Lit. δ_H[18]	Obs. δ_H	$\Delta(\delta_H)$
1				N/A	10.04 (1H, br.s)	
2	121.9*	122.1	**0.2**	7.14 (1H, s)	7.13 (1H, d, J = 1.8 Hz)	**−0.01**
3	110.2	110.3	**0.1**			
4	36.9	36.9	**0.0**	3.68 (1H, dd, J = 11.0, 5.9)	3.68 (1H, dd, J = 11.2, 5.9)	**0.00**
5	72.6	72.7	**0.1**	4.11 (1H, d, J = 11.0)	4.11 (1H, d, J = 11.2 Hz)	**0.00**
6	195.3	195.1	**−0.2**			
7	106.4	106.3	**−0.1**			
8	175.4	175.8	**0.4**			
10	63.8	63.9	**0.1**			
11	53.9	54.0	**0.1**	2.60 (1H, m)	2.60 (1H, dt, J = 11.7, 5.9 Hz)	**0.00**
12	27.0	27.1	**0.1**	3.02 (1H, dd, J = 15.9, 5.9)<br>3.06 (1H, t, J = 14.9	3.15-2.95 (2H, m)	**N/A**
13	129.7	129.7	**0.0**			
14	116.6	116.6	**0.0**	6.81 (1H, d, J = 6.9)*	6.81 (1H, d, J = 7.0 Hz)	**−0.01**
15	123.1*	123.2	**0.1**	7.04 (1H, dd, J = 8.2, 6.9)	7.03 (1H, dd, J = 8.1, 7.0)	**−0.01**
16	109.5	109.5	**0.0**	7.19 (1H, d, J = 8.3)*	7.18 (1H, d, J = 8.1 Hz)	**0.00**
17	134.5	134.7	**0.2**			
18	127.0	127.0	**0.0**			
19	183.5	184.2	**0.7**			
20	19.0	19.2	**0.2**	2.38 (3H, s)	2.38 (3H, s)	**0.00**
21	24.6	24.6	**0.0**	1.60 (3H, s)	1.60 (3H, s)	**0.00**
22	26.4	26.4	**0.0**	1.68 (3H, s)	1.67 (3H, s)	**−0.01**

Notes: * These signals have been reassigned.

(+)-iso-α-Cyclopiazonic acid, 2

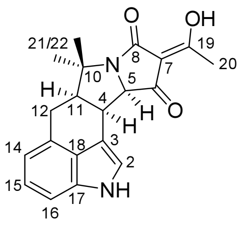

(+)-iso-α-Cyclopiazonic Acid (2)

Off-white solid.

TLC (10:90 MeOH-DCM): R_f = 0.18.

^{1}H NMR (500 MHz, Acetone-d_6+0.1%TFA): δ = 9.86 (br.s, 1H, NH), 7.11 (d, *J* = 8.2 Hz, 1H, H-14), 7.01 (dd, *J* = 8.2, 7.0 Hz, 1H, H-15), 6.88 (s, 1H, H-2), 6.78 (d, *J* = 7.0 Hz, 1H, H-16), 4.65 (d, *J* = 5.5 Hz, 1H, H-5), 3.78 (t, *J* = 5.5 Hz, 1H, H-4), 3.26 (dd, *J* = 17.5, 6.0 Hz, 1H, H-12a), 3.09 – 2.99 (m, 2H, H-12b,11), 2.39 (s, 3H, H-20), 1.48 (s, 3H, H-21/22), 0.85 (s, 3H, H-22/21).

^{13}C NMR (125 MHz, Acetone-d_6+0.1%TFA): δ = 183.5* (C-19), 134.7 (C-17), 129.8 (C-13), 128.4 (C-18), 123.5 (C-15), 120.9 (C-2), 116.3 (C-16), 109.10 (C-14), 108.8 (C-3), 107.0* (C-7), 72.3* (C-5), 63.0 (C-10), 53.5 (C-11), 36.4 (C-4), 30.1* (C-22/21), 26.8 (C-12), 21.7 (C-21/22), 19.2 (C-20). (Signals marked with * could not be observed in ^{13}C directly due to broadening and were found in HSQC/HMBC spectra, as appropriate. Signals for C-6 and C-8 were not observed).

HRMS (TOF ESI$^+$), m/z: calcd for $C_{20}H_{21}N_2O_3^+$ [M+H]$^+$ 337.1547, found 337.1555.

IR (neat) ν_{max}: 3403 (br. s), 1681 (s), 1597 (s), 1506 (m), 1443 (m), 1209 (m), 751 (w).

$[\alpha]_D^{21}$ = +108° (*c* = 0.33, $CHCl_3$); lit.[18] $[\alpha]_D^{20}$ = +323.5° (*c* = 0.150, $CHCl_3$).

LCMS (50→90% MeCN-H_2O + 0.1% HCOOH): t_R 4.29 min.

Comparison of NMR Data

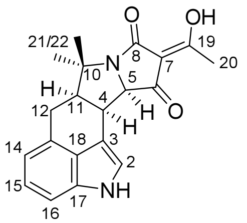

(+)-iso-α-Cyclopiazonic Acid (2)

acetone-d_6 + 0.1% TFA

#	Lit. δ_C[18]	Obs. δ_C	$\Delta(\delta_C)$	Lit. δ_H[18]	Obs. δ_H	$\Delta(\delta_H)$
1				N/A	9.86 (1H, br.s)	
2	120.8[b]	120.9	**0.1**	6.89 (1H, s)	6.88 (1H, s)	**−0.01**
3	108.7[b]	108.8	**0.1**			
4	36.5	36.4	**−0.1**	3.78 (1H, t, J = 5.0)	3.78 (1H, t, J = 5.5)	**0.00**
5	72.3	72.3[a]	**0.0**	4.64 (1H, br. s)	4.65 (1H, d, J = 5.5)	**0.01**
6	195.0	N/A[a]	**N/A**			
7	107.1	107.0[a]	**−0.1**			
8	173.2	N/A[a]	**N/A**			
10	62.9	63.0	**0.1**			
11	53.5	53.5	**0.0**	½ × 3.06 (2H, m)[b]	½ × 3.04 (2H, m)	**−0.02**
12	26.8	26.8	**0.0**	3.27 (1H, m)[b]	3.26 (1H, dd, J = 17.5, 6.0)	**−0.01**
				½ × 3.06 (2H, m)[b]	½ × 3.04 (2H, m)	**−0.02**
13	129.9	129.8	**−0.1**			
14	116.3	116.3	**0.0**	6.78 (1H, d, J = 7.1)[b]	6.78 (1H, d, J = 7.0)	**0.00**
15	123.5[b]	123.5	**0.0**	7.02 (1H, dd, J = 8.0, 7.1)	7.01 (1H, dd, J = 8.2, 7.0)	**−0.01**
16	109.1[b]	109.1	**0.0**	7.11 (1H, d, J = 8.0)[b]	7.11 (1H, d, J = 8.2)	**0.00**
17	134.6	134.7	**0.1**			
18	128.4	128.4	**0.0**			
19	183.1	183.5[a]	**0.4**			
20	19.1	19.2	**0.1**	2.40 (3H, s)	2.39 (3H, s)	**−0.01**
21	21.7	21.7	**0.0**	0.85 (3H, s)	0.85 (3H, s)	**0.00**
22	30.5	30.1[a]	**−0.4**	1.48 (3H, s)	1.48 (3H, s)	**0.00**

Notes: A. These signals could not be observed by ^{13}C NMR directly due to broadening (C-5,6,7,8,19) or being hidden under the solvent peak (C-22) and, where appropriate, were inferred from HSQC/HMBC. B. These signals were misassigned in the isolation report.[18] We reassigned them based on the HSQC and HMBC data of the synthetic material.

***N*-((4-(3-Methylbut-2-en-1-yl)-1-tosyl-1*H*-indol-3-yl)methylene)-4-nitrobenzenesulfonamide, 14**

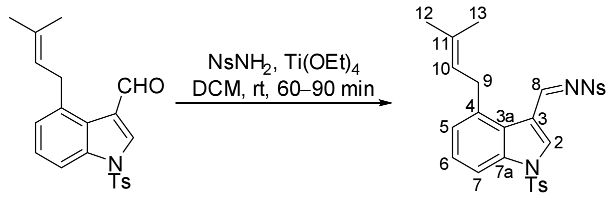

18 **14**

The starting aldehyde **18** (500 mg, 1.36 mmol) and 4-nitrobenzenesulfonamide (330 mg, 1.63 mmol, 1.2 eq) were placed into a flame-dried Schlenk tube and backfilled with nitrogen. Anhydrous DCM (6.8 mL, 0.2 M) was then added. Neat $Ti(OEt)_4$ (90%, 0.63 mL, 2.7 mmol, 2.0 eq) was added, and the reaction mixture was stirred at 23 °C until LCMS indicated complete consumption of the starting material (60−90 min).
The reaction mixture was transferred into a 100 mL Erlenmeyer flask and diluted with 20 mL DCM. Water (30 mL) was added and the mixture was shaken for 5 sec, then gently swirled, to ensure the hydrolysis of [Ti] species. The resulting slurry was quickly filtered through a loose plug of glass wool (w×h: 2.5×5 cm), washing the solids with 20 mL of DCM and 20 mL of water. The resulting liquid mixture, mostly free from solids and slime, was transferred into a separating funnel. Note: if the filtration is not performed, the subsequent extraction becomes very inefficient and time consuming; also, if too tight a filter is used, it gets blocked almost instantly.
The layers were separated, and the aqueous layer was extracted with DCM (3×10 mL). The combined organic layer was washed with brine (30 mL), dried over Na_2SO_4 and concentrated to give yellow solid, which contained the desired product along with 10−20% of $NsNH_2$. The crude was transferred into a 50 mL round-bottom flask as a solution in 5 mL of DCM and stirred at 500 rpm. n-Pentane (5 mL) was added dropwise to precipitate impurities (mostly $NsNH_2$), and after 10 min the solution was filtered through a plug of Celite (w×h: 1.5×2 cm), washing with 20 mL of 1:1 DCM-*n*-pentane. The volatiles were evaporated to give the product as yellow foam, which was used as is, without further purification (~90% purity by NMR). Small amounts of analytically pure samples could be prepared by recrystallization from 2:3 MTBE-heptane.
Note: the product is extremely unstable of silica or alumina; all attempts to perform chromatography on it resulted in partial or complete decomposition.

Yield: 600 mg (80%).
Yellow foam
White solid (r/c MTBE-heptane)

TLC (75% EtOAc-petrol): R_f = 0.55.

Mp: 162−164 °C (MTBE-hexane).

^{1}H NMR ($CDCl_3$, 500 MHz): δ = 9.37 (s, 1H, H-8), 8.55 (s, 1H, H-2), 8.39 (d, *J* = 8.9 Hz, 2H, 2 × M-H-Ph_{Ns}), 8.18 (d, *J* = 8.9 Hz, 2H, 2 × *o*-H-Ph_{Ns}), 7.90−7.77 (m, 3H, H-7, 2 × *o*-H-Ph_{Ts}), 7.37−7.29 (m, 3H, H-6, 2 × M-H-Ph_{Ts}), 7.18 (d, *J* = 7.4 Hz, 1H, H-5), 5.12 (m, 1H, H-10), 3.72 (d, *J* = 5.9 Hz, 2H, H-9), 2.39 (s, 3H, Ts-CH_3), 1.77 (s, 3H, H-13), 1.73 (s, 3H, H-12).

^{13}C NMR ($CDCl_3$, 125 MHz): δ = 166.8 (C-8), 150.6 (Ph_{Ns}-C_p), 146.7 (Ph_{Ts}-C_p), 144.9 (Ph_{Ns}-C_i), 136.0, 135.9, 135.4 (C-7a, C-4, C-11), 134.1, 134.1 (C-2, Ph_{Ts}-C_i), 130.6 (Ph_{Ts}-C_M), 129.3 (Ph_{Ns}-C_o), 127.7 (Ph_{Ts}-C_o), 126.2, 126.1, 126.0 (C-5, C-6, C-3a), 124.5 (Ph_{Ns}-C_M), 121.4 (C-10), 117.3 (C-3), 112.0 (C-7), 33.9 (C-9), 25.8 (C-13), 21.9 (Ts-$\underline{C}H_3$), 18.4 (C-12).

HRMS (TOF ESI^+), m/z: calcd for $C_{27}H_{25}N_3O_6S_2Na$ $[M+Na]^+$ 574.1085, found 574.1065.

IR (neat) ν_{max}: 3107 (br. w), 2914 (br. w), 1593 (m), 1570 (m), 1529 (s), 1376 (m), 1348 (m), 1159 (s), 1151 (s), 1086 (s), 855 (m), 784 (m), 738 (m).

1-((4-(Ethoxycarbonyl)-3-methylisoxazol-5-yl)methyl)tetrahydro-1*H*-thiophen-1-ium trifluoromethanesulfonate, 15a[19]

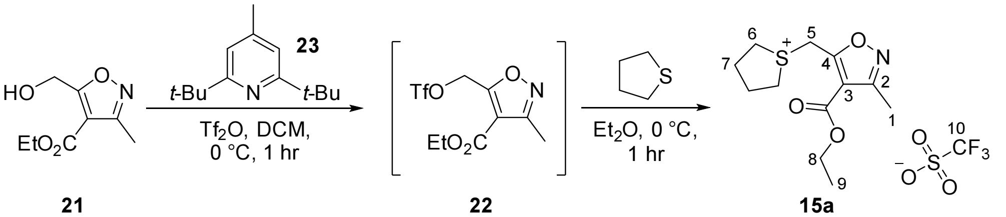

The starting alcohol **21** (1.00 g, 5.4 mmol) and 2,6-di(*t*-butyl)-4-methylpyridine **23** (1.33 g, 6.49 mmol, 1.2 eq) were dissolved in anhydrous DCM (50 mL) in a flame-dried two-neck 250 mL flask and cooled to 0 °C. Then, Tf_2O (1.09 mL, 6.49 mmol, 1.2 eq) was added and the clear colorless reaction mixture was stirred at 0 °C. In 5 min, white precipitate was observed. After 1 hr at 0 °C, the reaction was quickly passed through a 1.5×5 cm pad of silica into a flame-dried flask, washing the silica with 50 mL of anhydrous DCM. The filtrate was concentrated to give yellowish waxy solid of the intermediate triflate **22**, which was used immediately in the next step.
Triflate **22** was suspended in anhydrous Et_2O and cooled to 0 °C. Tetrahydrothiophene (0.57 mL, 10.9 mmol, 2 eq) was added dropwise to the rapidly stirred solution and the resulting suspension was stirred at 0 °C for 1 hr. The product was then isolated by filtration, washed with 50 mL of Et_2O and dried under high-vacuum.

Yield: 0.92 g (42%).
White solid.

TLC (10% MeOH-DCM): R_f = 0.32, streaks.

Mp 92-93 °C (Et_2O).

^{1}H NMR (500 MHz, $CDCl_3$): δ = 5.02 (s, 2H, H-5), 4.38 (q, *J* = 7.1 Hz, 2H, H-8), 3.92–3.80 (m, 2H, H-6a), 3.71–3.58 (m, 2H, H-6b), 2.47 (s, 3H, H-1), 2.45–2.30 (m, 4H, H-7), 1.38 (t, *J* = 7.2 Hz, 3H, H-9).

^{13}C NMR (125 MHz, $CDCl_3$): δ = 165.2 (C_q), 161.3 (C_q), 160.5 (C_q), 120.9 (q, J = 321 Hz, C-10), 113.8 (C_q), 62.3 (C-8), 44.8 (C-6), 35.8 (C-5), 29.0 (C-7), 14.2 (C-9), 11.9 (C-1).

IR (neat) ν_{max}: 1719 (s), 1618 (m), 1272 (s), 1261 (s), 1248 (s), 1152 (s), 1033 (s).

(−)-(1*S*,1'*R*,2'*S*,4*R*,4'*S*)-3'-((4-(Ethoxycarbonyl)-3-methylisoxazol-5-yl)methyl)-7,7-dimethyl-2-oxo-3'-thia[1,2'-bi(bicyclo[2.2.1]heptan)]-3'-ium trifluoromethanesulfonate, (−)-15b

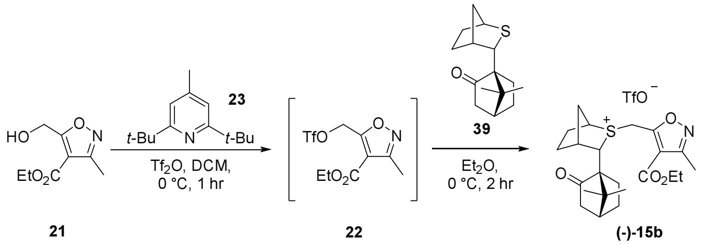

The starting alcohol **21** (100 mg, 0.54 mmol) and 2,6-di-*tert*-butyl-4-methylpyridine **23** (133 mg, 0.65 mmol, 1.2 eq) were placed into a flame-dried Schlenk tube, followed by anhydrous DCM (5 mL). The clear colorless solution was cooled to 0 °C. Triflic anhydride (110 µL, 0.65 mmol, 1.2 eq) was then added and the reaction was stirred at 0 °C for 1 hr. The resulting mixture was quickly filtered through a 1.5×1.5 cm plug of silica (pre-washed with 10 mL of anhydrous DCM) and washed with 7 mL of anhydrous DCM. The filtrate was concentrated in cold and the resulting triflate **22** was quickly resuspended in anhydrous ether (2 mL) at 0 °C.
(+)-(1*S*,1'*R*,2'*S*,4*R*,4'*S*)-7,7-dimethyl-3'-thia[1,2'-bi(bicyclo[2.2.1]heptan)]-2-one (+)-**39** (149 mg, 0.59 mmol) was then added and the resulting white suspension was stirred at 0 °C for 2 hr. The white precipitate of **15b** (contaminated with excess pyridine and other impurities) was carefully filtered and washed with ether (10 mL).

Purification on Biotage Isolera system (ZIP KP-Sil 10 g, 1→20% MeOH-DCM, 1-10-2 CV) afforded the target material as white crystalline solid.

Yield: 256 mg (83%).
White crystalline solid.

Mp: 128–129 °C (dec.).

TLC (10% MeOH-DCM): R_f = 0.50.

^{1}H NMR (500 MHz, $CDCl_3$): δ = 5.08 (d, *J* = 14.6 Hz, 1H), 4.96 (d, *J* = 14.7 Hz, 1H), 4.93 (br. dd, *J* = 3.3, 1.5 Hz, 1H), 4.43 (br. d, *J* = 4.5 Hz, 1H), 4.38 (q, *J* = 7.1 Hz, 2H), 3.25 (br.s, 1H), 2.95 (br.d, *J* = 13.0 Hz, 1H), 2.60 (dt, *J* = 18.8, 4.0 Hz, 1H), 2.47 (s, 3H), 2.46–2.39 (m, 1H), 2.33 (dt, *J* = 12.9, 1.9 Hz, 1H), 2.27–2.13 (m, 4H), 2.00 (d, *J* = 18.8 Hz, 1H), 1.78–1.59 (m, 3H), 1.49–1.42 (m, 1H), 1.38 (t, *J* = 7.2 Hz, 3H), 1.27 (s, 3H), 1.13 (s, 3H).

^{13}C NMR (125 MHz, $CDCl_3$): δ = 216.1, 165.9, 161.2, 160.3, 120.8 (q, *J* = 320.2 Hz, CF_3), 112.8, 70.5, 62.2, 60.7, 60.0, 50.3, 45.3, 44.2, 43.5, 41.6, 37.6, 33.2, 27.0, 24.4, 22.0, 19.5, 14.3, 11.9.

HRMS (TOF ESI^+), m/z: calcd for $C_{23}H_{32}NO_4S^+$ [R_3S^+] 418.2047, found 418.2039.

IR (neat) ν_{max}: 1740 (m), 1720 (m), 1280 (m), 1259 (s), 1150 (s), 1098 (m), 1031 (s).

$[\alpha]_D^{23}$ = −38° (*c* = 1.0, $CHCl_3$).

4-Allyl-1-tosyl-1*H*-indole-3-carbaldehyde, 17

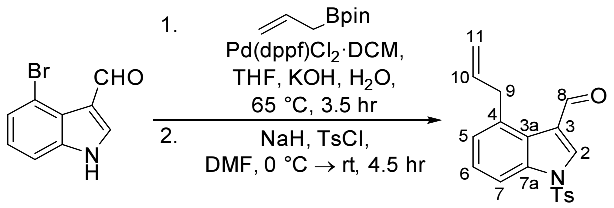

16 **17**

A 500 mL three-neck flask was charged with 4-bromo-3-formylindole (10.00 g, 44.64 mmol), then evacuated and refilled with nitrogen 3 times. Degassed (nitrogen sparging for 30 min) anhydrous THF (110 mL) was then added, followed by solution of KOH in degassed water (2.0 M, 33 mL, 67 mmol, 1.3 eq; prepared by dissolving 6.4 g of KOH in 50 mL degassed water (nitrogen sparging for 30 min) and cooling to 23 °C). Then, $Pd(dppf)Cl_2 \cdot DCM$ (1200 mg, 1.47 mmol, 3.3 mol%) was added in one portion. A reflux condenser was attached, the overhead space flushed with nitrogen (3 times), and the reaction mixture was placed into an oil bath (65 °C) and stirred for 20 min. A solution of allyl-Bpin (10.9 mL, 58 mmol, 1.3 eq) in anhydrous THF (20 mL) was then added with a syringe pump (9.9 mL/hr). After all allyl-Bpin was added, the reaction mixture was stirred for another 20 min, when TLC analysis showed complete consumption of the starting material.

The reaction mixture was cooled to 23 °C, and diluted with 200 mL of ether and 100 mL of water. The phases were separated, and the aqueous layer was extracted with ether (3x30 mL). The combined organic layer was washed with brine (50 mL), dried over Na_2SO_4, and concentrated to give crude indole as deep-red oil.

The crude material was immediately dissolved in anhydrous DMF (120 mL) in a flame-dried 500 mL flask and cooled to 0 °C. Then, NaH (2.50 g, 60% in oil, 62.5 mmol, 1.4 eq) was added portionwise (warning: intense gas evolution!) When bubbling ceased (~10 min), TsCl (11.0 g, 58 mmol, 1.3 eq) was added in one portion. The reaction mixture was stirred at 0 °C for 20 min and at 23 °C for 4 hr.

The reaction mixture was then cooled to 0 °C and the reactants carefully quenched with 100 mL of water. The mixture was then poured into a mixture of 50 mL water, 100 mL of brine and 100 mL of ether. The layers were separated and the aqueous layer was extracted with ether (6x60 mL). The combined organic layer was washed with brine (100 mL), dried (Na_2SO_4) and concentrated to give dark brown oil. Purification on Biotage Isolera system (ZIP KP-Sil, 120 g, 5 → 35% EtOAc-petrol, 1-10-2 CV) provided the target material as brownish solid.

Yield: 13.56 g (91%).
Brownish solid.

TLC (20% EtOAc-*n*-pentane): R_f = 0.35.

Mp: 115−116 °C (EtOAc-petrol).

^{1}H NMR ($CDCl_3$, 400 MHz): δ = 10.07 (s, 1H, H-8), 8.32 (s, 1H, H-Ar), 7.85 (*app* d, *J* = 8.4 Hz, 3H, 3×H-Ar), 7.35−7.28 (m, 3H, 3×H-Ar), 7.18 (d, *J* = 7.5 Hz, 1H, H-Ar), 6.00 (ddt, *J* = 17.1, 10.2, 6.1 Hz, 1H, H-10), 5.02 (dq, *J* = 10.2, 1.5 Hz, 1H, H-11a), 4.91 (dq, *J* = 17.1, 3×1.7 Hz, 1H, H-11b), 3.95 (d, *J* = 6.1 Hz, 2H, H-9), 2.37 (s, 3H, H-TsC$\underline{H}_3$).

^{13}C NMR ($CDCl_3$, 100 MHz): δ = 185.0, 146.3, 137.2, 137.2, 136.2, 135.2, 134.4, 130.4, 127.5, 126.2, 126.2, 125.4, 123.8, 116.0, 111.6, 39.4, 21.8.

HRMS (ESI$^+$), m/z: calcd for $C_{19}H_{18}NO_3S$ $[M+H]^+$ 340.1002, found 340.0999; calcd for $C_{19}H_{17}NNaO_3S$ $[M+Na]^+$ 362.0821, found 362.0816.

IR (solid) ν_{max}: 1692, 1640, 1534, 1374, 1272, 1149, 1089, 1041, 878.

4-(3-Methylbut-2-en-1-yl)-1-tosyl-1*H*-indole-3-carbaldehyde, 18

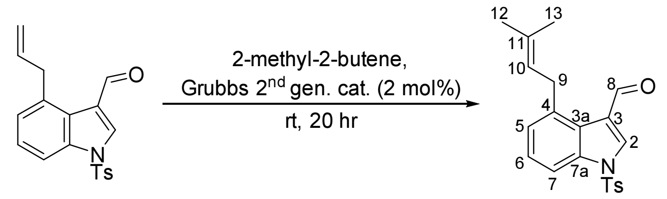

17 **18**

Finely ground indole **17** (2.50 g, 7.37 mmol) was suspended in neat 2-methyl-2-butene (75 mL) in a flame-dried 250 mL flask under nitrogen. Grubbs 2nd generation catalyst (117 mg, 0.14 mmol, 2 mol%) was added. The flask was flushed with nitrogen and the mixture was stirred at 23 °C, with periodic manual swirl. After 20 hr, NMR analysis of an aliquot indicated complete conversion (the product and starting material coelute on TLC).
The reaction mixture was diluted with 10 mL of DCM, the volatiles were evaporated, and the crude mixture was purified on Biotage Isolera system (45 g KP-Sil + 5 g Thelos NM loading cartridge, 5 → 35% EtOAc-hexane, 3-10-2 CV) to give the product as off-white amorphous solid.
Note: the starting material **17** is poorly soluble in 2-methyl-2-butene, and thus must be ground prior to use, to ensure complete conversion.

Yield: 2.49 g (92%).
Off-white amorphous solid.

TLC (25% EtOAc-*n*-pentane): R_f = 0.49.

^{1}H NMR ($CDCl_3$, 400 MHz): δ = 10.11 (s, 1H, H-8), 8.32 (s, 1H, H-Ar), 7.86−7.81 (m, 3H, 3× H-Ar), 7.33−7.29 (m, 3H, 3× H-Ar), 7.17 (d, *J* = 7.4 Hz, 1H, H-Ar), 5.26 (*app* t, *J* = 6.9 Hz, 1H, H-10), 3.86 (d, *J* = 6.8 Hz, 2H, H-9), 2.38 (s, 3H, H-TsC$\underline{H}_3$), 1.72 (s, 6H, H-12,13).

^{13}C NMR ($CDCl_3$, 100 MHz): δ = 185.5, 146.2, 136.9, 136.1, 136.1, 134.4, 133.5, 130.4, 127.5, 126.1, 125.4, 125.4, 123.9, 122.5, 111.3, 34.1, 25.9, 21.8, 21.8, 18.2.

HRMS (ESI$^+$), m/z: calcd for $C_{21}H_{21}NNaO_3S$ $[M+Na]^+$ 390.1134, found 390.1128.

IR (neat) ν_{max}: 3122 (w), 3048 (w), 2732 (w), 1688 (s), 1529 (m), 1377 (s), 1279 (m), 1177 (s).

1-(4-Bromo-1-tosyl-1*H*-indol-3-yl)-2,2-dimethylbut-3-en-1-ol, 19

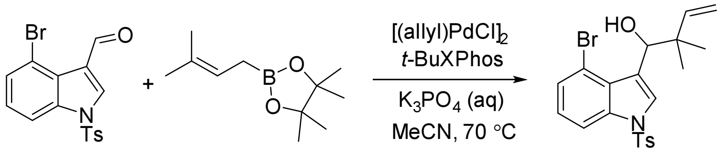

46 19

*An undesired product obtained during early attempts to prepare indole **18**.*

An oven-dried Schlenk flask was cooled under vacuum and filled with nitrogen. It was charged with $[(allyl)PdCl]_2$ (5.5 mg, 0.015 mmol), *t*-BuXphos (25.5 mg, 0.060 mmol) and 4-bromo-*N*-tosylindole-3-carbaldehyde (378 mg, 1.0 mmol), evacuated and refilled with nitrogen three times. Anhydrous CH_3CN (2.0 mL) and 2.5 M K_3PO_4 solution (2.0 mL) were then added. The resulting mixture was heated to 70 °C and stirred for 15 min. A solution of prenyl-Bpin **46** (196 mg, 1.0 mmol) in 1.0 mL of anhydrous CH_3CN was added to the reaction mixture at 70 °C over 12 h with a syringe pump. The reaction mixture was stirred at this temperature for additional 6 h. It was then cooled to 23 °C, diluted with 10 mL of EtOAc and 5 mL of H_2O, and the two phases were partitioned. The aq. phase was extracted with EtOAc (2×10 mL), and the combined organic phase was dried over Na_2SO_4, filtered and concentrated in vacuo. Purification by flash column chromatography (EtOAc:*n*-pentane, 1:2) afforded indole **19** (270 mg, 60%) as a pale yellow oil.

Yield: 270 mg (60%).
Pale yellow oil.

TLC (15% EtOAc-*n*-pentane): R_f = 0.21.

^{1}H NMR (400 MHz; $CDCl_3$): δ = 7.99 (d, *J* = 8.1 Hz, 1H, H-Ar), 7.73 (*app* d, *J* = 7.9 Hz, 3H, 3×H-Ar), 7.39 (d, *J* = 7.5 Hz, 1H, H-Ar), 7.21 (d, *J* = 8.4 Hz, 2H, 2×H-Ar), 7.11 (t, *J* = 8.1 Hz, 1H, H-Ar), 6.11 (dd, *J* = 17.6, 10.8 Hz, 1H, CH_2=C*H*), 5.75 (1 H, s, O-CH), 5.16 (dd, *J* = 10.8, 1.2 Hz, 1H, CH=CH*H*), 5.04 (dd, *J* = 17.6, 1.2 Hz, 1H, CH=C*H*H), 2.33 (s, 3H, CH_3), 2.07 (br s, 1H, OH), 1.13 (s, 1H, CH_3), 1.01 (s, 1H, CH_3)

^{13}C NMR (100 MHz; $CDCl_3$) δ = 145.5, 144.6, 135.8, 134.9, 130.1, 128.8, 128.6, 127.0, 126.7, 125.1, 123.9, 114.2, 114.1, 113.0, 71.1, 42.8, 25.1, 21.7

IR (solid) ν_{max}: 3563, 2967, 2928, 1596, 1551, 1411, 1368, 1172, 1099, 978, 811.

HRMS (ESI^+), m/z: calcd for $C_{21}H_{21}BrNO_2S$ $[M+H-H_2O]^+$ 430.0471, found 430.0464; calcd for $C_{21}H_{22}BrNNaO_3S$ $[M+Na]^+$ 470.0396, found 470.0383

Ethyl 5-(hydroxymethyl)-3-methylisoxazole-4-carboxylate, 21[20]

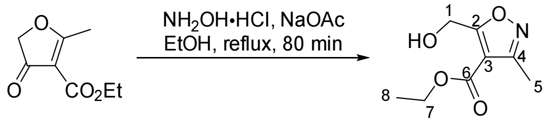

42 21

The known isoxazole **21** was prepared using a modified literature procedure.[20,21]
Furanone **42** (5.50 g, 32.3 mmol, 1.0 eq) was dissolved in anhydrous EtOH (30 mL), followed by $NH_2OH\cdot HCl$ (2.25 g, 32.3 mmol, 1.0 eq) and NaOAc (2.65 g, 32.3 mmol, 1.0 eq). The resulting suspension was heated at reflux for 80 min, when TLC (60% EtOAc-petrol) indicated complete consumption of the starting material.
Volatiles were evaporated. The residue was diluted with half-sat. NH_4Cl (100 mL) and extracted with 3×30 mL of Et_2O. The combined organic layers were washed with brine (30 mL), dried over Na_2SO_4 and concentrated. Drying under high-vacuum to remove traces of EtOH gave pale brown waxy solid, which was used as received (note: contains ~2% of isoxazole regioisomer). An analytically pure sample was prepared by chromatography on Biotage Isolera system (100 mg of crude **21** on 10 g SNAP Ultra cartridge, 10 → 50% EtOAc-petrol, 1-10-2 CV).

Yield: 5.87 g (98%).
Clear colorless oil.

TLC (50% EtOAc-petrol): R_f = 0.35.

^{1}H NMR (400 MHz, $CDCl_3$,): δ = 4.87 (br. d, *J* = 7.0 Hz, 2H, H-1), 4.35 (q, *J* = 7.2 Hz, 2H, H-7), 3.98 (br t, *J* = 7.0 Hz, 1H, OH), 2.43 (s, 3H, H-5), 1.38 (t, *J* = 7.2 Hz, 3H, H-8).

^{13}C NMR (100 MHz, $CDCl_3$): δ = 177.3 (C-2), 163.3 (C-6), 159.7 (C-4), 109.9 (C-3), 61.6 (7), 56.9 (C-1), 14.3 (C-8), 11.8 (C-5).

IR (neat) ν_{max}: 3401 (br. s), 1714 (s), 1606 (s), 1296 (s), 1104 (s).

Ethyl 3-methyl-5-((2*S*,3*R*)-3-(4-(3-methylbut-2-en-1-yl)-1-tosyl-1*H*-indol-3-yl)-1-((4-nitrophenyl)sulfonyl)-aziridin-2-yl)isoxazole-4-carboxylate, 24

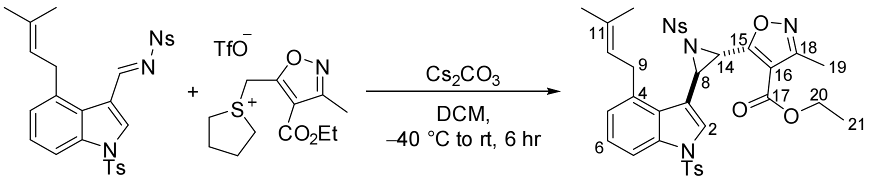

14 **15a** **24**
trans:*cis* = 9:1
J_{cis}(8-14) = 6.8 Hz
J_{trans}(8-14) = 4.7 Hz

Sulfonium salt **15a** (526 mg, 1.3 mmol, 1.3 eq) and anhydrous Cs_2CO_3 (423 mg, 1.3 mmol, 1.3 eq) were placed into a flame-dried Schlenk tube. Anhydrous DCM (14 mL) was added and the yellowish suspension was stirred at 0 °C for 1 hr, then at 23 °C for 1 hr, then recooled to −40 °C.
Neat imine **14** (550 mg, 0.99 mmol, 1.0 eq) was added in one portion, and the tube walls were washed with 2 mL of anhydrous DCM. The reaction mixture was stirred at −40 °C for 2 hr, then at 0 °C for 2 hr.
The reactants were quenched at 0 °C with 5 mL of half-sat. NaCl. The reaction mixture was then subjected to extraction (30 mL water – 3×20 mL DCM). The combined organic layer was dried over Na_2SO_4, and concentrated to give brown-orange foam. The crude material was analyzed by quantitative NMR and used as is.
Trans:*cis* = 9:1. The *trans*-isomer is highly unstable and isomerizes into the *cis*-isomer under acidic conditions (e.g. SiO_2, $CDCl_3$). The *trans/cis* ratios should be measured within 10 min of NMR sample preparation. Solutions in C_6D_6 are reasonably stable.

Yield: 823 mg (~63% pure by qNMR, 72% yield).
Yellow foam.

TLC (25% EtOAc-petrol): R_f = 0.44.

^{1}H NMR (400 MHz, CD_3CN, *trans*:*cis* = 3.7:1.0, *cis*-isomer marked as*): δ = 8.45 (d, *J* = 8.7 Hz, 2H, 2 × M-H-Ph_{Ns})*, 8.31 (d, *J* = 8.7 Hz, 2 × *o*-H-Ph_{Ns})*, 8.10 (d, *J* = 8.7 Hz, 2 × M-H-Ph_{Ns}), 7.79 (dd, *J* = 8.5, 2.3 Hz, 3H, *o*-H-Ph_{Ns}, 1 × H-Ar), 7.74 (d, *J* = 8.7 Hz, 2H, 2 × H-Ar), 7.61 (s, 1H, 1 × H-Ar), 7.31–7.20 (m, 3H, 3 × H-Ar), 6.98 (d, *J* = 7.5 Hz, 1H, 1 × H-Ar), 5.31 (d, *J* = 4.8 Hz, 1H, H-14), 5.17 (br m, 1H, H-10), 5.12 (br m, 1H, H-10)*, 4.97 (d, *J* = 6.8 Hz, 1H, H-14)*, 4.83 (d, *J* = 4.6 Hz, 1H, H-8), 4.76 (d, 6.7 Hz, 1H, H-8)*, 4.47 (q, *J* = 7.1 Hz, 2H, H-20), 4.31–4.21 (m, 2H, H-20)*, 3.80 (dd, *J* = 16.3, 7.6 Hz, 1H, H-9)*, 3.70 (dd, *J* = 16.3, 7.4 Hz, 1H, H-9), 3.45 (m, 1H, H-9), 2.49 (s, 3H, CH_3), 2.33 (s, 3H, CH_3), 2.33 (s, 3H, CH_3)*, 2.27 (s, 3H, CH_3)*, 1.82 (s, 3H, CH_3), 1.78 (s, 3H, CH_3)*, 1.72 (s, 3H, CH_3)*, 1.71 (s, 3H, CH_3), 1.50 (t, *J* = 7.1 Hz, 3H, H-21), 1.33 (t, *J* = 7.2 Hz, 3H, H-21)*.

^{13}C NMR (100 MHz, CD_3CN, *trans/cis* = 1.7:1.0): δ = 168.9 (C-15), 168.3 (C-15)*, 162.2 (C-Ar), 161.6 (C-Ar), 147.3 (C-Ar), 147.1 (C-Ar), 144.6 (C-Ar), 136.7 (C-Ar), 136.1 (C-Ar), 135.2 (C-Ar), 135.1 (C-Ar), 135.0 (C-11)*, 134.7 (C-11), 131.3 (C-Ar), 131.2 (C-Ar), 130.8 (C-Ar), 130.0 (C-Ar), 128.0 (C-Ar), 127.8 (C-Ar), 126.7 (C-Ar), 126.6 (C-Ar), 126.5 (C-Ar), 125.8 (C-Ar), 125.4 (C-Ar), 125.2 (C-Ar), 125.0 (C-Ar), 123.6 (C-Ar), 123.5 (C-Ar), 114.6 (C-Ar), 114.1

(C-Ar), 113.7 (C-Ar), 113.7 (C-Ar), 112.4 (C-Ar), 112.4 (C-Ar), 100.9 (C-Ar), 62.4 (C-10), 62.3 (C-10), 46.0 (C-8), 43.7 C-8)*, 42.8 (C-14)*, 41.0 (C-14), 32.9 (C-9), 32.5 (C-9), 25.7 (CH_3), 21.6 (CH_3), 18.2 ($\underline{C}H_3$), 18.2 ($\underline{C}H_3$), 14.5 (C-21), 14.4 (C-21), 11.7 ($\underline{C}H_3$), 11.7 ($\underline{C}H_3$).

HRMS (TOF ESI$^+$), m/z: calcd for $C_{35}H_{34}N_4O_9S$ [M+Na$^+$] 741.1665, found 741.1674.

IR (neat) ν_{max}: 3106 (br. w), 1719 (m), 1532 (m), 1348 (s), 1168 (s), 1090 (s), 854 (w), 742 (m).

SFC (15% MeCN, 125 bar CO_2, 4.0 mL/min, Whelk-O): t_R 10.32 min, 13.18 min.

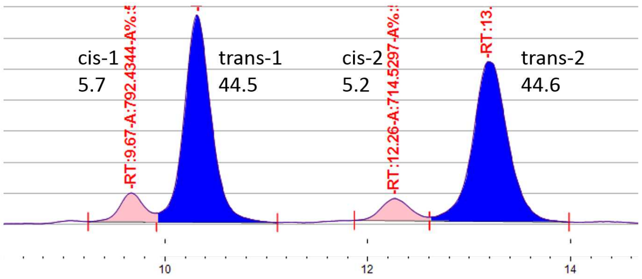

Ethyl 3-methyl-5-((2*R*,3*R*)-3-(4-(3-methylbut-2-en-1-yl)-1-tosyl-1*H*-indol-3-yl)-1-((4-nitrophenyl)sulfonyl)-aziridin-2-yl)isoxazole-4-carboxylate, *cis*-24

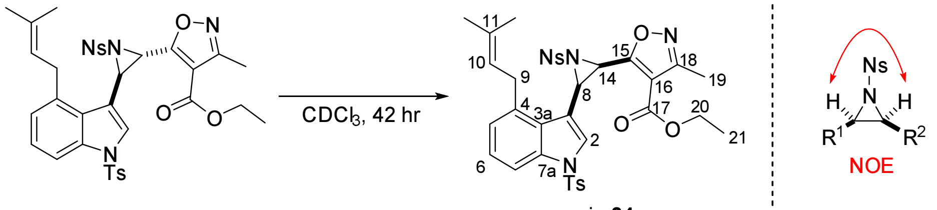

trans-**24**
d.r. ~5:1

cis-**24**

Crude aziridine **24** mixtures obtained during optimization experiments (10 samples combined, 650 mg, *trans/cis* ~5:1, purity ~40%) were dissolved in $CDCl_3$ (10 mL) in a scintillation vial. The yellow solution was aged for 42 hr, when NMR analysis of an aliquot indicated complete disappearance of the *trans*-isomer. The reaction mixture was evaporated and purified on Biotage (SNAP Ultra 25 g, 15 → 55% Et_2O-petrol, 1-10-6 CV). A small, analytically pure sample was obtained using reverse-phase prep-HPLC (ACE C18, 21.5×250 mm, 80 → 95% MeCN–water over 20 min, t_R = 12.5 min).

Yield: 120 mg (46%).
White solid.

TLC (50% Et_2O-petrol): R_f = 0.24.

Mp: 84–86 °C ($CDCl_3$).

^{1}H NMR (500 MHz, $CDCl_3$): δ = 8.48–8.43 (m, 2H, H-Ns), 8.34–8.29 (m, 2H, H-Ns), 7.71–7.61 (m, 4H, H-7, -2, -Ts), 7.19 (d, *J* = 8.3 Hz, 2H, H-Ts), 7.15 (dd, *J* = 8.4, 7.4 Hz, 1H, H-6), 6.96 (dd, *J* = 7.4, 0.9 Hz, 1H, H-5), 5.20–5.14 (m, 1H, H-10), 4.97 (d, *J* = 6.8 Hz, 1H, H-14), 4.82 (dd, *J* = 6.8, 1.1 Hz, 1H, H-8), 4.35–4.25 (m, 2H, H-20), 3.80 (dd, *J* = 16.3, 7.9 Hz, 1H, H-9a), 3.47 (br-d, *J* = 16.3 Hz, 1H, H-9b), 2.32 (s, 3H, H-Ts$\underline{CH_3}$), 2.28 (s, 3H, H-19), 1.82 (s, 3H, H-12-or-13), 1.79 (s, 3H, H-12-or-13), 1.34 (t, *J* = 7.1 Hz, 3H, H-21).

^{13}C NMR (125 MHz, $CDCl_3$): δ = 167.7 (C_q), 161.4 (C_q), 159.7 (C_q), 151.2 (C_q), 145.1 (C_q), 143.1 (C_q), 135.4 (C_q), 134.8 (C_q), 134.8 (C_q), 134.3 (C_q), 130.2 (C-Ts), 129.7 (C-Ns), 127.6 (C_q), 127.2 (C-Ts), 126.2 (C-2), 125.2 (C_q), 124.8 (C-Ns), 123.9 (C-5), 122.9 (C-11), 112.5 (C_q), 112.5 (C_q), 111.8 (C-7), 61.5 (C-20), 43.4 (C-8), 42.4 (C-14), 32.7 (C-9), 25.8 (C-12-or-13), 21.7 (C-Ts$\underline{CH_3}$), 18.2 (C-12-or-13), 14.3 (C-21), 11.7 (C-19).

HRMS (TOF ESI+), m/z: calcd for $C_{35}H_{34}N_4O_9S_2Na^+$ [M+Na]+ 741.1659, found 741.1653.

IR (neat) ν_{max}: 3107 (w), 1718 (s), 1533 (s), 1371 (s), 1348 (s), 1169 (s), 1091 (s), 743 (s).

SFC (15% MeCN, 125 bar CO_2, 4.0 mL/min, Whelk-O): t_R 10.18 min, 12.76 min.

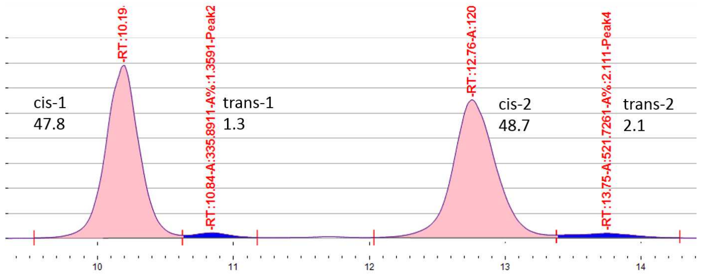

(±)-Ethyl 5-((6a*R*,9*S*,9a*R*)-7,7-dimethyl-8-((4-nitrophenyl)sulfonyl)-2-tosyl-6,6a,7,8,9,9a-hexahydro-2*H*-isoindolo[4,5,6-cd]indol-9-yl)-3-methylisoxazole-4-carboxylate, 25

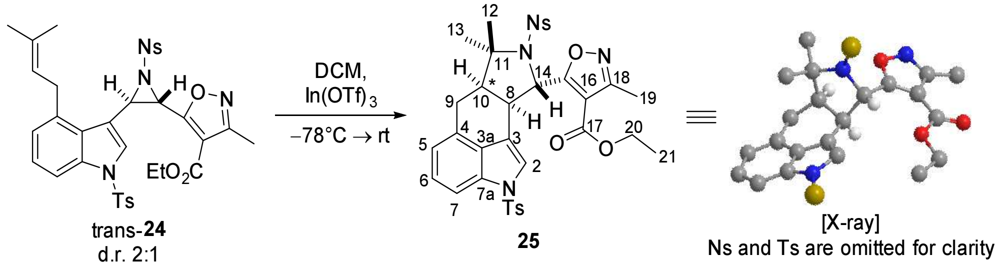

A solution of aziridine **24** (500 mg, 0.700 mmol, 1.0 eq.) in DCM (25 mL) was cooled to −78 °C for 30 min and $In(OTf)_3$ (782 mg, 1.40 mmol, 2.00 eq.) was added. After an additional 30 min at −78 °C the reaction mixture was warmed to 23 °C. After 30 min the reactants were quenched with $NaHCO_3$ (aq. sat., 24 mL), the aqueous layer was separated and extracted with DCM (3×30 mL). The combined organic layer was dried over $MgSO_4$, filtered and concentrated *in vacuo*.

Analysis by ^{1}H NMR indicated a 3.2:1 mixture of diastereomers at C-10 in favor of the desired isomer (30% qNMR yield). Purification using a Biotage Isolera system (SNAP KP-Sil 50g, 6 → 50% EtOAc-petrol 1-10-5 CV) followed by recrystallisation from hot MeCN/water (9:1) afforded cycloadduct **25** (120 mg, 0.17 mmol, 24%) as a colorless solid.

Yield: 120 mg (24%).
Colorless solid.

MP = 234-235 °C (MeCN).

TLC (25% EtOAc-petrol): R_f = 0.34.

^{1}H NMR (500 MHz, $CDCl_3$): δ = 8.27 (d, *J* = 8.9 Hz, 2H, 2 × M-H-Ph_{Ns}), 7.91 (d, *J* = 8.9 Hz, 2H, 2 × *o*-H-Ph_{Ns}), 7.71 (d, *J* = 8.4 Hz, 2H, 2 × *o*-H-Ph_{Ts}), 7.62 (d, *J* = 8.3 Hz, 1H, H-7), 7.25–7.16 (m, 4H, H-2, H-6, 2 × M-H-Ph_{Ts}), 6.97 (d, *J* = 7.3 Hz, 1H, H-5), 6.03 (d, *J* = 2.7 Hz, 1H, H-14), 4.41 (q, *J* = 7.1 Hz, 2H, H-20), 3.46 (m, 1H, H-8), 3.10 (dd, *J* = 17.5, 6.1 Hz, 1H, H-9b), 2.98 (td, *J* = 6.1, 3.4 Hz, 1H, H-10), 2.76 (dd, *J* = 17.5, 3.0 Hz, 1H, H-9a), 2.43 (s, 3H, H-19), 2.33 (s, 3H, Ts-C$\underline{H}_3$), 1.79 (s, 3H, H-13), 1.41 (t, *J* = 7.1 Hz, 3H, H-21), 0.79 (s, 3H, H-12).

^{13}C NMR (125 MHz, $CDCl_3$): δ = 176.3 (C-15), 162.1, 159.8 (C-17, C-18), 150.0 (C-*p*-Ph_{Ns}), 145.8 (C-*i*-Ph_{Ns}), 145.1 (C-*p*-Ph_{Ts}), 135.2, 133.4 (C-7a, C-*i*-Ph_{Ts}), 130.1 (C-M-Ph_{Ts}), 129.4 (C-4), 128.9, 128.9 (C-3a, C-*o*-Ph_{Ns}), 126.9 (C-*o*-Ph_{Ts}), 126.3 (C-2), 124.2 (C-M-Ph_{Ns}), 121.0, 120.8 (C-5, C-6), 117.4 (C-3), 111.7 (C-7), 109.3 (C-16), 70.6 (C-11), 61.5 (C-20), 60.0 (C-14), 47.4 (C-10), 40.3 (C-8), 30.4 (C-13), 25.7 (C-9), 23.7 (C-12), 21.7 (Ts-$\underline{C}H_3$), 14.4 (C-19), 12.0 (C-21).

HRMS (MALDI), m/z: calcd for $C_{35}H_{34}NaN_4O_9S$ $[M+Na]^+$ 741.1665, found 741.1670.

IR (neat) ν_{max}: 2984 (br. w), 1721 (m), 1532 (s), 1351 (s), 1177 (s), 1166 (s), 1093 (s), 855 (w), 737 (m).

Table 8. Crystal data and structure refinement for compound (±)-**25**.

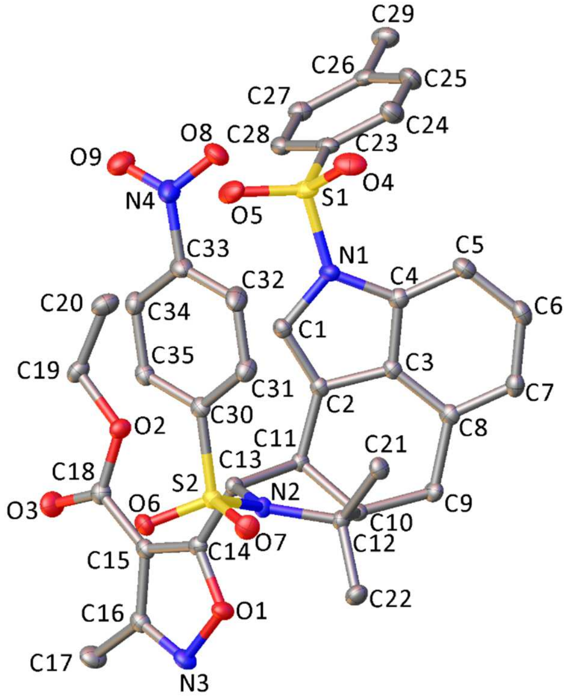

Illustration of the structure of (±)-**25** with atomic numbering scheme depicted. Thermal ellipsoids depicted at the 50% probability level. Only one position of the disordered atom O8 is shown and the acetonitrile solvent molecules and hydrogen atoms omitted for clarity.

Identification code	**25**
CCDC number	**1584104**
Empirical formula	$C_{38}H_{38.5}N_{5.5}O_9S_2$
Formula weight	780.36
Temperature/K	100(2)
Crystal system	triclinic
Space group	*P*-1
a/Å	12.3280(3)
b/Å	13.1199(4)
c/Å	13.5679(3)
α/°	98.3503(15)
β/°	115.0793(14)
γ/°	105.4667(16)
Volume/Å^3	1829.87(9)
Z	2
ρ_{calc}g/cm^3	1.416
μ/mm^{-1}	0.210
F(000)	818.0
Crystal size/mm^3	0.391 × 0.319 × 0.145
Radiation	MoKα (λ = 0.71073)
2θ range for data collection/°	3.47 to 55.998
Index ranges	-16 ≤ h ≤ 16, -17 ≤ k ≤ 17, -17 ≤ l ≤ 17
Reflections collected	33854
R_{int} / R_{sigma}	0.0374 / 0.0354

Data/restraints/parameters	8814/9/534
Goodness-of-fit on F^2	1.046
Final R indexes [I>=2σ (I)]	R_1 = 0.0390, wR_2 = 0.0922
Final R indexes [all data]	R_1 = 0.0524, wR_2 = 0.0983
Largest diff. peak/hole / e $Å^{-3}$	0.46/-0.49

Note: Occupancies of disordered fragments refined before being fixed. Restraints applied to maintain sensible thermal and geometric parameters.

5-((6a*R*,9*S*,9a*R*)-7,7-Dimethyl-2-tosyl-6,6a,7,8,9,9a-hexahydro-2*H*-isoindolo[4,5,6-cd]indol-9-yl)-3-methylisoxazole-4-carboxylic acid, 26

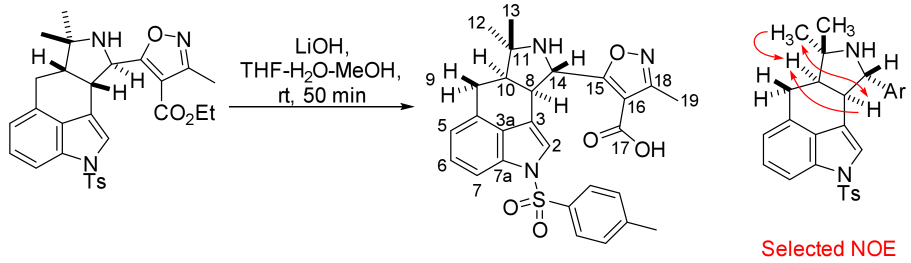

44 **26**

The starting ester **44** (19 mg, 0.036 mmol) was dissolved in THF (0.5 mL) in a 3 mL vial. Solution of LiOH in water (0.5 mL, 1 M) was added, resulting in a biphasic emulsion. MeOH (0.3 mL) was added dropwise until the layers mixed. The resulting cloudy white mixture was stirred at 23 °C for 50 min, when LCMS (50→90% MeCN-water) indicated complete consumption of the starting material ($t_{1/2}$ ≈ 4 min).
The reactants were quenched with sat. NH_4Cl (5 mL) and the mixture was then extracted with DCM (4×2 mL). The combined organic layer was washed with brine (2 mL), dried over Na_2SO_4, and concentrated to give yellowish solid, which was used in the next step as received. A small portion was purified by silica chromatography (2 → 5 → 10% MeOH-DCM) to give analytically pure material as white solid.

Yield: 15 mg (83%).
White solid.

Mp: decomposes without melting above 100 °C.

TLC (10% MeOH-DCM): R_f = 0.46; (100% EtOAc): R_f = 0.03.

^{1}H NMR (500 MHz, DMSO-d$_6$): δ = 7.86 (d, *J* = 8.4 Hz, 2H, H-Ts), 7.67 (d, *J* = 8.2 Hz, 1H, H-7), 7.40 (d, *J* = 1.0 Hz, 1H, H-2), 7.37 (d, *J* = 8.7 Hz, 2H, H-Ts), 7.31 (t, *J* = 8.0, 7.0 Hz, 1H, H-6), 7.12 (d, *J* = 7.3 Hz, 1H, H-5), 4.77 (d, *J* = 9.0 Hz, 1H, H-14), 3.97 (dd, *J* = 8.7, 6.9 Hz, 1H, H-8), 3.01 (dd, *J* = 16.8, 6.2 Hz, 1H, H-9a), 2.90 (dd, *J* = 16.8, 9.4 Hz, 1H, H-9b), 2.49−2.45 (m, 1H, H-10), 2.44 (s, 3H, H-19), 2.31 (s, 3H, H-TsCH$_3$), 1.40 (s, 3H, H-12), 1.19 (s, 3H, H-13).

^{13}C NMR (125 MHz, DMSO-d$_6$): δ = 170.6 (C-15), 162.8 (C_q), 161.2 (C_q), 145.4 (C-34), 134.2 (C_q), 132.2 (C_q), 130.2 (C-33, -35), 130.1 (C_q), 127.7 (C_q), 126.7 (C-32, -36), 125.9 (C-6), 122.1 (C-2), 121.0 (C-5), 112.8 (C_q), 110.8 (C-7), 63.6 (C-11), 57.6 (C-14), 46.7 (C-10), 40.8 (C-8), 27.7 (C-12), 24.4 (C-9), 22.7 (C-13), 21.0 (C-37), 11.6 (C-19).

HRMS (TOF ESI$^+$), m/z: calcd for $C_{27}H_{28}N_3O_5S$ [M+H]$^+$ 506.1744, found 506.1748.

IR (neat) ν_{max}: 1617 (m), 1435 (m), 1388 (s), 1369 (s), 1174 (s). The IR spectrum suggests that **26** exists in a zwitterionic form in solid state.

(6a*S*,12b*R*,12c*S*)-7,7,10-Trimethyl-2-tosyl-2,6,6a,7,12b,12c-hexahydro-9*H*-isoxazolo[5'',4'':3',4']pyrrolo-[1',2':2,3]isoindolo[4,5,6-cd]indol-9-one, 27

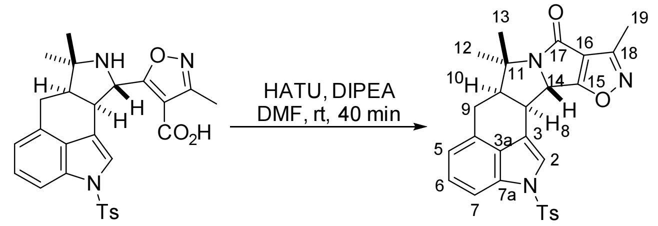

26 **27**

The starting amino acid **26** (40 mg, 0.079 mmol, 1.0 eq) was dissolved in anhydrous DMF (1.2 mL) in a flame-dried 3 mL vial, followed by HATU (36 mg, 0.95 mmol, 1.2 eq) and DIPEA (28 µL, 0.16 mmol, 2 eq). The clear reaction mixture was stirred at 23 °C for 30 min (LCMS [50→90% MeCN-water] indicated complete consumption of the starting material within 3 min). The reaction mixture was diluted with water (5 mL) and extracted with DCM (3×2 mL). The combined organic layer was washed with brine (2 mL), dried over Na_2SO_4 and concentrated to dryness. The crude material was purified on Biotage Isolera system (5 g ZIP KP-Sil, 10→60% EA-petrol, 1-10-2 CV).

Yield: 31 mg (80%).
Off-white foam.

TLC (60% EtOAc-petrol): R_f = 0.50.

^{1}H NMR (500 MHz, Chloroform-d) δ = 7.83 (d, J=8.3, 2H, H-Ts), 7.79 (d, J=8.0, 1H, H-5/7), 7.50 (s, 1H, H-2), 7.31 (dd, J=7.5, 8.0, 1H, H-6), 7.26 (d, J=8.0, 2H, H-Ts), 7.08 (d, J=7.5, 1H, H-7/5), 4.68 (d, J=11.2, 1H, H-14), 3.54 (dd, J=11.2, 5.6, 1H, H-8), 3.11 – 2.97 (m, 2H, H-9), 2.71 – 2.63 (m, 1H, H-10), 2.45 (s, 3H, H-19), 2.35 (s, 3H, H-TsCH$_3$), 1.66 (s, 3H, H-13/12), 1.64 (s, 3H, H-12/13).

^{13}C NMR (125 MHz, Chloroform-d) δ = 184.9 (C_q), 163.7 (C_q), 155.2 (C_q), 145.2 (C_q), 135.5 (C_q), 133.4 (C_q), 130.1 (C-Ts), 129.6 (C_q), 128.1 (C_q), 127.1 (C-Ts), 126.2 (C-6), 122.2 (C-2), 121.5 (C_q), 121.2 (C-5/7), 115.5 (C_q), 111.9 (C-5/7), 63.5 (C-14), 62.9 (C-11), 53.5 (C-10), 36.0 (C-8), 26.2 (C-9/12/13), 25.9 (C-9/12/13), 24.9 (C-12/13), 21.8 (C-TsCH$_3$), 10.1 (C-19).

HRMS (MALDI), m/z: calcd for $C_{27}H_{25}N_3O_4SNa^+$ $[M+Na]^+$ 510.1458, found 510.1451.

IR (neat) ν_{max}: 3110 (w), 1701 (s), 1366 (s), 1177 (s).

(−)-*N*-Ts α-CPA imine, 28

Method A: by one-pot carbonylative lactamization / N-O cleavage

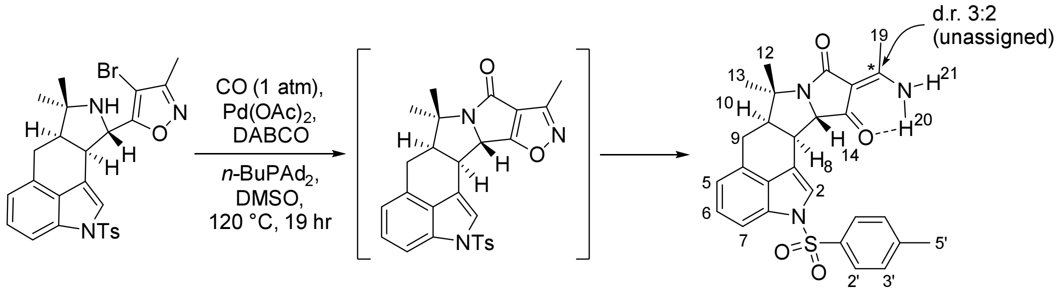

34 **27** putative intermediate **28**

The starting material **34** (65 mg, 0.12 mmol), DABCO (42 mg, 0.37 mmol, 3.1 eq), *n*-BuPAd$_2$ (54 mg, 0.15 mmol, 1.2 eq), naphthaline (2 mg, internal standard), and $Pd(OAc)_2$ (1.5 mg, 0.006 mmol, 5 mol%) were placed into a

flame-dried thick-walled tube (20×125 mm) and back-filled with nitrogen (3 times). Anhydrous degassed DMSO was added (1.8 mL), giving yellow suspension. The overhead space was flushed with CO (1 atm, balloon) for 5 min through a 21G×120 mm Sterican needle under sonication. The reaction was then placed into a pre-heated oil bath (120 °C) and stirred at this temperature under static CO atmosphere for 19 hr.
The deep-red, almost black, reaction mixture was cooled to 23 °C and diluted with DCM (10 mL) and sat. NH_4Cl (20 mL), then extracted with DCM (4×5 mL). The combined organic layer was washed with brine (10 mL), dried over Na_2SO_4 and concentrated. Purification by chromatography on silica (70% EtOAc-petrol) afforded the desired product as a deep red wax. A small sample was purified by prep-HPLC (ACE C18, 21×250 mm, 70→95% MeCN-H_2O over 25 min) to yield white foam.

Yield: 48 mg (80%).
White foam.

Method B: by the reduction of N-O bond.

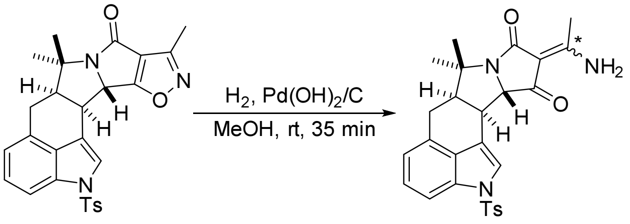

rac-27 **rac-28**
d.r. ~ 1:1

The starting isoxazole **27** (9.0 mg, 0.018 mmol) was dissolved in HPLC-grade MeOH (1 mL) in a 3 mL vial, followed by $Pd(OH)_2/C$ (3.0 mg, 20 wt%, wet with 50% water, 0.002 mmol, 0.1 eq). Hydrogen was carefully bubbled through the solution for 5 min, after which the mixture was stirred under static hydrogen pressure for 30 min. LCMS (50→90% MeCN-water) indicated complete consumption of the starting material.
The crude reaction mixture was filtered through Celite (0.5×1 cm), washing with 5 mL of MeOH. The volatiles were evaporated and the residue was purified by column chromatography (70% EtOAc-petrol).

Yield: 7 mg (80%).
White amorphous solid.

Characterization Data

TLC (80% EtOAc-*n*-pentane): R_f = 0.37.

Note: Analysis of NMR spectra is greatly complicated by the existence of the *E*- and *Z*-isomers as inseparable 1:1−1.5:1 mixtures. The ^{1}H NMR signals could be assigned to the individual isomers only with moderate confidence. The ^{13}C NMR spectrum is reported as is.

^{1}H NMR ($CDCl_3$, 500 MHz): δ = 10.02 (br s, 0.4×1H, H-20), 9.84 (br d, *J* = 5.7 Hz, 0.6×1H, H-20), 7.85−7.79 (m, 2H, H-2'), 7.73 (d, *J* = 8.2 Hz, 1H, H-7), 7.54 (s, 0.6×1H, H-2), 7.50 (s, 0.4×1H, H-2,), 7.26−7.21 (m, 1H, H-6), 7.22 (d, *J* = 8.3 Hz, 2H, H-3'), 7.04−6.99 (m, 1H, H-5), 5.76 (br s, 0.4×1H, H-21), 5.67 (br d, *J* = 5.7 Hz, 1H, 0.6×1H, H-21), 3.96 (d, *J* = 11.0 Hz, 0.4×1H, H-14), 3.91 (d, *J* = 10.9 Hz, 0.6×1H, H-14), 3.57−3.48 (m, 1H, H-8), 3.03−2.87 (m, 2H, H-9), 2.51 (s, 0.4×3H, H-19), 2.55−2.45 (m, 1H, H-11), 2.49 (s, 0.6×3H, H-19), 2.33 (s, 3H, H-5'), 1.61 (s, 0.4×3H, H-12/13), 1.60 (s, 0.6×3H, H-12/13), 1.58 (s, 0.4×3H, H-13/12), 1.56 (s, 0.6×3H, H-13/12).

^{13}C NMR ($CDCl_3$, 125 MHz): δ = 198.7, 196.3, 175.4, 172.5, 167.7, 167.3, 144.8, 144.7, 135.77, 135.73, 133.27, 133.20, 130.15, 130.02, 129.96, 128.80, 128.78, 127.08, 127.05, 125.6, 125.5, 123.0, 122.7, 120.8, 120.7, 117.5, 117.4, 111.6, 101.5, 70.1, 69.1, 63.0, 62.9, 52.9, 52.8, 35.7, 26.41, 26.38, 26.04, 25.98, 25.3, 25.2, 21.7, 20.1, 19.2.

HRMS ($MALDI^+$), m/z: calcd for $C_{27}H_{27}N_3O_4S$ $[M+H]^+$ 490.1795, found 490.1788.

IR (neat) ν_{max}: 3312 (m), 3171 (br. m), 1671 (m), 1616 (s), 1521 (s), 1362 (s), 1177 (s).

$[\alpha]_D^{21}$ = −6° (c = 0.32, $CHCl_3$).

4-Bromo-5-(hydroxymethyl)-3-methylisoxazole, 29[22]

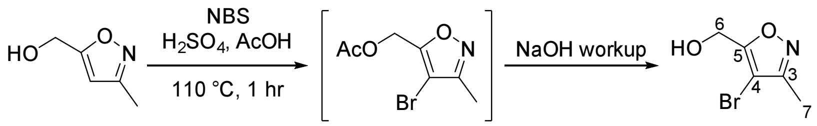

35 **36** **29**

The starting isoxazole **35** (3.00 g, 26.7 mmol) was dissolved in reagent grade acetic acid (130 mL) in a 250 mL round-bottom flask, followed by *N*-bromosuccinimide (NBS, 5.67 g, 31.9 mmol, 1.2 eq) and conc. H_2SO_4 (2.8 mL, 53 mmol, 2.0 eq). The brownish reaction mixture was stirred at 23 °C for 5 min, until most of solid dissolved. Then, a reflux condenser was attached, the overhead space was exchanged with nitrogen (3 times), and the reaction was stirred at 110 °C for 1 hr.
The reaction mixture was cooled down to 23 °C, diluted with 30 mL of ether, and concentrated (20 Torr, 30 → 35 °C) to approx. 1/10 of the original volume. The resulting brown oil was dissolved in methanol (60 mL), placed into an ice bath, and treated with 50 mL of 2 M NaOH, followed by solid NaOH in 0.5 g portions, until pH 10. This allows for quenching the remaining acid, as well as hydrolyses the intermediate acetate **36** almost instantly (TLC [40% Et_2O-*n*-pentane]: acetate **36** R_f = 0.65, alcohol **29** R_f = 0.38). The mixture was then diluted with sat. NH_4Cl (250 mL) and extracted with Et_2O (4×50 mL). The combined organic layer was washed with brine (50 mL), dried over Na_2SO_4 and concentrated to give brown oil. Purification on Biotage Isolera system (SNAP KP-Sil 50 g, 10 → 80% Et_2O-*n*-pentane, 1-10-2 CV) yielded the product as off-white solid.

Yield: 4.33 g (85%).
Off-white solid.

TLC (40% Et_2O-*n*-pentane): R_f = 0.38.

Mp: 63−65 °C (Et_2O).

^{1}H NMR ($CDCl_3$, 400 MHz): δ = 4.72 (d, J = 6.6 Hz, 2H, H-6), 2.84 (br s, 1H, OH), 2.27 (s, 3H, H-7).

^{13}C NMR ($CDCl_3$, 100 MHz): δ = 167.0 (C_q), 159.7 (C_q), 93.7 (C-4), 54.9 (C-6), 10.6 (C-7).

IR (neat) ν_{max}: 3360 (br.s), 1606 (w), 1439 (m), 1406 (m), 1084 (s), 1041 (s).

1-((4-Bromo-3-methylisoxazol-5-yl)methyl)tetrahydrothiophen-1-ium trifluoromethanesulfonate, 31a

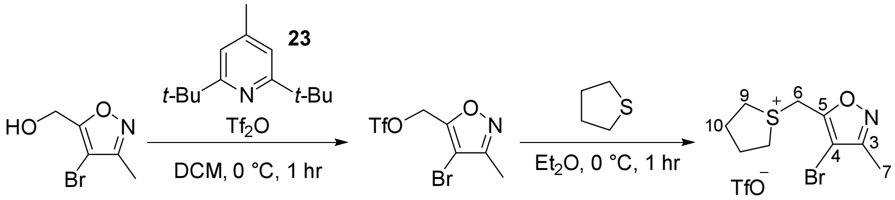

29 **30** **31a**

Step 1. The starting alcohol **29** (100 mg, 0.52 mmol) and 2,6-di(*t*-butyl)-4-methylpyridine **23** (128 mg, 0.63 mmol, 1.2 eq) were dissolved in anhydrous DCM (5 mL) in a flame-dried Schlenk tube and cooled to 0 °C. Then, Tf_2O (0.10 mL, 0.63 mmol, 1.2 eq) was added and the resulting clear colorless solution was stirred at 0 °C. In 5 min, white precipitate was observed. After 1 hr at 0 °C, the reaction mixture was quickly filtered through a 1.5×1 cm (i.d./h) pad of silica (pre-washed with 12 mL of anhydrous DCM) into a flame-dried flask (25 mL, pear-shaped), washing the filter cake with 7 mL of anhydrous DCM. The filtrate was concentrated to give yellowish-white waxy solid of the intermediate triflate **30**, which was used immediately in the next step.

Step 2. The triflate **30** was suspended in 4 mL of anhydrous Et_2O in the same flask, cooled to 0 °C (*note*: the compound is only partially soluble in this solvent), and rapidly stirred at 600 rpm. Neat tetrahydrothiophene was added dropwise, and the resulting white suspension was stirred at 0 °C for 1 hr. The solids were then filtered and washed with 20 mL of ether to provide the crude product as off-white wax. Purification by column chromatography (2 → 20% MeOH-DCM) gave the target material as white powder.

Yield: 200 mg (93%).
White powder.

TLC (15% MeOH-DCM): R_f = 0.20.

Mp: 88−90 °C (DCM).

^{1}H NMR (CD_3CN, 500 MHz): δ = 4.57 (s, 2H, H-8), 3.66−3.52 (m, 2H, H-10a), 3.51−3.39 (m, 2H, H-10b), 2.31 (s, 3H, H-6), 2.29−2.22 (m, 4H, H-11).

^{13}C NMR (CD_3CN, 125 MHz): δ = 161.8 (C_q), 158.7 (C_q), 100.1 (C-2), 45.4 (C-10), 35.9 (C-8), 29.6 (C-11), 10.8 (C-6). Note: the CF_3 group is not seen.

^{19}F NMR (CD_3CN, 377 MHz) δ = −79.3.

IR (neat) ν_{max}: 1605 (w), 1416 (w), 1260 (s), 1223 (s), 1158 (s), 1073 (m), 1031 (s).

(−)-(1*S*,1'*R*,2'*S*,4*R*,4'*S*)-3'-((4-Bromo-3-methylisoxazol-5-yl)methyl)-7,7-dimethyl-2-oxo-3'-thia[1,2'-bi(bicyclo[2.2.1]heptan)]-3'-ium trifluoromethanesulfonate, 31b

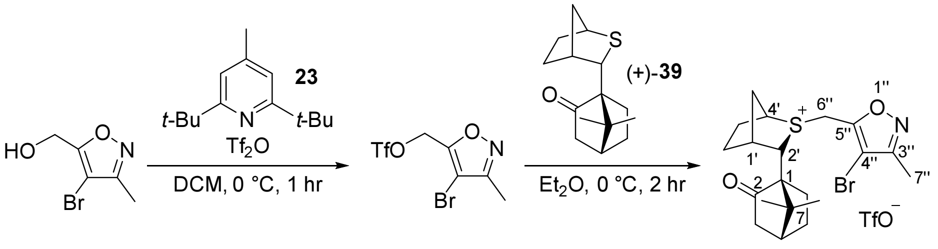

Step 1. The starting alcohol **29** (250 mg, 1.30 mmol) and 2,6-di(*t*-butyl)-4-methylpyridine **23** (320 mg, 1.56 mmol, 1.2 eq) were dissolved in anhydrous DCM (9 mL) in a flame-dried Schlenk tube and cooled to 0 °C. Then, Tf_2O (0.26 mL, 1.56 mmol, 1.2 eq) was added and the resulting clear colorless solution was stirred at 0 °C. In 5 min, white precipitate was observed. After 1 hr at 0 °C, the cold reaction mixture was passed through a 1.5×1 cm (i.d. × h) pad of silica (pre-washed with 12 mL of anhydrous DCM) into a flame-dried flask (50 mL, pear-shaped), washing the filter cake with 30 mL of anhydrous DCM. The filtrate was concentrated to give off-white waxy solid of the intermediate triflate **30**, which was used immediately in the next step.
Step 2. The triflate **30** was suspended in 10 mL of anhydrous Et_2O in the same flask, cooled to 0 °C (note: the compound is only partially soluble in this solvent), and rapidly stirred at 600 rpm. A solution of (+)-**39** (143 mg, 0.57 mmol, 1.1 eq) in anhydrous Et_2O (3 mL) was added dropwise, and the resulting suspension was stirred at 0 °C for 2 hr.
After 2 hr at 0 °C, the precipitate was filtered off, washed with 30 mL of Et_2O and dried in air. Purification on Biotage Isolera system (SNAP Ultra 10 g, 2 → 20% MeOH-DCM, 1-10-2 CV, loaded in DCM) provided the desired product as off-white powder.
Notes: 1. The filter cake in step 1 needs to be washed with sufficient amount of DCM to ensure complete elution of triflate **30**. 2. Failure to pre-dissolve the sulfide (+)-**39**, or too rapid addition of it, leads to coagulation of the reaction mixture, poor yields and hard-to-purify mixtures.

Yield: 595 mg (80%).
White solid.

TLC (15% MeOH-DCM): R_f = 0.49.

Mp: 110 °C (dec.).

^{1}H NMR ($CDCl_3$, 500 MHz): δ = 5.00 (d, *J* = 15.3 Hz, 1H), 4.95 (br s, 1H), 4.57 (d, *J* = 15.2 Hz, 1H), 4.36 (d, *J* = 5.0 Hz, 1H), 3.26 (br t, *J* = 4.0 Hz, 1H), 2.97 (d, *J* = 13.1 Hz, 1H), 2.61 (dt, *J* = 18.9, 4.0 Hz, 1H), 2.46 (td, *J* = 13.0, 12.3, 3.7 Hz, 1H), 2.37-2.30 (m, 4H), 2.30−2.13 (m, 4H), 2.02 (d, *J* = 18.9 Hz, 1H), 1.78−1.57 (m, 4H), 1.50−1.42 (m, 1H), 1.28 (s, 3H, CH_3), 1.13 (s, 3H, CH_3).

^{13}C NMR ($CDCl_3$, 125 MHz): δ = 216.0 (C_{CO}), 160.8 (C_q), 158.1 (C_q), 120.9 (q, *J* = 320.0 Hz, C_{CF3}), 98.7 (C_q), 69.9 (CH), 60.7 (C_q), 59.7 (CH), 50.4 (C_q), 45.3 (CH), 44.2 (CH_2), 43.6 (CH), 41.5 (CH_2), 36.6 (CH_2), 33.3 (CH_2), 26.98 (CH_2), 26.96 (CH_2), 24.5 (CH_2), 22.0 (CH_3), 19.6 (CH_3), 10.8 (CH_3).

^{19}F NMR ($CDCl_3$, 377 MHz): δ = −78.3.

HRMS (TOF ESI$^+$), m/z: calcd for $C_{20}H_{27}BrNO_2S$ [R_3S^+] 424.0940, found 424.0940.

IR (neat) ν_{max}: 1730 (m), 1265 (s), 1258 (s), 1146 (m), 1028 (s), 636 (s).

$[\alpha]_D^{23}$ = −48° (*c* = 0.63, $CHCl_3$).

(−)-(1*R*,4*R*,5*R*,6*R*)-6-((4-Bromo-3-methylisoxazol-5-yl)methyl)-4,7,7-trimethyl-6-thiabicyclo[3.2.1]octan-6-ium trifluoromethanesulfonate, (−)-31c

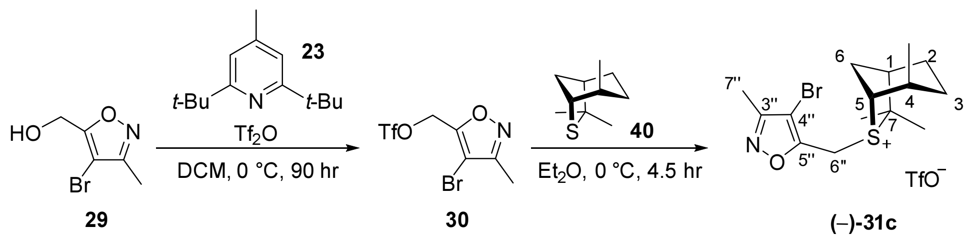

The starting alcohol **29** (300 mg, 1.56 mmol) and 2,6-di(*t*-butyl)-4-methylpyridine **23** (384 mg, 1.88 mmol, 1.2 eq) were dissolved in anhydrous DCM (12 mL) in a flame-dried Schlenk tube and cooled to 0 °C. Then, Tf_2O (0.32 mL, 1.9 mmol, 1.2 eq) was added and the resulting clear colorless solution was stirred at 0 °C. In 5 min, white precipitate was observed. After 90 min at 0 °C, the solids were removed by filtration through a 1×3 cm (i.d./h) pad of silica (pre-washed with 22 mL of anhydrous DCM) into a flame-dried 50 mL flask, washing the filter cake with 20 mL of anhydrous DCM. The filtrate was concentrated to give yellowish-white waxy solid of the intermediate triflate **30**, which was used immediately in the next step.
The triflate **30** was suspended in anhydrous Et_2O (12 mL) and cooled to 0 °C. A solution of the chiral auxiliary **40** (0.32 mL, 1.9 mmol, 1.2 eq) in anhydrous Et_2O (4 mL) was added dropwise and the resulting mixture was stirred at 0 °C for 4.5 hr.
The white suspension was then carefully filtered, attention being given not to transfer any of the brown wax covering the flask walls. The filter cake was washed with Et_2O (20 mL) and dried under vacuum to give the product as ivory powder.
Notes:
1. It is important to add the auxiliary **40** slowly as a solution in Et_2O, otherwise the reaction mixture is prone to aggregation and the product purity suffers.
2. During the reaction, the product crushes out as nice free-flowing white solid, while the unreacted triflate **30** and the side-products remain stuck to the flask walls as brown wax.

3. The compound **31c** is unstable in $CDCl_3$, acidic, or protic solvents ($t_{½}$[$CDCl_3$] ~90 min). The decomposition product is alkene **41** (cf. ref [[23,24]]). Our early attempts to prepare **31c** in the DCM-water mixtures led to the exclusive formation of **41**.

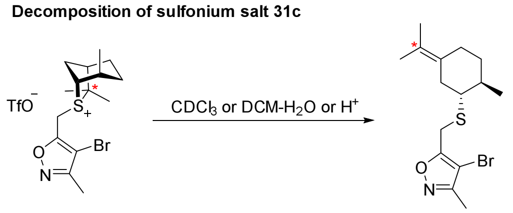

31c **41**

4. We could not identify the means to purify **31c**, therefore it is paramount that it is generated in sufficient purity straight from the reaction.

Yield: 368 mg (47%).
Ivory powder.

TLC (10% MeOH-DCM): R_f = 0.22.

Mp: 85−86 °C (Et_2O).

^{1}H NMR (CD_3CN, 500 MHz): δ = 4.68 (d, J = 14.5 Hz, 1H, H-26a), 4.55 (d, J = 14.5 Hz, 1H, H-26b), 4.09 (br s, 1H, H-13), 2.56−2.42 (m, 3H, H-10, -12), 2.40−2.33 (m, 1H, H-14), 2.30 (s, 3H, H-25), 1.81 (s, 4H), 1.76−1.63 (m, 5H), 1.59 (m, 1H), 1.15 (d, J = 7.0 Hz, 3H, H-18).

^{13}C NMR (CD_3CN, 125 MHz): δ = 161.9 (C_q), 158.6 (C_q), 99.4 (C-21), 76.8 (C-9), 68.2 (C-13), 51.1 (C-10), 33.0, 32.9, 32.7, 26.1, 25.8, 23.4, 22.5, 17.8 (C-18), 10.8 (C-25). *Note:* the CF_3 group is not seen.

^{19}F NMR (CD_3CN, 377 MHz): δ = −79.3.

HRMS (TOF ESI+), m/z: calcd for $C_{15}H_{23}BrNOS$ [M^+] 344.0678, found 344.0691.

IR (neat) ν_{max}: 1281 (s), 1254 (s), 1151 (s), 1033 (s), 637 (s).

$[\alpha]_D^{22}$ = −41° (c = 1.0, $CHCl_3$).

1-((4-Bromo-3-methylisoxazol-5-yl)methyl)tetrahydrothiophen-1-ium tetrafluoroborate, 31d

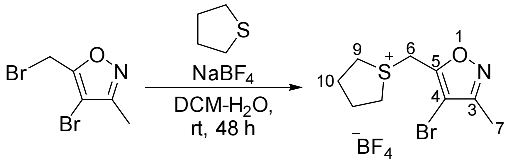

38 **31d**

To a solution of **38** (2.35 g, 9.22 mmol) in 18 mL of degassed DCM were added tetrahydrothiophene (2.44 mL, 27.7 mmol), degassed water (6 mL) and $NaBF_4$ (3.04 g, 27.7 mmol), sequentially. The resulting biphasic mixture was stirred vigorously (1000 rpm) at 23 °C under nitrogen for 48 hr. It was then diluted with water (10 mL) and DCM (50 mL). The two layers were partitioned, and the aqueous layer was extracted further with DCM (3×15 mL). The combined organic layer was dried over Na_2SO_4, filtered and concentrated to give a white solid. The crude product was dissolved in 30 mL of DCM and added dropwise to rapidly stirring Et_2O (250 mL). The flask containing the crude product was rinsed with additional DCM (2×5 mL) and added to Et_2O. An immediate white solid formation was observed. The mixture was stirred in an ice-water bath for 30 min and then kept chilled and unstirred for 2 h. The white solid was filtered (gravity), washed with Et_2O and dried *in vacuo* to afford sulfonium salt **31d** (2.276 g, 70%) as a white solid.

Yield: 2.28 g (70%).
White solid.

Mp: 120−121 °C (Et_2O/DCM).

TLC (15% MeOH-DCM): R_f = 0.15.

^{1}H NMR (CD_3CN, 400 MHz): δ = 4.57 (s, 2H, H-6), 3.64 – 3.54 (m, 2H, H-9a), 3.50 – 3.40 (m, 2H, H-9b), 2.30 (s, 3H, H-7), 2.29 – 2.22 (m, 4H, H-10).

^{13}C NMR (CD_3CN, 100 MHz): δ = 161.8 (C_q), 158.7 (C_q), 100.1 (C_q), 45.3 (C-9), 35.8 (C-6), 29.5 (C-10), 10.8 (C-7).

HRMS (ESI), m/z: calcd for $C_9H_{13}Br^{79}NOS^+$ [R_3S^+] 261.9896, found 261.9886.

IR (neat) ν_{max}: 3001, 2955, 1610, 1430, 1415, 1286, 1014.

(+)-4-Bromo-3-methyl-5-((2*S*,3*S*)-3-(4-(3-methylbut-2-en-1-yl)-1-tosyl-1*H*-indol-3-yl)-1-((4-nitrophenyl)-sulfonyl)aziridin-2-yl)isoxazole, (+)-*cis*-32

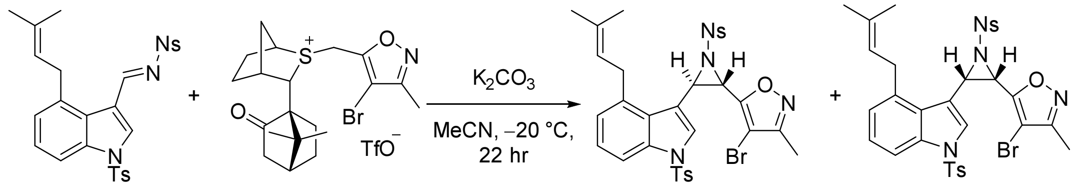

14 **31b** ***trans-32*** [unstable] er 98:2 9:1 **(+)-*cis-32*** er 89:11

The starting imine **14** (410 mg, 76% pure by qNMR, 0.57 mmol) and chiral sulfonium salt **31b** (335 mg, 0.68 mmol, 1.2 eq) were placed into a flame-dried Schlenk tube and back-filled with nitrogen. Anhydrous MeCN (8 mL) was added and the brown solution was cooled to −20 °C (Cryostat). Flame-dried K_2CO_3 (156 mg, 1.13 mmol, 2.0 eq) was then added and the reaction mixture was stirred at −20 °C for 22 hr.
The reactants were quenched with water (30 mL) and the mixture was then extracted with DCM (4×20 mL). The combined organic layer was washed with brine (20 mL), dried over Na_2SO_4 and concentrated to give brown foam. The product was used crude without further purification. A 10-mg sample was used for qNMR analysis, and another 10-mg sample subjected to prep-HPLC and subsequent chiral SFC analysis.

Purification

The *trans*-aziridine ***trans*-32** is highly unstable and converts into *cis*-isomer ***cis*-32** in $CDCl_3$, MeCN/water, or on SiO_2/DCM. Analytically pure *cis*-aziridine was routinely prepared by ageing the crude *trans/cis*-mixtures in $CDCl_3$ (6–22 hr), and then purifying by preparative reverse-phase HPLC (80−95% MeCN-water over 25 min; t_R: *cis* 11.09 min, *trans* 11.83 min). Attempted separation of the *trans*-**32** by prep-HPLC led to the isolation of ~1:1 *trans/cis*-mixtures, presumably as the result of isomerization in the presence of a protic solvent (water).

Crude yield: 651 mg (35% pure by qNMR, 56% yield, *trans/cis* 9:1).

For characterization data, see below.

(±)-4-Bromo-3-methyl-5-(3-(4-(3-methylbut-2-en-1-yl)-1-tosyl-1*H*-indol-3-yl)-1-((4-nitrophenyl)sulfonyl)aziridin-2-yl)isoxazole, 32

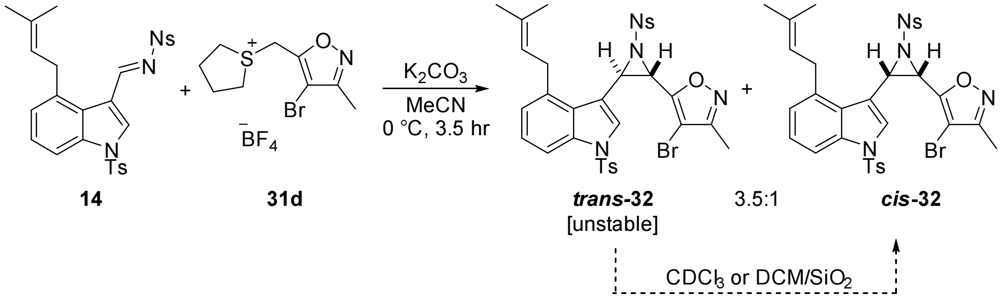

Imine **14** (0.71 g, 1.3 mmol), sulfonium salt **31d** (587 mg, 1.68 mmol, 1.3 eq) and flame-dried K_2CO_3 (356 mg, 2.58 mmol, 2.0 eq) were placed into a flame-dried Schlenk tube, and back-filled with nitrogen. The tube was places into a 0 °C bath. Anhydrous MeCN (12 mL) was then added and the resulting suspension was stirred at 0 °C for 3 hr, until LCMS indicated complete consumption of the imine.
Note: the product and the starting imine co-elute on TLC in EtOAc-petrol mixtures and thus the reaction is better monitored by reverse-phase LCMS (50−90% MeCN-water).
The reactants were quenched at 0 °C with 50 mL of water. The aqueous layer was then separated and extracted with DCM (4×15 mL). The combined organic layer was washed with brine (15 mL), dried over Na_2SO_4 and concentrated to give orange foam, which was immediately used in the next step without purification. A small portion (20 mg) was used for qNMR analysis.

Crude yield: 1.15 g (64% purity by qNMR, 79% yield, *trans/cis* 3.5:1).

Data for (+)-*cis*-***32***

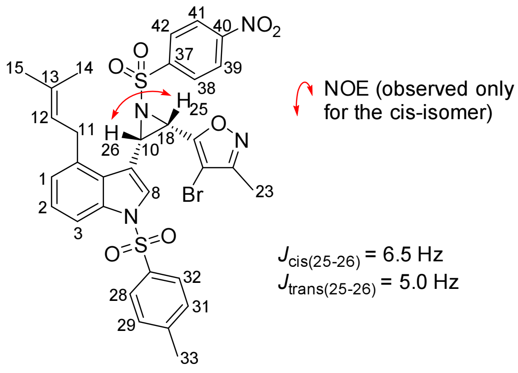

(+)-*cis*-32

White foam.

TLC (33% EtOAc-petrol): R_f = 0.50.

^{1}H NMR ($CDCl_3$, 500 MHz): δ = 8.47 (d, *J* = 8.6 Hz, 2H, H-39, -41), 8.32 (d, *J* = 8.7 Hz, 2H, H-38,42), 7.69 (d, *J* = 8.4 Hz, 1H, H-3), 7.63 (d, *J* = 8.1 Hz, 2H, H-28,32), 7.55 (s, 1H, H-8), 7.19 (d, *J* = 8.2 Hz, 2H, H-29,31), 7.16 (t, *J* = 8.0 Hz, 1H, H-2), 6.99 (d, *J* = 7.4 Hz, 1H, H-1), 5.27−5.18 (m, 1H, H-12), 4.73 (d, *J* = 6.5 Hz, 1H, H-26), 4.31 (d, *J* = 6.6 Hz, 1H, H-25), 3.74 (dd, *J* = 16.5, 7.5 Hz, 1H, H-11a), 3.57 (dd, *J* = 16.5, 5.5 Hz, 1H, H-11b), 2.33 (s, 3H, H-33), 2.11 (s, 3H, H-23), 1.80 (s, 3H, H-14), 1.79 (s, 3H, H-15).

^{13}C NMR ($CDCl_3$, 125 MHz): δ = 159.6 (C_q), 159.4 (C_q), 151.3 (C-40), 145.2 (C_q), 143.0 (C-37), 135.5 (C_q), 134.9 (C_q), 134.6 (C_q), 134.2 (C-13), 130.2 (C-29,31), 129.8 (C-38,42), 127.5 (C_q), 127.1 (C-28,32), 126.0 (C-8), 125.3 (C-2), 124.8 (C-39,41), 124.0 (C-1), 122.7 (C-12), 112.7 (C-9), 111.8 (C-3), 96.3 (C-19), 42.7 (C-10), 41.4 (C-16), 32.9 (C-11), 25.8 (C-15), 21.8 (C-33), 18.3 (C-14), 10.4 (C-23).

HRMS (ESI+), m/z: calcd for $C_{32}H_{29}BrN_4O_7S_2Na^+$ $[M+Na]^+$ 747.0553, found 747.0549.

IR (neat) ν_{max}: 3107 (w), 1533 (s), 1370 (m), 1348 (s), 1168 (s), 1142 (m), 1089 (s).

$[\alpha]_D^{21}$ = +40° (*c* = 1.0, $CHCl_3$).

Chiral SFC: Whelk-O1, 4 mL/min, 15% MeCN, 125 bar CO_2, 40 °C;
trans-**32**: t_R = 12.17 min (minor enantiomer), t_R = 15.32 min (major enantiomer), 98:2 *er*.
cis-**32**: t_R = 10.05 min (minor enantiomer), t_R = 13.26 min (major enantiomer), 89:11 *er*.

Racemic, trans/cis 60:40:

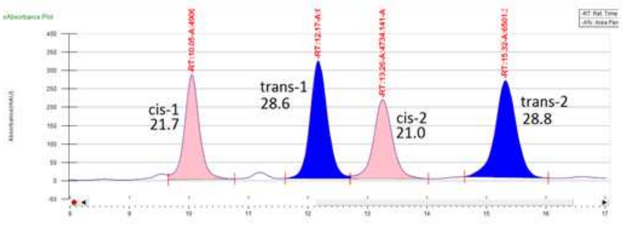

Racemic, cis:

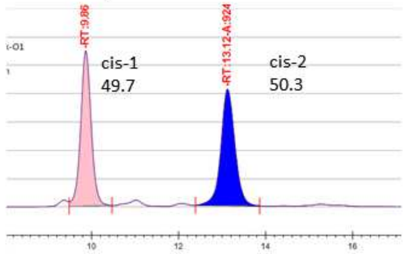

Enantioenriched, trans, e.r 98:2
(partially isomerized into cis on the column)

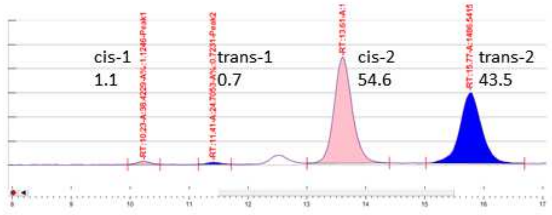

Enantioenriched, cis, e.r. 89:11

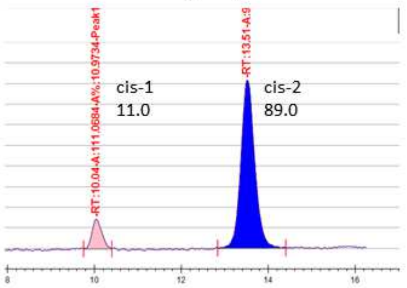

*Data for trans-**32***

Compound ***trans*-32** was too unstable to be isolated in pure form. Only selected data available.

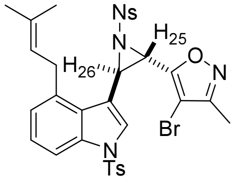

$J_{cis(25\text{-}26)}$ = 6.5 Hz
$J_{trans(25\text{-}26)}$ = 5.0 Hz

***trans*-32**

TLC (33% EtOAc-petrol): R_f = 0.50.

Selected ^{1}H NMR data ($CDCl_3$, 400 MHz): δ = 4.94 (dd, *J* = 5.0, 1.0 Hz, 1H, H_{26}), 4.57 (d, *J* = 5.0 Hz, 1H, H_{25}).

(+)-4-Bromo-5-((6a*R*,9*S*,9a*R*)-7,7-dimethyl-8-((4-nitrophenyl)sulfonyl)-2-tosyl-6,6a,7,8,9,9a-hexahydro-2*H*-isoindolo[4,5,6-*cd*]indol-9-yl)-3-methylisoxazole, (+)-33

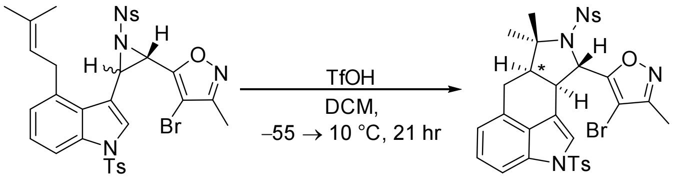

32
trans/cis 9:1
trans: er 98:2
cis: er 89:11

(+)-**33**
d.r. 3.5:1
er 98:2 (major d-r)

Freshly made crude aziridine **32** (630 mg, 35% pure by NMR, 0.30 mmol; *trans/cis* 9:1, er 98:2 *trans*, 89:1 *cis*) was dissolved in anhydrous DCM (5 mL) in a flame-dried Schlenk tube and cooled to −60 °C. Freshly made solution of TfOH in DCM (3 mL, 0.1 M, 0.3 mmol, 1 eq) was then added, and the resulting yellow solution was stirred between −55 and +10 °C over 21 hr. RP-LCMS monitoring (50 → 90% MeCN-water) showed very rapid consumption of the *trans*-aziridine (<10 min), while the *cis*-isomer reacted overnight.
The resulting crude mixture was diluted with half-sat. $NaHCO_3$ (30 mL) and extracted with DCM (4×10 mL). The combined organic layer was washed with brine (10 mL), dried over Na_2SO_4 and concentrated to give brown foam (595 mg, 27% purity by NMR, 73% NMR yield).
The bulk sample was purified by chromatography on Biotage Isolera system (SNAP Ultra 25 g, 50 → 100% DCM-petrol, 1-5-15 CV) to give the desired product as a 3:1 mixture of diastereomers. A 30-mg aliquot was subjected to prep-HPLC separation (ACE 5 C18, 80→95% MeCN-water over 25 min) to obtain an analytically pure sample of (+)-**33** for chiral SFC analysis, *er* and optical rotation measurements.

Yield: 108 mg (50%), d.r. 3.5:1

(±)-4-Bromo-5-((6a*R*,9*S*,9a*R*)-7,7-dimethyl-8-((4-nitrophenyl)sulfonyl)-2-tosyl-6,6a,7,8,9,9a-hexahydro-2*H*-isoindolo[4,5,6-*cd*]indol-9-yl)-3-methylisoxazole, (±)-33

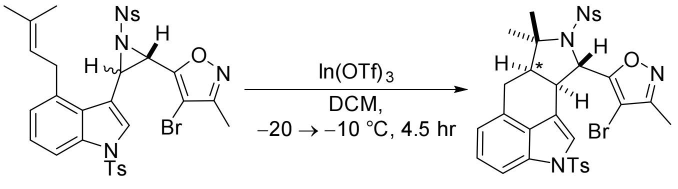

rac-**32**
trans/cis 3.3:1

rac-**33**
d.r. 3:1

Freshly made crude aziridine *rac*-**32** (*trans/cis* 3.3:1, 830 mg, 60% pure by NMR, 0.69 mmol) was dissolved in anhydrous DCM (20 mL) in a flame-dried Schlenk tube and cooled to −20 °C. $In(OTf)_3$ (771 mg, 1.37 mmol, 2.0 eq) was then quickly added in one portion, and the resulting yellowish suspension was stirred at −10 °C (Cryostat) for 4.5 hr. RP-LCMS monitoring (50 → 90% MeCN-water) showed very rapid consumption of the *trans*-aziridine (<10 min), while the *cis*-isomer generally took 2−4 hr to react.
The reactants were quenched with 1 mL of half-sat. $NaHCO_3$ at −10 °C, and the mixture was then partitioned between half-sat. $NaHCO_3$ (60 mL) and DCM (10 mL). The aqueous layer was separated and further extracted with DCM (3×15 mL). The combined organic layer was washed with brine (20 mL), dried over Na_2SO_4 and concentrated. Purification on Biotage Isolera system (ZIP KP-Sil 10 g, 50 → 100% DCM-*n*-pentane, 1-5-15 CV) provided the target material as a mixture of two diastereomers (d.r. 3:1) as pale-yellow foam.
Attempted crystallization from MeCN gave the product as white powder but did not separate the diastereomers. Thus, analytically pure samples of individual diastereomers were obtained by subsequent prep-HPLC purification (ACE 5 C18, 80 → 95% MeCN-water over 25 min). Crystals of *rac*-**33** suitable for single-crystal X-Ray crystallography were grown from dilute (~2-3 mg/mL) solutions in MeCN-MeOH (7:3) by ageing for 3 days at 23 °C.

Yield: 308 mg (62%), d.r. 3:1.

*Data for the major (desired) isomer, (+)-**33***

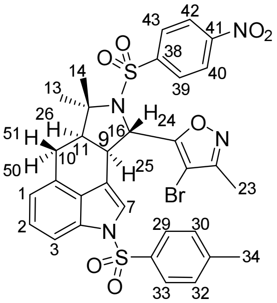

(+)-**33**
98:2 *er*

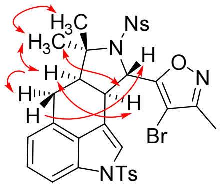

Diagnostic NOE

White powder (racemic mixture, from MeCN).
White needles ((+)-enantiomer, from MeOH).

Mp (racemic mixture): 248−249 °C (MeCN), dec. after melting.

Mp ((+)-enantiomer): 219−221 °C (MeOH), dec.

TLC: R_f = 0.43 (DCM), 0.48 (30% EtOAc-petrol).

^{1}H NMR ($CDCl_3$, 500 MHz): δ = 8.23 (d, *J* = 8.8 Hz, 2H, H-40,42), 7.86 (d, *J* = 8.8 Hz, 2H, H-39,43), 7.73 (d, *J* = 8.4 Hz, 2H, H-29,33), 7.70 (d, *J* = 8.3 Hz, 1H, H-3/1), 7.30−7.27 (m, 1H, H-2), 7.22 (d, *J* = 8.2 Hz, 2H, H-30,32), 7.15 (d, *J* = 1.4 Hz, 1H, H-7), 7.01 (d, *J* = 7.3 Hz, 1H, H-1/3), 5.10 (d, *J* = 4.9 Hz, 1H, H-24), 3.67 (m, 1H, H-25), 3.10 (dd, *J* = 17.1, 5.9 Hz, 1H, H-50), 2.84 (q, *J* = 6.0 Hz, 1H, H-26), 2.72 (dd, *J* = 17.1, 5.8 Hz, 1H, H-51), 2.34 (s, 3H, H-34), 2.25 (s, 3H, H-23), 1.74 (s, 3H, H-13), 1.04 (s, 3H, H-14).

^{13}C NMR ($CDCl_3$, 125 MHz): δ = 166.6 (C-17), 159.6 (C-19), 150.0 (C-41), 146.3 (C-38), 145.2 (C-31), 135.1 (C-28), 133.5 (C-4), 130.1 (C-30,32), 129.3 (C_q), 128.7 (C-39,43), 128.6 (C_q), 126.9 (C-29,33), 126.4 (C_q), 124.1 (C-40,42), 121.1 (C-3-or-1), 121.0 (C-7), 117.2 (C-8), 111.8 (C-1-or-3), 94.4 (C-18), 70.8 (C-12), 59.5 (C-16), 48.0 (C-11), 39.5 (C-9), 29.9 (C-13), 25.6 (C-10), 24.1 (C-14), 21.7 (C-34), 10.6 (C-23).

HRMS (TOF ESI$^+$), m/z: calcd for $C_{32}H_{29}BrN_4O_7S_2Na^+$ $[M+Na]^+$ 747.0553, found 747.0556.

IR (neat) ν_{max}: 3105 (w), 1606 (w), 1531 (s), 1350 (s), 1178 (s), 1164 (s), 1092 (s), 737 (s).

$[\alpha]_D^{21}$ = +10° (*c* = 0.4, $CHCl_3$).

Chiral SFC: Whelk-O1, 4 mL/min, 20% MeCN, 125 bar CO_2, 40 °C, t_R = 8.05 min (minor enantiomer), t_R = 11.03 min (major enantiomer), 98:2 *er*.

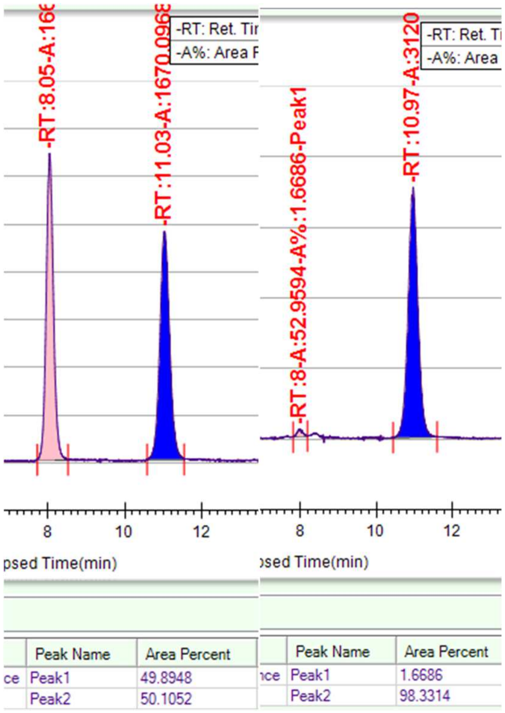

Table 9. Crystal data and structure refinement for compound (±)-**33**.

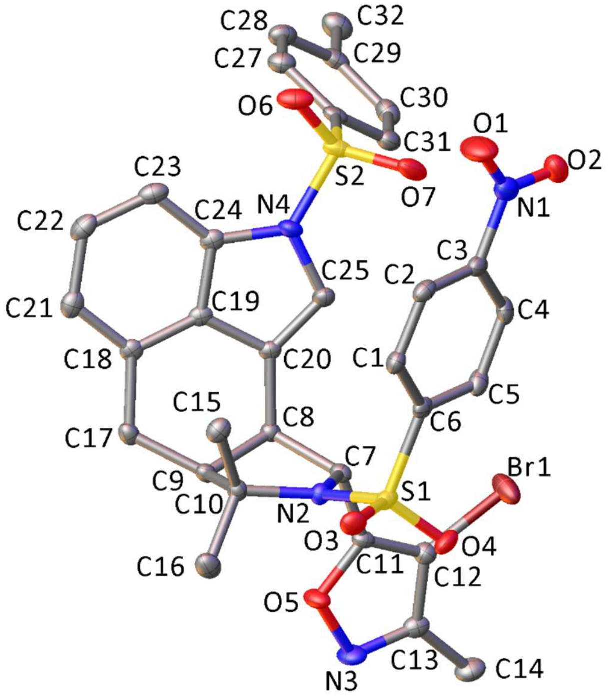

Illustration of the structure of (±)-**33** with atomic numbering scheme depicted. Only one of the two unique molecules in the asymmetric unit is shown for clarity, the relative orientations of all groups are the same in both molecules. Thermal ellipsoids depicted at the 50% probability level and hydrogen atoms omitted for clarity.

Identification code	**33**
CCDC number	**1584105**
Empirical formula	$C_{32}H_{29}BrN_4O_7S_2$
Formula weight	725.62
Temperature/K	100(2)
Crystal system	triclinic
Space group	*P*-1
a/Å	13.4320(2)
b/Å	14.3213(2)
c/Å	18.2173(3)
α/°	108.0650(10)
β/°	97.8160(10)
γ/°	103.4350(10)
Volume/Å^3	3157.35(9)
Z	4
ρ_{calc}g/cm^3	1.526
μ/mm^{-1}	1.489
F(000)	1488.0
Crystal size/mm^3	0.496 × 0.294 × 0.272
Radiation	MoKα (λ = 0.71073)
2θ range for data collection/°	3.128 to 54.382
Index ranges	-17 ≤ h ≤ 17, -18 ≤ k ≤ 18, -23 ≤ l ≤ 23
Reflections collected	54018
R_{int} / R_{sigma}	0.0499 / 0.0491
Data/restraints/parameters	14009/0/837
Goodness-of-fit on F^2	1.031
Final R indexes [I>=2σ (I)]	R_1 = 0.0355, wR_2 = 0.0725
Final R indexes [all data]	R_1 = 0.0587, wR_2 = 0.0792
Largest diff. peak/hole / e Å^{-3}	0.37/-0.54

Note: Squeeze applied to remove disordered methanol, 1 per void, that could not be sensibly modelled.

(±)-4-Bromo-5-((6a*S*,9*S*,9a*R*)-7,7-dimethyl-8-((4-nitrophenyl)sulfonyl)-2-tosyl-6,6a,7,8,9,9a-hexahydro-2*H*-isoindolo[4,5,6-*cd*]indol-9-yl)-3-methylisoxazole, iso-33

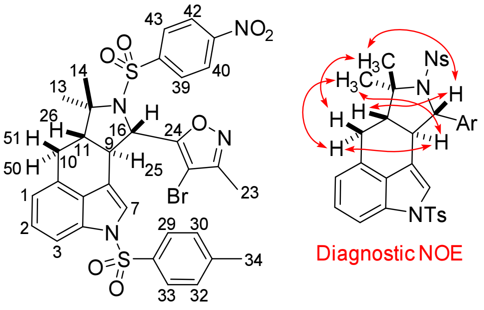

iso-33

Isolated as a minor diastereomer in the synthesis of compound **33**.

White amorphous solid (racemic mixture).

TLC (DCM): R_f = 0.43; (30% EtOAc-petrol): R_f = 0.48.

^{1}**H NMR** ($CDCl_3$, 500 MHz): δ = 8.28 (d, *J* = 8.8 Hz, 2H, H-40,42), 7.95 (d, *J* = 8.8 Hz, 2H, H-39,43), 7.77−7.70 (m, 3H, H-Ar,29,33), 7.28−7.22 (m, 3H, H-Ar,30,32), 7.04−6.99 (m, 2H, 2×H-Ar), 4.94 (d, *J* = 10.0 Hz, 1H, H-24), 3.66 (ddd, *J* = 12.5, 10.0, 1.9 Hz, 1H, H-25), 2.90 (dd, *J* = 15.5, 4.0 Hz, 1H, H-51), 2.82 (dd, *J* = 15.5, 12.5 Hz, 1H, H-

50), 2.36 (s, 3H, H-34), 2.35 (s, 3H, H-23), 2.07 (td, J = 12.5, 4.0 Hz, 1H, H-26), 1.65 (s, 3H, H-14), 1.58 (s, 3H, H-13).

^{13}C NMR ($CDCl_3$, 125 MHz): δ = 165.6 (C_q), 160.0 (C_q), 150.0 (C_q), 146.9 (C_q), 145.3 (C_q), 135.5 (C_q), 133.6 (C_q), 130.6 (C_q), 130.1 (C-30,32), 129.3 (C_q), 128.7 (C-39,43), 127.0 (C-29,33), 126.2 (CH), 124.2 (C-40,42), 121.6 (CH), 118.8 (CH), 117.9 (C_q), 112.0 (CH), 95.1 (C-18), 69.2 (C-12), 58.4 (C-16), 54.6 (C-11), 42.4 (C-9), 27.9 (C-10), 27.2 (C-14), 24.7 (C-13), 21.8 (C-34), 10.8 (C-23).

HRMS (MALDI$^+$), m/z: calcd for $C_{32}H_{29}O_7N_4S_2BrNa^+$ [M+Na]$^+$ 747.0553, found 747.0559.

IR (neat) ν_{max}: 3107 (w), 1602 (w), 1529 (s), 1350 (s), 1177 (s), 1161 (s), 1118 (s), 1089 (s), 1071 (s), 731 (s).

(–)-4-Bromo-5-((6a*R*,9*S*,9a*R*)-7,7-dimethyl-2-tosyl-6,6a,7,8,9,9a-hexahydro-2*H*-isoindolo[4,5,6-*cd*]indol-9-yl)-3-methylisoxazole, (–)-34

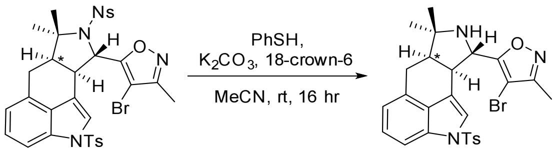

(+)-33 d.r. 3.5:1 **(–)-34** d.r. 3.5:1

[Both racemic and enantioenriched material were prepared using the following protocol]
Starting sulfonamide (+)-**33** (98 mg, 0.135 mmol, d.r. 3.5:1), 18-crown-6 (108 mg, 0.41 mmol, 3 eq), flame-dried K_2CO_3 (52 mg, 0.41 mmol, 3 eq), and naphthaline (~3 mg, internal LCMS standard) were placed into a flame dried Schlenk tube and back-filled with nitrogen. Degassed MeCN (1 mL) was added, followed by PhSH (0.03 mL, 0.3 mmol, 2 eq). The resulting brown suspension was stirred at r.t. for 3.5 hr, when LCMS monitoring indicated complete conversion.
The reaction mixture was diluted with water (10 mL) and extracted with DCM (4×3 mL). The combined organic layer was washed with brine (4 mL), dried over Na_2SO_4 and concentrated to give brown oil. Purification by silica chromatography (0.6×6 cm, 10→33→75% EA-*n*-pentane) provided the product as a beige foam (50 mg, 68%, d.r. 3.5:1).
Individual diastereomers were then isolated using reverse-phase preparative HPLC (ACE 5 C18, 250×21.5 mm, 60 → 95% MeCN-water over 20 min).

Yield: 131 mg (63%, d.r. 3.5:1).

*Data for the major (desired) diastereomer, **34***

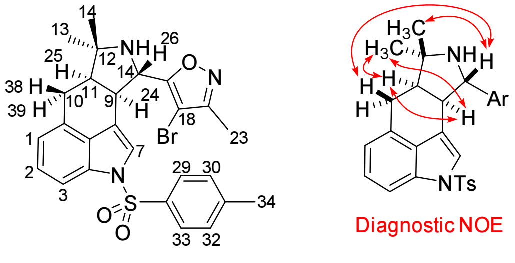

(–)-34

White powder (racemic mixture).
Colorless oil ((–)-enantiomer)

Mp (racemic mixture): 173−174 °C (MeCN).

TLC (50% EtOAc-*n*-pentane): R_f = 0.40.

Prep-HPLC (ACE 5 C18, 250×21.5 mm, 60 → 95% MeCN-water/20 min): t_R 12.40 min.

^{1}H NMR (C_6D_6, 500 MHz): δ = 8.08 (d, *J* = 8.0 Hz, 1H, H-1/3), 7.71 (d, *J* = 8.5 Hz, 2H, H-29,33), 7.37 (d, *J* = 1.0 Hz, 1H, H-7), 7.21 (t, *J* = 8.0 Hz, 1H, H-2), 6.85 (d, *J* = 8.0 Hz, 1H, H-3/1), 6.52 (d, *J* = 8.5 Hz, 2H, H-30,32), 4.28 (d, *J* = 8.5 Hz, 1H, H-26), 3.79 (dd, *J* = 8.5, 7.0 Hz, 1H, H-24), 2.48 (dd, *J* = 16.5, 6.0 Hz, 1H, H-39), 2.34 (dd, *J* = 16.5, 9.0 Hz, 1H, H-38), 1.96 (s, 3H, H-23), 1.82 (ddd, *J* = 9.0, 7.0, 6.0 Hz, 1H, H-25), 1.63 (s, 3H, H-34), 1.16 (s, 3H, H-13), 0.69 (s, 3H, H-14).

^{13}C NMR (C_6D_6, 125 MHz): δ = 170.0 (C-17), 159.7 (C-19), 144.4 (C-31), 136.4 (C_q), 134.0 (C_q), 131.0 (C_q), 129.8 (C-30,32), 129.0 (C_q), 127.0 (C-29,33), 126.0 (C-2), 121.4 (C-7/1), 121.0 (C-1/7), 120.0 (C_q), 111.9 (C-3), 92.7 (C-18), 61.8 (C-12), 59.2 (C-16), 48.2 (C-11), 41.0 (C-9), 31.2 (C-13), 25.9 (C-10), 24.5 (C-14), 21.0 (C-34), 10.3 (C-23).

IR (neat) ν_{max}: 1598 (w), 1436 (w), 1364 (m), 1176 (s).

HRMS (ESI$^+$), m/z: calcd for $C_{26}H_{27}{}^{79}BrN_3O_3S^+$ [M+H]$^+$ 540.0951, found 540.0939.

$[\alpha]_D^{21}$ = −23° (*c* = 1.0, $CHCl_3$).

*Data for the minor (undesired) diastereomer, (−)-iso-**34***

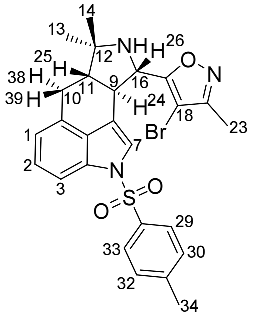

(−)-iso-34

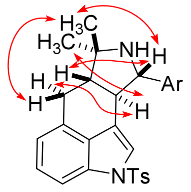

Diagnostic NOE

White powder (racemic mixture).
Colorless oil ((−)-enantiomer).

Mp (racemic mixture): 197−199 °C (dec; MeCN).

TLC (50% EtOAc-*n*-pentane): R_f = 0.40.

Prep-HPLC: ACE 5 C18, 250×21.5 mm, 60 → 95% MeCN-water/20 min, t_R = 13.50 min.

^{1}H NMR (C_6D_6, 500 MHz): δ = 8.10 (d, *J* = 8.2 Hz, 1H, H-1/3), 7.75 (d, *J* = 8.4 Hz, 2H, H-29,33), 7.48 (d, *J* = 1.9 Hz, 1H, H-7), 7.20 (dd, *J* = 8.0, 7.5 Hz, 1H, H-2), 6.83 (d, *J* = 7.3 Hz, 1H, H-1/3), 6.47 (d, *J* = 8.1 Hz, 2H, H-30,32), 4.29 (d, *J* = 10.0 Hz, 1H, H-26), 3.14 (ddd, *J* = 12.3, 10.0, 1.9 Hz, 1H, H-24), 2.37 (dd, *J* = 15.5, 3.8 Hz, 1H, H-39), 2.20 (dd, *J* = 15.5, 12.5 Hz, 1H, H-38), 1.93 (s, 3H, H-23), 1.58 (s, 3H, H-34), 1.43 (td, *J* = 12.5, 3.7 Hz, 1H, H-25), 0.97 (s, 3H, H-13), 0.88 (s, 3H, H-14), 0.48 (br s, 1H, NH).

^{13}C NMR (C_6D_6, 125 MHz): δ = 169.7 (C_q), 159.7 (C_q), 144.4 (C_q), 136.4 (C_q), 134.4 (C_q), 133.0 (C_q), 130.9 (C_q), 129.8 (C-30,32), 127.0 (C-29,33), 125.9 (C-2), 121.6 ($C_{q/1/3}$), 121.5 ($C_{q/1/3}$), 118.9 (C-7), 112.1 (C-1/3), 92.7 (C-18), 58.4 (C-12), 55.8 (C-16), 55.0 (C-11), 44.4 (C-9), 29.1 (C-14), 28.3 (C-10), 25.9 (C-13), 20.9 (C-34), 10.3 (C-23).

HRMS (TOF ESI$^+$), m/z: calcd for $C_{26}H_{27}Br^{79}N_3O_3S^+$ [M+H$^+$] 540.0951, found 540.0958.

IR (neat) ν_{max}: 3350 (w), 1598 (w), 1434 (m), 1368 (m), 1176 (s), 1115 (s), 1092 (s).

$[\alpha]_D^{21}$ = −8° (*c* = 0.48, $CHCl_3$).

(3-Methylisoxazol-5-yl)methanol, 35[25,26]

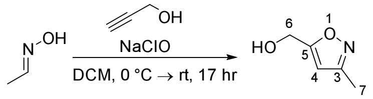

35

A solution of acetaldehyde oxime (mixture of *syn* and *anti*-isomers, 1.00 g, 16.9 mmol) and propargyl alcohol (2.0 mL, 34 mmol, 2.0 eq) in 60 mL of DCM was cooled to 0 °C and stirred for 10 min. Aqueous NaOCl (14.8 wt%, 14 mL, 34 mmol, 2.0 eq) was then added dropwise over 20 min. The reaction was allowed to warm up to 23 °C gradually and stirred overnight (17 hr) under nitrogen. It was then diluted with 10 mL of water, and the two phases were partitioned. The aqueous layer was extracted with DCM (2×20 mL), and the combined organic layer was dried over $MgSO_4$, filtered and concentrated. Purification by flash column chromatography (33→50% EtOAc-hexane) afforded alcohol **35** as a volatile light-yellow oil.

Yield: 1.24 g (65%).
Light-yellow oil.

TLC (33% EtOAc-*n*-pentane): R_f = 0.13 (UV inactive, stains with $KMnO_4$).

^{1}H NMR ($CDCl_3$, 400 MHz): δ = 6.05 (s, 1H, CH), 4.66 (s, 2H, CH_2), 3.57 (br s, 1H, OH), 2.24 (s, 3H, CH_3).

^{13}C NMR ($CDCl_3$, 100 MHz): δ = 171.6, 160.0, 102.6, 56.2, 11.4.

IR (neat) ν_{max}: 3346 (br. s), 2933 (w), 1609 (s), 1417 (s), 1133 (m), 1026 (s), 997 (s).

5-(Bromomethyl)-3-methylisoxazole, 37[27]

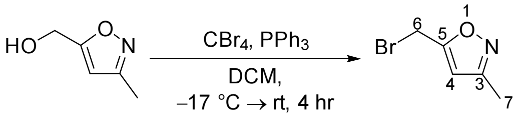

35 37

A solution of alcohol **35** (3.36 g, 29.7 mmol) in 60 mL of anhydrous DCM was cooled to ca. −17 °C in a MeOH-ice bath, and stirred for 10 min under nitrogen. PPh_3 (9.35 g, 35.6 mmol, 1.20 eq) and CBr_4 (11.8 g, 35.6 mmol, 1.20 eq) were then added sequentially. The resulting dark orange mixture was stirred at this temperature for 1 hr, and then at 23 °C for 3 hr, at which time TLC analysis indicated full consumption of alcohol **35**. All volatiles were removed *in vacuo*. Purification by flash column chromatography (14 → 20% EtOAc-*n*-pentane) afforded **37** (4.48 g, 86%) as a pale yellow oil.

Yield: 4.48 g (86%).
Pale yellow oil.

TLC (20% EtOAc-*n*-pentane): R_f = 0.33.

^{1}H NMR ($CDCl_3$, 400 MHz): δ = 6.15 (s, 1H, CH), 4.42 (s, 1H, CH_2), 2.29 (s, 1H, CH_3).

^{13}C NMR ($CDCl_3$, 100 MHz): δ = 167.3, 160.3, 104.6, 18.8, 11.6.

HRMS (CI^+), m/z: calcd for C_5H_7NOBr $[M+H]^+$ 175.9711, found 175.9718.

IR (neat) ν_{max}: 1607, 1444, 1419, 1215, 1003, 911, 807, 741.

4-Bromo-5-(bromomethyl)-3-methylisoxazole, 38

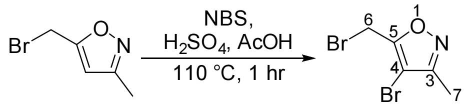

37 **38**

The starting isoxazole **37** (1.00 g, 5.68 mmol) was dissolved in reagent grade acetic acid (50 mL), followed by *N*-bromosuccinimide (NBS, 1.21 g, 6.8 mmol) and sulfuric acid (95%, 0.61 mL, 11 mmol), in a 100 mL recovery flask, to give a clear colorless solution. A reflux condenser was attached, the setup was briefly back filled with nitrogen (5 times) and the reaction mixture was heated at 110 °C for 1 hr.
After cooling, the mixture was diluted with 10 mL of Et_2O and stripped of volatiles at 30–33 °C. The residue was diluted with DCM (10 mL) and remaining reactants were quenched with 40 mL of sat. Na_2CO_3 (caution: intense gas evolution). The layers were separated and the aqueous layer was extracted with 3×10 mL of DCM. The combined organic layer was washed with brine (20 mL), dried over Na_2SO_4 and concentrated to give cloudy pale-brown oil. The crude mixture was purified by silica chromatography on a Biotage Isolera system (SNAP KP-Sil 25 g, 2 → 30% EtOAc-petrol, 1–10–2 CV) to give a colorless oil, which solidified (white, waxy solid) upon standing in freezer (ca. −20 °C).

Yield: 1.14 g (79%).
Colorless oil → white waxy solid.

TLC (10% Et_2O-*n*-pentane): R_f = 0.37.

^{1}H NMR ($CDCl_3$, 500 MHz): δ = 4.44 (s, 2H, H_6), 2.29 (s, 3H, H_7).

^{13}C NMR ($CDCl_3$, 125 MHz): δ = 164.1, 160.1, 95.3, 17.1, 10.7.

HRMS (EI), m/z: calcd for $C_5H_5NOBr_2$ $[M]^+$ 252.8738, found 252.8743.

IR (neat) ν_{max}: 2983 (w), 1607 (m), 1423 (m), 1402 (m), 1275 (m), 1211 (m), 1132 (m), 1063 (s), 914 (m).

Ethyl 2-methyl-4-oxo-4,5-dihydrofuran-3-carboxylate, 42[21]

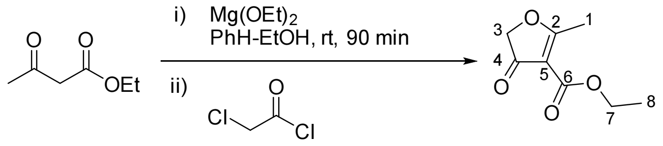

20 MeCN-PhH, 0 °C to rt, 3 h **42**

The known furanone **42** was prepared following a literature procedure.[21] Magnesium ethoxide (9.36 g, 82 mmol, 1.1 eq) was placed into a flame-dried 100 mL flask, followed by anhydrous ethanol (1.7 mL), anhydrous benzene (18 mL), and reagent-grade ethyl acetoacetate **20** (10.0 g, 74.7 mmol, 1.0 eq). The mixture was stirred at 23 °C for 90 min, then cooled to 0 °C. Anhydrous MeCN (18 mL) was added, followed by chloroacetyl chloride (6.5 mL, 82 mmol, 1.1 eq) with vigorous stirring (~600 rpm). The reaction mixture was manually swirled for 30 sec, then allowed to warm up to 23 °C over 3 hr with magnetic stirring.
The mixture was poured into 50 mL of ice water containing 3 mL of conc. H_2SO_4. The layers were separated and the aqueous layer was extracted with 3×20 mL Et_2O. The organic layer was washed with brine (20 mL), dried over $MgSO_4$ and filtered. The resulting solution was cooled to 0 °C. TEA (10.3 mL) was added to the rapidly stirred solution, to precipitate acidic side-products. The stirring was continued at 0 °C for 30 min. The solids were then filtered off, washing with Et_2O. The clear solution was concentrated to give brown oil.
The product was purified on Biotage Isolera system (3 portions, ZIP KP-Sil 120 g, loaded neat, 20 → 100% Et_2O-petrol), collecting the second major peak. The resulting yellow solid was used in the next step. A small, analytically pure, sample was prepared by crystallization from hot Et_2O.

Yield: 5.63 g (44%).
White needles.

Mp: 75–76 °C (Et_2O; lit.[21] mp 76 °C).

TLC (10% EtOAc-petrol): R_f = 0.33.

^{1}H NMR (400 MHz, $CDCl_3$,): δ = 4.59 (s, 2H, H-3), 4.31 (q, *J* = 7.1 Hz, 2H, H-7), 2.61 (s, 3H, H-1), 1.34 (t, *J* = 7.1 Hz, 3H, H-8).

^{13}C NMR (100 MHz, $CDCl_3$): δ = 198.3 (C-2), 195.9 (C-4), 162.4 (C-6), 109.8 (C-5), 75.7 (C-3), 60.6 (C-7), 17.9 (C-1), 14.4 (C-8).

IR (neat) ν_{max}: 3445 (br. w), 1702 (s), 1586 (s), 1153 (s).

Ethyl 5-(2-hydroxy-2-(4-(3-methylbut-2-en-1-yl)-1-tosyl-1*H*-indol-3-yl)-1-((4-nitrophenyl)sulfonamido)ethyl)-3-methylisoxazole-4-carboxylate, 43

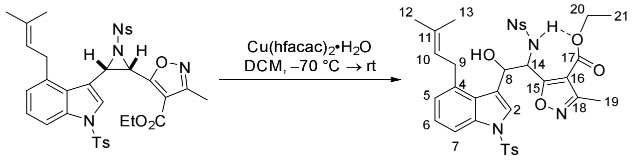

24 **43**

Common side-product in the (3+2)-cycloaddition optimization studies to prepare ***25***.

The starting aziridine (15 mg, 0.021 mmol) and naphthalene (6.7 mg, internal standard) were placed into a flame-dried Schlenk tube, followed by DCM (1 mL). The clear colorless solution was cooled to −70 °C, and $Cu(hfacac)_2 \cdot H_2O$ (10 mg, 0.021 mmol, 1.0 eq) was then added. The reaction was allowed to warm to room temperature over 6 hr, while being monitored by HPLC with the internal standard.

When all the starting material has been consumed, the reactants were quenched with 3 mL of 1:1 sat. NH_4Cl – sat. $NaHCO_3$. The products were then extracted with DCM (3×2 mL). The combined organic extracts were washed with 1:1 sat. aq. NH_4Cl – sat. aq. $NaHCO_3$ (2×2 mL), brine (2 mL), then dried over Na_2SO_4 and concentrated to give clear yellow oil. Purification by prep-HPLC (ACE C18, 21×250mm, 80 → 95% MeCN–water over 20 min, t_R = 7.33 min) gave the amino alcohol **43** as a white film.

Yield: 2.2 mg (15%).
White film.

TLC (33% EtOAc-petrol): R_f = 0.34.

^{1}H NMR (500 MHz, $CDCl_3$): δ = 8.01–7.96 (m, 2H, H-Ns), 7.78–7.74 (m, 2H, H-Ts), 7.73 (d, *J* = 0.9 Hz, 1H, H-2), 7.72–7.68 (m, 2H, H-Ns), 7.66 (dd, *J* = 8.3, 1.0 Hz, 1H, H-5/7), 7.23 (d, *J* = 8.6 Hz, 2H, H-Ts), 7.14 (dd, *J* = 8.3, 7.4 Hz, 1H, H-6), 6.96 (dd, *J* = 7.4, 1.0 Hz, 1H, H-5/7), 6.59 (d, *J* = 7.2 Hz, 1H, NH), 5.46 (td, *J* = 3.8, 1.0 Hz, 1H, H-8), 5.35 (dd, *J* = 8.9, 3.8 Hz, 1H, H-14), 5.12–5.06 (m, 1H, H-10), 4.46 – 4.34 (m, 2H, H-20), 3.56 (dd, *J* = 16.5, 7.0 Hz, 1H, H-9a), 3.48 (dd, *J* = 16.5, 6.6 Hz, 1H, H-9b), 2.95 (d, *J* = 3.8 Hz, 1H, OH), 2.36 (s, 3H, H-19), 2.34 (s, 3H, H-TsCH$_3$), 1.69 (d, *J* = 1.4 Hz, 3H, H-12/13), 1.63 (d, *J* = 1.3 Hz, 3H, H-12/13), 1.41 (t, *J* = 7.1 Hz, 3H, H-21).

^{13}C NMR (125 MHz, $CDCl_3$): δ = 174.1 (C-15), 162.7 (C-17), 159.7 (C_q), 149.8 (C_q), 145.4 (C_q), 144.4 (C_q), 135.3 (C_q), 135.0 (C_q), 134.6 (C_q), 134.0 (C-11), 130.2 (C-Ts), 127.9 (C-Ns), 127.3 (C-Ts), 125.9 (C_q), 125.3 (C-2/6), 125.2 (C-2/6), 124.3 (C-5/7), 124.1 (C-Ns), 122.3 (C-10), 119.9 (C_q), 111.5 (C-5/7), 110.7 (C_q), 68.8 (C-8), 62.2 (C-20), 54.5 (C-14), 32.3 (C-9), 25.7 (C-12/13), 21.8 (C-TsCH$_3$), 18.1 (C-12/13), 14.2 (C-21), 12.1 (C-19).

HRMS (TOF ESI$^+$), m/z: calcd for $C_{35}H_{36}N_4O_{10}S_2Na^+$ $[M+Na]^+$ 759.1765, found 759.1757.

IR (neat) ν_{max}: 3525 (br. w), 3292 (br.w), 1717 (m), 1531 (s), 1349 (s), 1171 (s), 1091 (s).

Ethyl 5-((6a*R*,9*S*,9a*R*)-7,7-dimethyl-2-tosyl-6,6a,7,8,9,9a-hexahydro-2*H*-isoindolo[4,5,6-cd]indol-9-yl)-3-methylisoxazole-4-carboxylate, 44

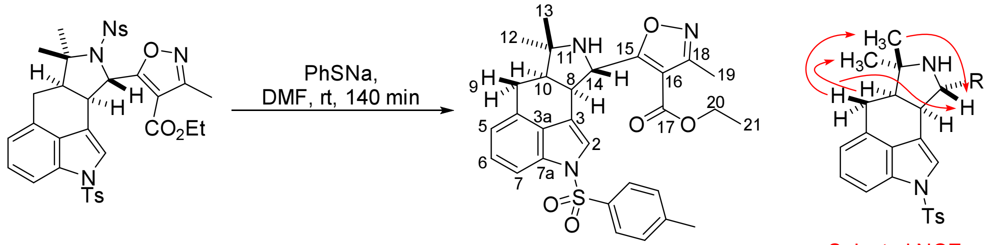

Selected NOE

25 **44**

The starting sulfonamide **25** (45 mg, 0.063 mmol, 1.0 eq) was dissolved in anhydrous degassed DMF (1 mL), followed by PhSNa (25 mg, 0.188 mmol, 3.0 eq). The resulting clear dark-red solution was stirred at 23 °C for 140 min, when LCMS (50→90% MeCN-water) indicated complete consumption of the starting material.
The mixture was diluted with half-sat. aq. NaCl (5 mL) and extracted with DCM (4×2 mL). The combined organic layer was washed with brine (2 mL), dried over Na_2SO_4 and concentrated to give brown oil. The desired compound **44** was isolated by silica chromatography (25 → 50% EtOAc-petrol), along with some side-product **45**.

Yield: 24 mg (73%).
Clear brownish oil.

TLC (66% EtOAc-petrol): R_f = 0.20.

^{1}H NMR (500 MHz, $CDCl_3$): δ = 7.75–7.70 (m, 3H, H-7,Ts), 7.26 (*app* t, *J* = 7.5 Hz, 2H, H-6, NH), 7.20 (d, *J* = 7.9 Hz, 2H, H-Ts), 7.16 (d, *J* = 1.0 Hz, 1H, H-2), 7.04 (d, *J* = 7.3 Hz, 1H, H-5), 4.94 (d, *J* = 9.1 Hz, 1H, H-14), 4.23–4.09 (m, 3H, H-8, -20), 3.03 (dd, *J* = 16.6, 6.1 Hz, 1H, H-9a), 2.86 (dd, *J* = 16.6, 9.2 Hz, 1H, H-9b), 2.49 (s, 3H, H-19), 2.44 (dt, *J* = 9.3, 6.3 Hz, 1H, H-10), 2.33 (s, 3H, H-TsCH$_3$), 1.39 (s, 3H, H-12), 1.15 (t, *J* = 7.1 Hz, 3H, H-21), 1.10 (s, 3H, H-13).

^{13}C NMR (125 MHz, $CDCl_3$): δ = 178.0 (C-15), 162.2 (C-17), 160.3 (C-18), 144.8 (C-Ar), 135.6 (C-Ar), 133.3 (C-Ar), 130.5 (C-Ar), 130.0 (C-Ts), 128.6 (C-Ar), 126.9 (C-Ts), 125.8 (C-6), 120.9 (C-2, -5), 119.4 (C-Ar), 111.4 (C-7), 109.9 (C-Ar), 62.1 (C-11), 60.9 (C-20), 58.8 (C-14), 48.4 (C-10), 39.7 (C-8), 31.6 (C-12), 25.9 (C-9), 24.8 (C-13), 21.7 (C-TsCH$_3$), 14.1 (C-21), 12.1 (C-19).

HRMS (TOF ESI$^+$), m/z: calcd for $C_{29}H_{32}N_3O_5S$ [M+H]$^+$ 534.2057, found 534.2050.

IR (neat) ν_{max}: 3400 (w), 1717 (s), 1600 (m), 1436 (m), 1366 (s), 1296 (s), 1177 (s), 1102 (s).

Ethyl 5-((6a*R*,9*S*,9a*R*)-7,7-dimethyl-8-((4-(phenylthio)phenyl)sulfonyl)-2-tosyl-6,6a,7,8,9,9a-hexahydro-2*H*-isoindolo[4,5,6-cd]indol-9-yl)-3-methylisoxazole-4-carboxylate, 45

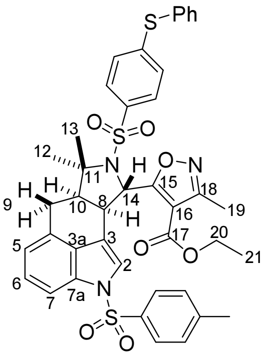

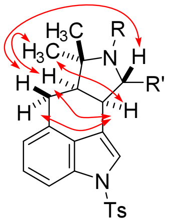

Selected NOE

45

Isolated as a major side product in the synthesis of amine **44**.

Yield: 7 mg (14%).
Brown oil.

TLC (60% EtOAc-petrol): R_f = 0.59.

^{1}H NMR (500 MHz, $CDCl_3$): δ = 7.73−7.69 (m, 2H, H-Ts), 7.64 (d, J = 8.2 Hz, 1H, H-7), 7.56−7.52 (m, 2H, H-Ns), 7.52−7.49 (m, H-SPh), 7.46−7.38 (m, 3H, H-SPh), 7.33 (d, *J* = 1.7 Hz, 1H, H-2), 7.25−7.18 (m, 3H, H-6,Ts), 7.09−7.05 (m, 2H, H-Ns), 6.98 (d, *J* = 7.2 Hz, 1H, H-5), 5.96 (d, *J* = 3.1 Hz, 1H, H-14), 4.31 (qd, *J* = 7.1, 1.4 Hz, 2H, H-20), 3.49−3.42 (m, 1H, H-8), 3.10 (dd, *J* = 17.5, 6.2 Hz, 1H, H-9a), 2.93 (td, *J* = 6.4, 3.7 Hz, 1H, H-10), 2.77 (dd, *J* = 17.5, 3.7 Hz, 1H, H-9b), 2.44 (s, 3H, H-19), 2.32 (s, 3H, H-$TsCH_3$), 1.75 (s, 3H, H-13), 1.35 (t, *J* = 7.2 Hz, 3H, H-21), 0.80 (s, 3H, H-12).

^{13}C NMR (125 MHz, $CDCl_3$): δ = 176.4 (C-15), 161.8 (C-17), 159.8 (C-18), 145.2 (C-Ar), 145.0 (C-Ar), 137.4 (C-Ar), 135.2 (C-Ar), 134.4 (C-Ar), 133.6 (C-Ar), 131.4 (C-Ar), 130.05 (C-Ar), 129.97 (C-Ar), 129.6 (C-Ar), 129.3 (C-Ar), 129.2 (C-Ar), 128.2 (C-Ar), 126.96 (C-Ts), 126.85 (C-Ts), 126.1 (C-Ar), 121.5 (C-2), 120.8 (C-5), 118.2 (C-16), 111.7 (C-7), 109.0 (C-Ar), 70.1 (C-11), 61.3 (C-20), 59.8 (C-14), 47.5 (C-10), 40.2 (C-8), 30.0 (C-13), 25.6 (C-9), 23.5 (C-12), 21.7 (C-$TsCH_3$), 14.4 (C-21), 12.0 (C-19).

HRMS (TOF ESI^+), m/z: calcd for $C_{41}H_{39}N_3O_7S_3Na$ $[M+Na]^+$ 804.1842, found 804.1843.

IR (neat) ν_{max}: 3059 (w), 1720 (m), 1369 (m), 1345 (m), 1294 (m), 1177 (s), 1161 (s), 1099 (s).

References

[1] W. C. Still, M. Kahn, A. Mitra, *J. Org. Chem.* **1978**, *43*, 2923–2925.
[2] Bruker, *SAINT+ Integr. Engine, Data Reduct. Software, Bruker Anal. X-ray Instruments Inc., Madison, WI, USA* **2007**.
[3] Bruker, *SADABS, Bruker AXS area Detect. scaling Absorpt. Correct. Bruker Anal. X-ray Instruments Inc., Madison, Wisconsin, USA* **2001**.
[4] L. Palatinus, G. Chapuis, *J. Appl. Crystallogr.* **2007**, *40*, 786–790.
[5] L. Palatinus, S. J. Prathapa, S. van Smaalen, *J. Appl. Crystallogr.* **2012**, *45*, 575–580.
[6] G. M. Sheldrick, *Acta Crystallogr. Sect. A Found. Adv.* **2015**, *71*, 3–8.
[7] G. M. Sheldrick, *Acta Crystallogr. Sect. C Struct. Chem.* **2015**, *71*, 3–8.
[8] G. M. Sheldrick, *Acta Crystallogr. Sect. A Found. Crystallogr.* **2008**, *64*, 112–122.
[9] O. V Dolomanov, L. J. Bourhis, R. J. Gildea, J. A. K. Howard, H. Puschmann, *J. Appl. Crystallogr.* **2009**, *42*, 339–341.
[10] A. L. Spek, *Acta Crystallogr. Sect. D Biol. Crystallogr.* **2009**, *65*, 148–155.
[11] A. L. Spek, *J. Appl. Crystallogr.* **2003**, *36*, 7–13.
[12] S. K. Bharti, R. Roy, *TrAC - Trends Anal. Chem.* **2012**, *35*, 5–26.
[13] A. B. Pangborn, M. A. Giardello, R. H. Grubbs, R. K. Rosen, F. J. Timmers, *Organometallics* **1996**, *15*, 1518–1520.
[14] D. B. G. Williams, M. Lawton, *J. Org. Chem.* **2010**, *75*, 8351–8354.
[15] T. D. Senecal, W. Shu, S. L. Buchwald, *Angew. Chem. Int. Ed.* **2013**, *52*, 10035–10039; *Angew. Chem.* **2013**, *125*, 10219–10223.
[16] W. L. F. Armarego, C. Chai, *Purification of Laboratory Chemicals*, Butterworth-Heinemann, Oxford, **2009**.
[17] W. R. C. Beyer, K. Woithe, B. Luke, M. Schindler, H. Antonicek, J. Scherkenbeck, *Tetrahedron* **2011**, *67*, 3062–3070.
[18] A. Q. Lin, L. Du, Y. C. Fang, F. Z. Wang, T. J. Zhu, Q. Q. Gu, W. M. Zhu, *Chem. Nat. Compd.* **2009**, *45*, 677–680.
[19] V. A. Moorthie, E. M. Mcgarrigle, R. Stenson, V. K. Aggarwal, *Arkivoc* **2007**, 139–151.
[20] C. Deshayes, M. Chabannet, S. Gelin, *Synthesis* **1984**, 868–870.
[21] S. Gelin, M. Chabannet, *Synthesis* **1978**, 448–450.
[22] E. Aktoudianakis, G. Chin, B. K. Corkey, J. Du, K. Elbel, R. H. Jiang, T. Kobayashi, R. Lee, R. Martinez, S. E. Metobo, et al., *Benzimidazole Derivatives as Bromodomain Inhibitors*, **2014**, US 2014/0336190 A1.
[23] M. Arshad, M. A. Fernandez, E. M. McGarrigle, V. K. Aggarwal, *Tetrahedron: Asymmetry* **2010**, *21*, 1771–1776.
[24] O. Illa, M. Namutebi, C. Saha, M. Ostovar, C. C. Chen, M. F. Haddow, S. Nocquet-Thibault, M. Lusi, E. M. McGarrigle, V. K. Aggarwal, *J. Am. Chem. Soc.* **2013**, *135*, 11951–11966.
[25] S. Chimichi, M. Boccalini, B. Cosimelli, F. Dall'Acqua, G. Viola, *Tetrahedron* **2003**, *59*, 5215–5223.
[26] S. Al-Busafi, M. Al-Belushi, K. Al-Muqbali, *Synth. Commun.* **2010**, *40*, 1088–1092.
[27] P. Bravo, G. Gaviraghi, *J. Heterocycl. Chem.* **1977**, *14*, 37–42.

NMR Spectra

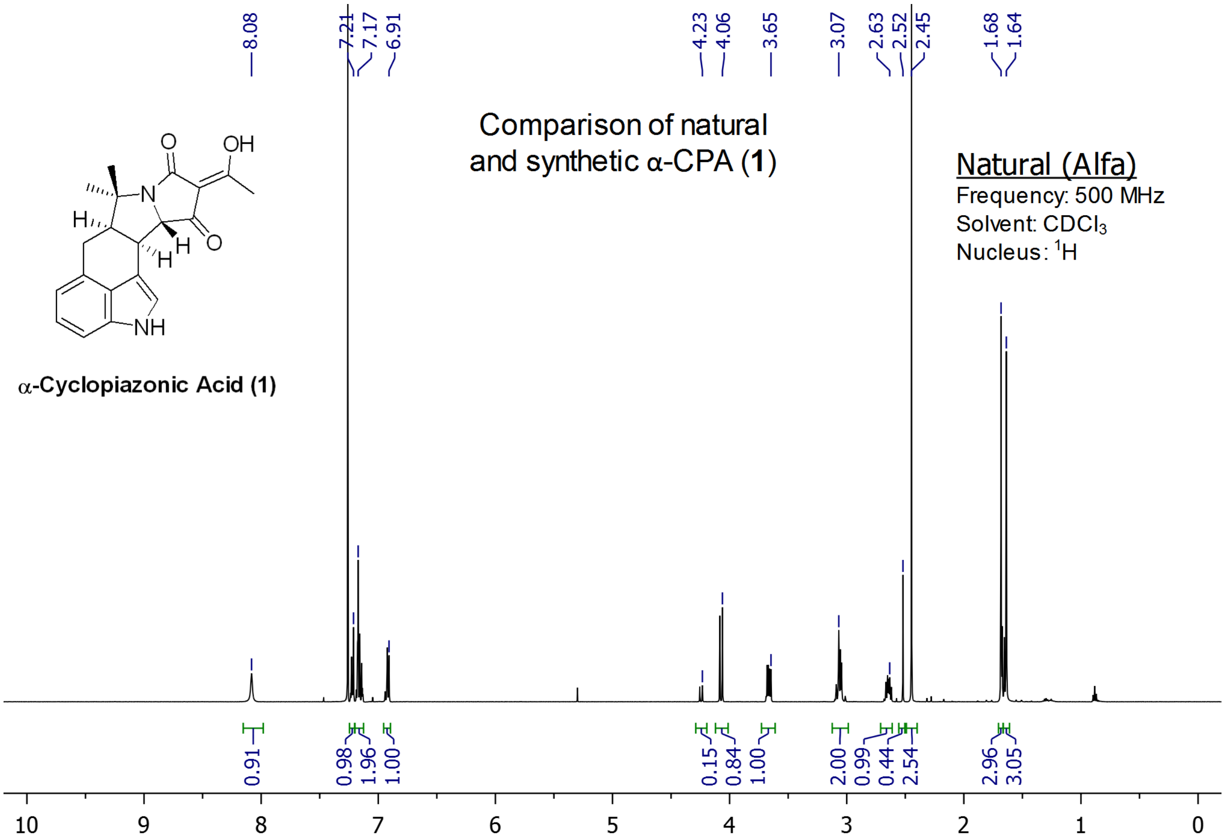
8.08
7.21
7.17
6.91
4.23
4.06
3.65
3.07
2.63
2.52
2.45
1.68
1.64
Comparison of natural and synthetic α-CPA (1)
O
OH
N
H
H
H
O
NH
α-Cyclopiazonic Acid (1)
Natural (Alfa)
Frequency: 500 MHz
Solvent: CDCl3
Nucleus: 1H
0.91
0.98
1.96
1.00
0.15
0.84
1.00
2.00
0.99
0.44
2.54
2.96
3.05
10 9 8 7 6 5 4 3 2 1 0

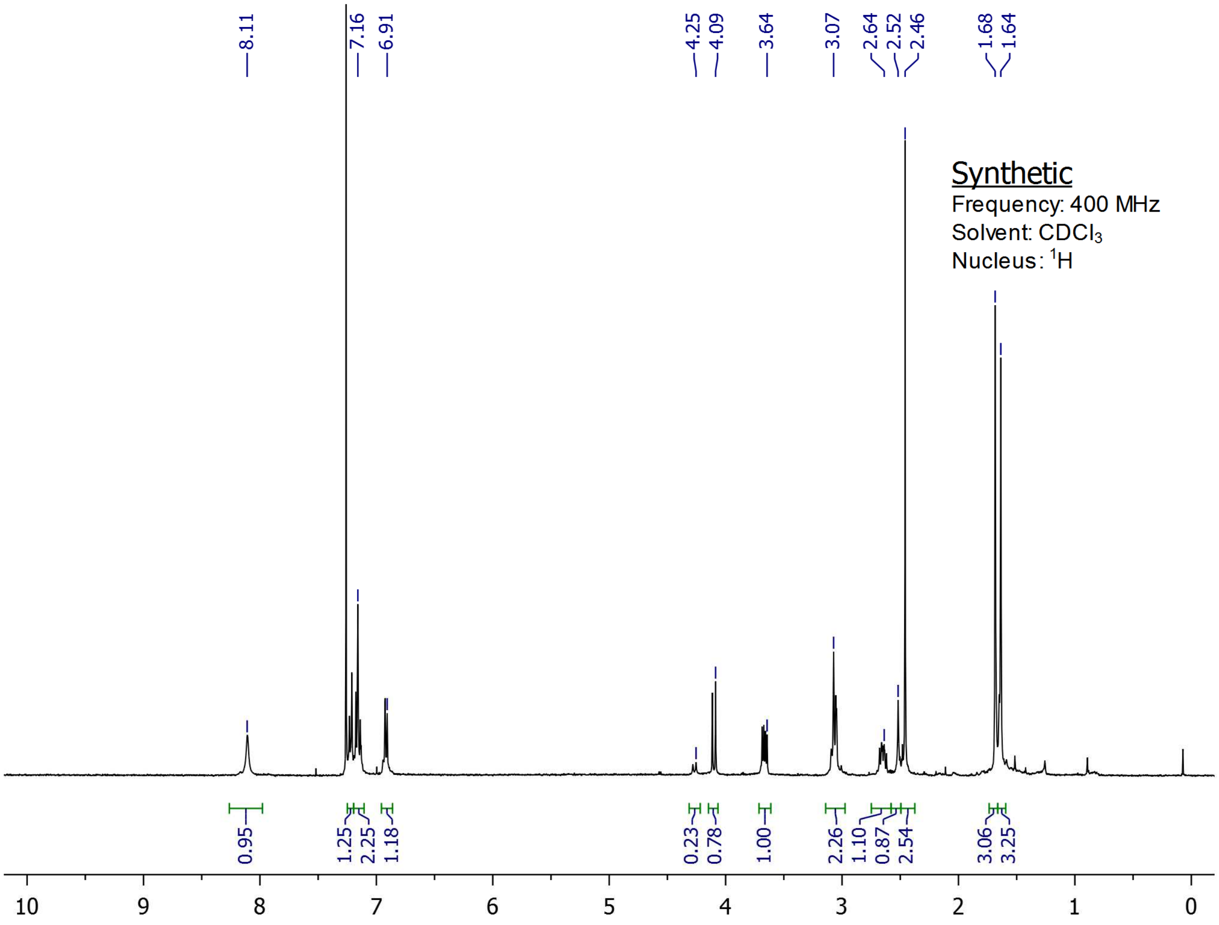
8.11
7.16
6.91
4.25
4.09
3.64
3.07
2.64
2.52
2.46
1.68
1.64
Synthetic
Frequency: 400 MHz
Solvent: CDCl3
Nucleus: 1H
0.95
1.25
2.25
1.18
0.23
0.78
1.00
2.26
1.10
0.87
2.54
3.06
3.25
10 9 8 7 6 5 4 3 2 1 0

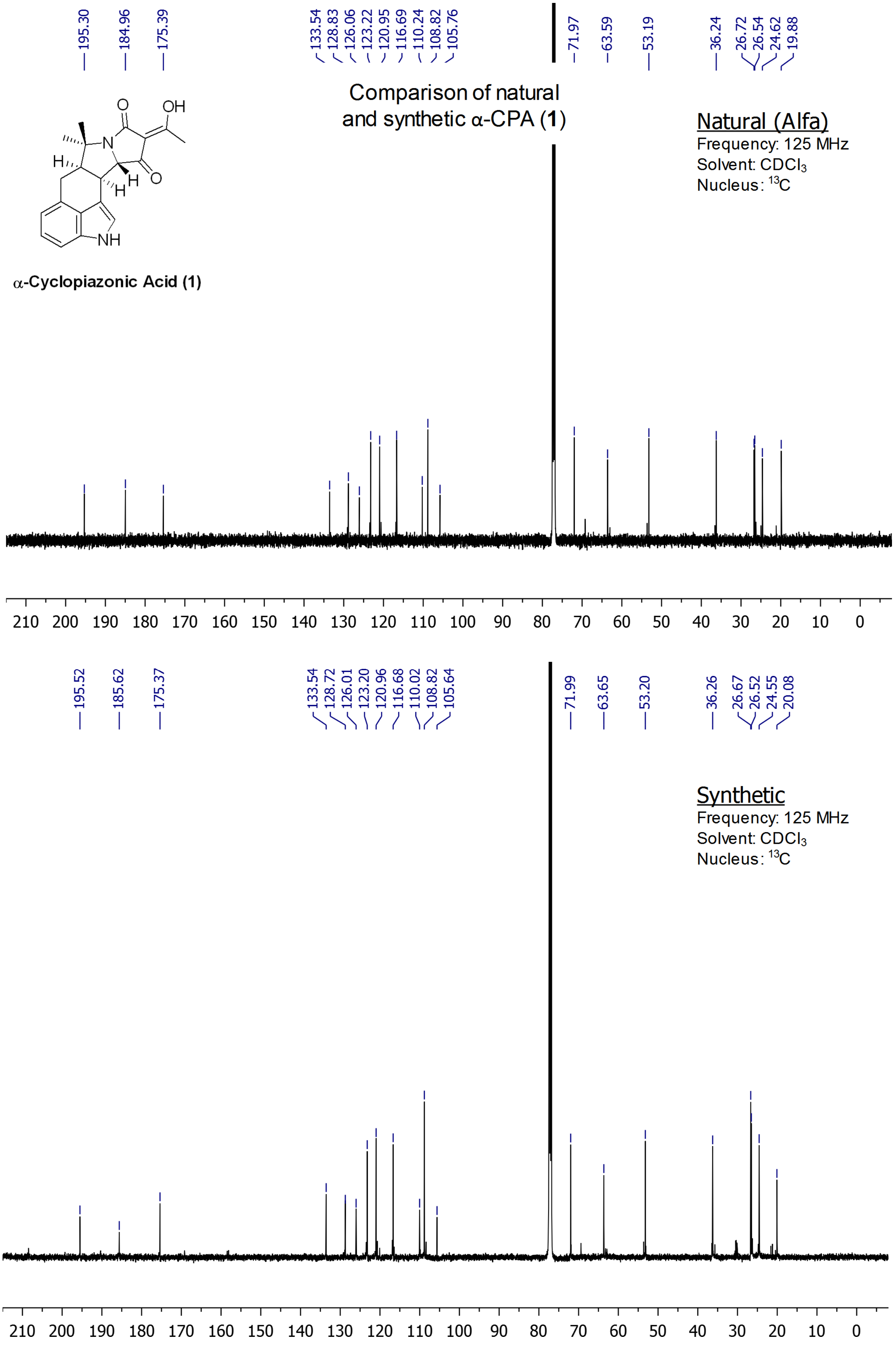

Comparison of natural and synthetic α-CPA (1)
α-Cyclopiazonic Acid (1)
Natural (Alfa)
Frequency: 125 MHz
Solvent: CDCl3
Nucleus: 13C
195.30 184.96 175.39 133.54 128.83 126.06 123.22 120.95 116.69 110.24 108.82 105.76 71.97 63.59 53.19 36.24 26.72 26.54 24.62 19.88
Synthetic
Frequency: 125 MHz
Solvent: CDCl3
Nucleus: 13C
195.52 185.62 175.37 133.54 128.72 126.01 123.20 120.96 116.68 110.02 108.82 105.64 71.99 63.65 53.20 36.26 26.67 26.52 24.55 20.08
210 200 190 180 170 160 150 140 130 120 110 100 90 80 70 60 50 40 30 20 10 0

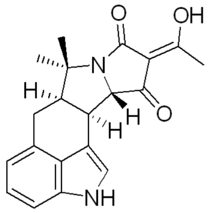

α-Cyclopiazonic Acid (1)

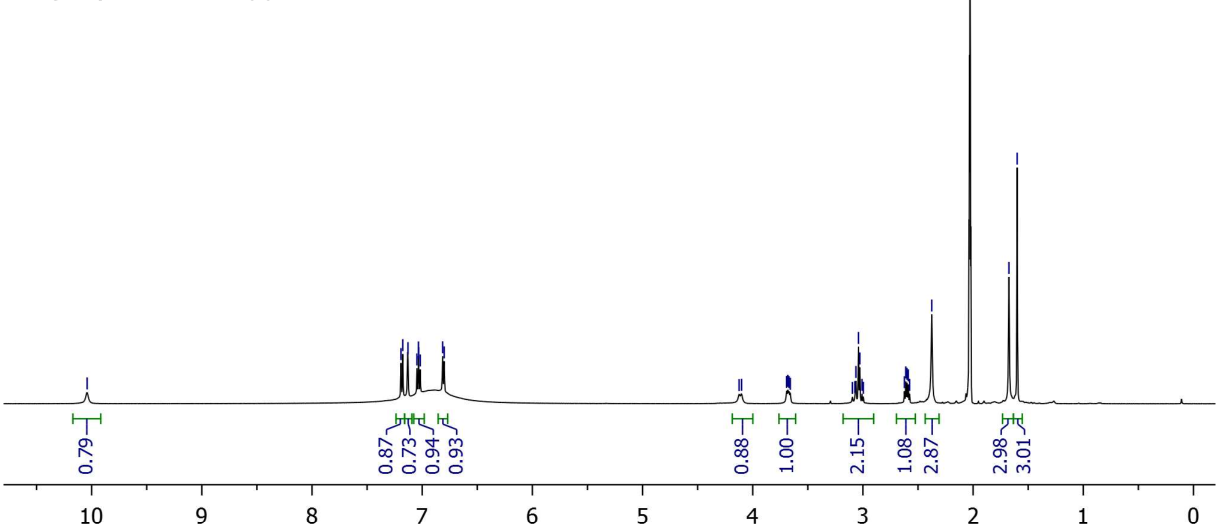

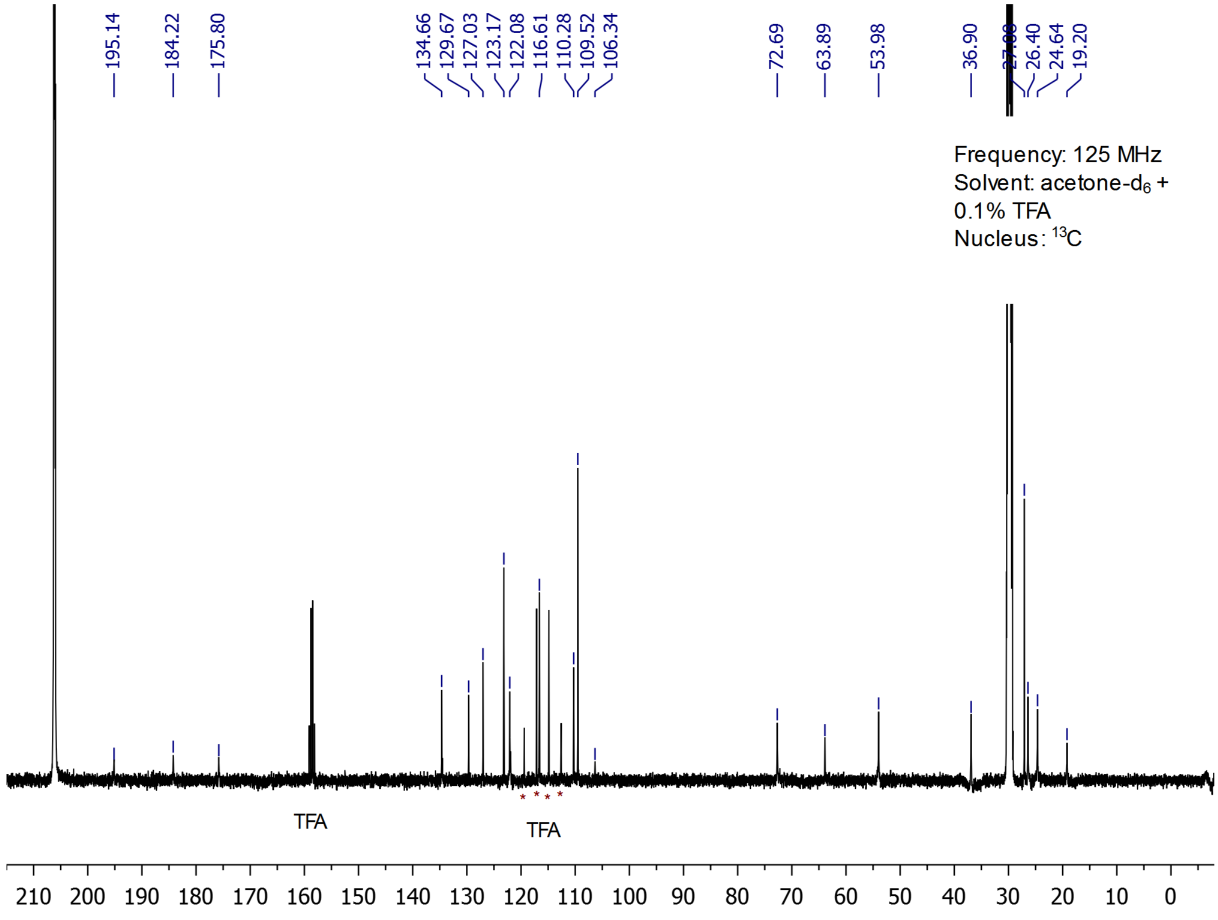

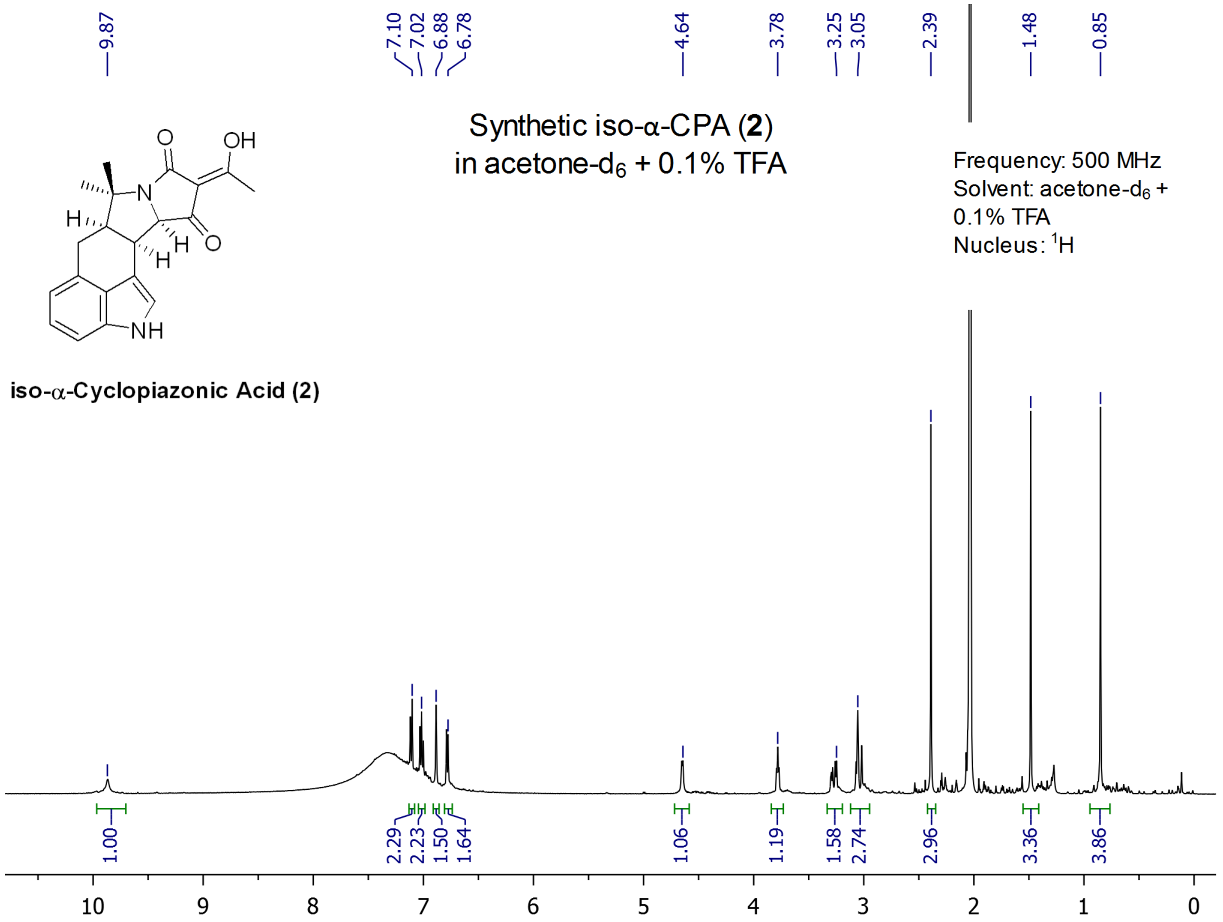

iso-α-Cyclopiazonic Acid (2)

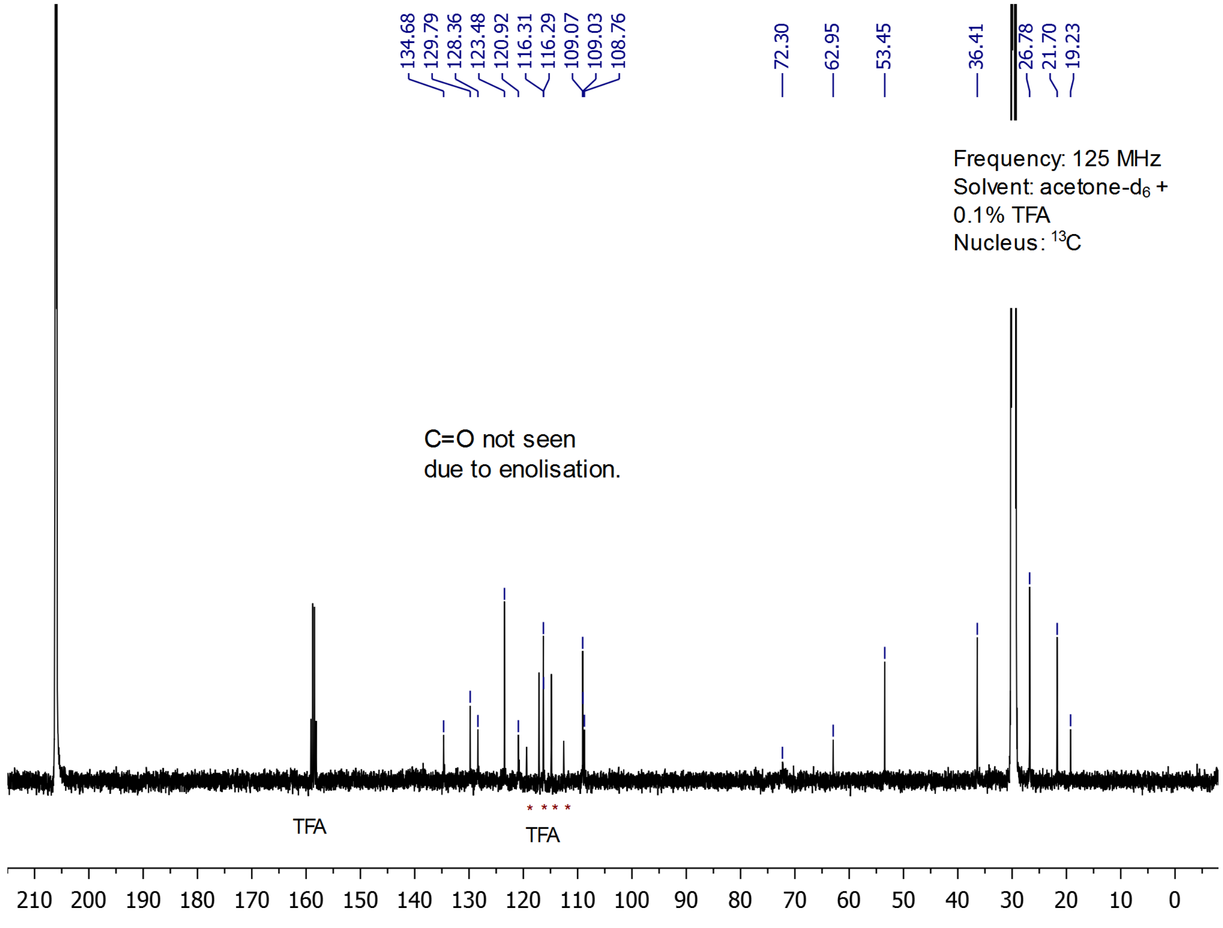

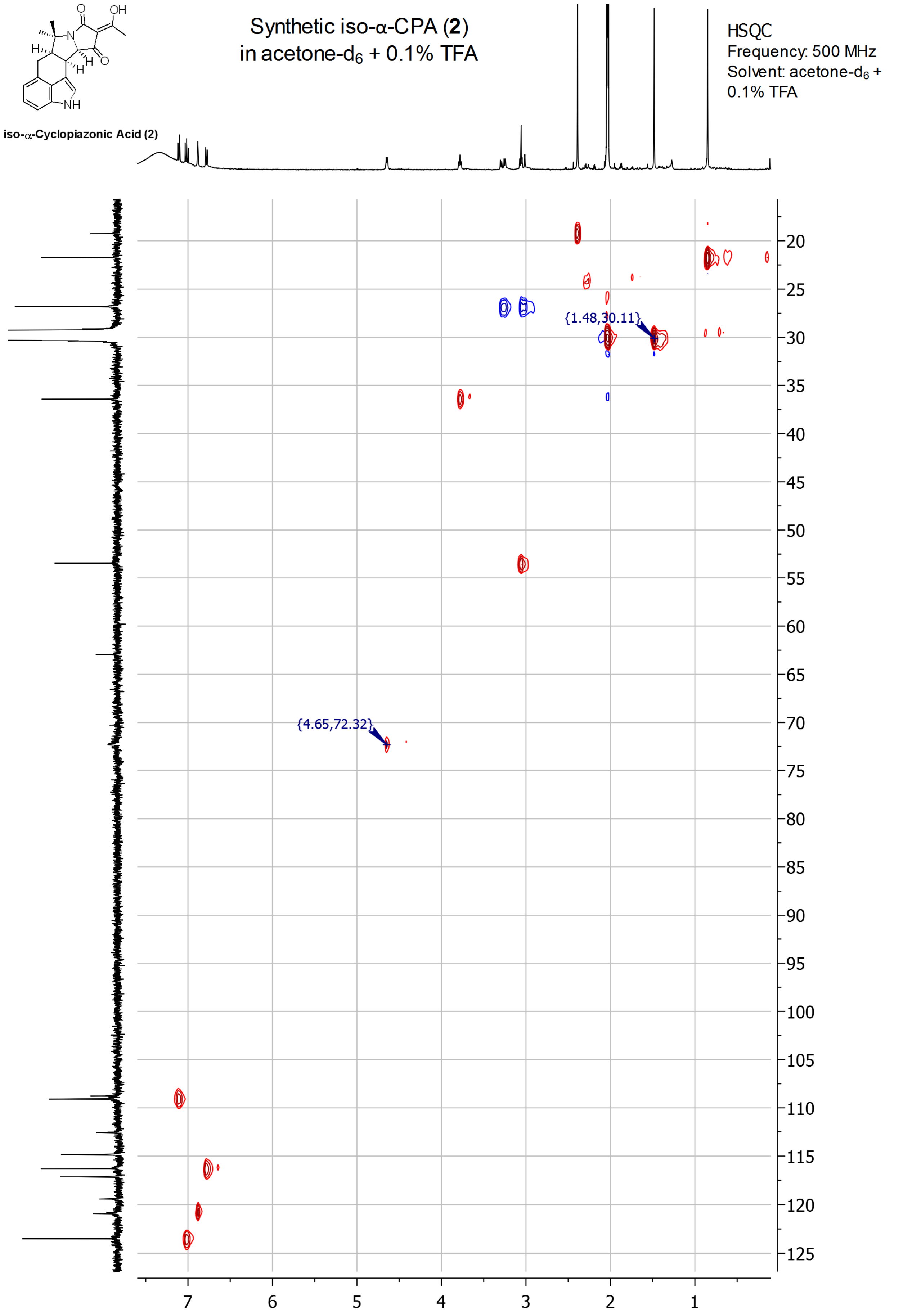

O
OH
N
H
H
H
O
NH
iso-α-Cyclopiazonic Acid (2)
Synthetic iso-α-CPA (2)
in acetone-d6 + 0.1% TFA
HSQC
Frequency: 500 MHz
Solvent: acetone-d6 +
0.1% TFA
{1.48,30.11}
{4.65,72.32}
20
25
30
35
40
45
50
55
60
65
70
75
80
85
90
95
100
105
110
115
120
125
7
6
5
4
3
2
1

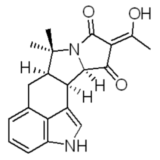

iso-α-Cyclopiazonic Acid (2)

Synthetic iso-α-CPA (**2**)
in acetone-d_6 + 0.1% TFA

HMBC
Frequency: 500 MHz
Solvent: acetone-d_6 +
0.1% TFA

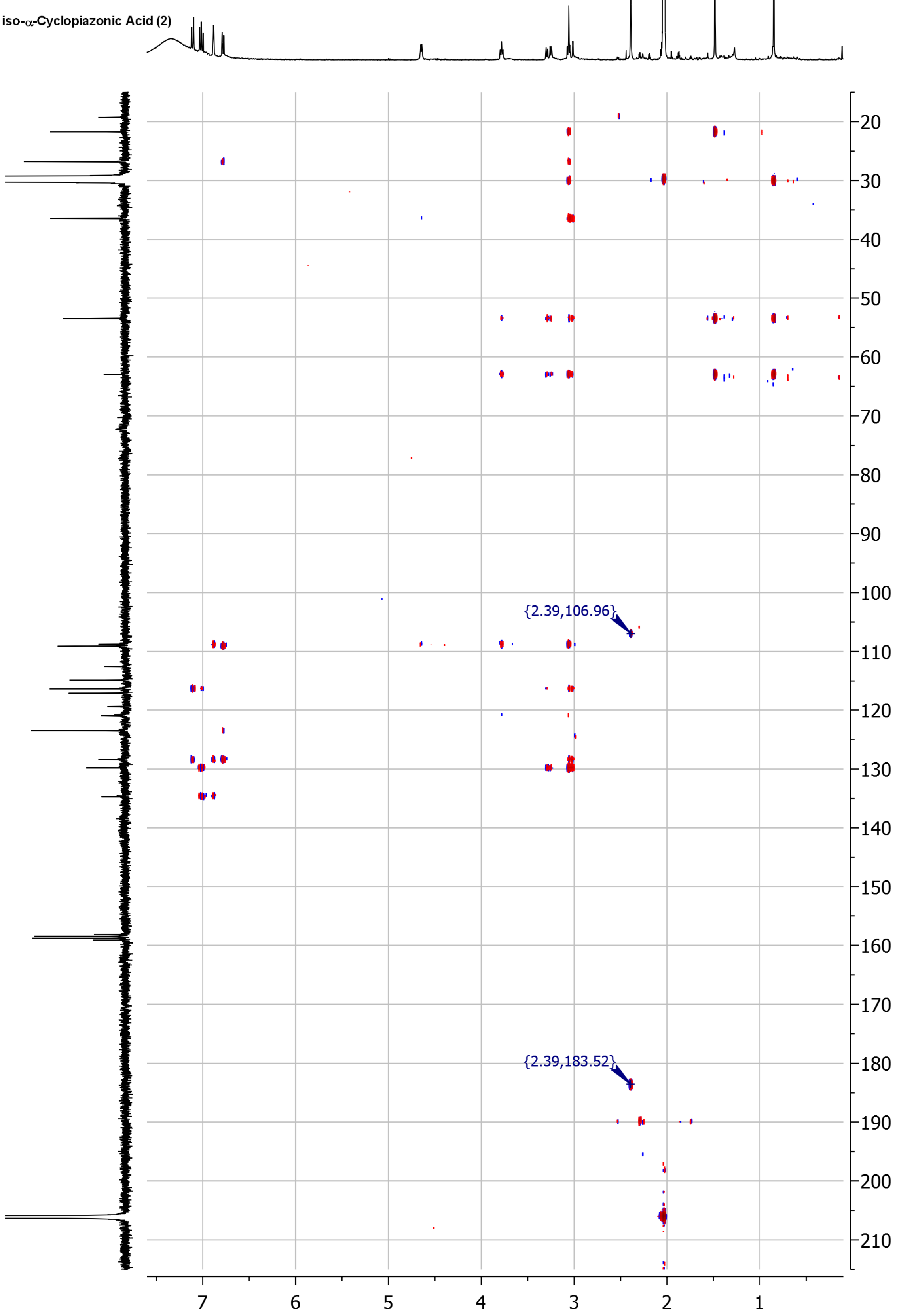

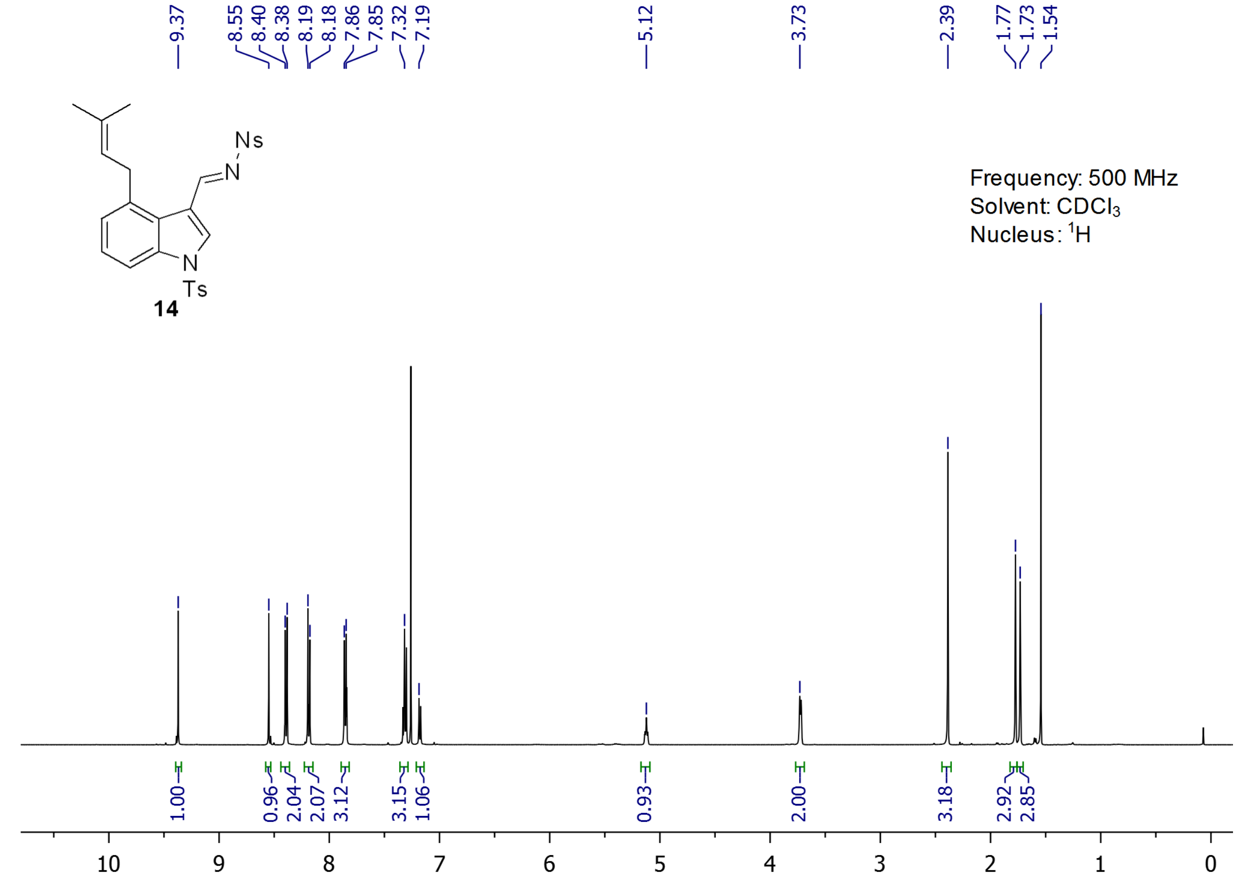
9.37
8.55
8.40
8.38
8.19
8.18
7.86
7.85
7.32
7.19
5.12
3.73
2.39
1.77
1.73
1.54
Ns
N
N
Ts
14
Frequency: 500 MHz
Solvent: CDCl3
Nucleus: 1H
1.00
0.96
2.04
2.07
3.12
3.15
1.06
0.93
2.00
3.18
2.92
2.85
10 9 8 7 6 5 4 3 2 1 0

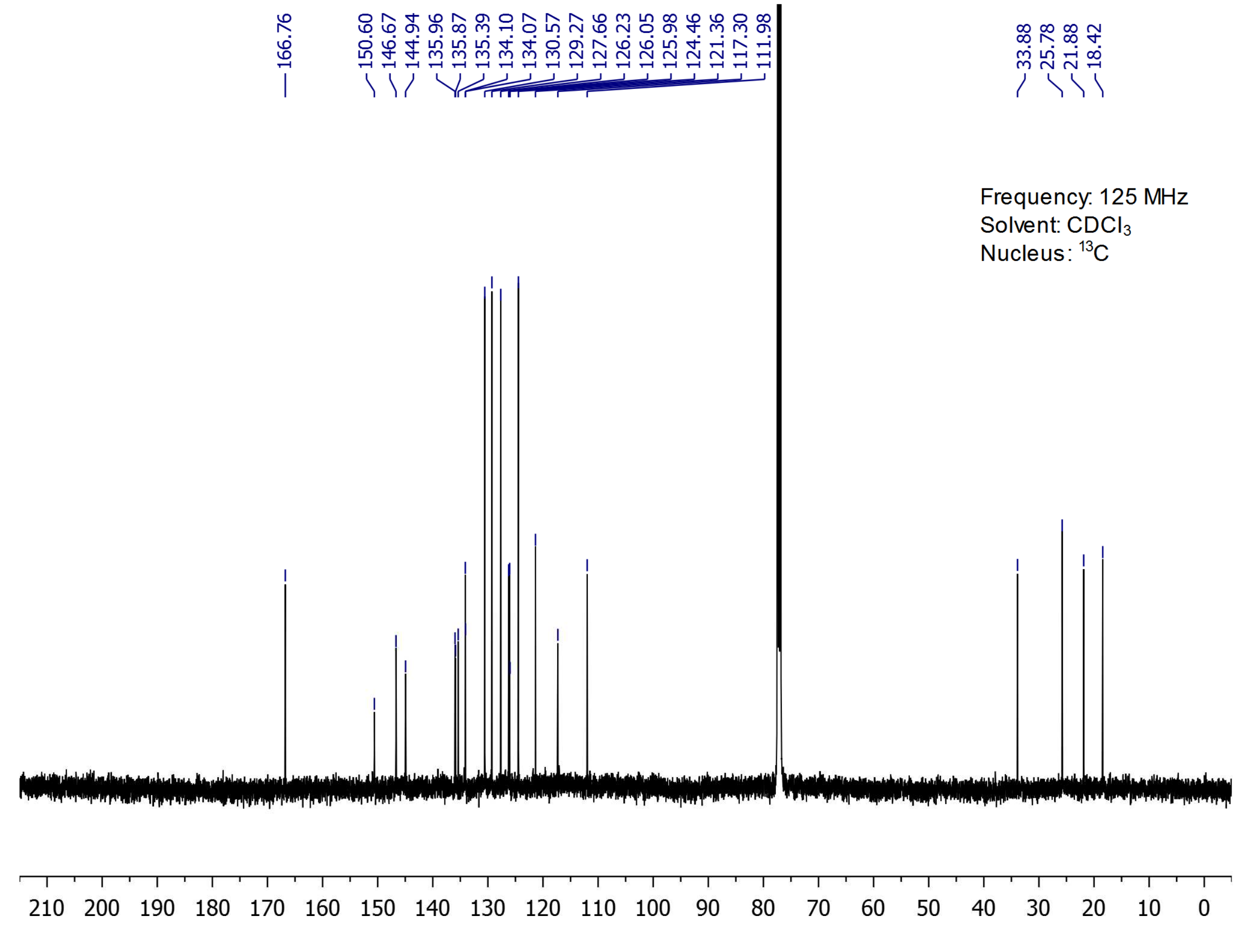
166.76
150.60
146.67
144.94
135.96
135.87
135.39
134.10
134.07
130.57
129.27
127.66
126.23
126.05
125.98
124.46
121.36
117.30
111.98
33.88
25.78
21.88
18.42
Frequency: 125 MHz
Solvent: CDCl3
Nucleus: 13C
210 200 190 180 170 160 150 140 130 120 110 100 90 80 70 60 50 40 30 20 10 0

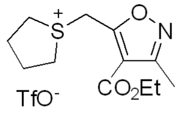
TfO-
CO2Et

15a

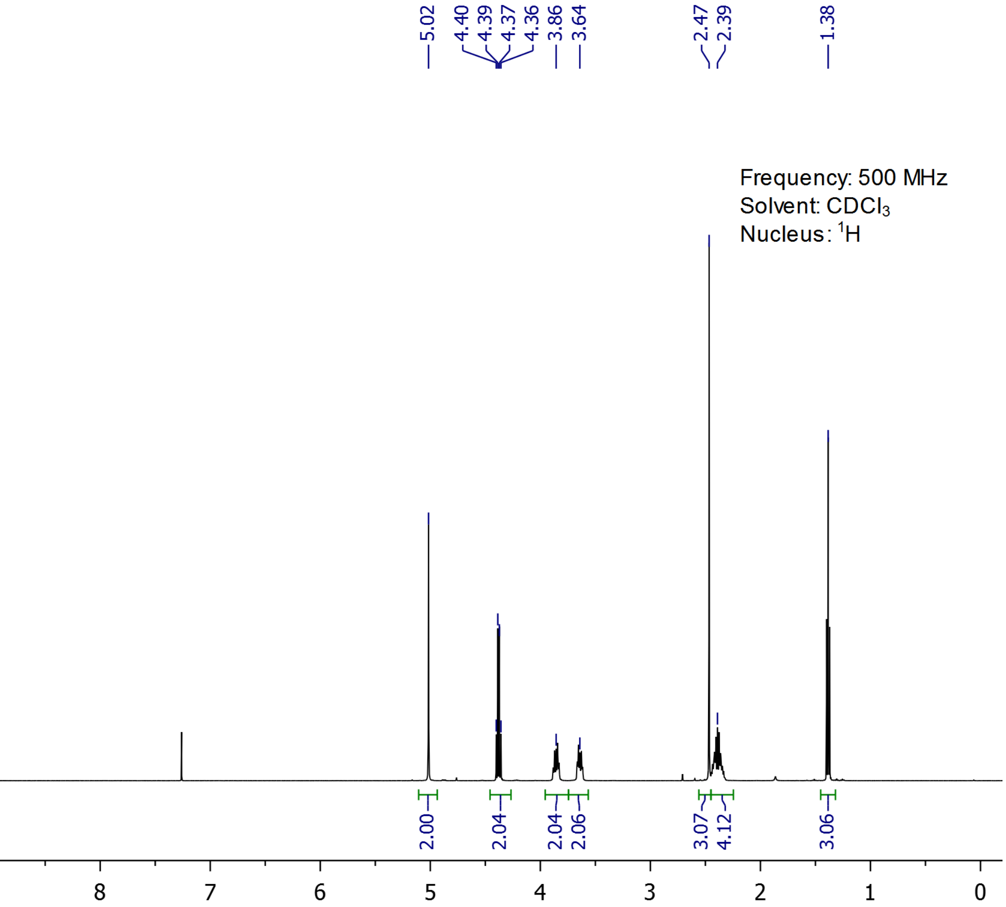
5.02
4.40
4.39
4.37
4.36
3.86
3.64
2.47
2.39
1.38
Frequency: 500 MHz
Solvent: CDCl3
Nucleus: 1H
2.00
2.04
2.04
2.06
3.07
4.12
3.06
10
9
8
7
6
5
4
3
2
1
0

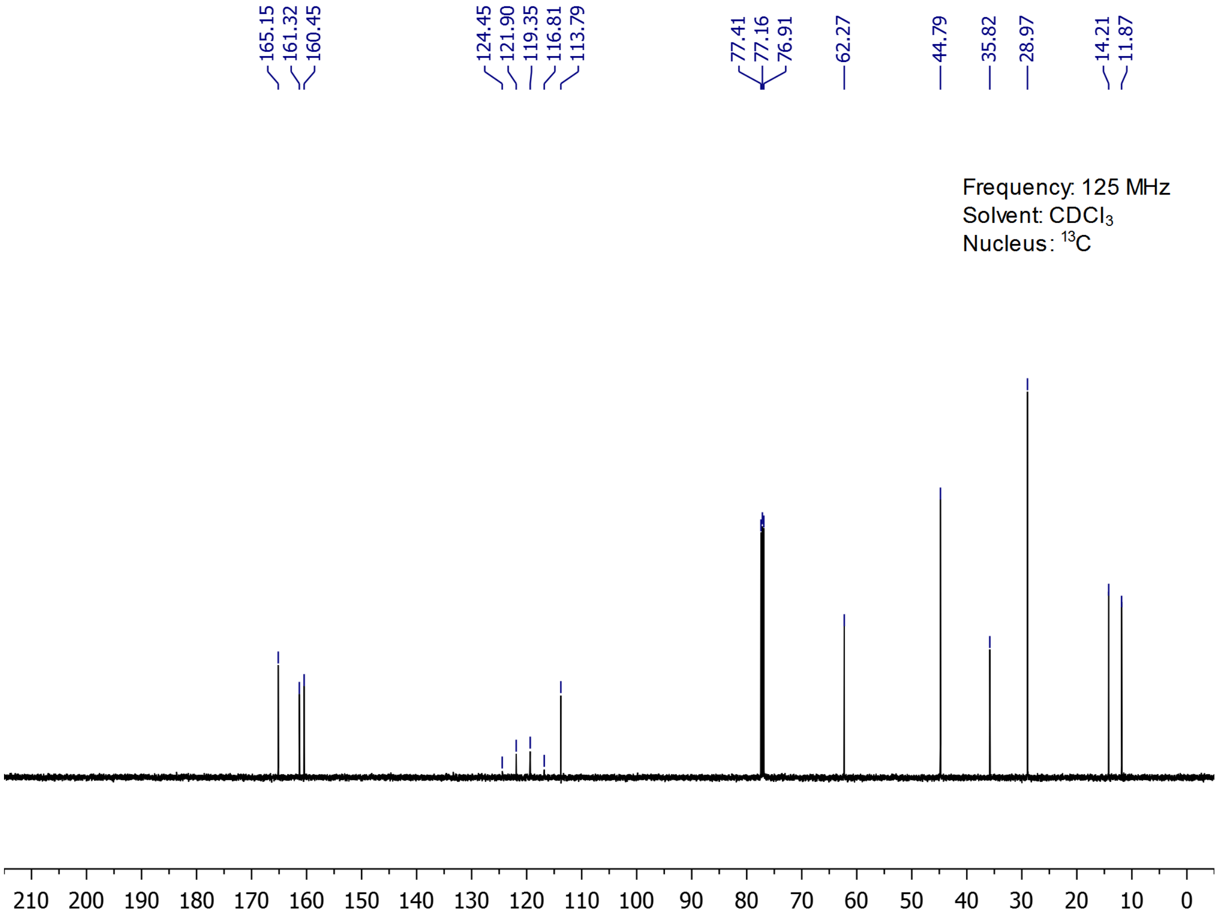
165.15
161.32
160.45
124.45
121.90
119.35
116.81
113.79
77.41
77.16
76.91
62.27
44.79
35.82
28.97
14.21
11.87
Frequency: 125 MHz
Solvent: CDCl3
Nucleus: 13C
210
200
190
180
170
160
150
140
130
120
110
100
90
80
70
60
50
40
30
20
10
0

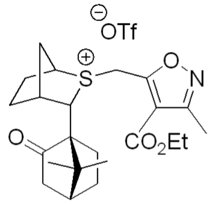
OTf
S
O
N
O
CO2Et

(-)-15b

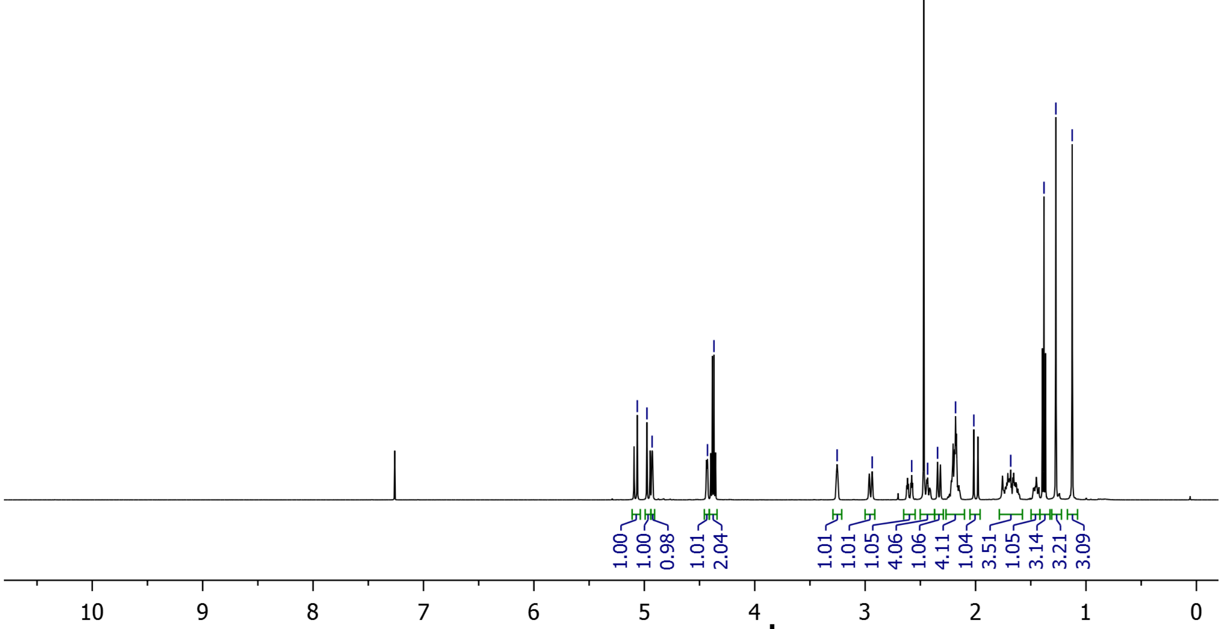
5.06
4.98
4.93
4.43
4.37
3.25
2.94
2.58
2.47
2.43
2.34
2.18
2.02
1.68
1.38
1.27
1.12
Frequency: 500 MHz
Solvent: CDCl3
Nucleus: 1H
1.00
1.00
0.98
1.01
2.04
1.01
1.01
1.05
4.06
1.06
4.11
1.04
3.51
1.05
3.14
3.21
3.09
10 9 8 7 6 5 4 3 2 1 0

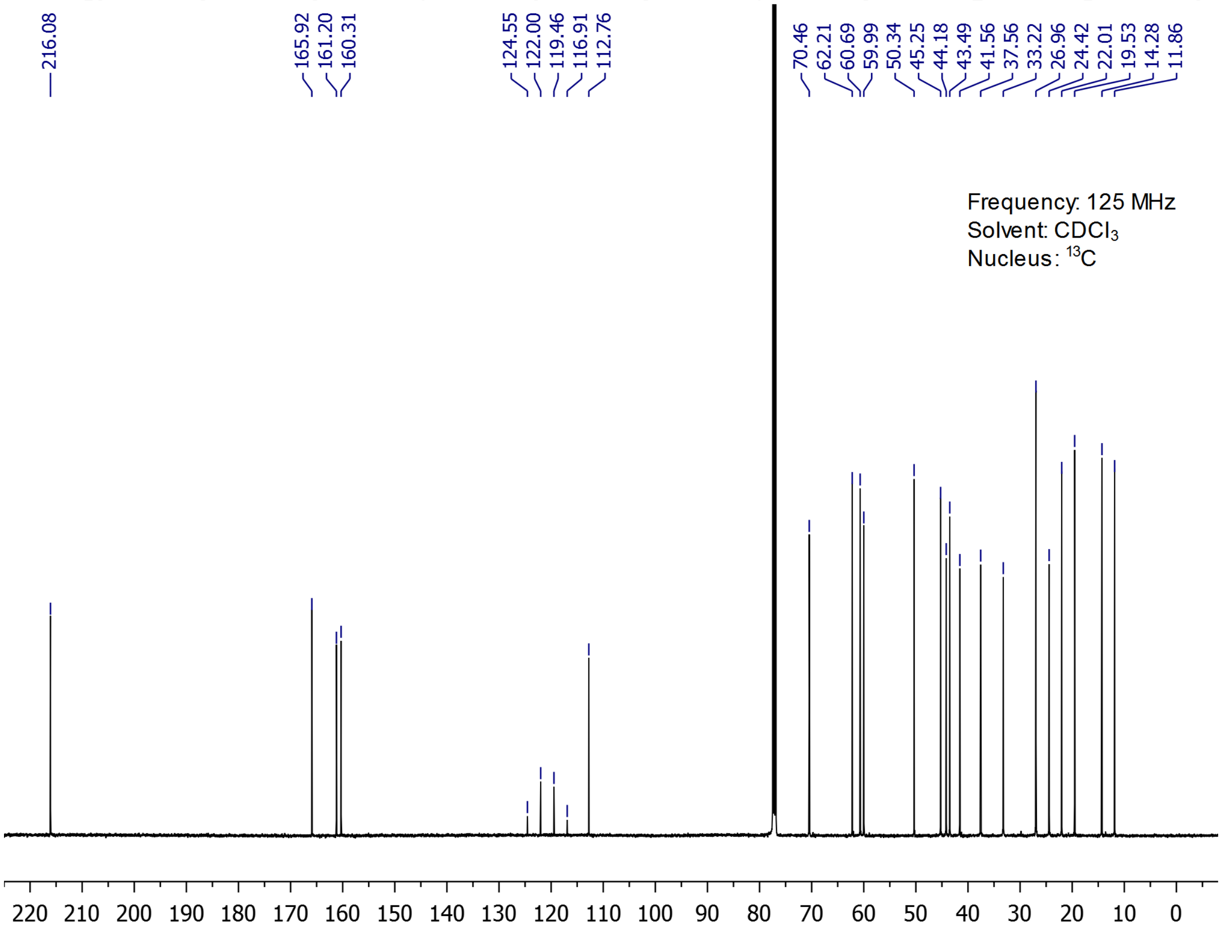
216.08
165.92
161.20
160.31
124.55
122.00
119.46
116.91
112.76
70.46
62.21
60.69
59.99
50.34
45.25
44.18
43.49
41.56
37.56
33.22
26.96
24.42
22.01
19.53
14.28
11.86
Frequency: 125 MHz
Solvent: CDCl3
Nucleus: 13C
220 210 200 190 180 170 160 150 140 130 120 110 100 90 80 70 60 50 40 30 20 10 0

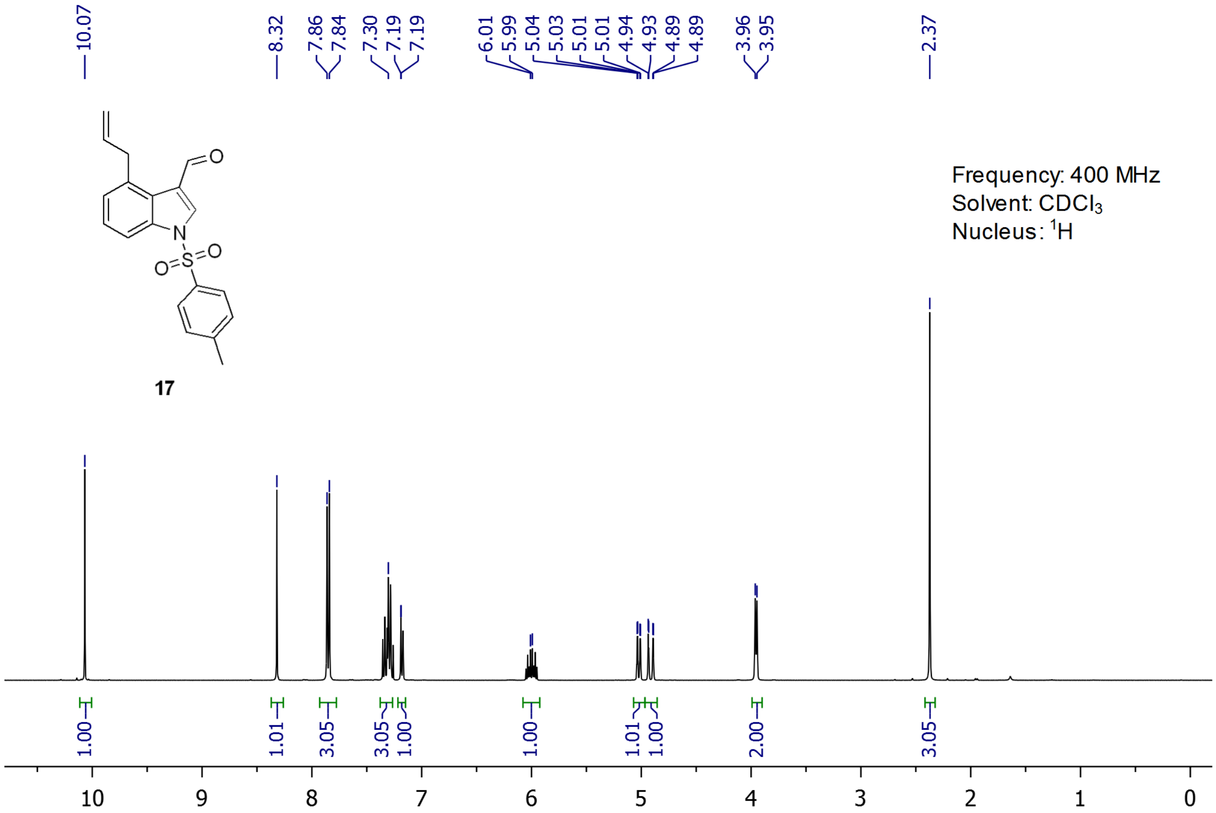
10.07
8.32
7.86
7.84
7.30
7.19
7.19
6.01
5.99
5.04
5.03
5.01
5.01
4.94
4.93
4.89
4.89
3.96
3.95
2.37
O
N
O=S=O
17
Frequency: 400 MHz
Solvent: CDCl3
Nucleus: 1H
1.00
1.01
3.05
3.05
1.00
1.00
1.01
1.00
2.00
3.05
10 9 8 7 6 5 4 3 2 1 0

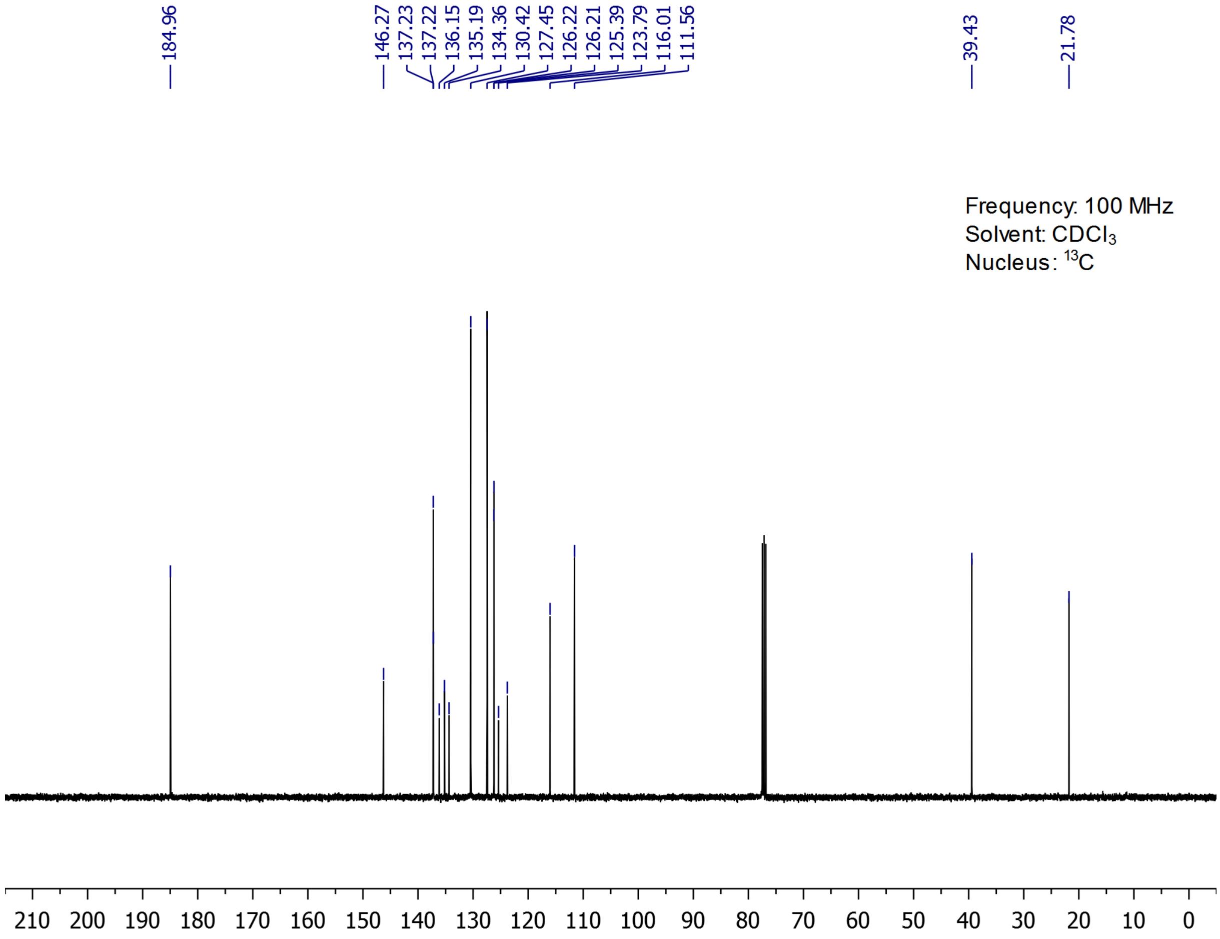
184.96
146.27
137.23
137.22
136.15
135.19
134.36
130.42
127.45
126.22
126.21
125.39
123.79
116.01
111.56
39.43
21.78
Frequency: 100 MHz
Solvent: CDCl3
Nucleus: 13C
210 200 190 180 170 160 150 140 130 120 110 100 90 80 70 60 50 40 30 20 10 0

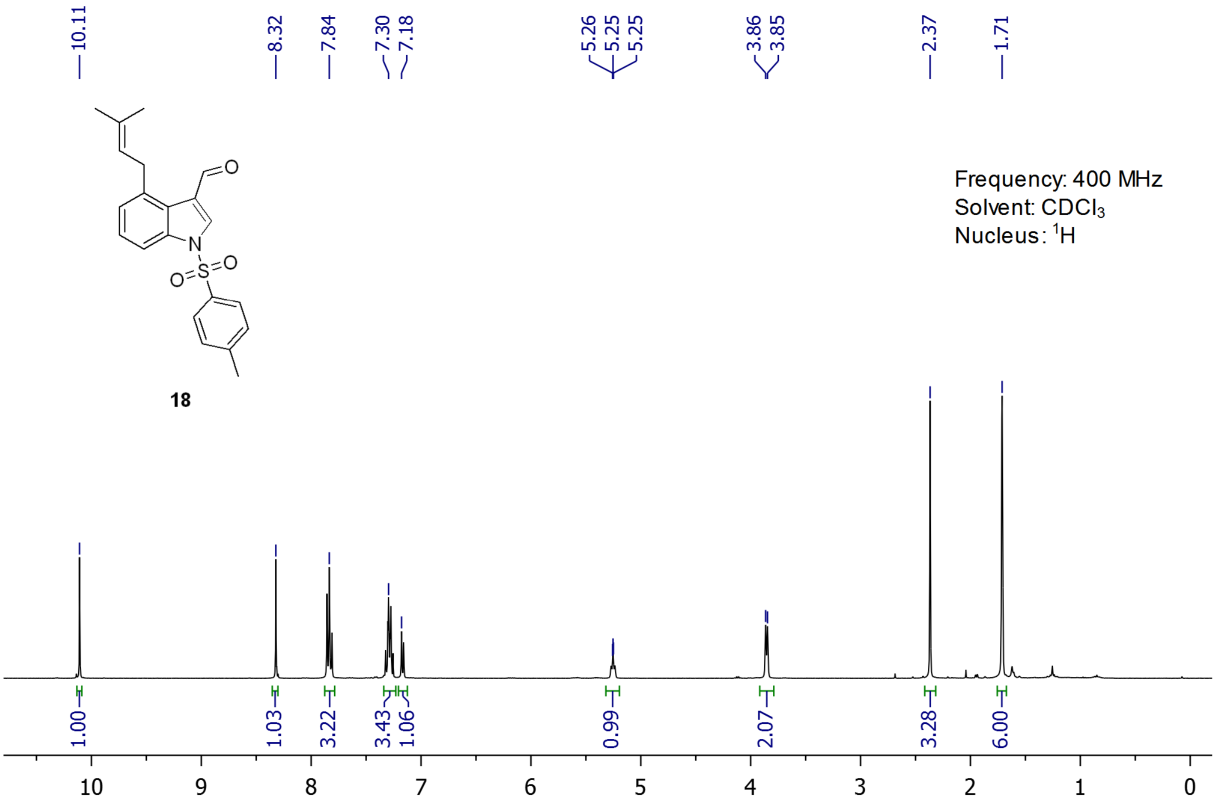
18
10.11
8.32
7.84
7.30
7.18
5.26
5.25
5.25
3.86
3.85
2.37
1.71
Frequency: 400 MHz
Solvent: $CDCl_3$
Nucleus: ^1H
1.00
1.03
3.22
3.43
1.06
0.99
2.07
3.28
6.00
10 9 8 7 6 5 4 3 2 1 0

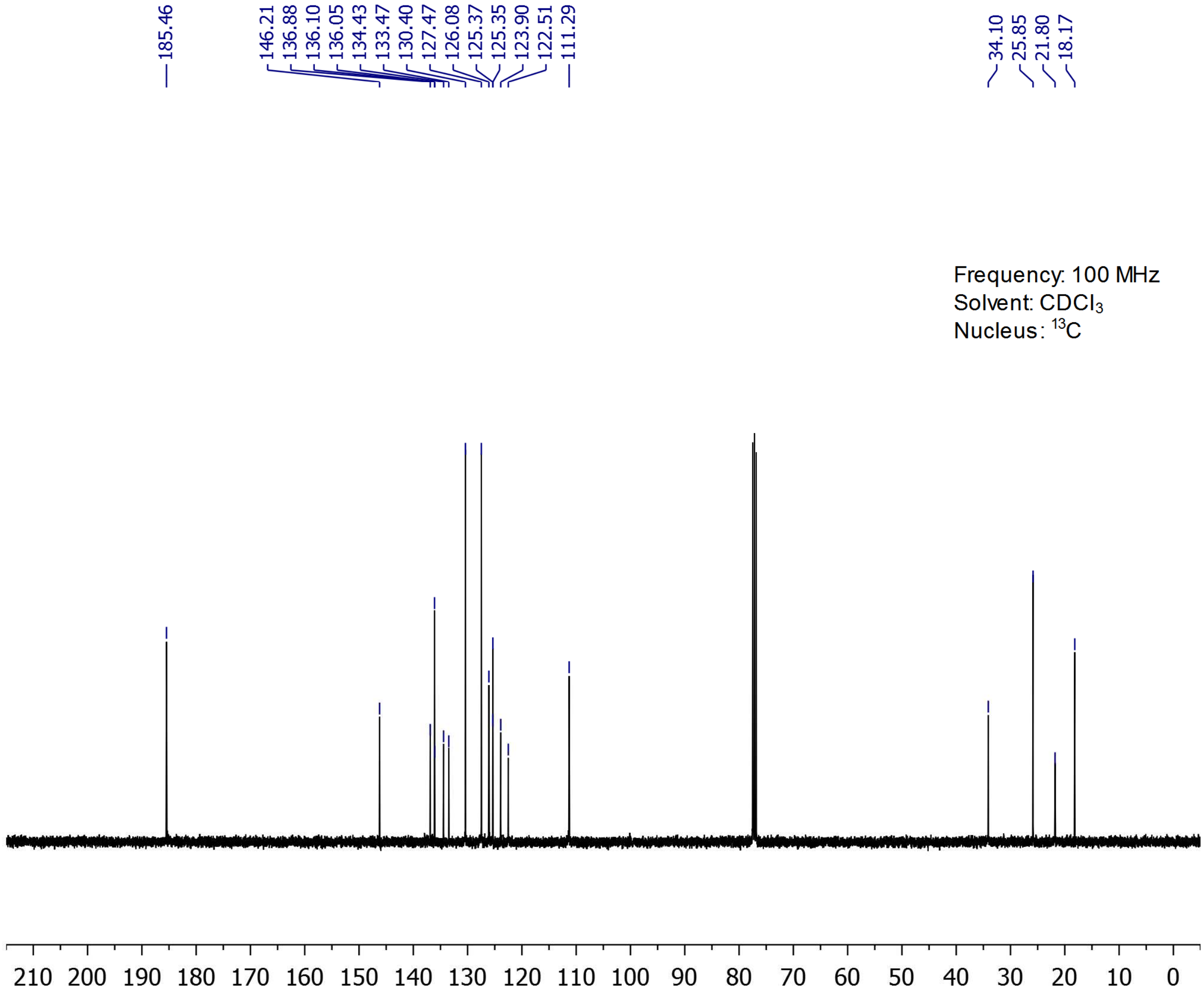
185.46
146.21
136.88
136.10
136.05
134.43
133.47
130.40
127.47
126.08
125.37
125.35
123.90
122.51
111.29
34.10
25.85
21.80
18.17
Frequency: 100 MHz
Solvent: $CDCl_3$
Nucleus: ^{13}C
210 200 190 180 170 160 150 140 130 120 110 100 90 80 70 60 50 40 30 20 10 0

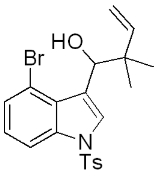
Br
HO
N
Ts

19

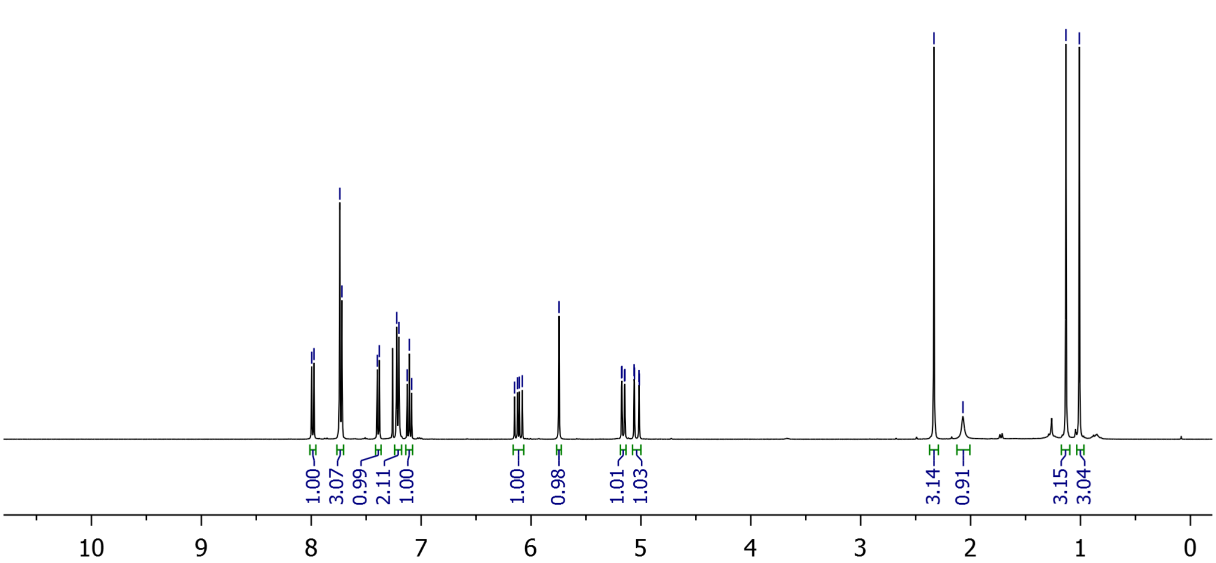
8.00
7.98
7.74
7.72
7.40
7.38
7.22
7.20
7.13
7.11
7.09
6.15
6.12
6.11
6.08
5.75
5.18
5.17
5.15
5.15
5.06
5.06
5.02
5.01
2.33
2.07
1.13
1.01
Frequency: 400 MHz
Solvent: $CDCl_3$
Nucleus: ^1H
1.00
3.07
0.99
2.11
1.00
1.00
0.98
1.01
1.03
3.14
0.91
3.15
3.04
10
9
8
7
6
5
4
3
2
1
0

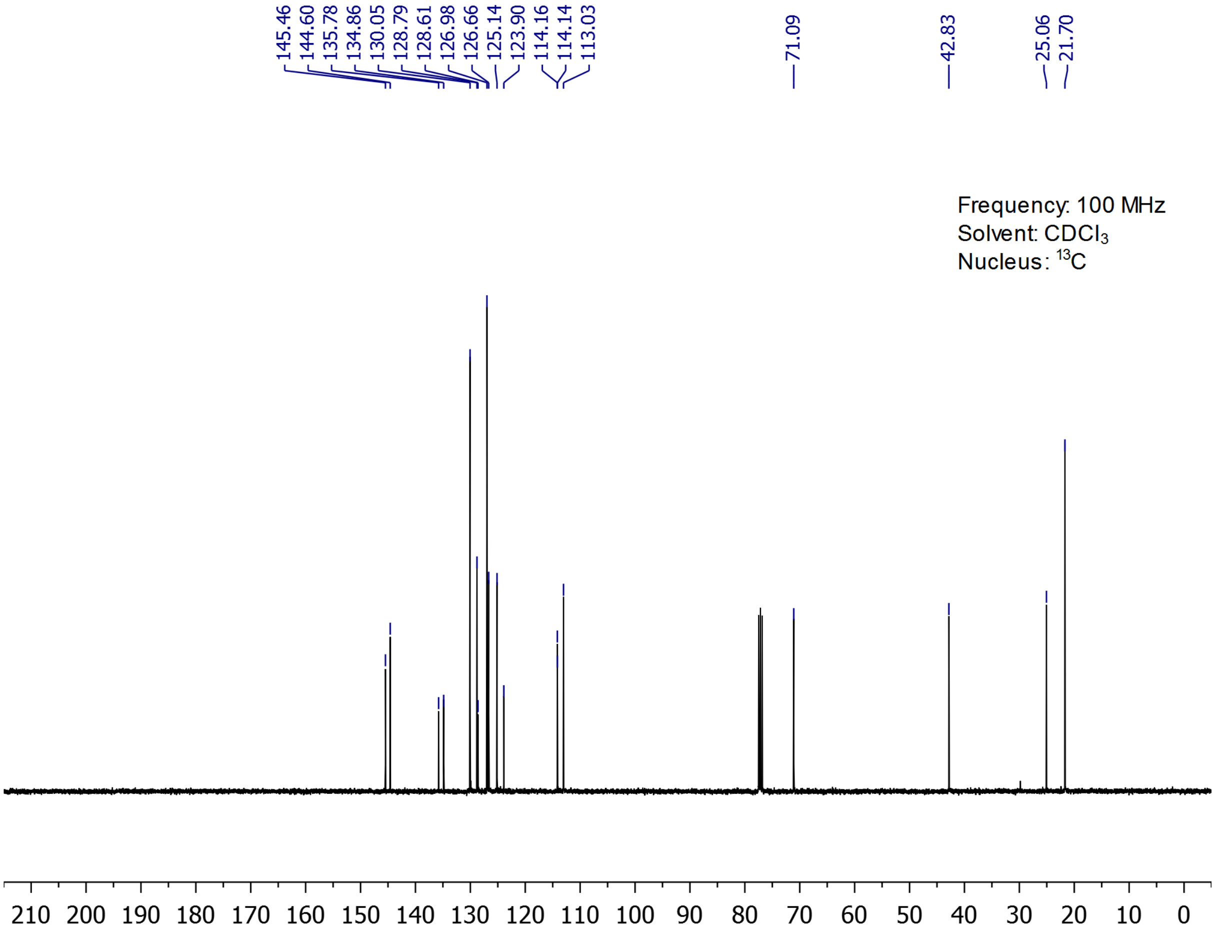
145.46
144.60
135.78
134.86
130.05
128.79
128.61
126.98
126.66
125.14
123.90
114.16
114.14
113.03
71.09
42.83
25.06
21.70
Frequency: 100 MHz
Solvent: $CDCl_3$
Nucleus: ^{13}C
210
200
190
180
170
160
150
140
130
120
110
100
90
80
70
60
50
40
30
20
10
0

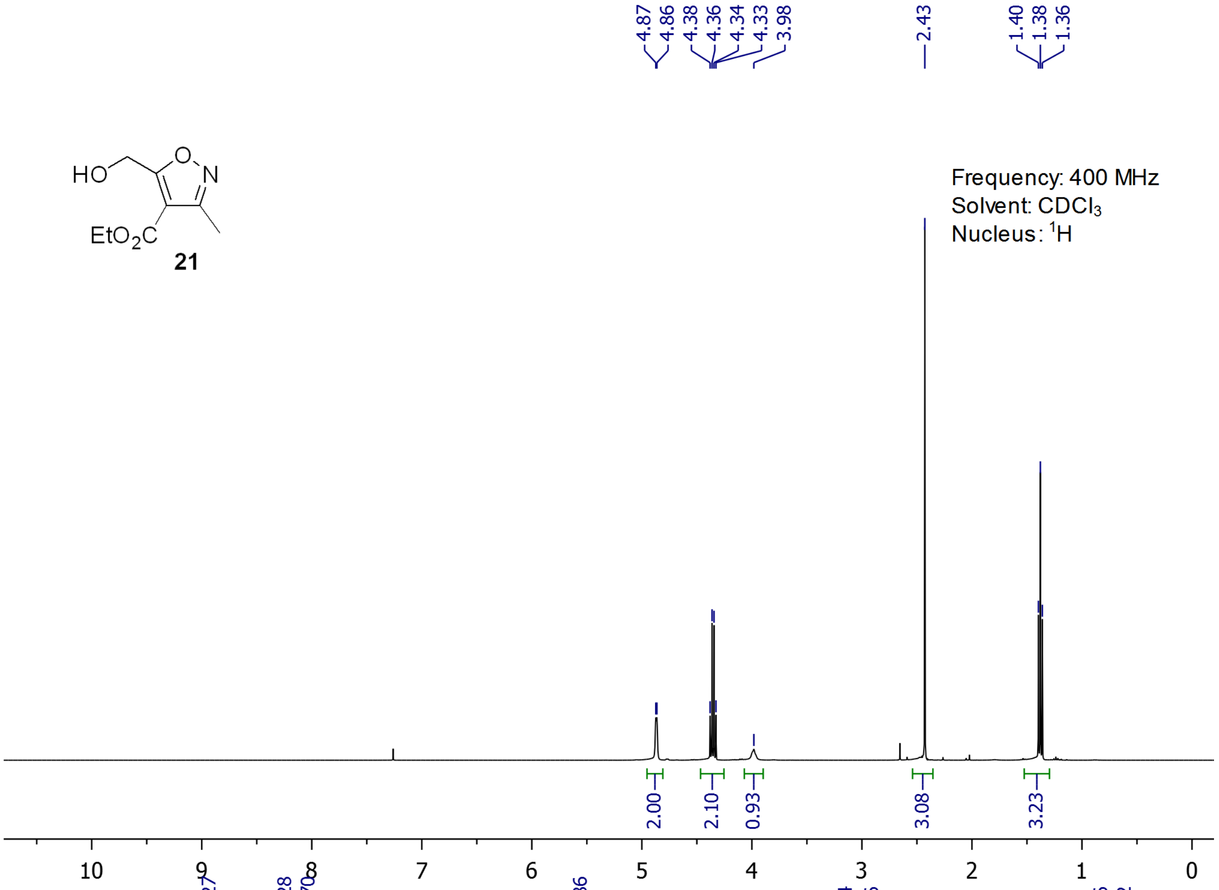
HO
O
N
EtO2C
21
4.87
4.86
4.38
4.36
4.34
4.33
3.98
2.43
1.40
1.38
1.36
Frequency: 400 MHz
Solvent: CDCl3
Nucleus: 1H
2.00
2.10
0.93
3.08
3.23

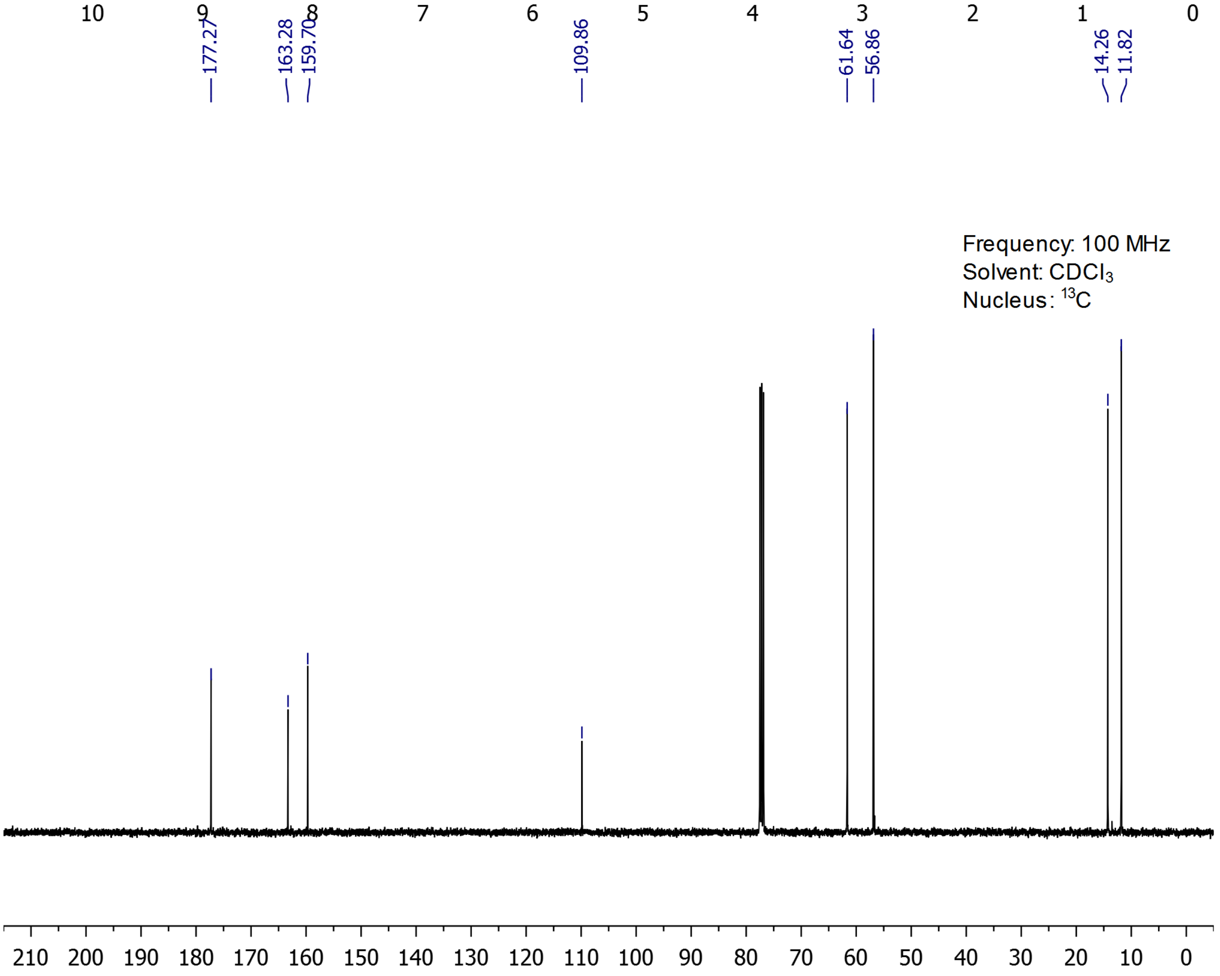
177.27
163.28
159.70
109.86
61.64
56.86
14.26
11.82
Frequency: 100 MHz
Solvent: CDCl3
Nucleus: 13C

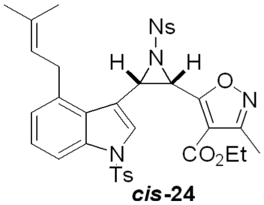
cis-**24**

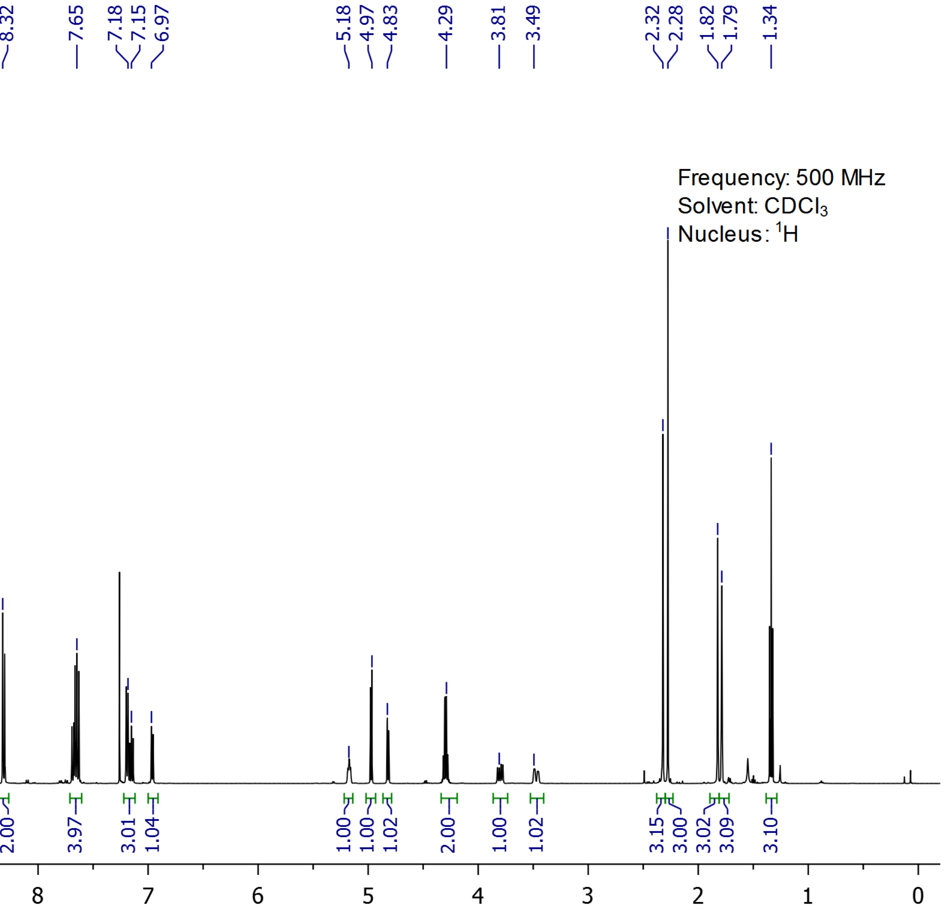
8.44
8.32
7.65
7.18
7.15
6.97
5.18
4.97
4.83
4.29
3.81
3.49
2.32
2.28
1.82
1.79
1.34
Frequency: 500 MHz
Solvent: CDCl3
Nucleus: 1H
1.97
2.00
3.97
3.01
1.04
1.00
1.00
1.02
2.00
1.00
1.02
3.15
3.00
3.02
3.09
3.10
10 9 8 7 6 5 4 3 2 1 0

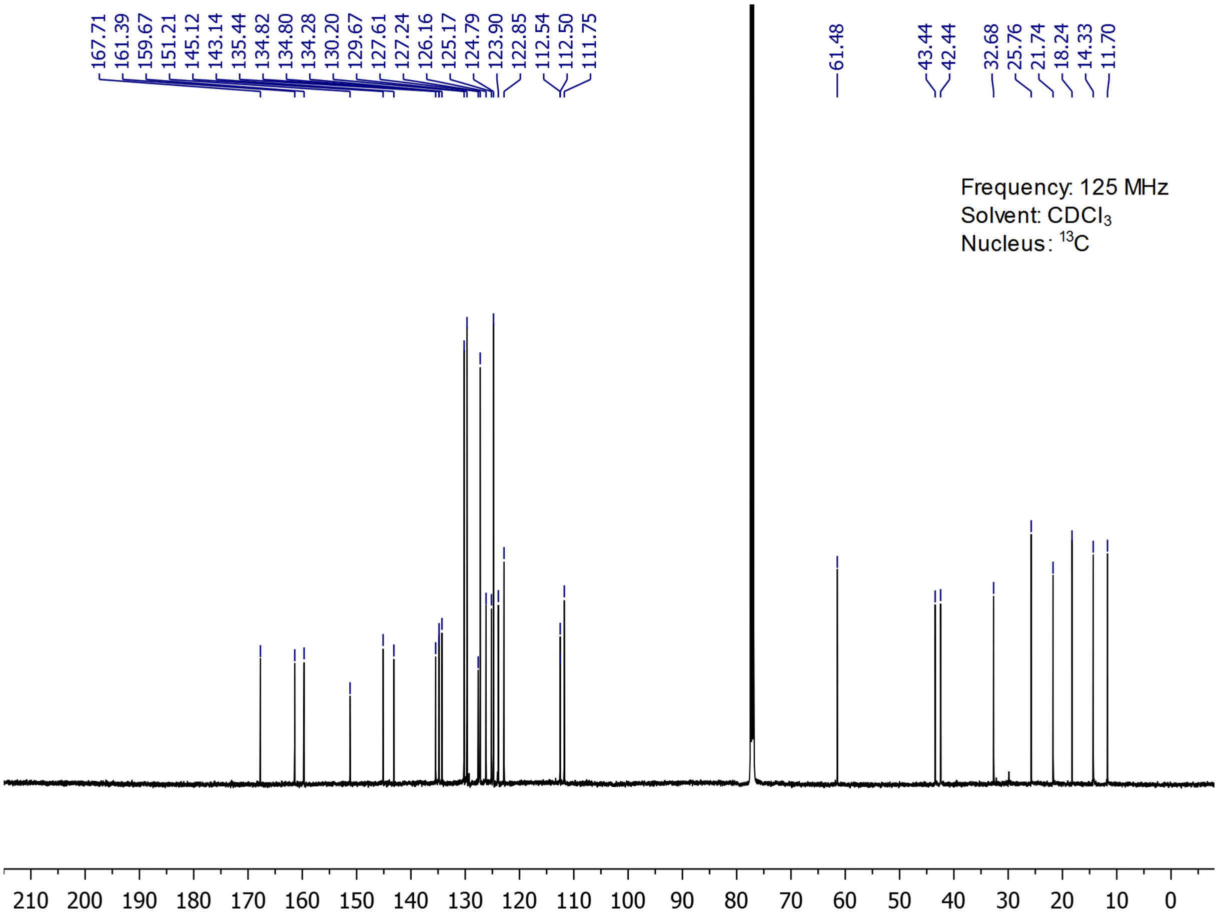
167.71
161.39
159.67
151.21
145.12
143.14
135.44
134.82
134.80
134.28
130.20
129.67
127.61
127.24
126.16
125.17
124.79
123.90
122.85
112.54
112.50
111.75
61.48
43.44
42.44
32.68
25.76
21.74
18.24
14.33
11.70
Frequency: 125 MHz
Solvent: CDCl3
Nucleus: 13C
210 200 190 180 170 160 150 140 130 120 110 100 90 80 70 60 50 40 30 20 10 0

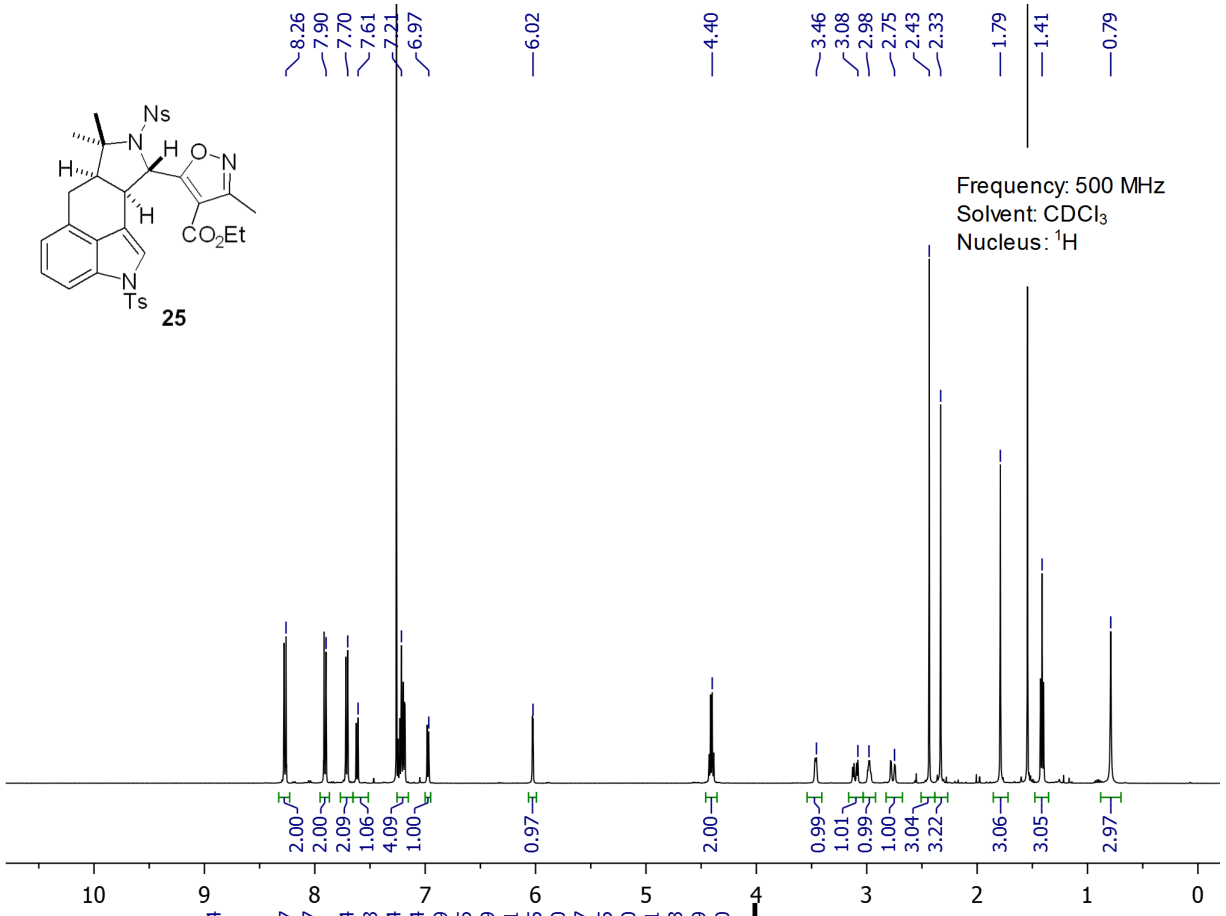
Ns
H
O
N
H
H
CO2Et
N
Ts
25
8.26
7.90
7.70
7.61
7.21
6.97
6.02
4.40
3.46
3.08
2.98
2.75
2.43
2.33
1.79
1.41
0.79
Frequency: 500 MHz
Solvent: CDCl3
Nucleus: 1H
2.00
2.00
2.09
1.06
4.09
1.00
0.97
2.00
0.99
1.01
0.99
1.00
3.04
3.22
3.06
3.05
2.97
10 9 8 7 6 5 4 3 2 1 0

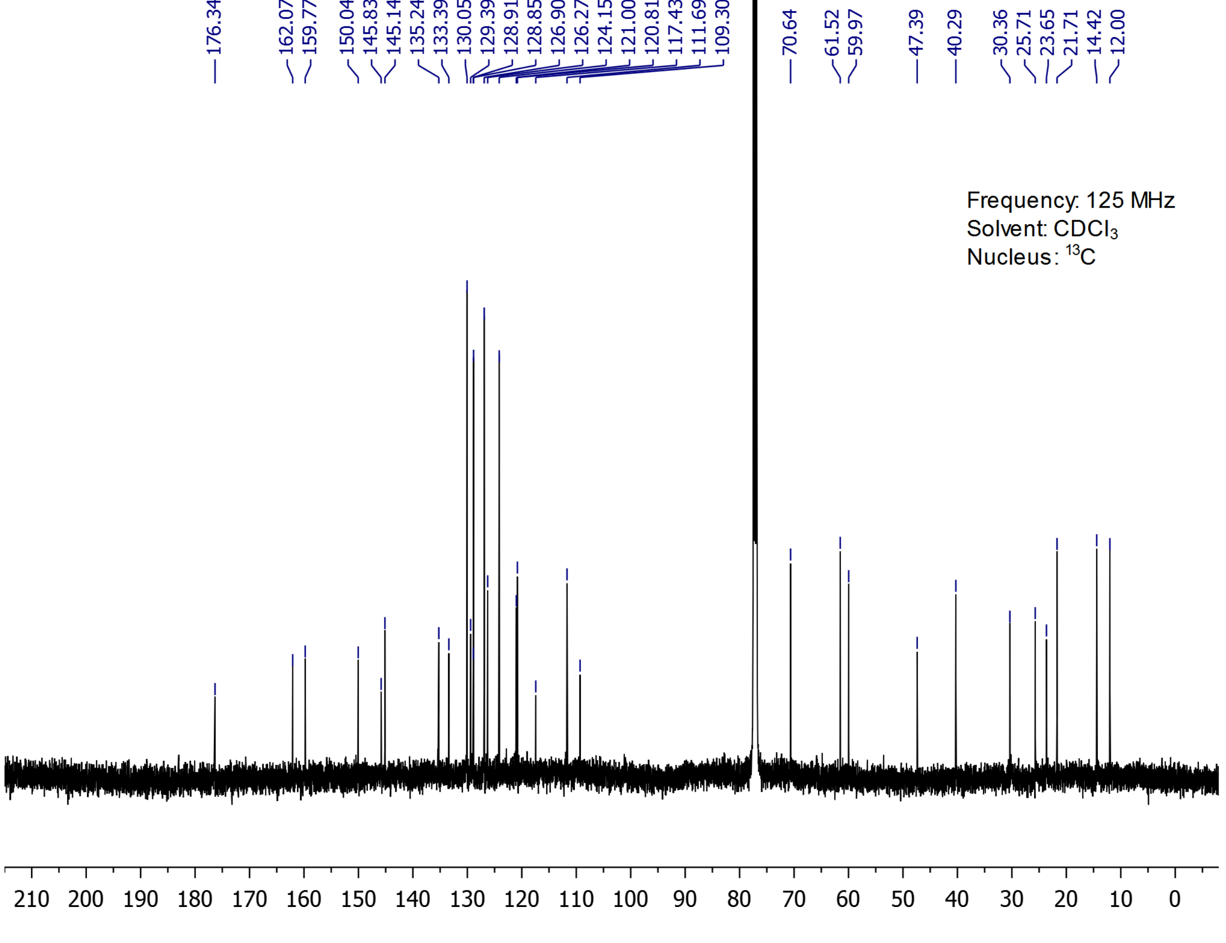
176.34
162.07
159.77
150.04
145.83
145.14
135.24
133.39
130.05
129.39
128.91
128.85
126.90
126.27
124.15
121.00
120.81
117.43
111.69
109.30
70.64
61.52
59.97
47.39
40.29
30.36
25.71
23.65
21.71
14.42
12.00
Frequency: 125 MHz
Solvent: CDCl3
Nucleus: 13C
210 200 190 180 170 160 150 140 130 120 110 100 90 80 70 60 50 40 30 20 10 0

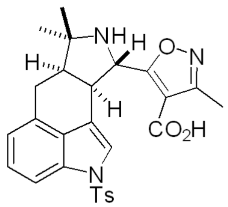

26

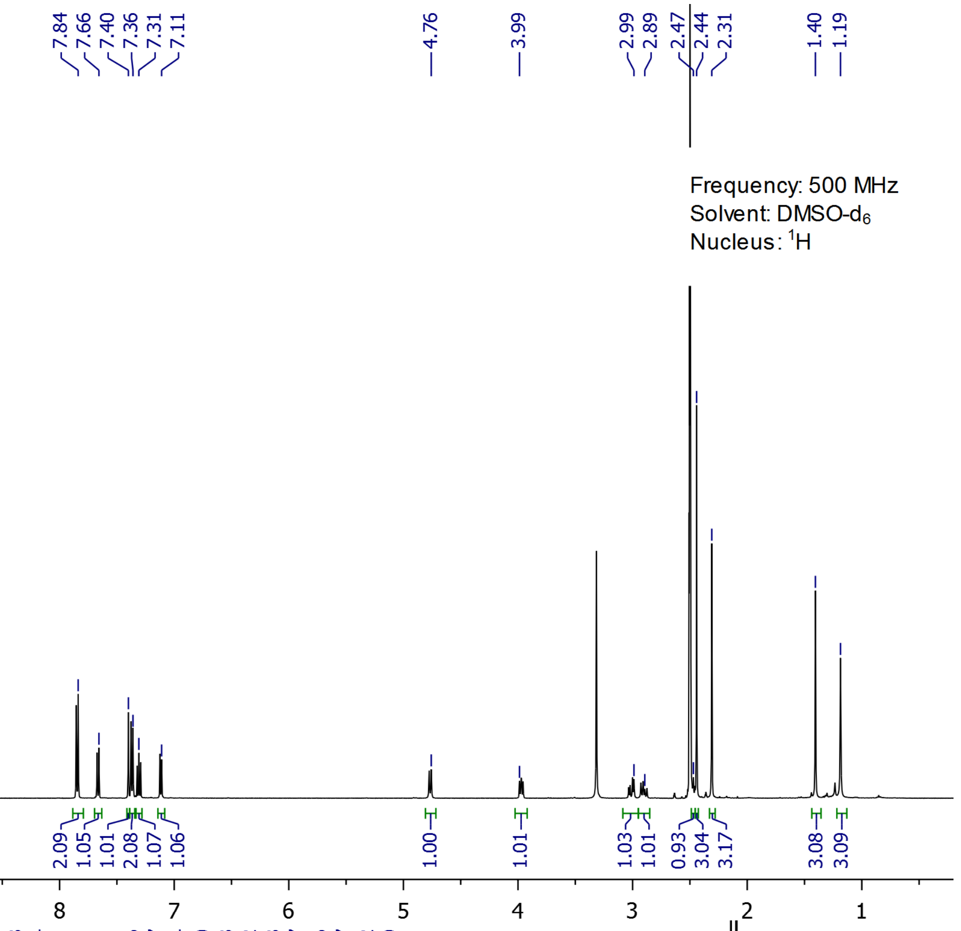
7.84
7.66
7.40
7.36
7.31
7.11
4.76
3.99
2.99
2.89
2.47
2.44
2.31
1.40
1.19
Frequency: 500 MHz
Solvent: DMSO-d6
Nucleus: 1H
2.09
1.05
1.01
2.08
1.07
1.06
1.00
1.01
1.03
1.01
0.93
3.04
3.17
3.08
3.09
10
9
8
7
6
5
4
3
2
1

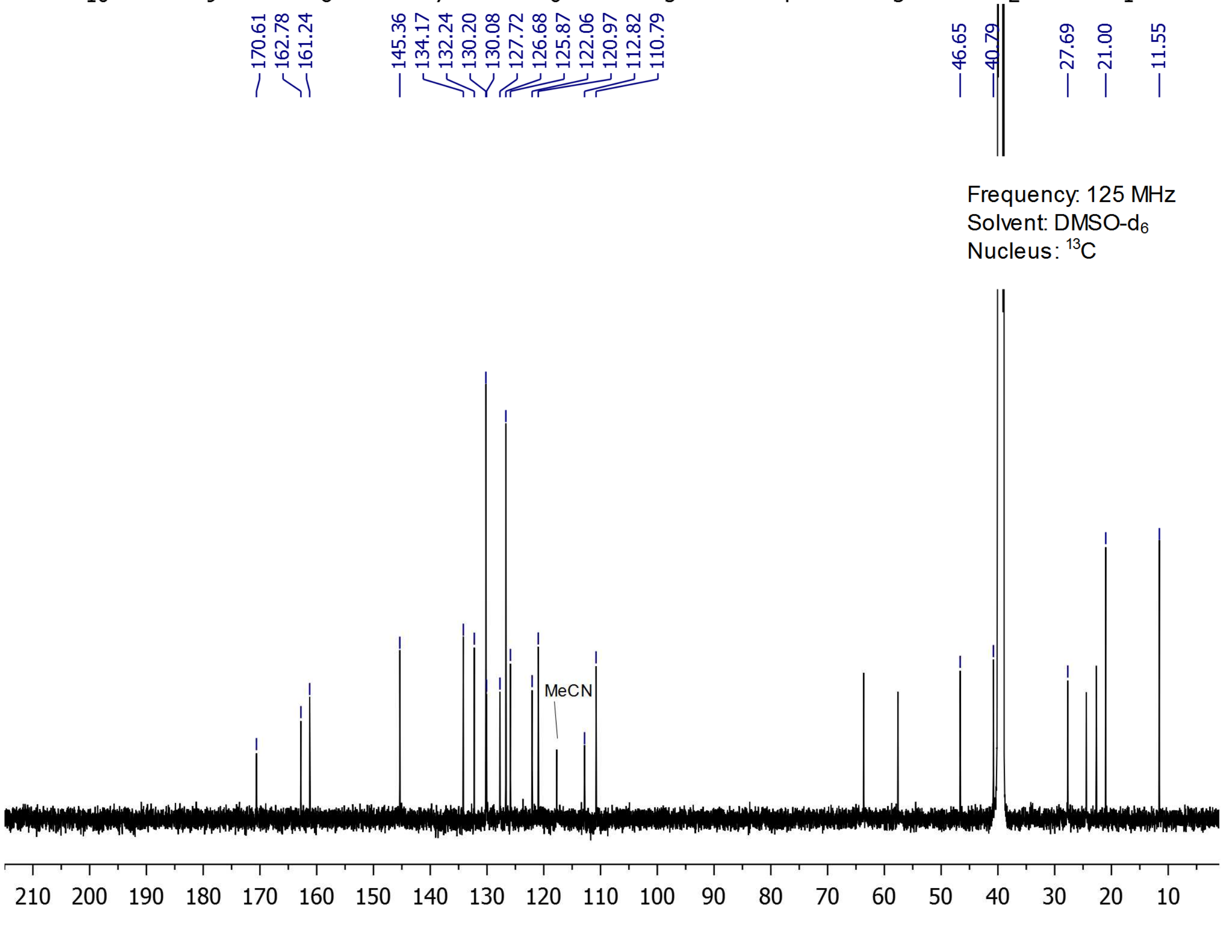
170.61
162.78
161.24
145.36
134.17
132.24
130.20
130.08
127.72
126.68
125.87
122.06
120.97
112.82
110.79
46.65
40.79
27.69
21.00
11.55
Frequency: 125 MHz
Solvent: DMSO-d6
Nucleus: 13C
MeCN
210
200
190
180
170
160
150
140
130
120
110
100
90
80
70
60
50
40
30
20
10

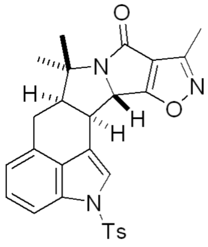
O
N
H
H
H
O
N
N
Ts

27

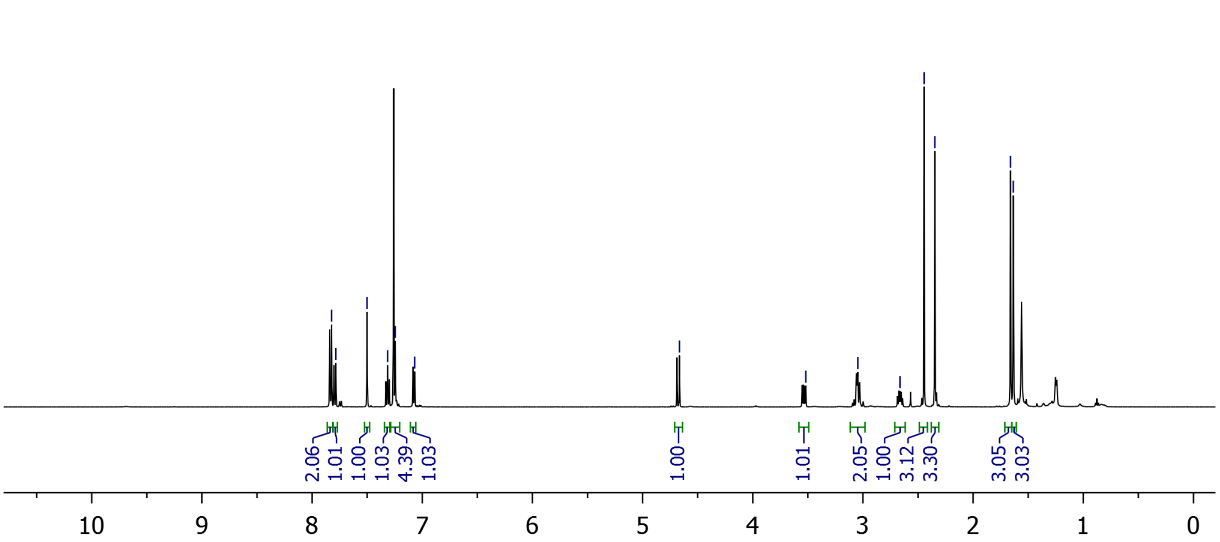
7.82
7.78
7.50
7.31
7.25
7.07
4.67
3.52
3.05
2.66
2.45
2.35
1.66
1.64
Frequency: 500 MHz
Solvent: $CDCl_3$
Nucleus: ^1H
2.06
1.01
1.00
1.03
4.39
1.03
1.00
1.01
2.05
1.00
3.12
3.30
3.05
3.03
10 9 8 7 6 5 4 3 2 1 0

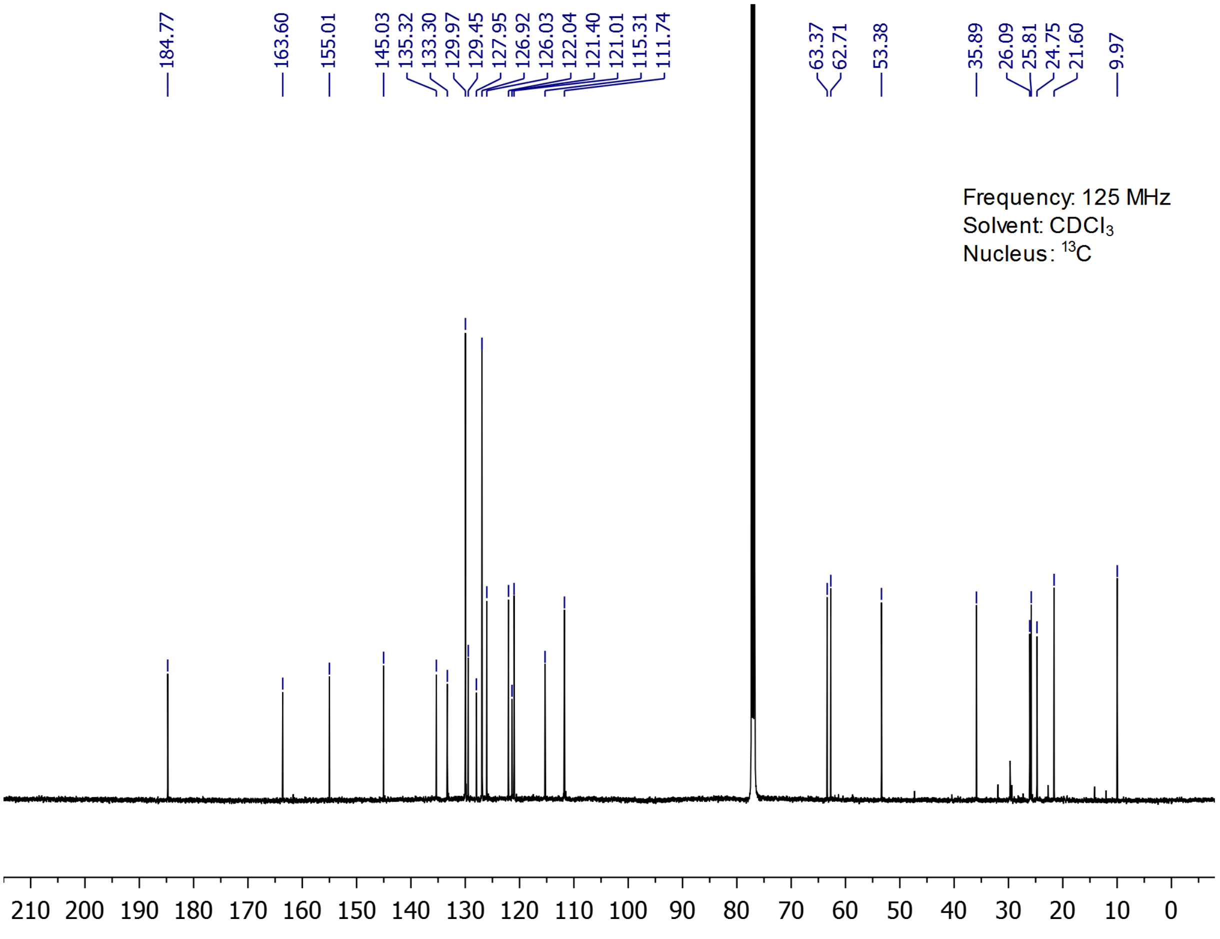
184.77
163.60
155.01
145.03
135.32
133.30
129.97
129.45
127.95
126.92
126.03
122.04
121.40
121.01
115.31
111.74
63.37
62.71
53.38
35.89
26.09
25.81
24.75
21.60
9.97
Frequency: 125 MHz
Solvent: $CDCl_3$
Nucleus: ^{13}C
210 200 190 180 170 160 150 140 130 120 110 100 90 80 70 60 50 40 30 20 10 0

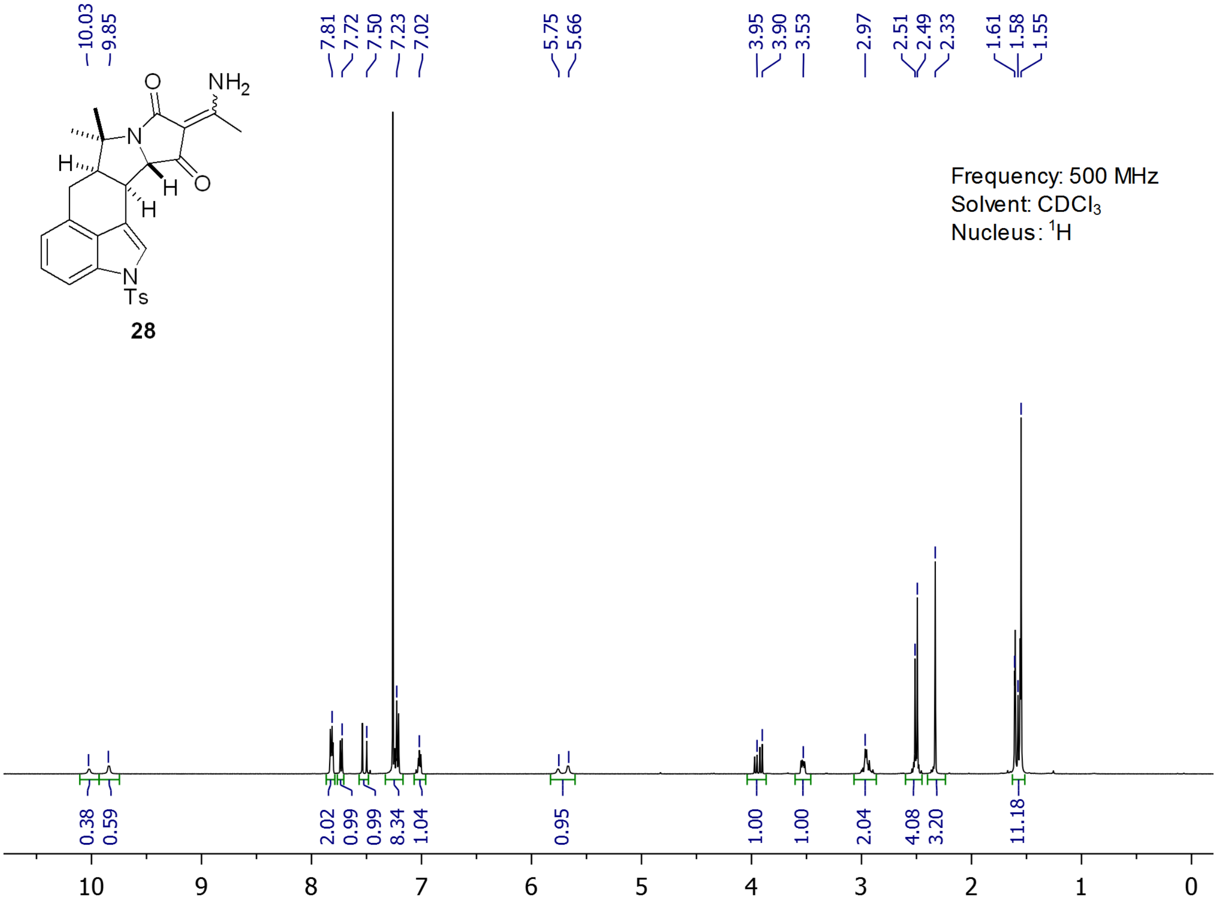

O
NH2
N
H
H
O
H
N
Ts
28
10.03
9.85
7.81
7.72
7.50
7.23
7.02
5.75
5.66
3.95
3.90
3.53
2.97
2.51
2.49
2.33
1.61
1.58
1.55
Frequency: 500 MHz
Solvent: CDCl3
Nucleus: 1H
0.38
0.59
2.02
0.99
0.99
8.34
1.04
0.95
1.00
1.00
2.04
4.08
3.20
11.18
10 9 8 7 6 5 4 3 2 1 0

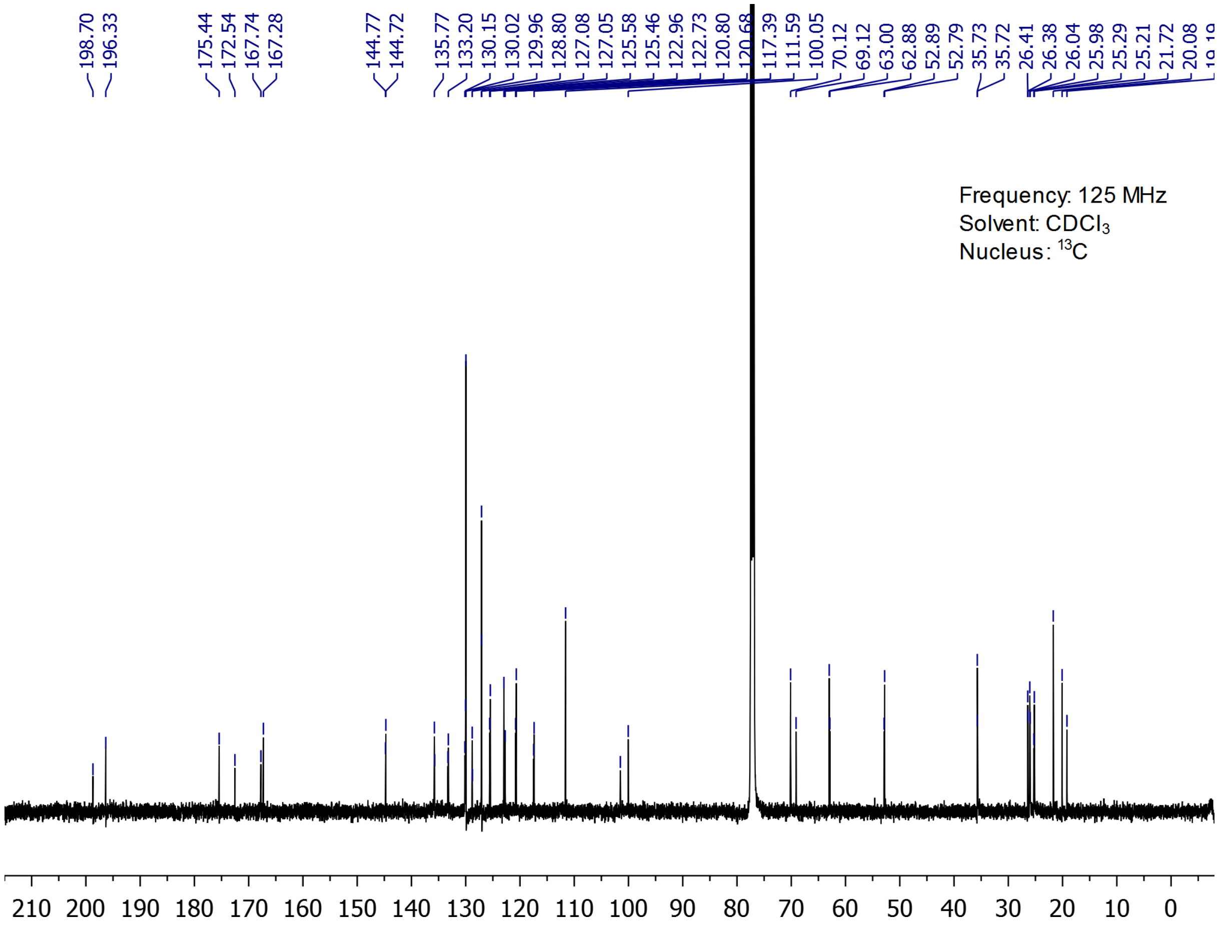

198.70
196.33
175.44
172.54
167.74
167.28
144.77
144.72
135.77
133.20
130.15
130.02
129.96
128.80
127.08
127.05
125.58
125.46
122.96
122.73
120.80
120.68
117.39
111.59
100.05
70.12
69.12
63.00
62.88
52.89
52.79
35.73
35.72
26.41
26.38
26.04
25.98
25.29
25.21
21.72
20.08
Frequency: 125 MHz
Solvent: CDCl3
Nucleus: 13C
210 200 190 180 170 160 150 140 130 120 110 100 90 80 70 60 50 40 30 20 10 0

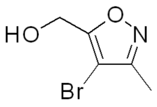
HO
O
N
Br

29

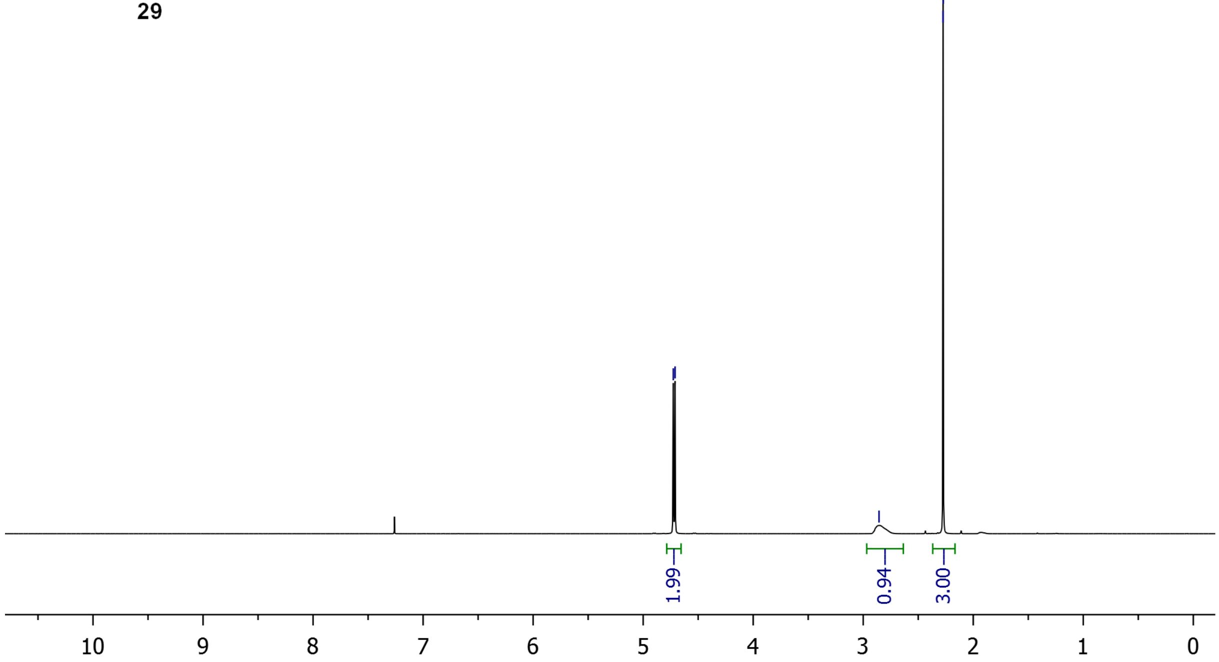
4.73
4.71
2.86
2.27
2.27
Frequency: 400 MHz
Solvent: CDCl3
Nucleus: 1H
1.99
0.94
3.00
10
9
8
7
6
5
4
3
2
1
0

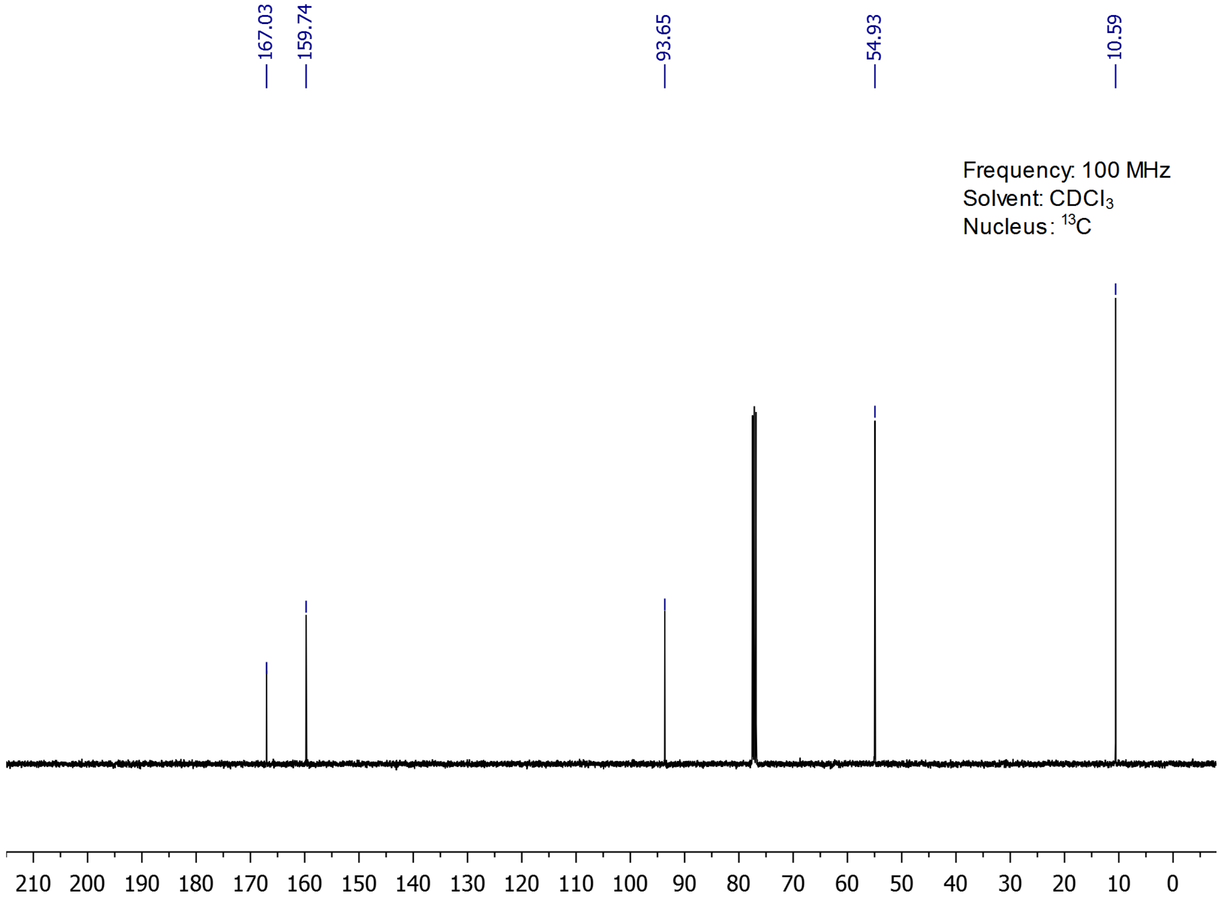
167.03
159.74
93.65
54.93
10.59
Frequency: 100 MHz
Solvent: CDCl3
Nucleus: 13C
210
200
190
180
170
160
150
140
130
120
110
100
90
80
70
60
50
40
30
20
10
0

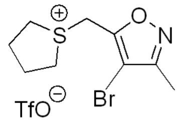
TfO
Br
O
N
S

31a

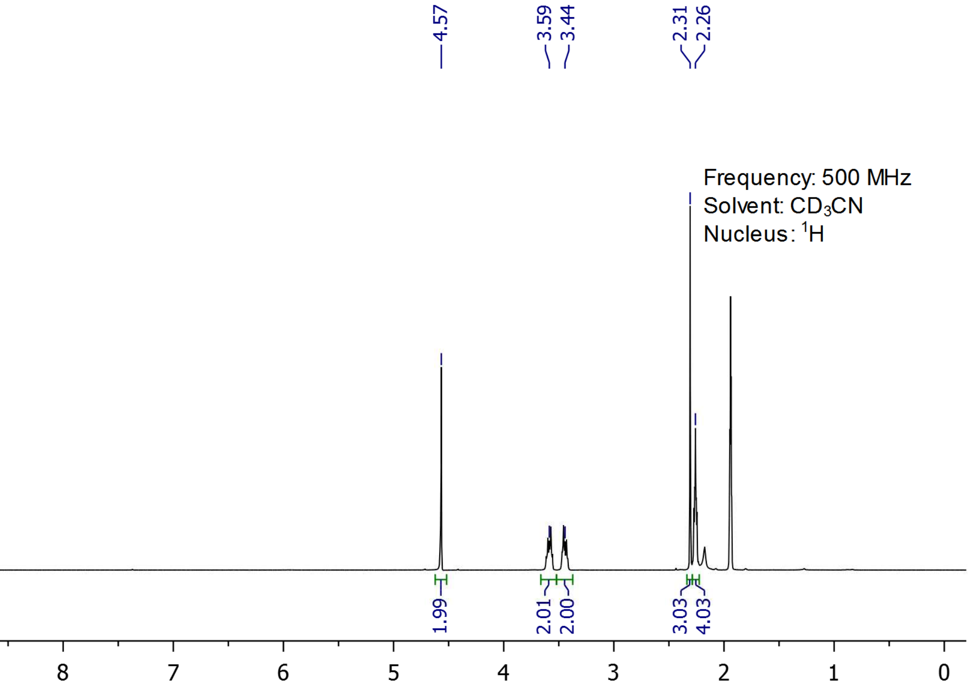
4.57
3.59
3.44
2.31
2.26
Frequency: 500 MHz
Solvent: CD_3CN
Nucleus: ^1H
1.99
2.01
2.00
3.03
4.03
10 9 8 7 6 5 4 3 2 1 0

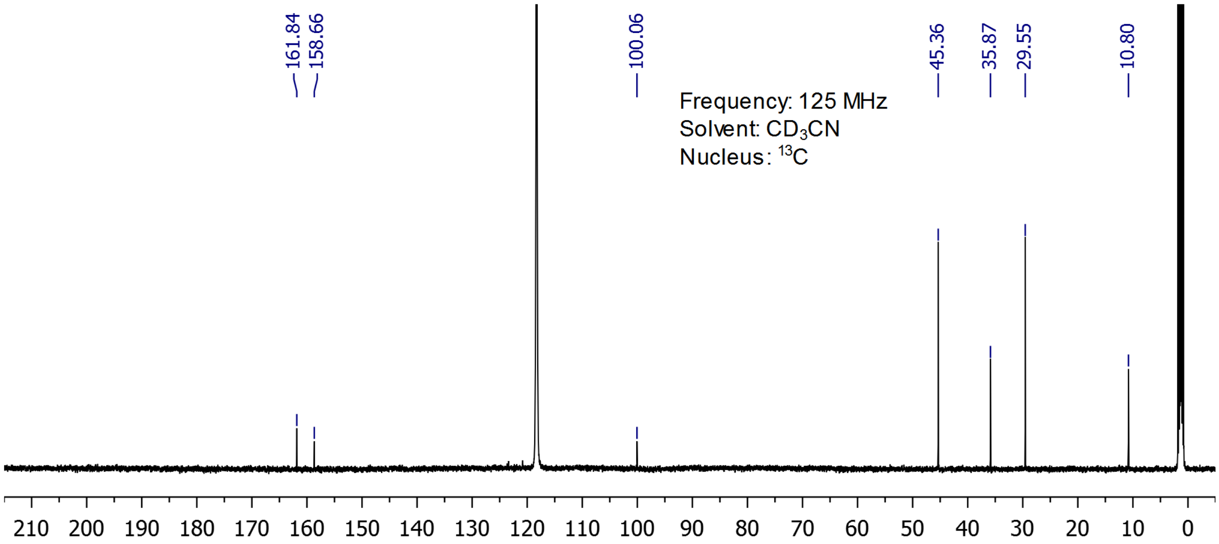
161.84
158.66
100.06
45.36
35.87
29.55
10.80
Frequency: 125 MHz
Solvent: CD_3CN
Nucleus: ^{13}C
210 200 190 180 170 160 150 140 130 120 110 100 90 80 70 60 50 40 30 20 10 0

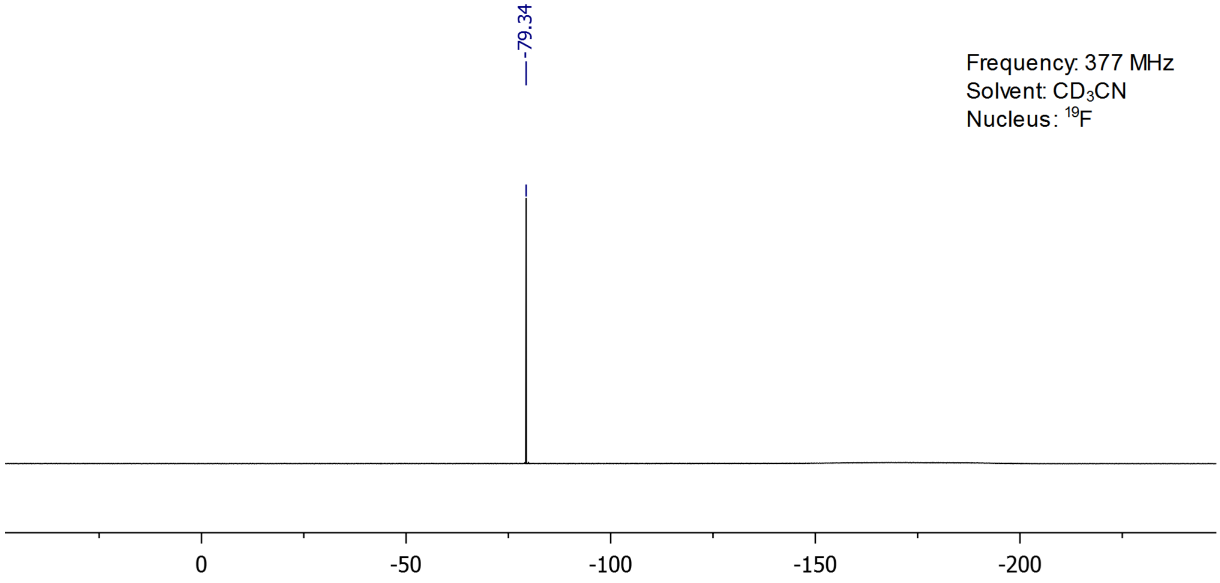
-79.34
Frequency: 377 MHz
Solvent: CD_3CN
Nucleus: ^{19}F
0 -50 -100 -150 -200

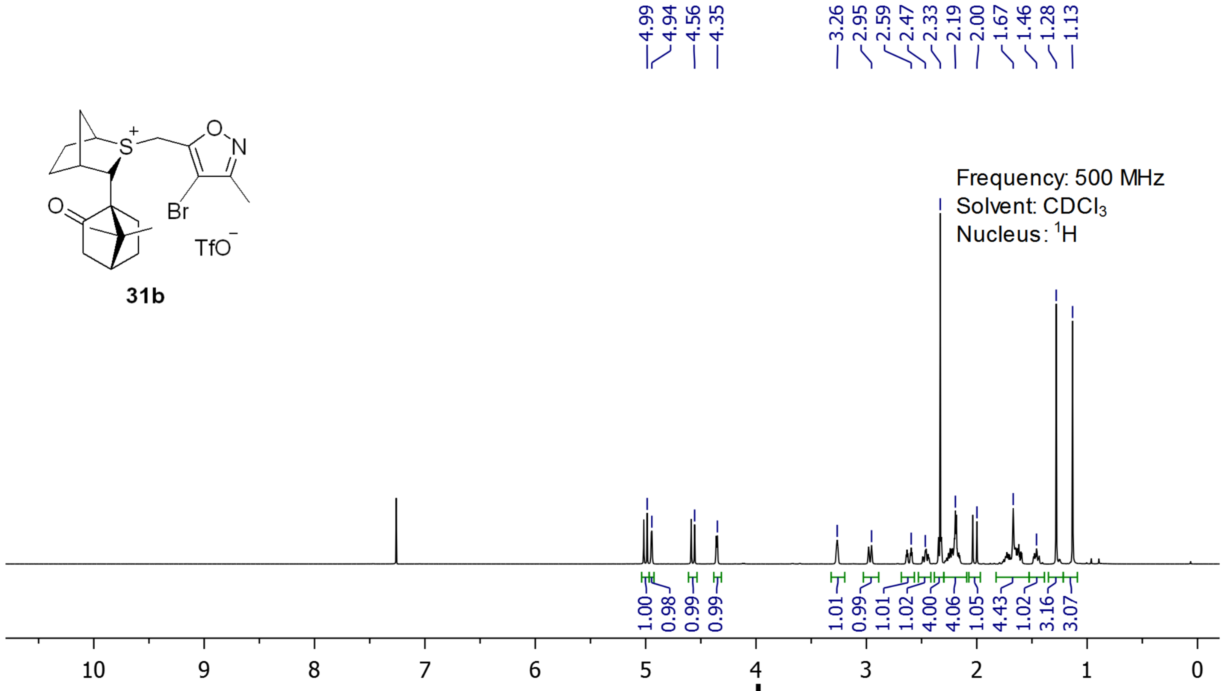
Br
TfO−
31b
4.99
4.94
4.56
4.35
3.26
2.95
2.59
2.47
2.33
2.19
2.00
1.67
1.46
1.28
1.13
Frequency: 500 MHz
Solvent: CDCl3
Nucleus: 1H
1.00
0.98
0.99
0.99
1.01
0.99
1.01
1.02
4.00
4.06
1.05
4.43
1.02
3.16
3.07

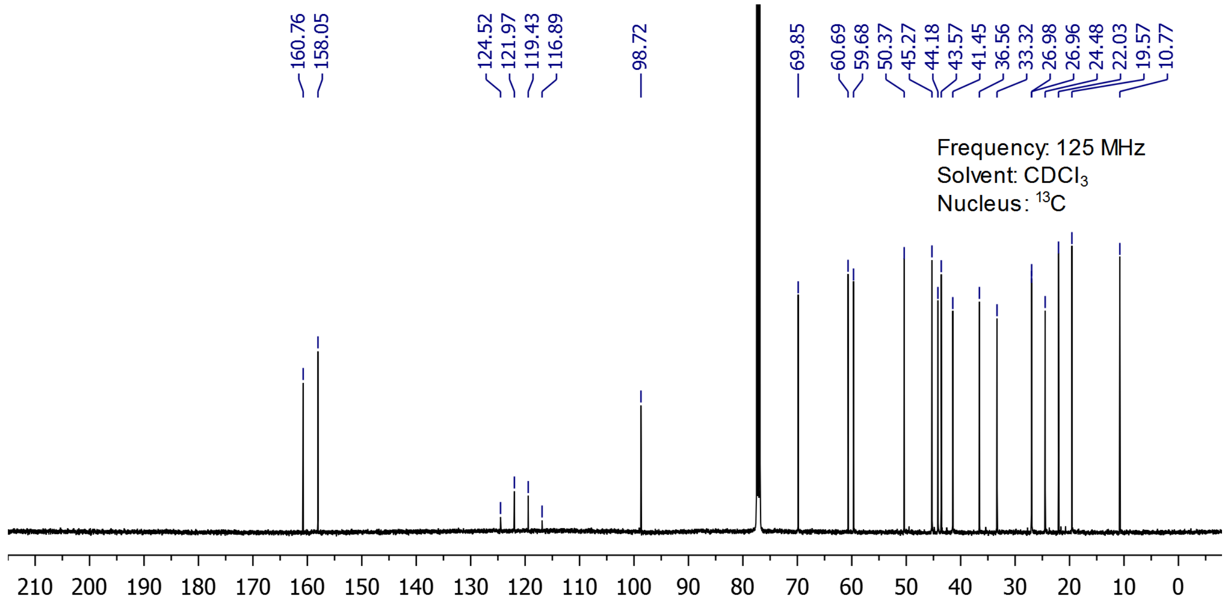
160.76
158.05
124.52
121.97
119.43
116.89
98.72
69.85
60.69
59.68
50.37
45.27
44.18
43.57
41.45
36.56
33.32
26.98
26.96
24.48
22.03
19.57
10.77
Frequency: 125 MHz
Solvent: CDCl3
Nucleus: 13C

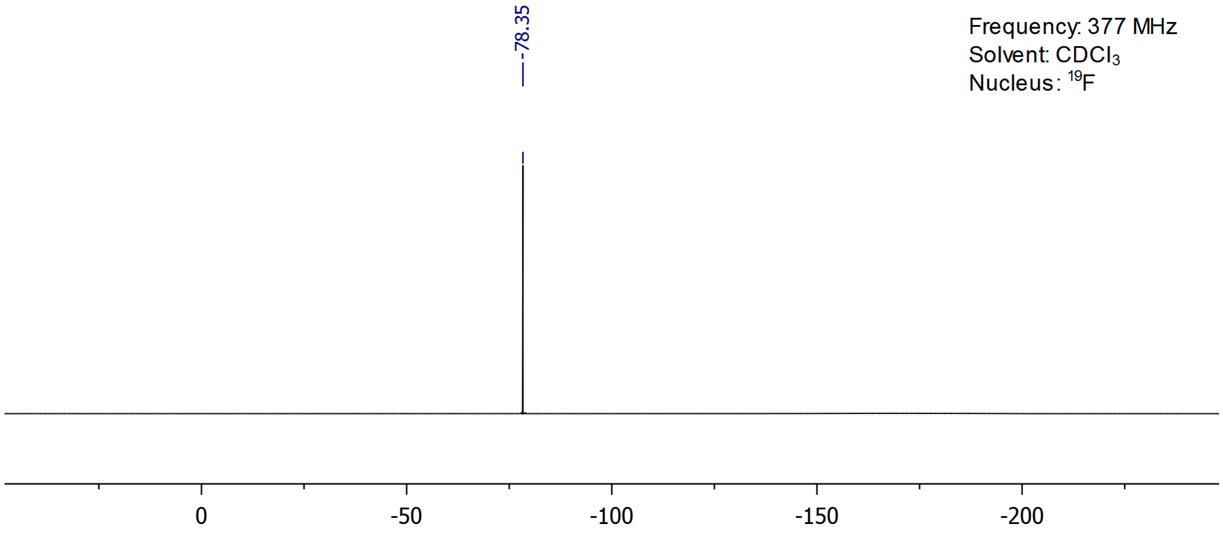
-78.35
Frequency: 377 MHz
Solvent: CDCl3
Nucleus: 19F

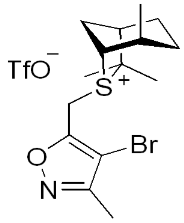
TfO⁻
S+
Br
O
N

31c

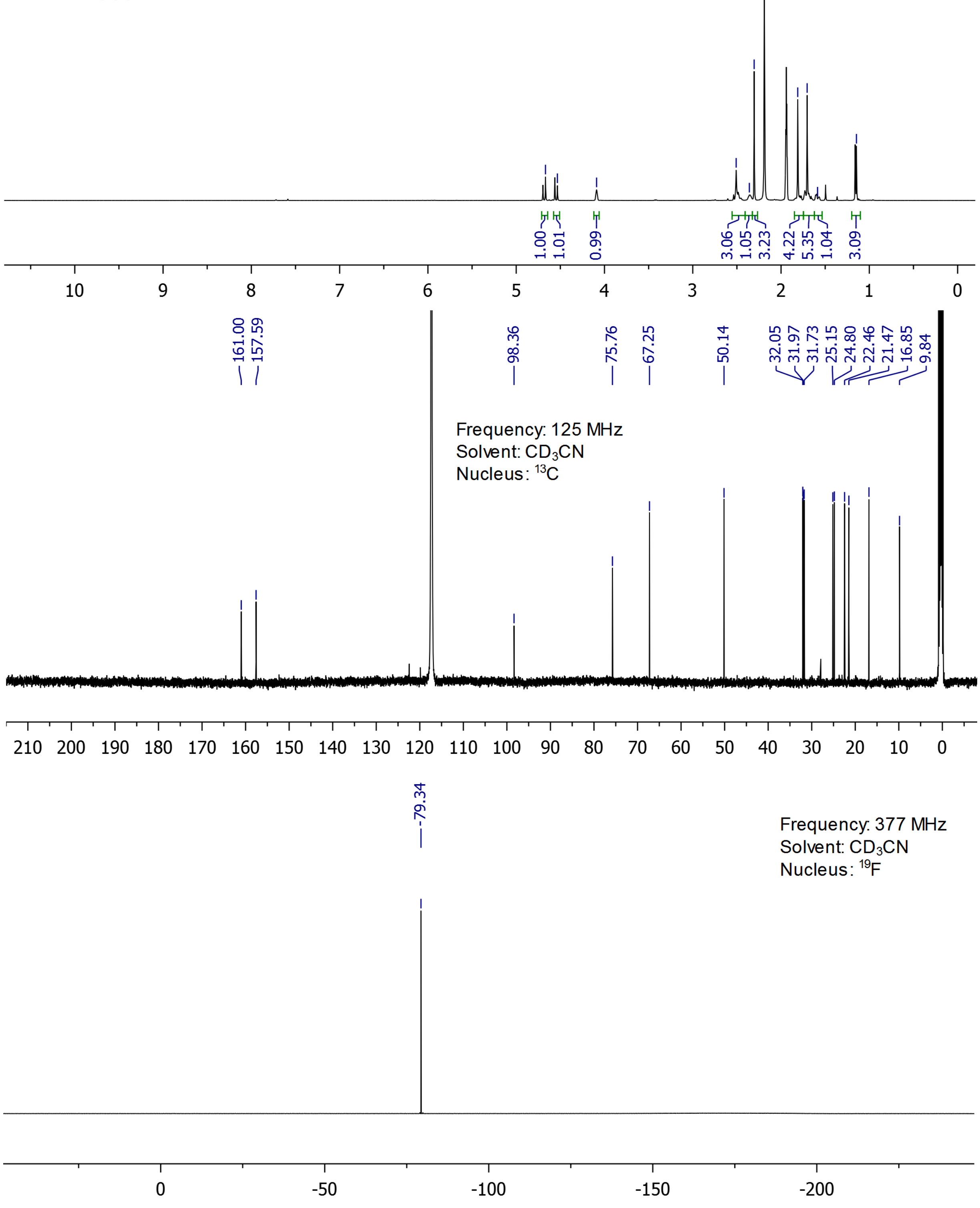
4.67
4.53
4.09
2.51
2.36
2.30
2.19
1.81
1.70
1.59
1.15
Frequency: 500 MHz
Solvent: CD3CN
Nucleus: 1H
1.00
1.01
0.99
3.06
1.05
3.23
4.22
5.35
1.04
3.09
161.00
157.59
98.36
75.76
67.25
50.14
32.05
31.97
31.73
25.15
24.80
22.46
21.47
16.85
9.84
Frequency: 125 MHz
Solvent: CD3CN
Nucleus: 13C
-79.34
Frequency: 377 MHz
Solvent: CD3CN
Nucleus: 19F

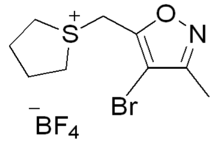

S
O
N
Br
$^{-}BF_4$

31d

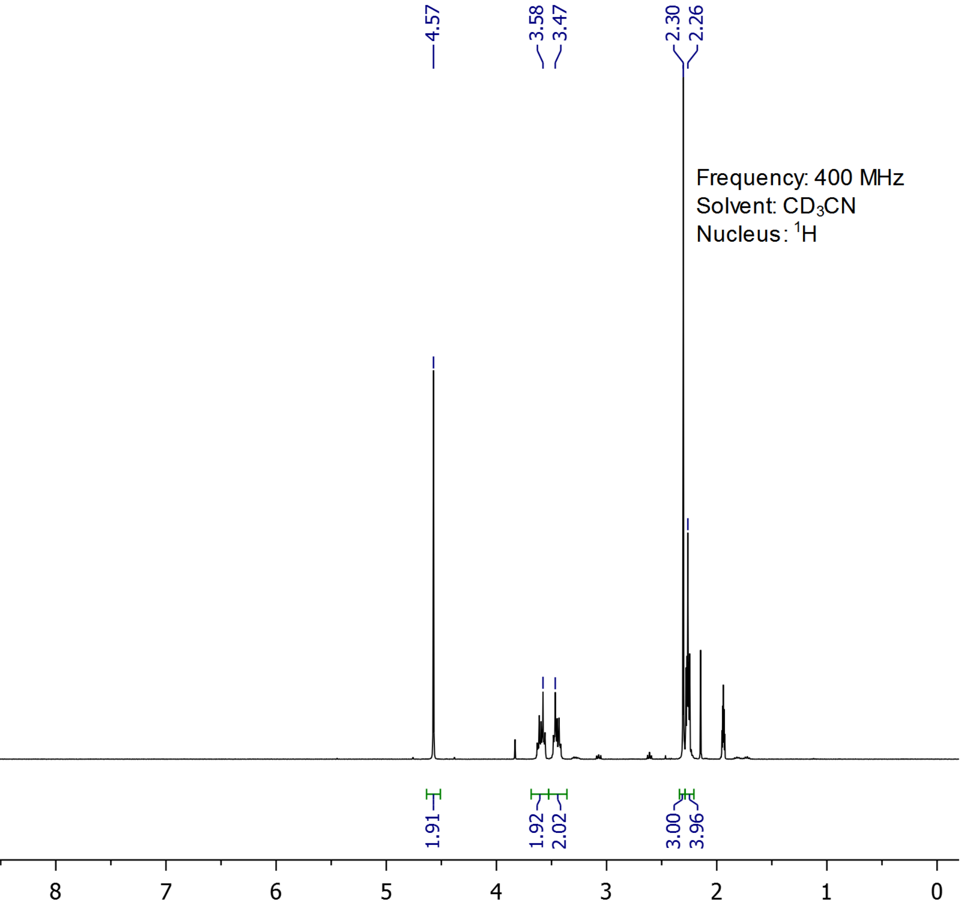

4.57
3.58
3.47
2.30
2.26
Frequency: 400 MHz
Solvent: CD_3CN
Nucleus: ^1H
1.91
1.92
2.02
3.00
3.96
10 9 8 7 6 5 4 3 2 1 0

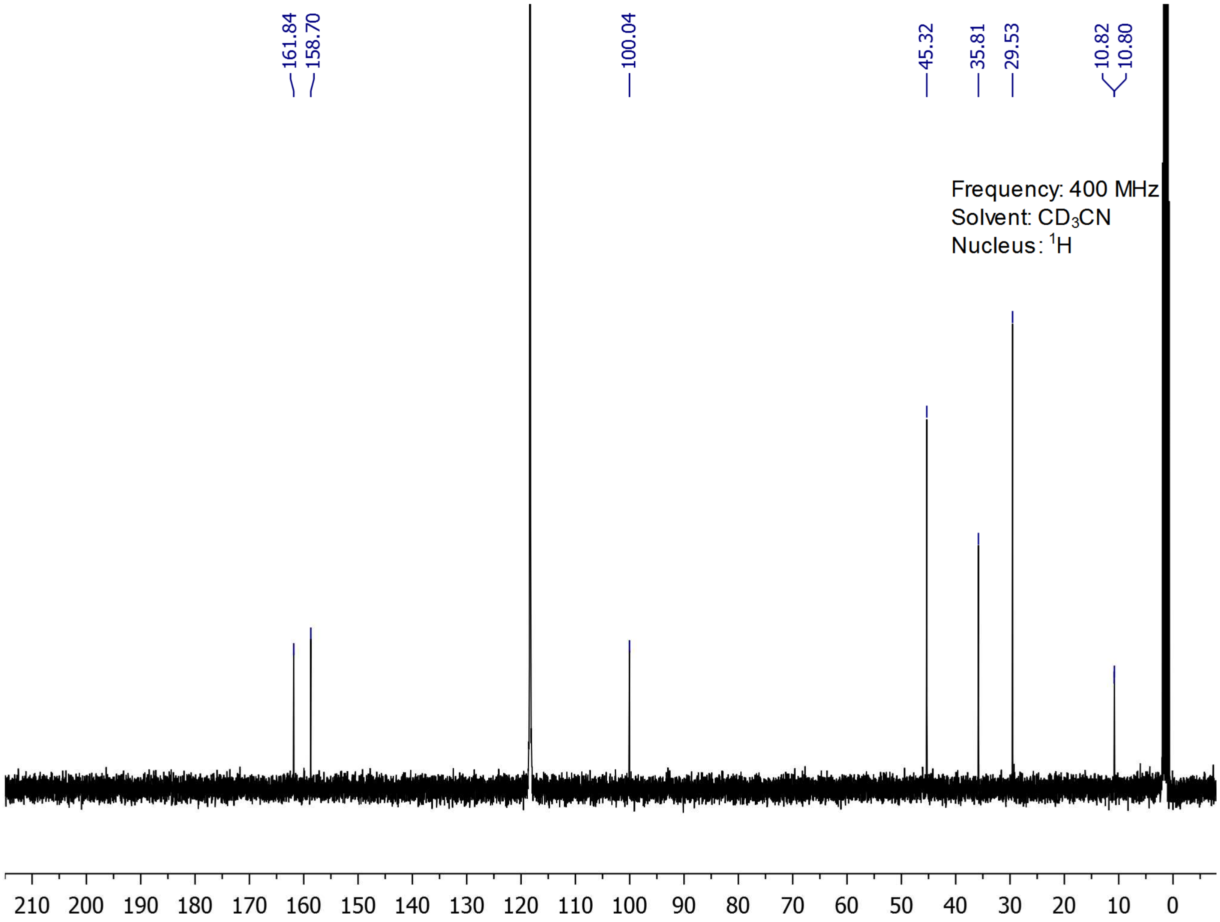

161.84
158.70
100.04
45.32
35.81
29.53
10.82
10.80
Frequency: 400 MHz
Solvent: CD_3CN
Nucleus: ^1H
210 200 190 180 170 160 150 140 130 120 110 100 90 80 70 60 50 40 30 20 10 0

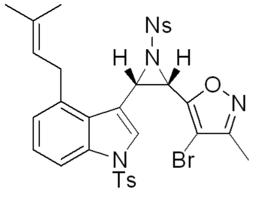
Ns
H N H
O
N
N
Ts
Br

***cis*-32**

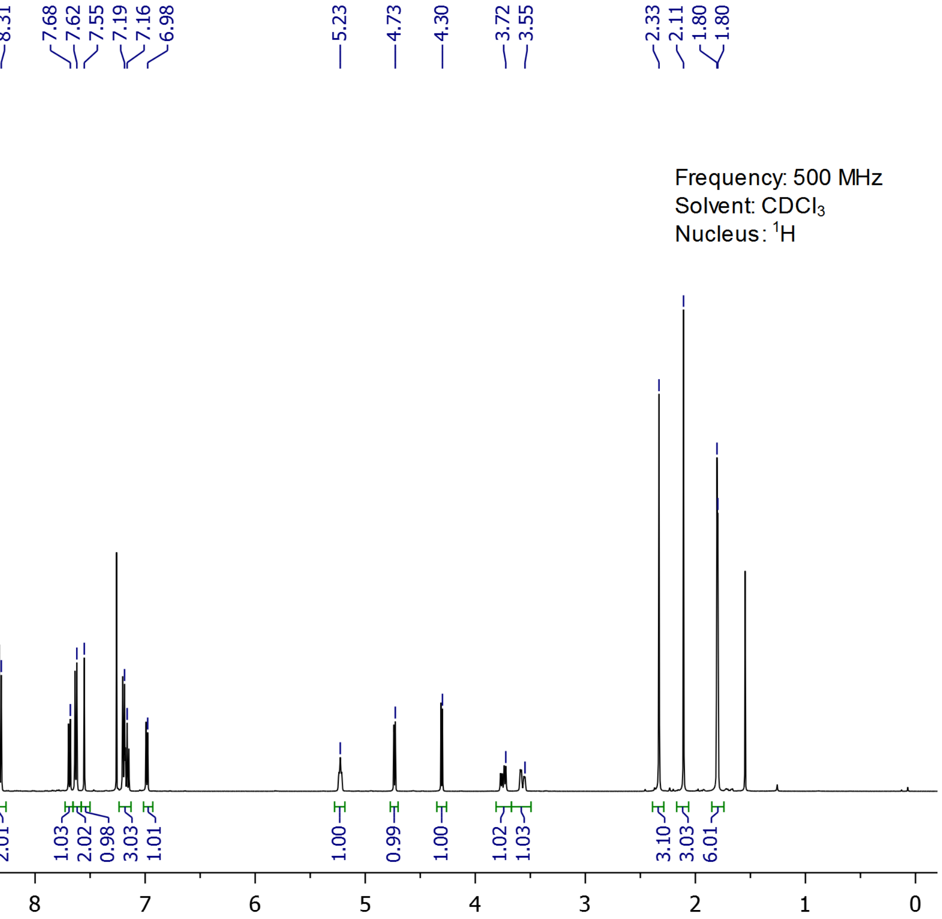
8.46
8.31
7.68
7.62
7.55
7.19
7.16
6.98
5.23
4.73
4.30
3.72
3.55
2.33
2.11
1.80
1.80
Frequency: 500 MHz
Solvent: CDCl3
Nucleus: 1H
2.00
2.01
1.03
2.02
0.98
3.03
1.01
1.00
0.99
1.00
1.02
1.03
3.10
3.03
6.01
10 9 8 7 6 5 4 3 2 1 0

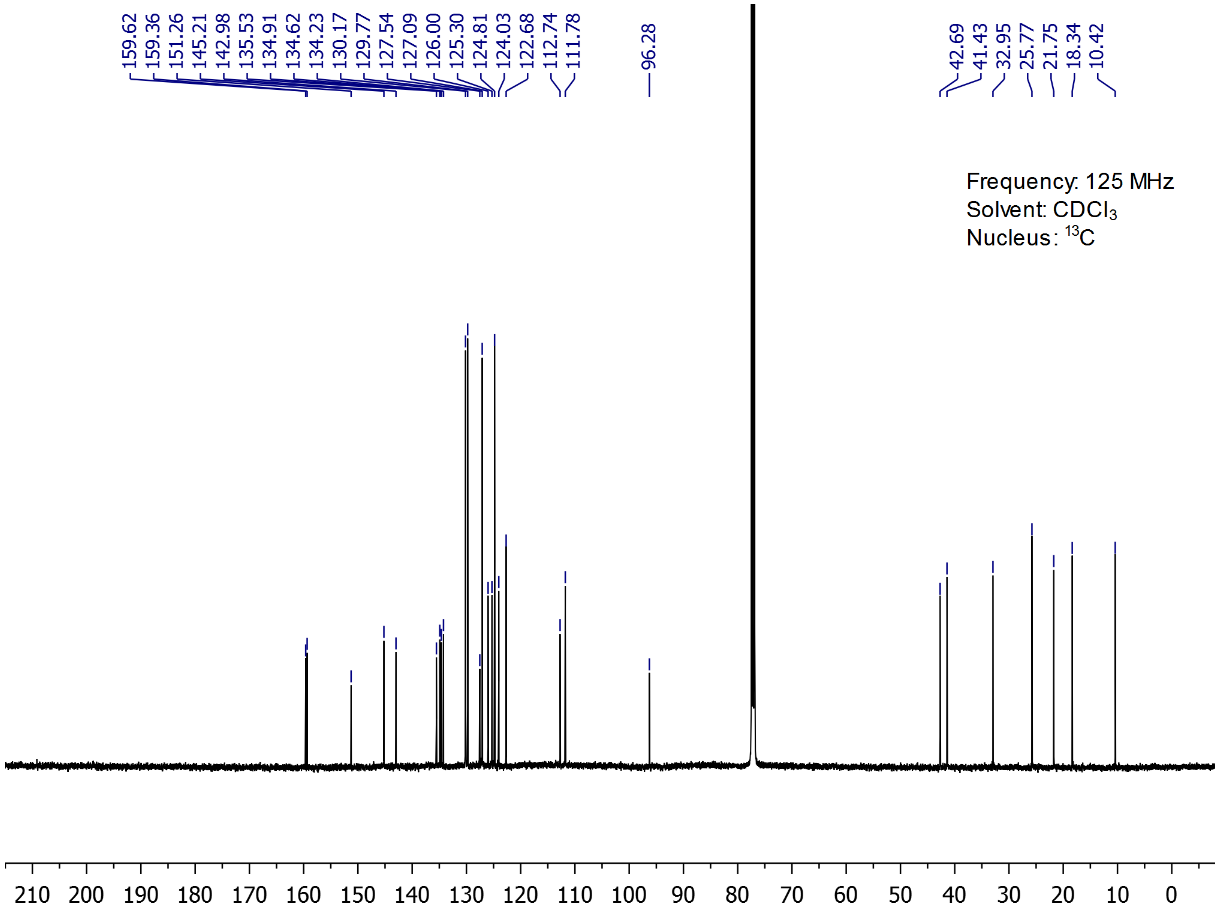
159.62
159.36
151.26
145.21
142.98
135.53
134.91
134.62
134.23
130.17
129.77
127.54
127.09
126.00
125.30
124.81
124.03
122.68
112.74
111.78
96.28
42.69
41.43
32.95
25.77
21.75
18.34
10.42
Frequency: 125 MHz
Solvent: CDCl3
Nucleus: 13C
210 200 190 180 170 160 150 140 130 120 110 100 90 80 70 60 50 40 30 20 10 0

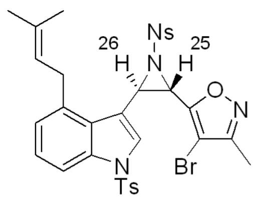

***trans*-32**
trans/cis 3:1

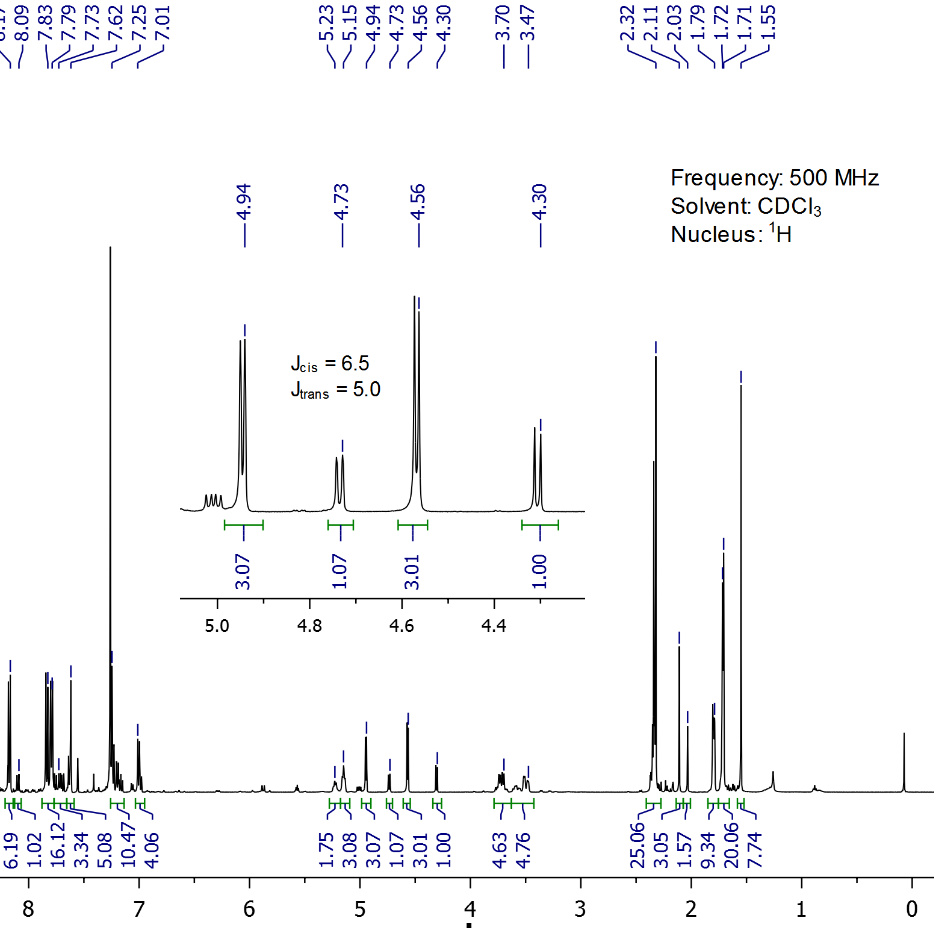

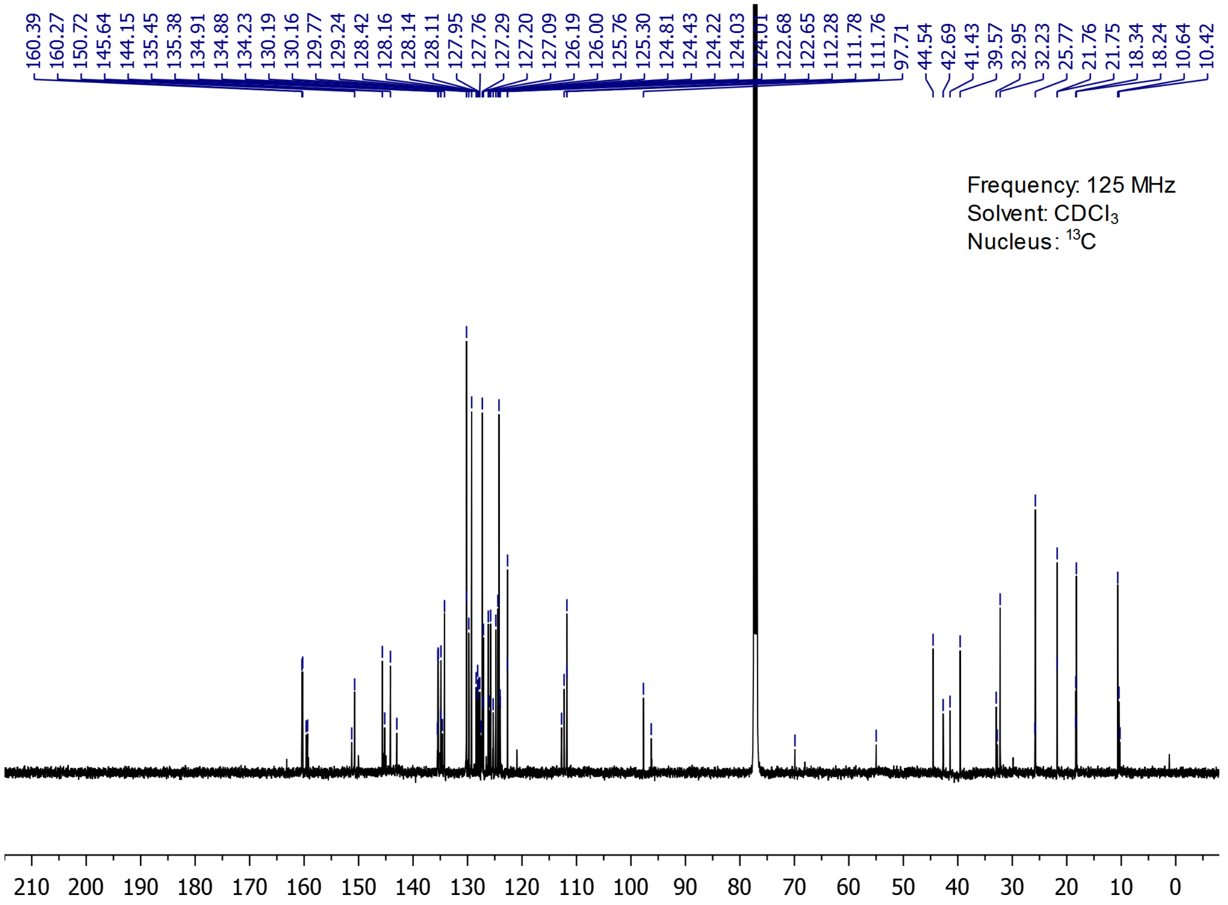

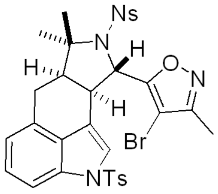
Ns
N
H
H
O
N
H
Br
NTs

33

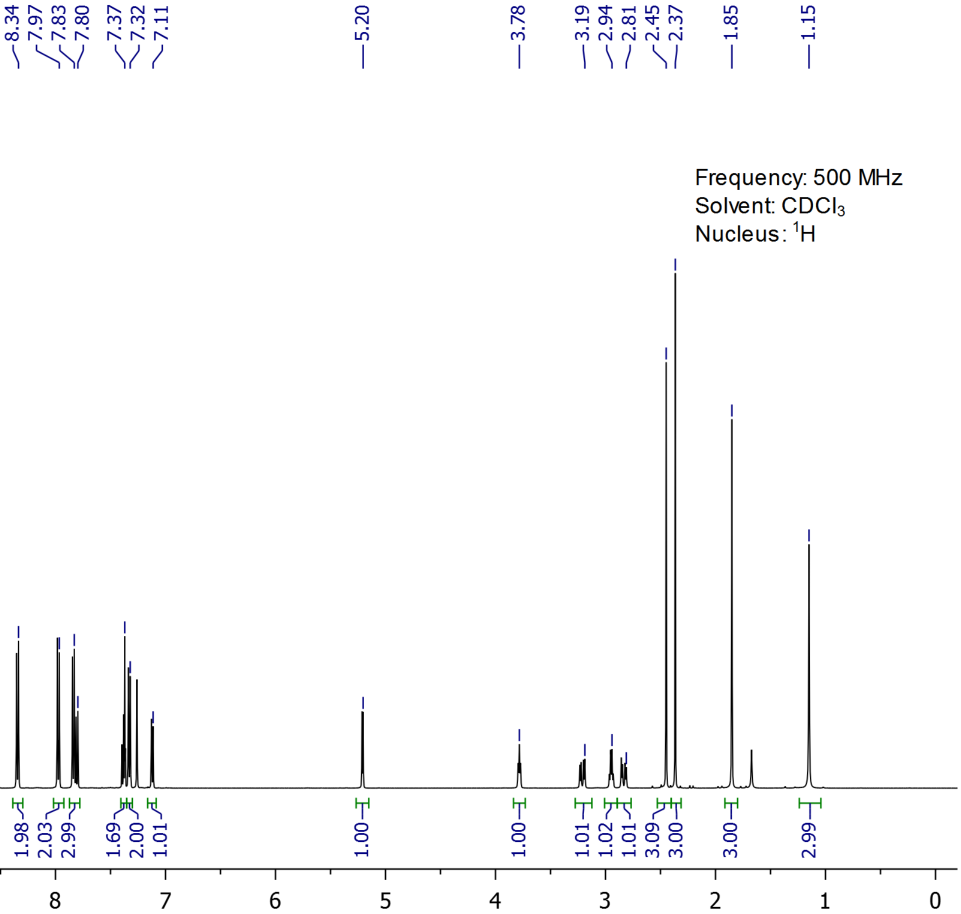
8.34
7.97
7.83
7.80
7.37
7.32
7.11
5.20
3.78
3.19
2.94
2.81
2.45
2.37
1.85
1.15
Frequency: 500 MHz
Solvent: $CDCl_3$
Nucleus: ^1H
1.98
2.03
2.99
1.69
2.00
1.01
1.00
1.00
1.01
1.02
1.01
3.09
3.00
3.00
2.99
10 9 8 7 6 5 4 3 2 1 0

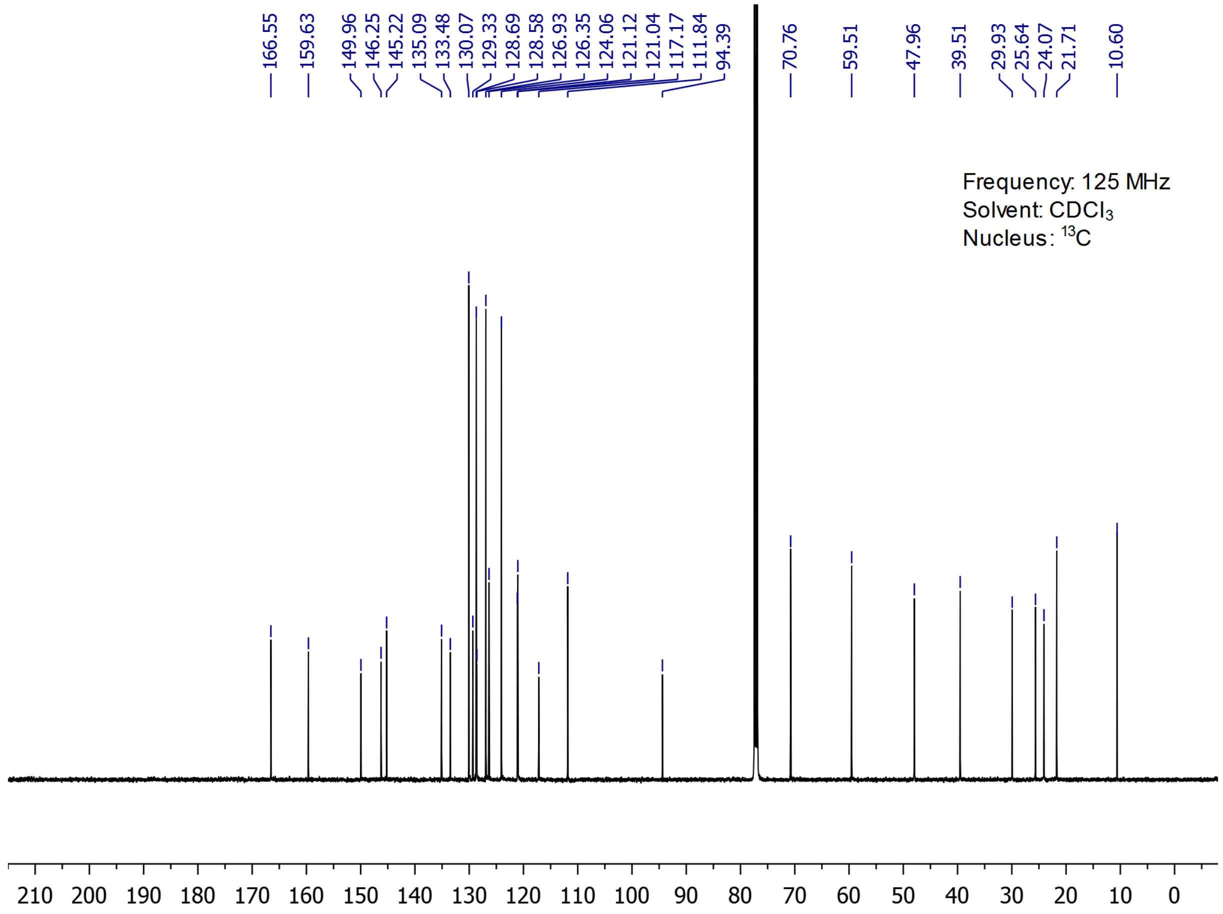
166.55
159.63
149.96
146.25
145.22
135.09
133.48
130.07
129.33
128.69
128.58
126.93
126.35
124.06
121.12
121.04
117.17
111.84
94.39
70.76
59.51
47.96
39.51
29.93
25.64
24.07
21.71
10.60
Frequency: 125 MHz
Solvent: $CDCl_3$
Nucleus: ^{13}C
210 200 190 180 170 160 150 140 130 120 110 100 90 80 70 60 50 40 30 20 10 0

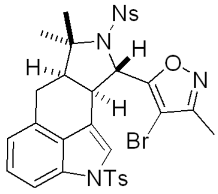
Ns
N
H
H
O
N
H
Br
NTs

iso-**33**

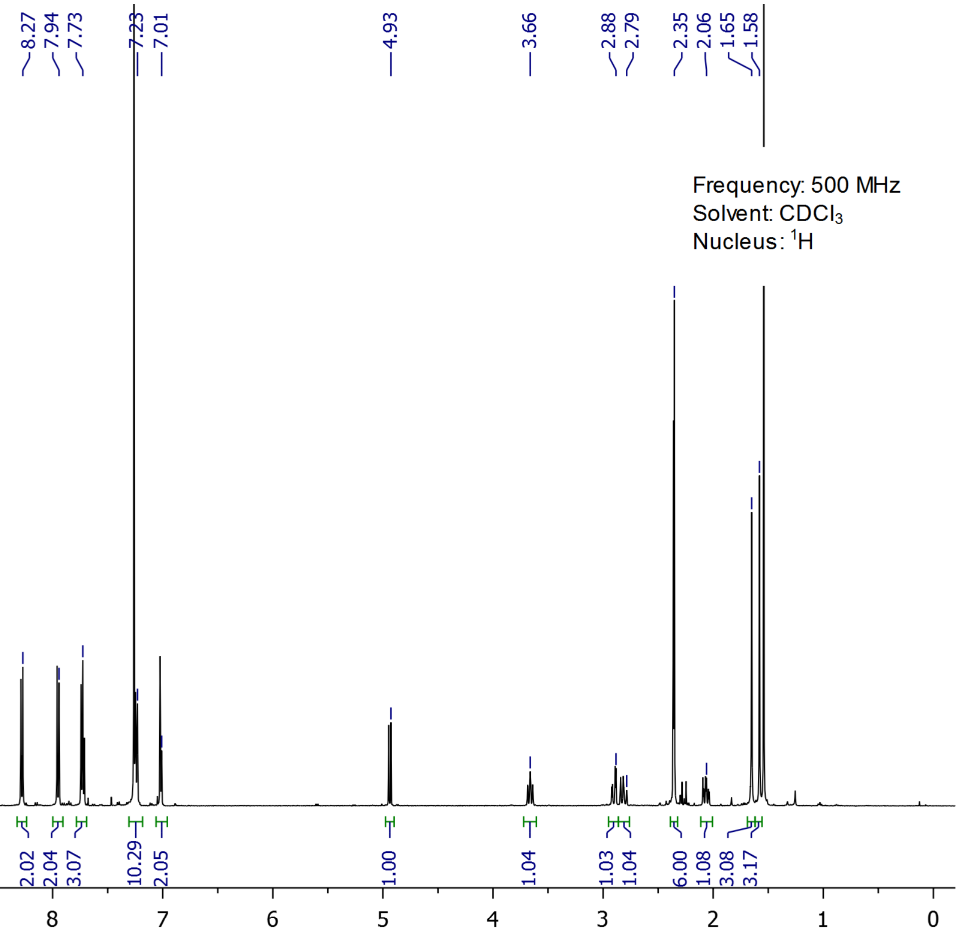
8.27
7.94
7.73
7.23
7.01
4.93
3.66
2.88
2.79
2.35
2.06
1.65
1.58
Frequency: 500 MHz
Solvent: CDCl3
Nucleus: 1H
2.02
2.04
3.07
10.29
2.05
1.00
1.04
1.03
1.04
6.00
1.08
3.08
3.17
10
9
8
7
6
5
4
3
2
1
0

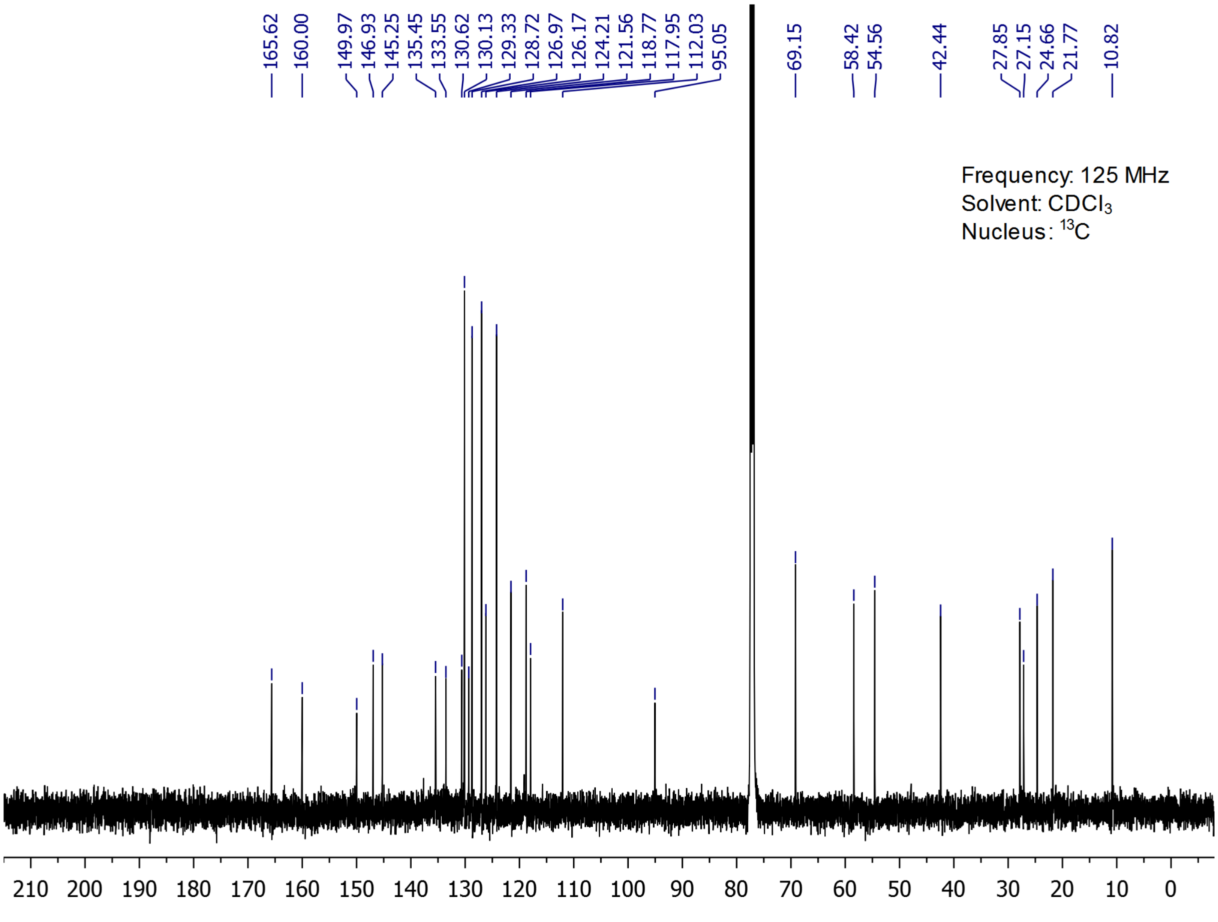
165.62
160.00
149.97
146.93
145.25
135.45
133.55
130.62
130.13
129.33
128.72
126.97
126.17
124.21
121.56
118.77
117.95
112.03
95.05
69.15
58.42
54.56
42.44
27.85
27.15
24.66
21.77
10.82
Frequency: 125 MHz
Solvent: CDCl3
Nucleus: 13C
210
200
190
180
170
160
150
140
130
120
110
100
90
80
70
60
50
40
30
20
10
0

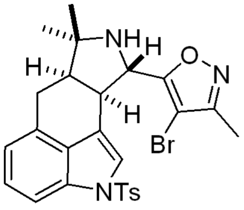
NH
H
O
N
H
H
Br
NTs

34

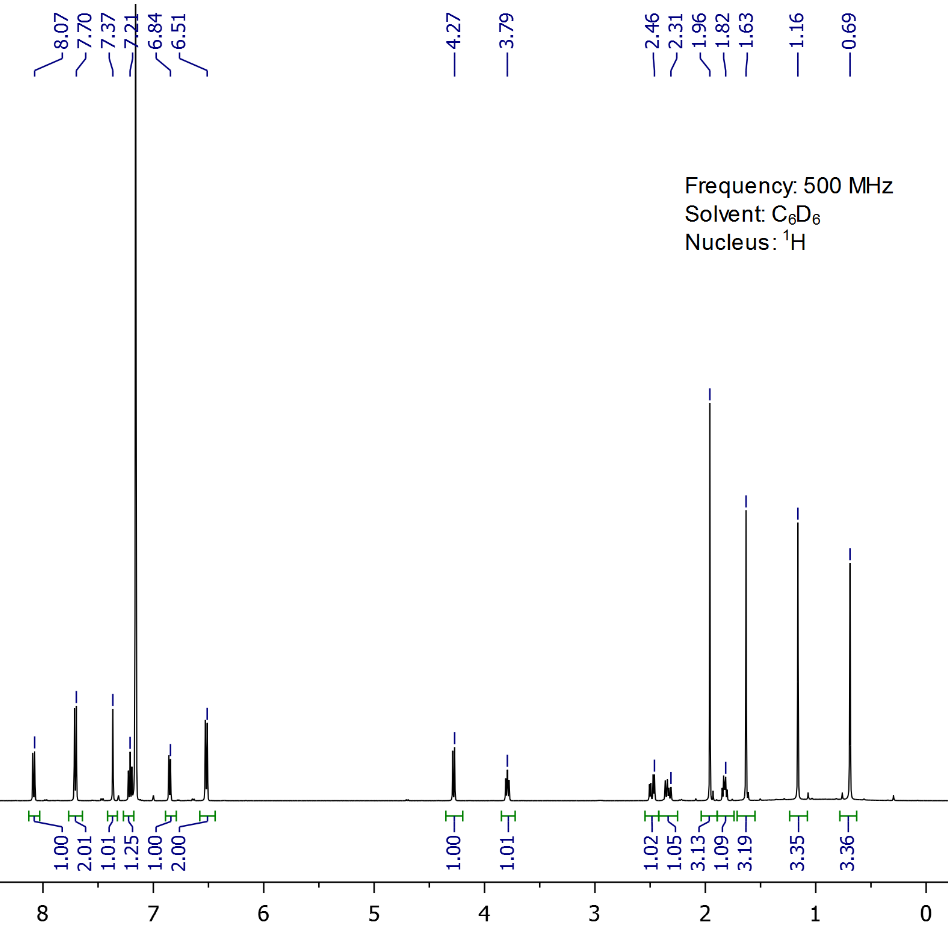
8.07
7.70
7.37
7.21
6.84
6.51
4.27
3.79
2.46
2.31
1.96
1.82
1.63
1.16
0.69
Frequency: 500 MHz
Solvent: C_6D_6
Nucleus: ^1H
1.00
2.01
1.01
1.25
1.00
2.00
1.00
1.01
1.02
1.05
3.13
1.09
3.19
3.35
3.36
10 9 8 7 6 5 4 3 2 1 0

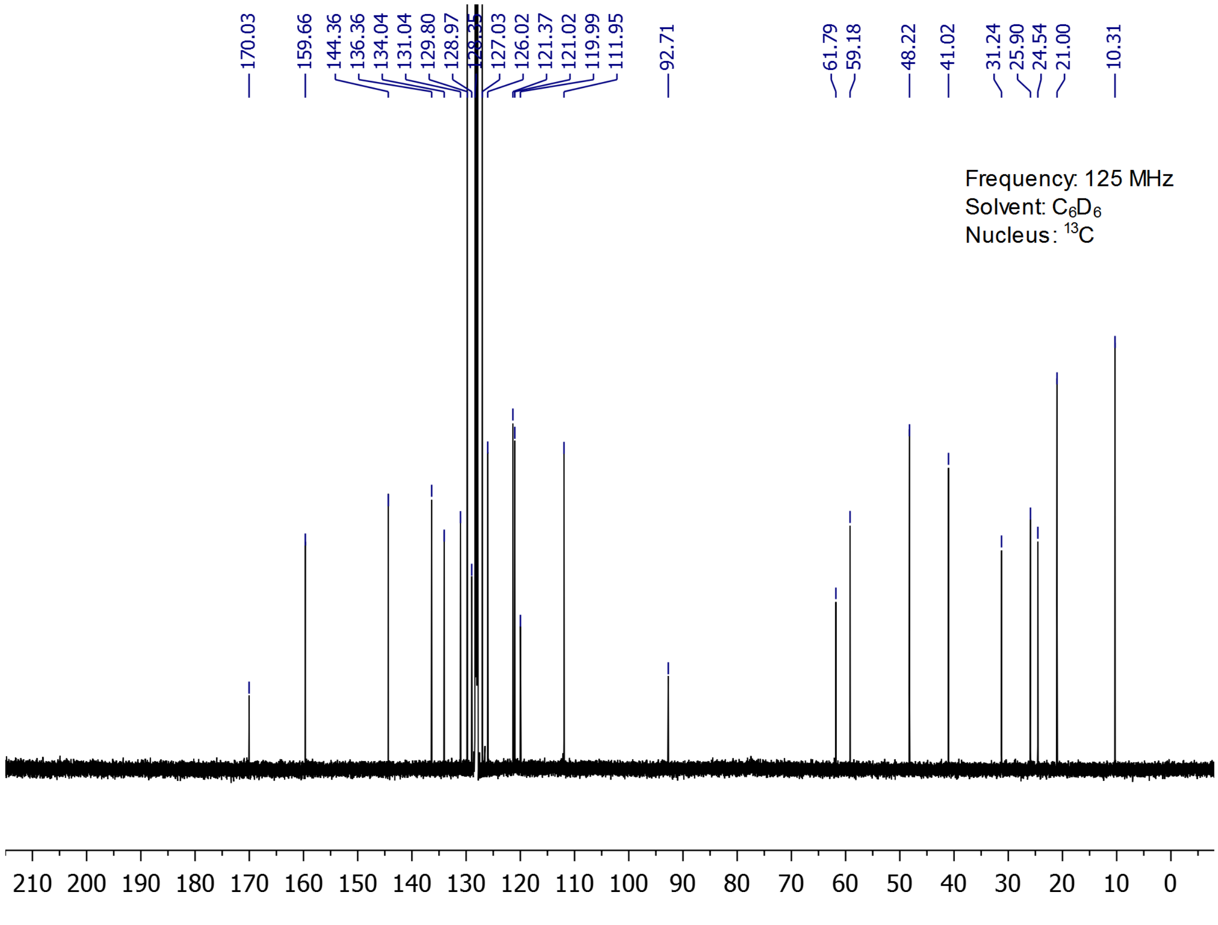
170.03
159.66
144.36
136.36
134.04
131.04
129.80
128.97
128.35
127.03
126.02
121.37
121.02
119.99
111.95
92.71
61.79
59.18
48.22
41.02
31.24
25.90
24.54
21.00
10.31
Frequency: 125 MHz
Solvent: C_6D_6
Nucleus: ^{13}C
210 200 190 180 170 160 150 140 130 120 110 100 90 80 70 60 50 40 30 20 10 0

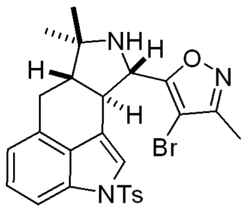
NH
H
O
N
H
H
Br
NTs

iso-**34**

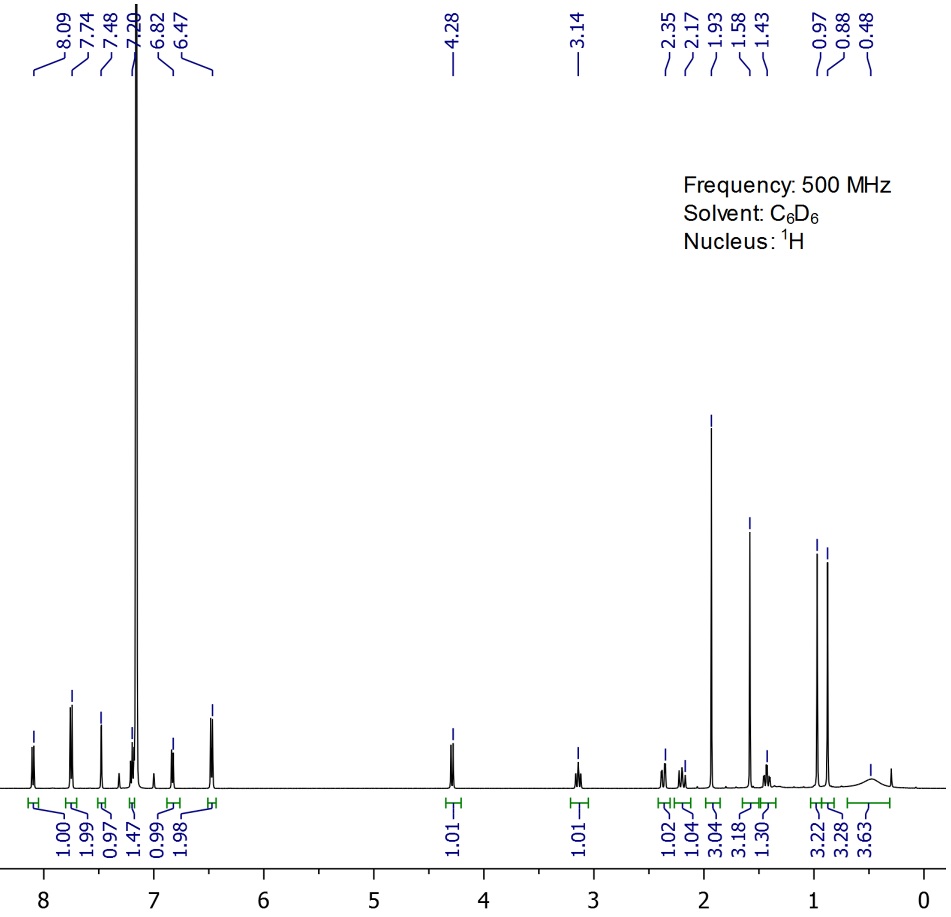
8.09
7.74
7.48
7.20
6.82
6.47
4.28
3.14
2.35
2.17
1.93
1.58
1.43
0.97
0.88
0.48
Frequency: 500 MHz
Solvent: C_6D_6
Nucleus: ^1H
1.00
1.99
0.97
1.47
0.99
1.98
1.01
1.01
1.02
1.04
3.04
3.18
1.30
3.22
3.28
3.63
10
9
8
7
6
5
4
3
2
1
0

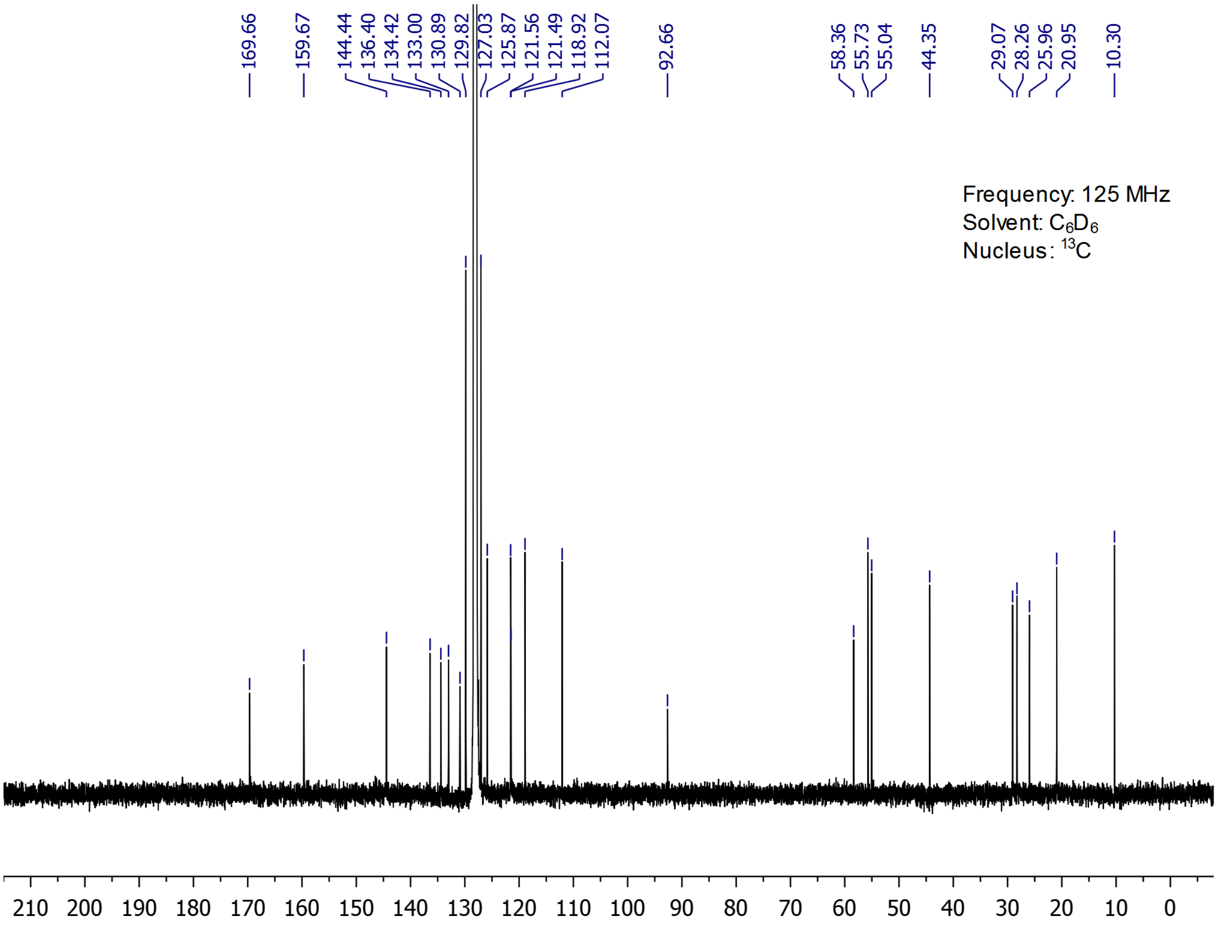
169.66
159.67
144.44
136.40
134.42
133.00
130.89
129.82
127.03
125.87
121.56
121.49
118.92
112.07
92.66
58.36
55.73
55.04
44.35
29.07
28.26
25.96
20.95
10.30
Frequency: 125 MHz
Solvent: C_6D_6
Nucleus: ^{13}C
210
200
190
180
170
160
150
140
130
120
110
100
90
80
70
60
50
40
30
20
10
0

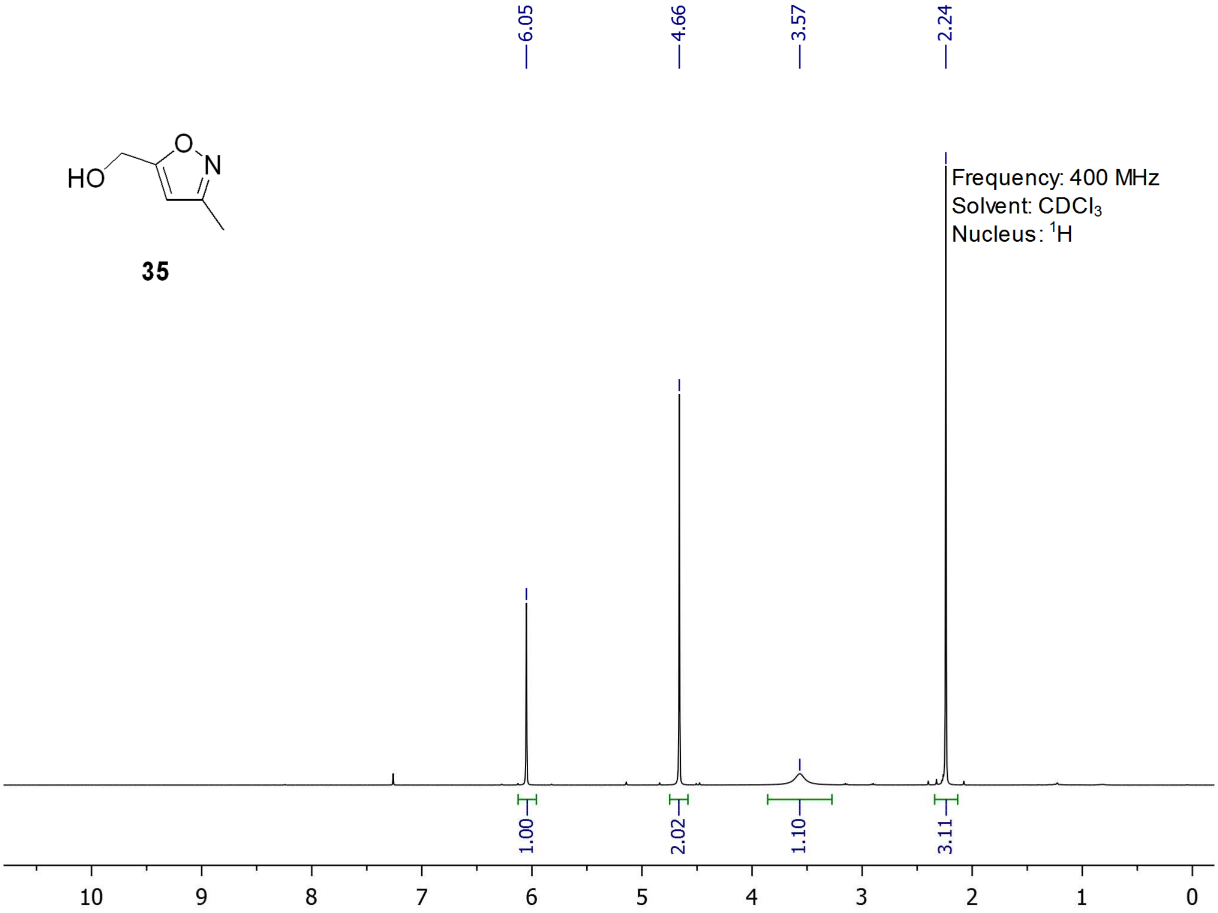

HO
O
N
35
6.05
4.66
3.57
2.24
Frequency: 400 MHz
Solvent: CDCl3
Nucleus: 1H
1.00
2.02
1.10
3.11
10 9 8 7 6 5 4 3 2 1 0

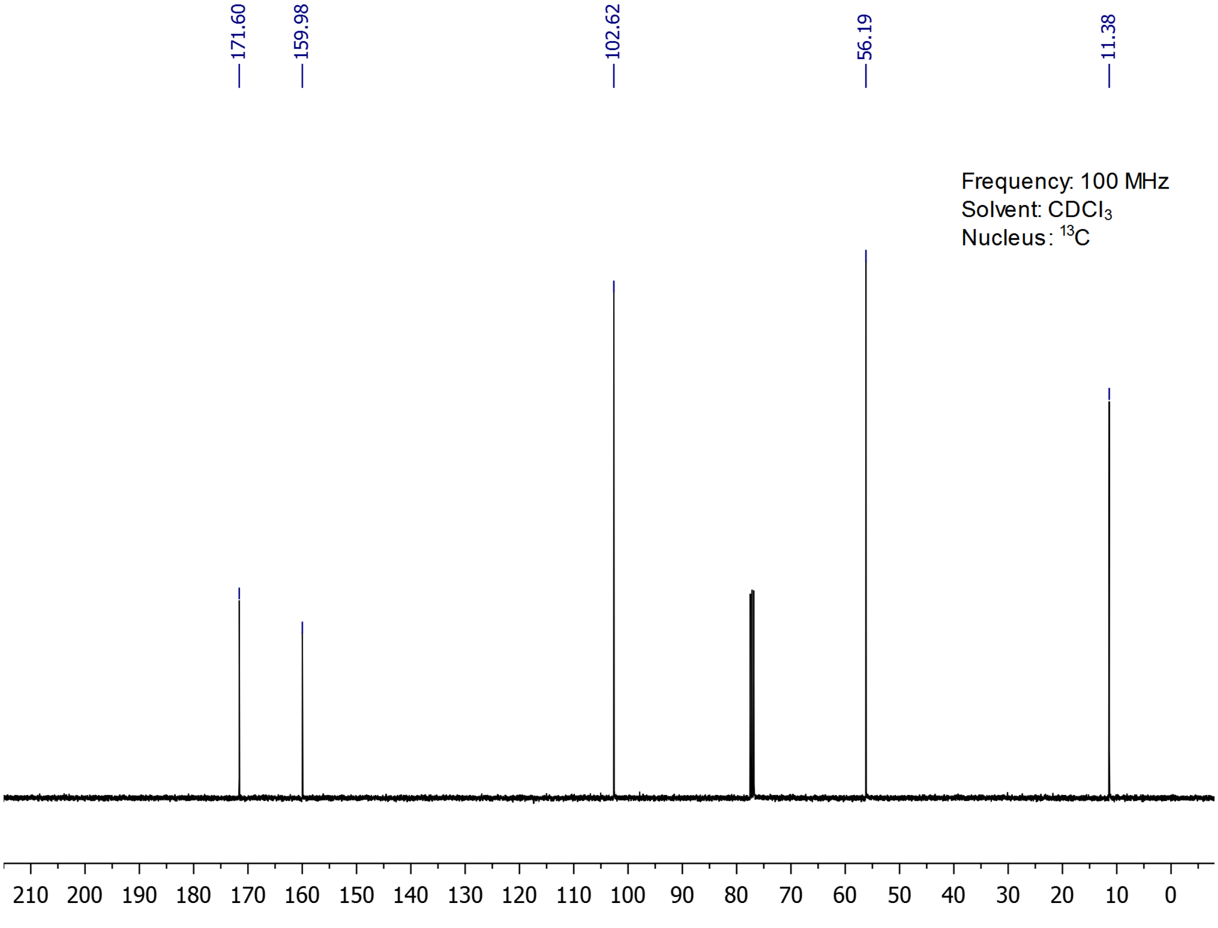

171.60
159.98
102.62
56.19
11.38
Frequency: 100 MHz
Solvent: CDCl3
Nucleus: 13C
210 200 190 180 170 160 150 140 130 120 110 100 90 80 70 60 50 40 30 20 10 0

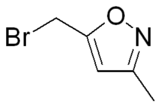
Br
O
N

37

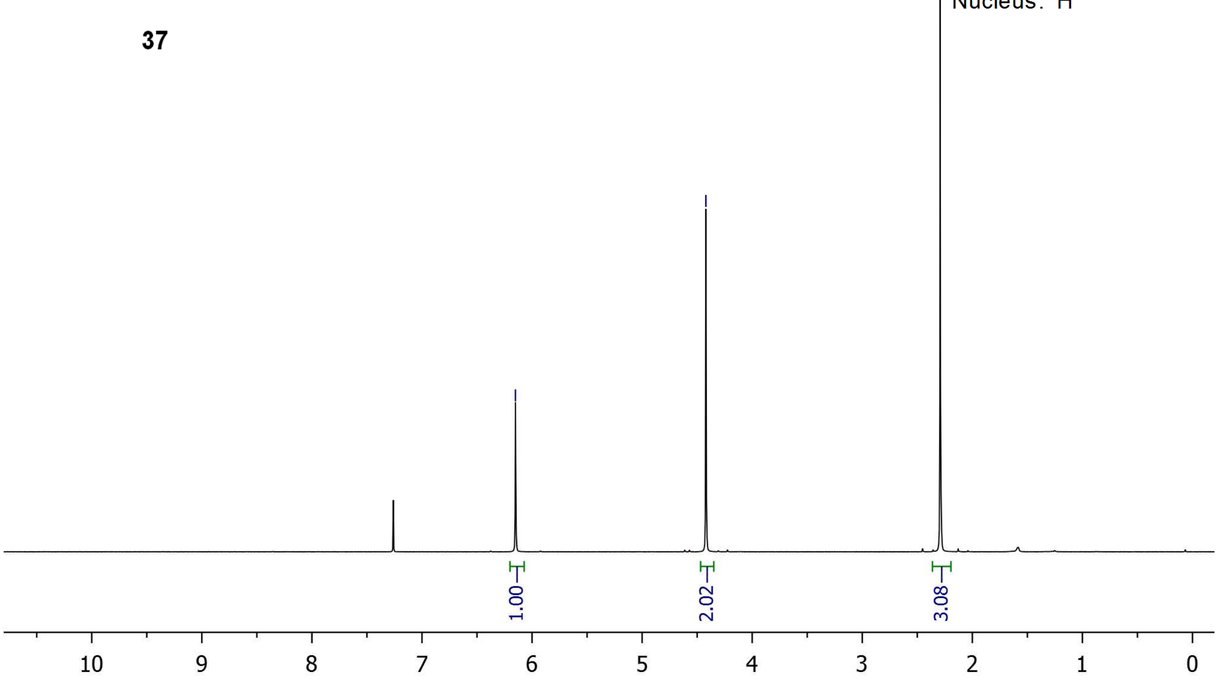
6.15
4.42
2.29
Frequency: 400 MHz
Solvent: CDCl3
Nucleus: 1H
1.00
2.02
3.08
10
9
8
7
6
5
4
3
2
1
0

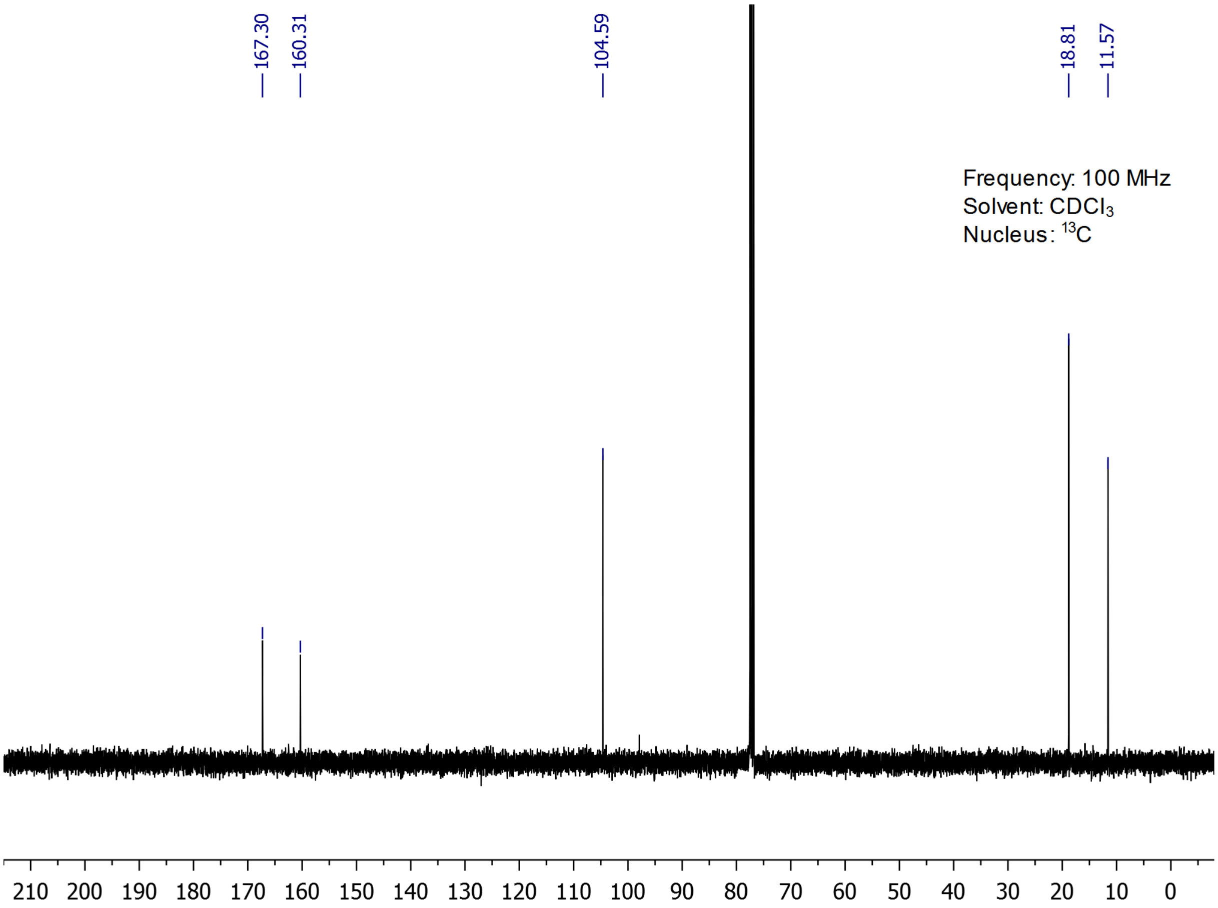
167.30
160.31
104.59
18.81
11.57
Frequency: 100 MHz
Solvent: CDCl3
Nucleus: 13C
210
200
190
180
170
160
150
140
130
120
110
100
90
80
70
60
50
40
30
20
10
0

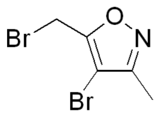
Br
O
N
Br

38

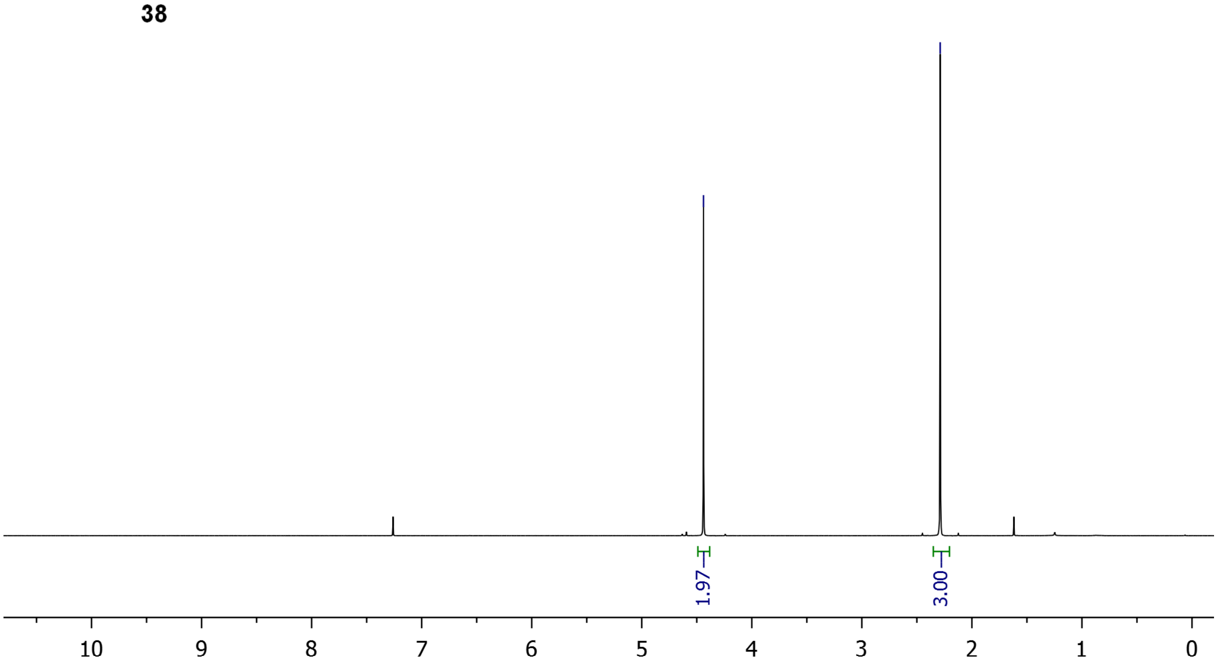
4.44
2.29
Frequency: 400 MHz
Solvent: CDCl3
Nucleus: 1H
1.97
3.00
10
9
8
7
6
5
4
3
2
1
0

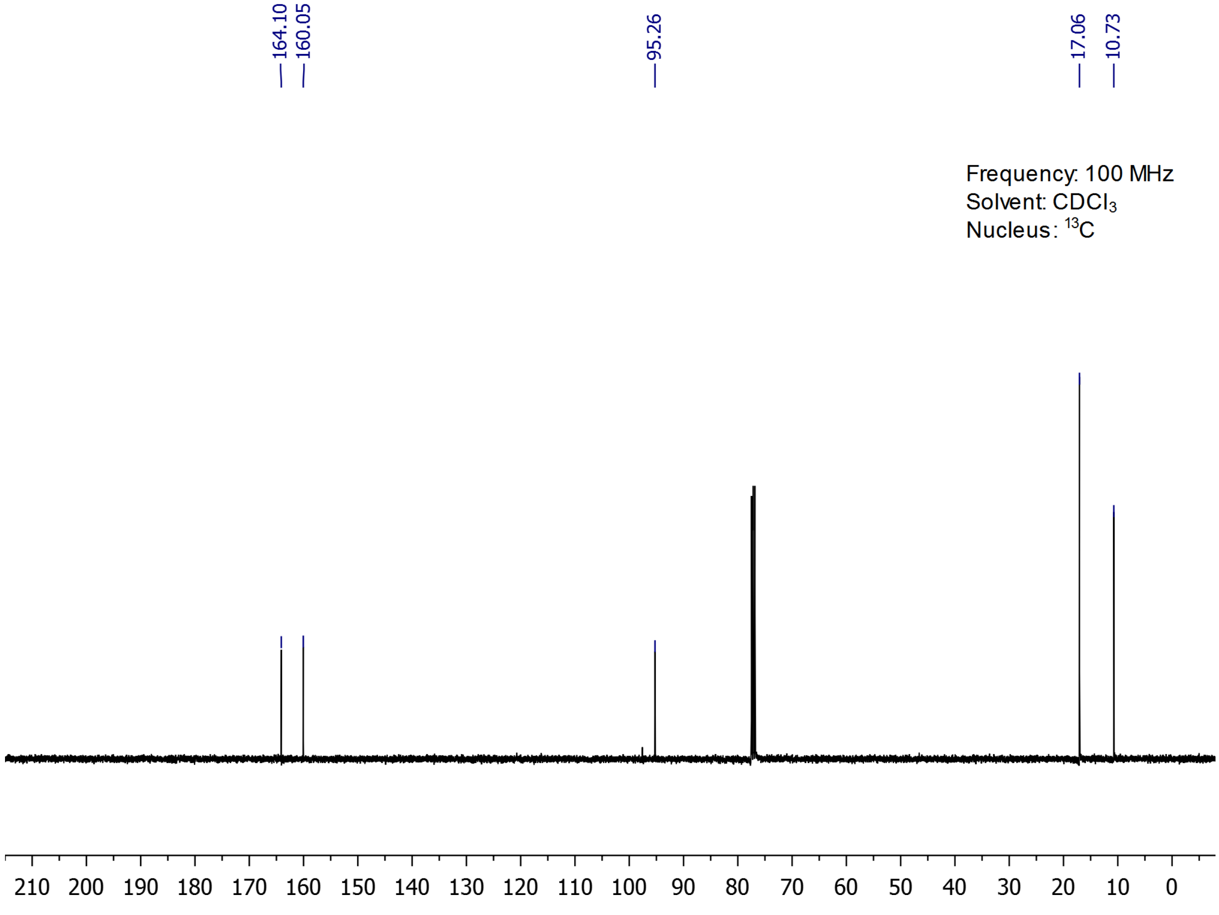
164.10
160.05
95.26
17.06
10.73
Frequency: 100 MHz
Solvent: CDCl3
Nucleus: 13C
210
200
190
180
170
160
150
140
130
120
110
100
90
80
70
60
50
40
30
20
10
0

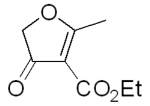
O
CO2Et

42

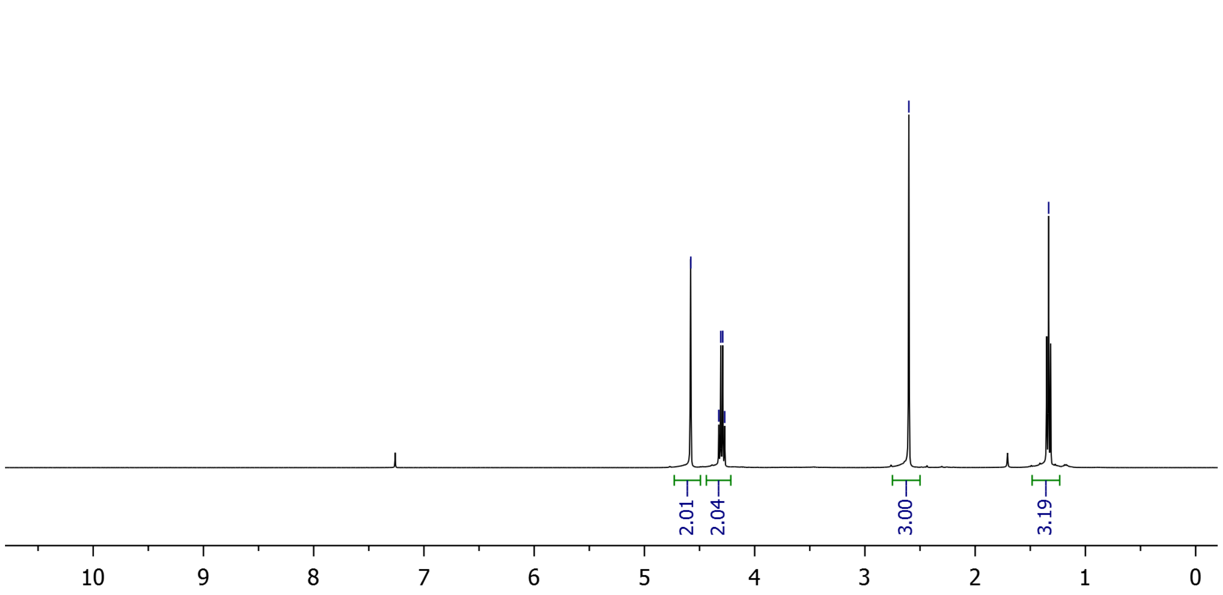
4.58
4.33
4.31
4.29
4.27
2.60
1.33
Frequency: 400 MHz
Solvent: CDCl3
Nucleus: 1H
2.01
2.04
3.00
3.19
10
9
8
7
6
5
4
3
2
1
0

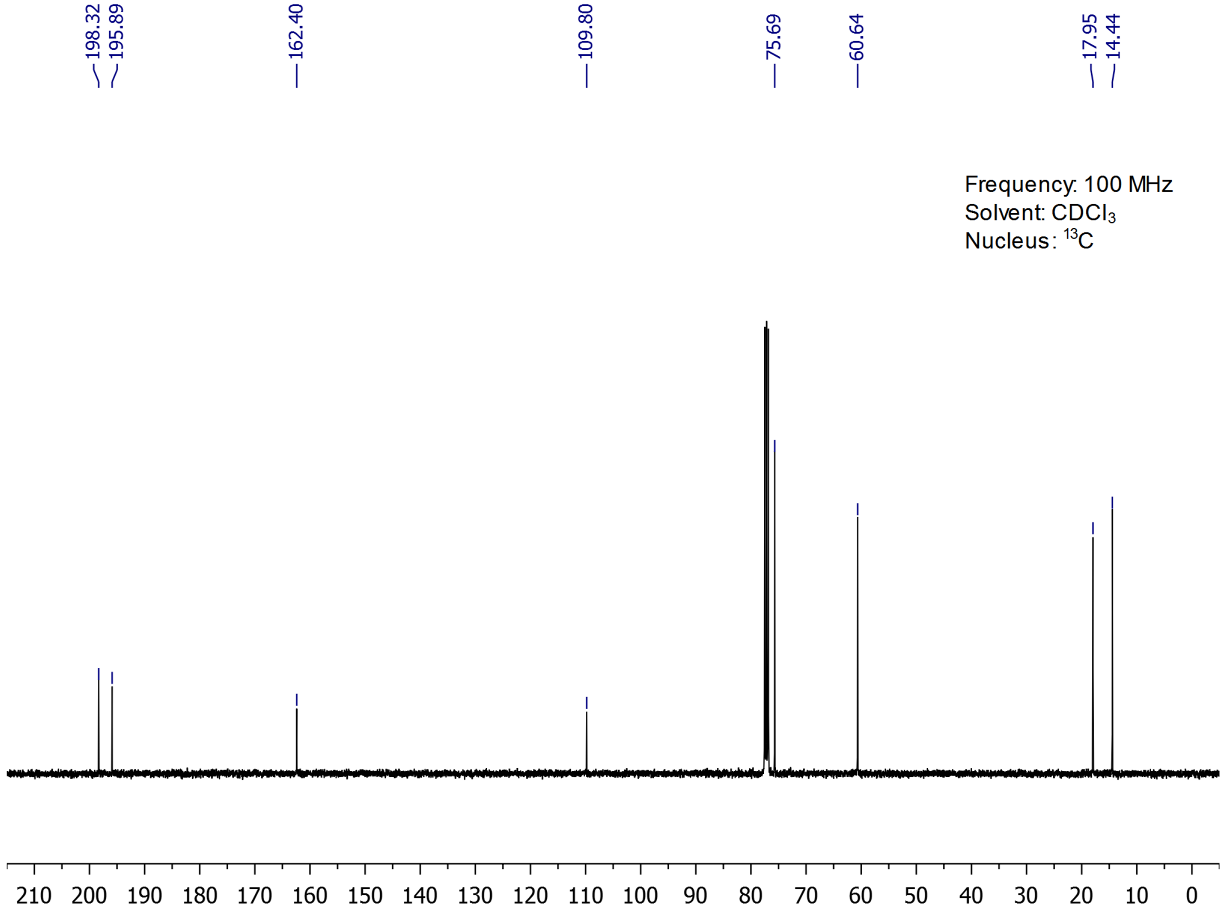
198.32
195.89
162.40
109.80
75.69
60.64
17.95
14.44
Frequency: 100 MHz
Solvent: CDCl3
Nucleus: 13C
210
200
190
180
170
160
150
140
130
120
110
100
90
80
70
60
50
40
30
20
10
0

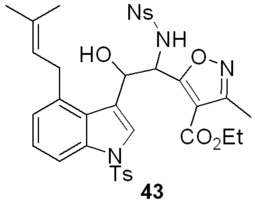
Ns
NH
HO
O
N
CO2Et
N
Ts

43

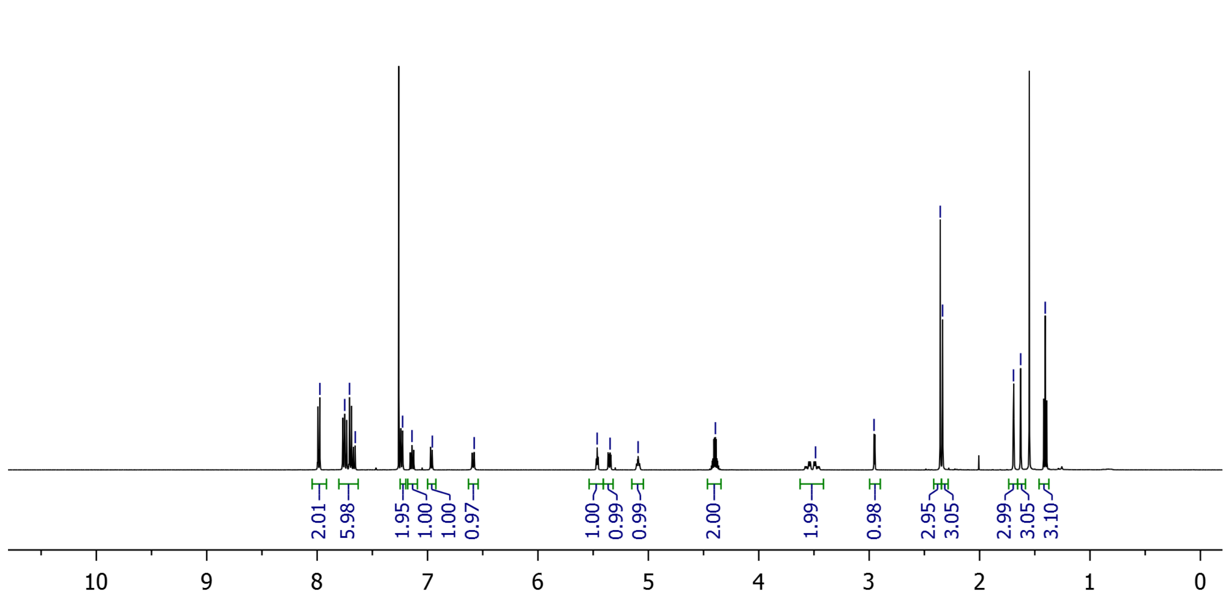
7.98
7.75
7.71
7.65
7.23
7.14
6.96
6.58
5.46
5.35
5.09
4.39
3.49
2.96
2.36
2.34
1.69
1.63
1.41
Frequency: 500 MHz
Solvent: CDCl3
Nucleus: 1H
2.01
5.98
1.95
1.00
1.00
0.97
1.00
0.99
0.99
2.00
1.99
0.98
2.95
3.05
2.99
3.05
3.10
10
9
8
7
6
5
4
3
2
1
0

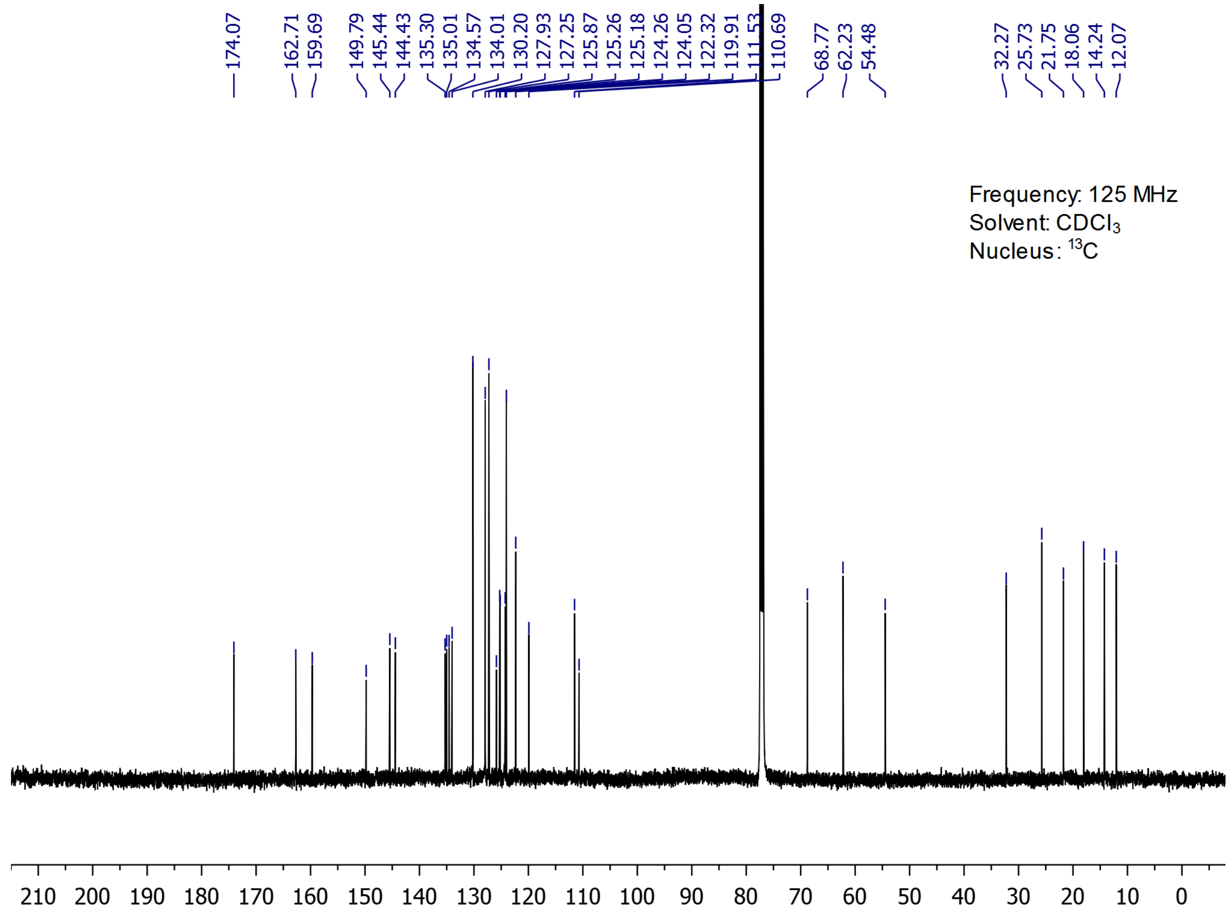
174.07
162.71
159.69
149.79
145.44
144.43
135.30
135.01
134.57
134.01
130.20
127.93
127.25
125.87
125.26
125.18
124.26
124.05
122.32
119.91
111.53
110.69
68.77
62.23
54.48
32.27
25.73
21.75
18.06
14.24
12.07
Frequency: 125 MHz
Solvent: CDCl3
Nucleus: 13C
210
200
190
180
170
160
150
140
130
120
110
100
90
80
70
60
50
40
30
20
10
0

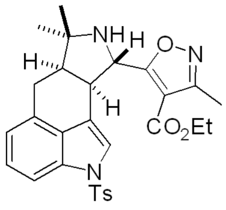
NH
H
O
N
H
H
CO2Et
N
Ts

44

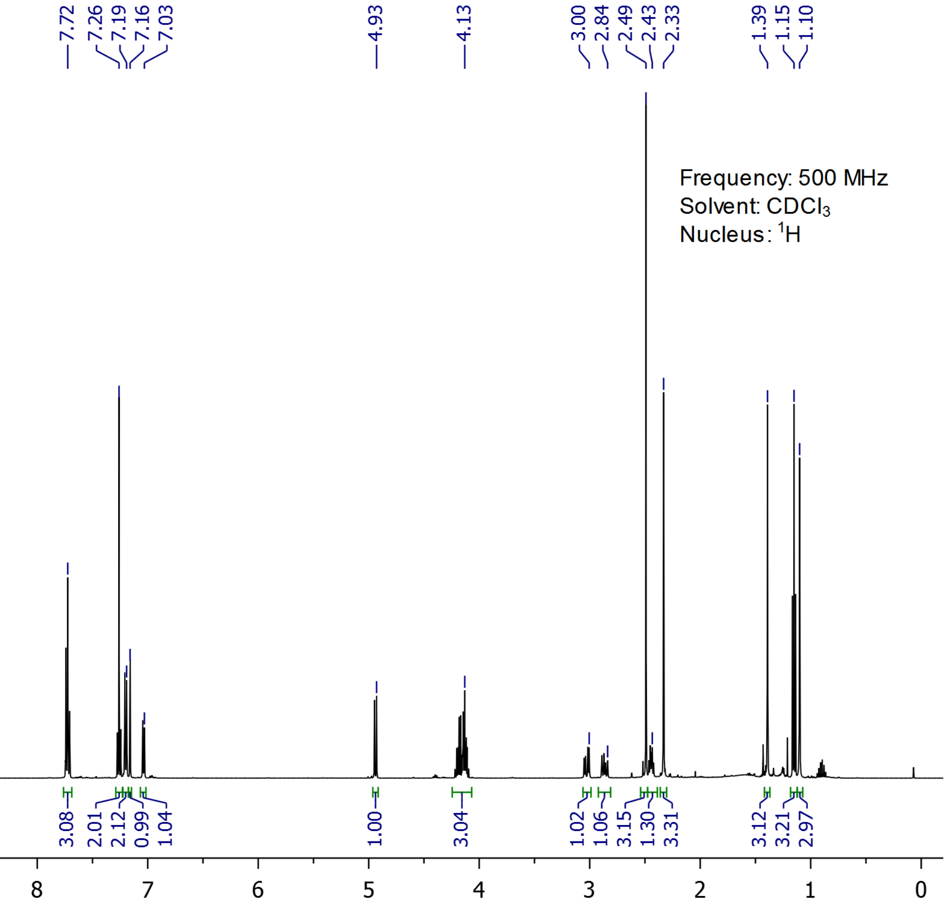
7.72
7.26
7.19
7.16
7.03
4.93
4.13
3.00
2.84
2.49
2.43
2.33
1.39
1.15
1.10
Frequency: 500 MHz
Solvent: CDCl3
Nucleus: 1H
3.08
2.01
2.12
0.99
1.04
1.00
3.04
1.02
1.06
3.15
1.30
3.31
3.12
3.21
2.97
10 9 8 7 6 5 4 3 2 1 0

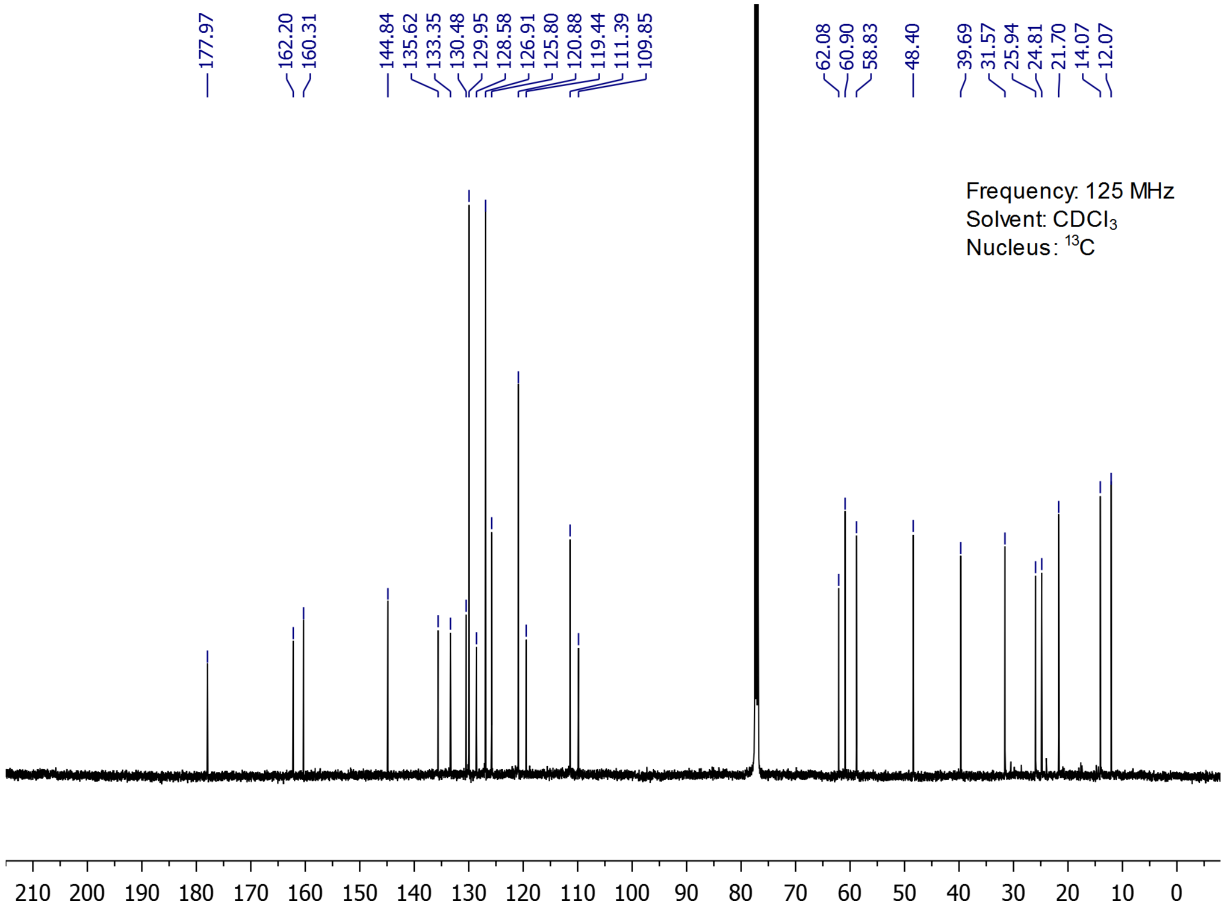
177.97
162.20
160.31
144.84
135.62
133.35
130.48
129.95
128.58
126.91
125.80
120.88
119.44
111.39
109.85
62.08
60.90
58.83
48.40
39.69
31.57
25.94
24.81
21.70
14.07
12.07
Frequency: 125 MHz
Solvent: CDCl3
Nucleus: 13C
210 200 190 180 170 160 150 140 130 120 110 100 90 80 70 60 50 40 30 20 10 0

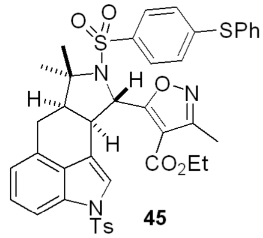
SPh
CO2Et
N
Ts
O
S
H
N

45

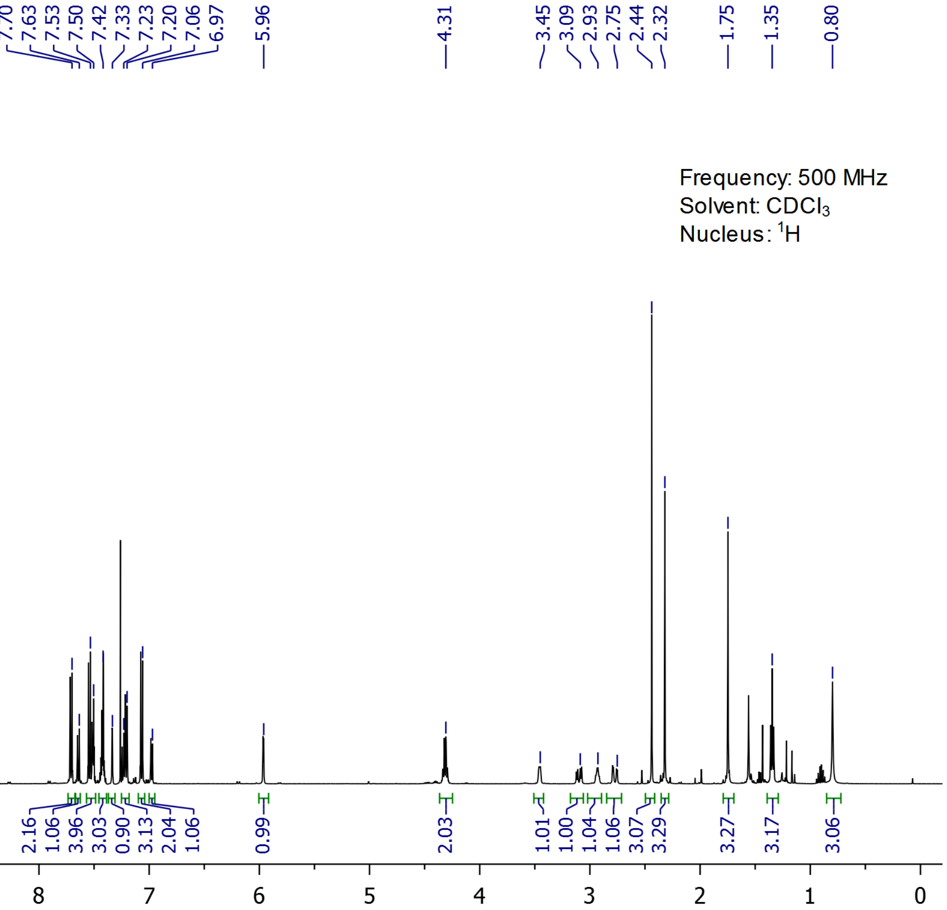
7.70
7.63
7.53
7.50
7.42
7.33
7.23
7.20
7.06
6.97
5.96
4.31
3.45
3.09
2.93
2.75
2.44
2.32
1.75
1.35
0.80
Frequency: 500 MHz
Solvent: CDCl3
Nucleus: 1H
2.16
1.06
3.96
3.03
0.90
3.13
2.04
1.06
0.99
2.03
1.01
1.00
1.04
1.06
3.07
3.29
3.27
3.17
3.06
10
9
8
7
6
5
4
3
2
1
0

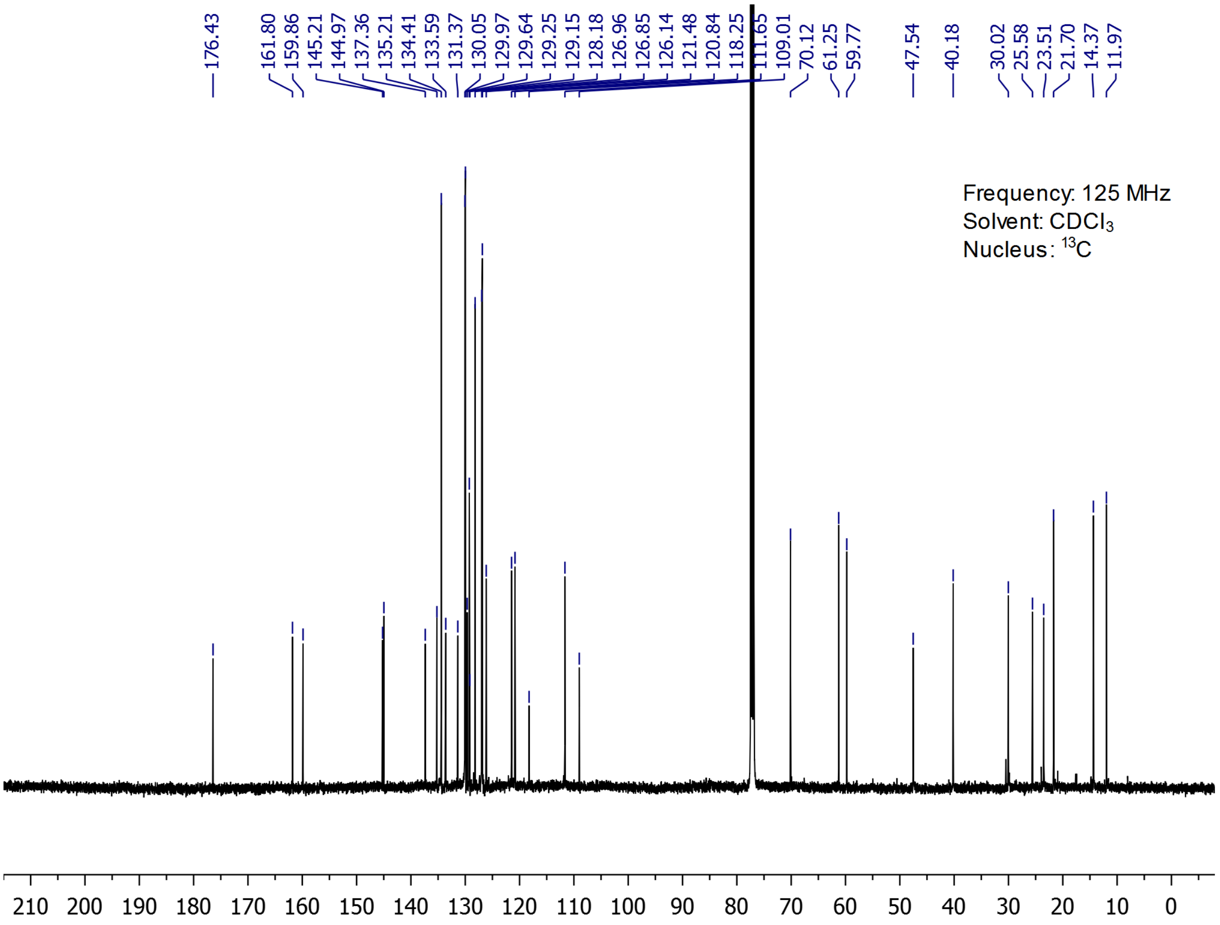
176.43
161.80
159.86
145.21
144.97
137.36
135.21
134.41
133.59
131.37
130.05
129.97
129.64
129.25
129.15
128.18
126.96
126.85
126.14
121.48
120.84
118.25
111.65
109.01
70.12
61.25
59.77
47.54
40.18
30.02
25.58
23.51
21.70
14.37
11.97
Frequency: 125 MHz
Solvent: CDCl3
Nucleus: 13C
210
200
190
180
170
160
150
140
130
120
110
100
90
80
70
60
50
40
30
20
10
0
